# Magnesium Pincer Complexes and Their Applications in Catalytic Semihydrogenation of Alkynes and Hydrogenation of Alkenes: Evidence for Metal–Ligand Cooperation

**DOI:** 10.1021/jacs.2c08491

**Published:** 2022-10-04

**Authors:** Yaoyu Liang, Uttam Kumar Das, Jie Luo, Yael Diskin-Posner, Liat Avram, David Milstein

**Affiliations:** † Department of Molecular Chemistry and Materials Science, Weizmann Institute of Science, Rehovot 7610001, Israel; ‡ Department of Chemical Research Support, Weizmann Institute of Science, Rehovot 7610001, Israel

## Abstract

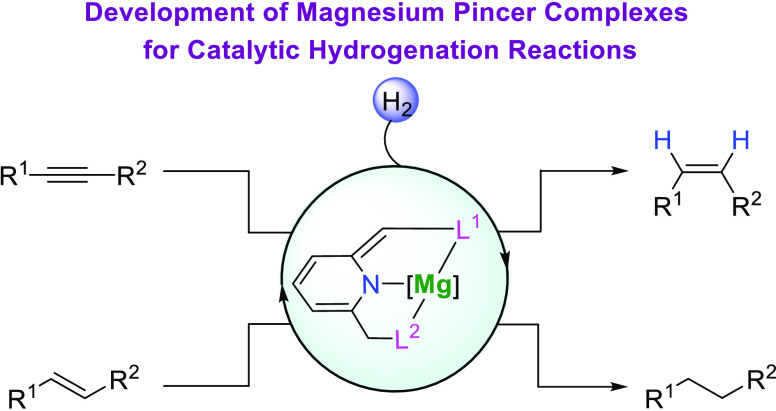

The development of catalysts for environmentally benign organic transformations is a very active area of research. Most of the catalysts reported so far are based on transition-metal complexes. In recent years, examples of catalysis by main-group metal compounds have been reported. Herein, we report a series of magnesium pincer complexes, which were characterized by NMR and X-ray single-crystal diffraction. Reversible activation of H_2_ via aromatization/dearomatization metal–ligand cooperation was studied. Utilizing the obtained complexes, the unprecedented homogeneous main-group metal catalyzed semihydrogenation of alkynes and hydrogenation of alkenes were demonstrated under base-free conditions, affording *Z*-alkenes and alkanes as products, respectively, with excellent yields and selectivities. Control experiments and DFT studies reveal the involvement of metal–ligand cooperation in the hydrogenation reactions. This study not only provides a new approach for the semihydrogenation of alkynes and hydrogenation of alkenes catalyzed by magnesium but also offers opportunities for the hydrogenation of other compounds catalyzed by main-group metal complexes.

## Introduction

The importance of catalysts in organic and inorganic transformations is indisputable. Currently, the most commonly used catalysts are derived from transition metals due to their efficiency in substrate activation and reaction acceleration.^1−3^ However, the costly and, in some cases, toxic characteristics of transition-metal compounds have become significant driving forces to explore alternative catalysts. In this respect, the earth-abundant, nontoxic, and environmentally friendly main-group metals have received increasing interest in recent years.^4−10^ Apart from some applications in Lewis acid catalysis, their catalytic activity has been demonstrated in hydroamination,^11−14^ hydrosilylation,^15−19^ hydroboration,^20−24^ hydrogenation,^25−32^ dehydrocoupling,^33−36^ and polymerization reactions.^37,38^ Nevertheless, the absence of partially filled d-orbitals prevented reversible changes in the oxidation states, such as in oxidative addition–reductive elimination processes, thus resulting in limitations in bond activation. In addition, the high reactivity of various main-group metal compounds can often lead to uncontrollable side reactions that largely impede their wide application in catalysis. To overcome these problems, sterically demanding ligands, such as β-diketiminate,^37,39^ tris(oxazolinyl)-borate,^40^ silylamido phenolate,^38^ aminotroponiminates,^14^ pybox,^41^ and benzimidazole,^19^ have been introduced to stabilize the complexes and provide distinctive bond activation modes, thus making a variety of catalytic reactions feasible. Despite progress, the development of main-group metal complexes with distinct bond activation strategies to broaden their application in catalysis is still highly desirable.

**Figure 1:**
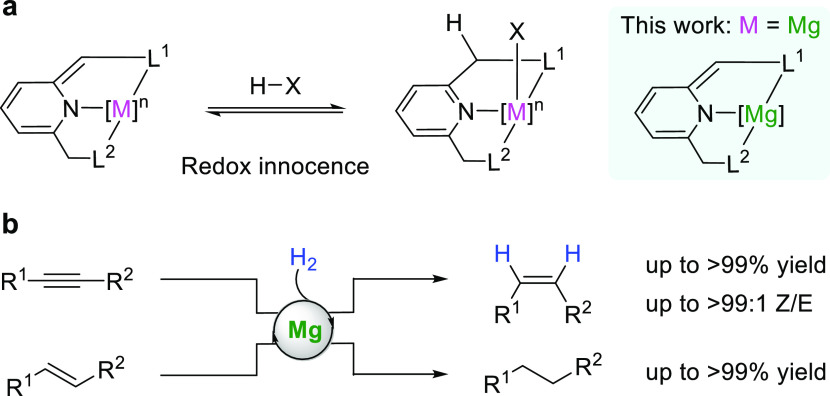
Overview of this work. (a) Metal–ligand cooperation by the aromatization/dearomatization of pincer complexes for reversible bond activation and the development of Mg pincer complexes. (b) Application of magnesium pincer complexes in semihydrogenation of alkynes and hydrogenation of alkenes.

Metal–ligand cooperation (MLC) involving the aromatization/dearomatization of pincer-type complexes is a versatile strategy for chemical bond activation (Figure 1a).^42−46^ One significant feature of this process is that the oxidation state of the metal center does not change since the bond activation process occurs across both the metal and ligand. Employing this activation mode, the redox-innocent main-group metals can favorably activate chemical bonds without changing their oxidation state. However, the bond activation via the MLC process has been dominated by costly and precious transition metals.^42−44^ In the last few years, increasing attention has been paid to the cheaper first-row transition metals,^45,46^ such as Fe, Mn, and Co. With respect to the application of main-group metals, it is still in its infancy compared to that of transition metals.^47−51^ In 2016, potassium and lithium dearomatized pincer complexes were synthesized and characterized by Danopoulos and co-workers, but activity studies have not been demonstrated.^50^ Recently, zinc dearomatized complexes were prepared supported by a (PNP)^*t*^Bu pincer ligand and applied to N–H and H–H bond activation via the MLC process.^51^ Inspired by these significant advances, we envisioned designing new complexes with other abundant main-group metals, such as magnesium, and extending the main-group metal complexes to more challenging catalytic reactions by employing the MLC activation process.

Catalytic semihydrogenation of alkynes is an important reaction in organic chemistry.^52^ Homogeneous catalysts using transition metals^53−60^ and frustrated Lewis pairs^61−63^ have been developed in recent years to efficiently access *Z*- or *E*-alkenes. On the contrary, homogeneous main-group metal-catalyzed semihydrogenation of alkynes has never been reported. Indeed, the main-group metal-catalyzed hydrogenation of unsaturated C–C bonds using H_2_ is rare,^25−31,64,65^ presumably because of the challenging H–H bond activation. In 2008, Harder and co-workers reported the hydrogenation of conjugated alkenes by alkaline earth metal complexes under relatively mild conditions, indicating that the early main-group metal-catalyzed hydrogenation of C–C unsaturated bonds is possible.^25^ In continuation of our work on the activation of small molecules by MLC,^66−69^ we hypothesized that dearomatized magnesium complexes may activate H_2_ via the same MLC process as that of transition metals, perhaps leading to the semihydrogenation of alkynes catalyzed by main-group metals.

Herein, we report a series of magnesium pincer complexes based on PNP-, PNN-, and NNN-type ligands, prepared under mild conditions and characterized by NMR and X-ray crystallography. These complexes undergo H–H bond activation via the MLC process without changing the metal oxidation state. The new complexes are applied to the catalytic semihydrogenation of alkynes, generating *Z*-alkenes with up to >99% yield and >99:1 stereoselectivity (Figure 1b). The complexes also catalyze the hydrogenation of alkenes under similar reaction conditions. A possible mechanism clearly demonstrating the catalytic process is proposed based on control experiments and DFT studies.

## Results and Discussion

### Preparation and Characterization of Magnesium Pincer Complexes

**Figure 2:**
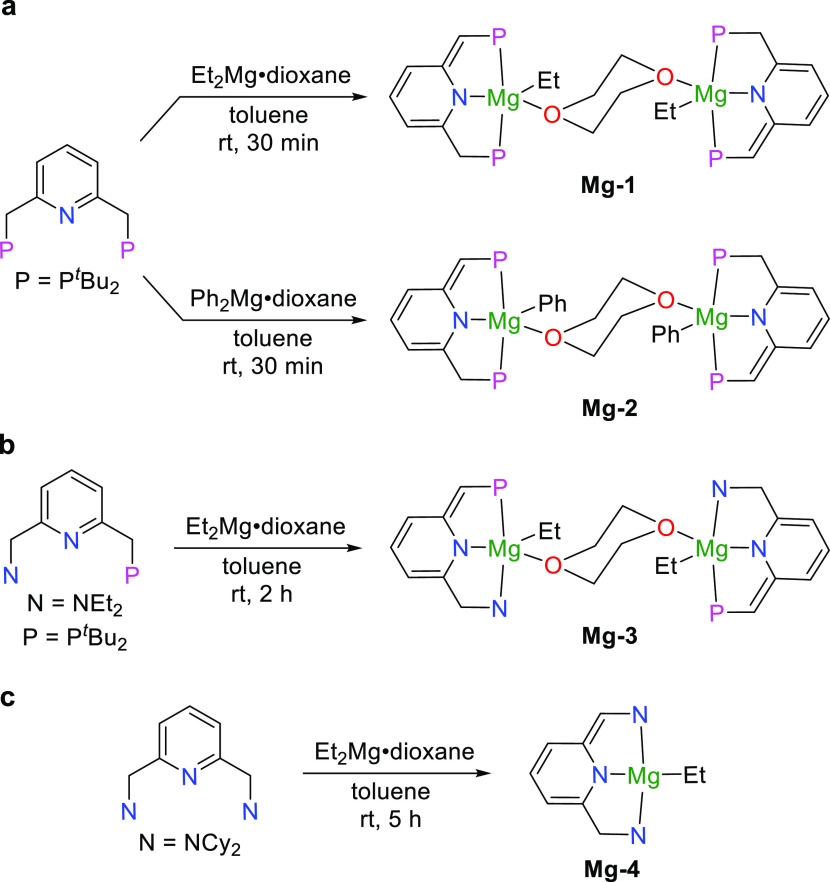
Synthesis of magnesium pincer complexes. (a) Synthesis of **Mg-1** and **Mg-2** with a PNP-type ligand. (b) Synthesis of **Mg-3** with a PNN-type ligand. (c) Synthesis of **Mg-4** with an NNN-type ligand.

**Figure 3:**
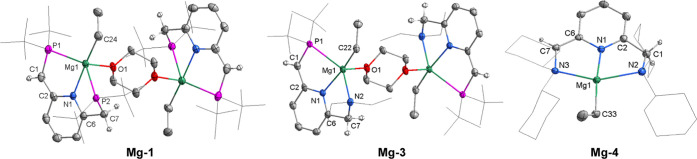
Crystal structures of complexes **Mg-1** (left), **Mg-3** (middle), and **Mg-4** (right). Selected hydrogen atoms are omitted for clarity. Some groups displayed are as wireframes for clarity. Selected bond lengths (Å) and angles (deg): (**Mg-1**) Mg1–P1 2.656(2), Mg1–P2 2.928(2), Mg1–N1 2.140(5), Mg1–O1 2.171(4), Mg1–C24 2.155(6), C1–P1 1.770(7), C1–C2 1.393(9), C7–P2 1.852(6), C6–C7 1.509(8), N1–C2 1.404(7), N1–C6 1.363(7), C24–Mg1–N1 165.5(2), and C24–Mg1–O1 100.5(2). (**Mg-3**) Mg1–P1 2.7516(6), Mg1–N1 2.1440(12), Mg1–N2 2.3011(13), Mg1–C22 2.1783(14), Mg1–O1 2.1288(10), C1–C2 1.382(2), C6–C7 1.510(2), and C22–Mg1–O1 104.98(5). (**Mg-4**) Mg1–N1 2.0333(12), Mg1–N2 2.4354(12), Mg1–N3 2.2943(11), Mg1–C33 2.1507(14), C1–C2 1.5023(18), C6–C7 1.3658(18), and N1–Mg1–C33 173.71(5).

The dearomatized magnesium complex **Mg-1** was prepared by the reaction of 2,6-bis(di-*tert*-butylphosphinomethyl)pyridine and Et_2_Mg·dioxane in toluene at room temperature (Figure 2a, top). The color of the solution changed from colorless to yellow after stirring for 10 min, indicating product formation. Unlike the preparation of dearomatized transition-metal pincer complexes that require an added base to deprotonate the side arm,^70−72^ in this case, deprotonation occurs by the ethyl ligand, generating ethane as the only byproduct. The ^31^P{^1^H} NMR spectrum of **Mg-1** exhibits two characteristic doublets (*J* = 3.1 Hz) at 23.54 and −2.67 ppm, indicating two different phosphorus nuclei. The small coupling constant indicates long Mg–P bonds. The ^1^H NMR spectrum exhibits three resonances for the pyridine backbone (6.44, 6.23, and 5.56 ppm), with similar shifts as in the case of the known lithium,^50^ potassium,^50^ and zinc compounds,^51^ suggesting the formation of an analogous dearomatized structure. A doublet at 3.59 ppm (*J* = 4.7 Hz, 1H) and a singlet at 2.55 ppm (2H) are assigned to the methine (C*H*) and methylene (C*H*_2_) groups of the side arm, respectively, indicating the formation of a dearomatized complex. Integration of the peaks suggests that the complex is formed as a dimer bridged by dioxane. Single crystals of **Mg-1** suitable for X-ray diffraction studies were grown at −32 °C using a mixed solvent of benzene and pentane. X-ray crystallography further confirmed the structure of five-coordinate **Mg-1** (Figure 3, left). The X-ray structure exhibits a dimer of two magnesium pincer complexes, supporting the observation by NMR. The magnesium center and (PNP)^*t*^Bu ligand form a distorted plane, which results in the different bond lengths of Mg1–P1 (2.66 Å) and Mg1–P2 (2.93 Å). As such, it is understandable that the ^31^P{^1^H} NMR spectrum of **Mg-1** exhibits two doublets with a small coupling constant. The dearomatized structure is clearly confirmed by the considerably shorter C1–C2 bond (1.39 Å) compared to the C6–C7 bond (1.51 Å). The result also matches the observation by NMR. Following a similar procedure using Ph_2_Mg·dioxane as a reactant, **Mg-2** was obtained with a phenyl group coordinated to the magnesium center (Figure 2a, bottom). The ^31^P{^1^H} NMR spectrum shows two singlets at a higher field (16.41 and −6.34 ppm) compared to that of **Mg-1**. The larger steric hindrance of the phenyl group compared to the ethyl group possibly resulted in longer Mg–P bonds, which led to the formation of singlet phosphorus resonances. The ^1^H NMR spectrum also exhibits characteristic methine (C*H*) and methylene (C*H*_2_) peaks of the side arm, which confirm the dearomatized structure. NMR signals assigned to the phenyl group are observed, confirming the presence of the phenyl ligand. Similar chemical shifts and integration are observed in the ^1^H NMR spectrum, indicating the formation of a structure similar to **Mg-1**.

Mixing the PNN-type ligand (PNN = 6-(di-*tert*-butylphosphinomethylene)-2-(*N*,*N*-diethylaminomethyl)-1,6-dihydropyridine) and Et_2_Mg·dioxane at room temperature for 2 h resulted in the formation of **Mg-3** as an orange solid after removing the excess solvent (Figure 2b). The ^31^P{^1^H} NMR spectrum exhibits a singlet at 0.43 ppm, suggesting the generation of a single product. A dearomatized pyridine backbone was observed in the ^1^H NMR spectrum, showing similar chemical shifts to those of **Mg-1** and **Mg-2**. Complex **Mg-3** was also formed as a dimer with two (PNN)MgEt units bridged by dioxane, as displayed by its X-ray structure (Figure 3, middle). Similar to **Mg-1**, the PNN ligand and magnesium center exhibit a distorted plane due to steric hindrance. The Mg1–P1 bond length (2.75 Å) is slightly longer than that of **Mg-1** (2.66 Å).

The successful preparation of **Mg-3** indicates that tertiary amines are proper ligands for dearomatized magnesium complexes. Thus, **Mg-4** was prepared to investigate its structure and activity using an NNN-type ligand (Figure 2c). The complex **Mg-4** exhibits three signals at 6.41, 5.97, and 4.95 ppm for the pyridine protons in the ^1^H NMR spectrum. These signals shifted to low frequencies, signifying the formation of a dearomatized NNN ligand. Unlike the other complexes described above, dioxane is not coordinated in this case, possibly because of the steric hindrance of the bulky substituents on the amines. This indicates that the four-coordinate magnesium complex is stable, which is in accord with reports in the literature.^37,40^ Single crystals of **Mg-4** suitable for X-ray diffraction studies were obtained by slow evaporation of a mixed solvent of Et_2_O and pentane at room temperature. The structure reveals a slightly distorted plane geometry (Figure 3, right). The angle between the ethyl group and amine of the pyridine ring is 173.7° (N1–Mg1–C33). The bond length of Mg1–N2 (2.44 Å) is longer than Mg1–N3 (2.29 Å). The different bond lengths of C1–C2 (1.50 Å) and C6–C7 (1.37 Å) further confirm the dearomatized structure of **Mg-4**.

### Hydrogen Activation by Magnesium Pincer Complexes

**Figure 4:**
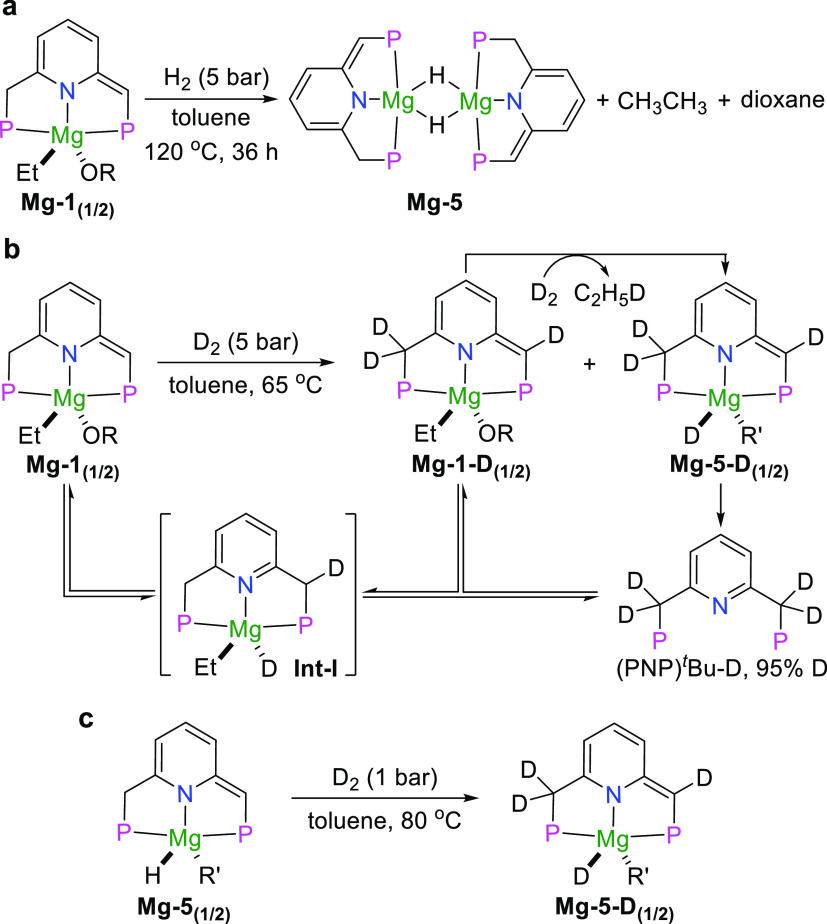
Reaction of magnesium complexes with H_2_ and D_2_. (a) Reaction of **Mg-1** with H_2_. (b) Reaction of **Mg-1** with D_2_ and the possible pathway of reversible D_2_ activation. (c) Reaction of **Mg-5** with D_2_.

Upon treatment of a toluene solution of **Mg-1** in a J. Young NMR rotating tube with 5 bar of H_2_ at room temperature for 24 h, only a trace amount of decomposition was observed by ^31^P{^1^H} NMR (Figure S34). After heating at 65 °C for another 36 h, two new broad peaks at 31.77 and −3.14 ppm were observed by ^31^P{^1^H} NMR, in addition to the free ligand resulting from partial decomposition (Figure S35). Increasing the temperature to 120 °C, **Mg-1** was fully transformed into a new species and free ligand after 36 h (Figure 4a). The ^1^H NMR spectrum of the solution exhibited new signals associated with the pyridine ring at a high field, suggesting that the dearomatized structure still remained. Both the dioxane ligand and the ethyl group disappeared and were replaced by a hydride. The hydride signal overlapped with the tertiary butyl groups of the ligand, but upon treatment of **Mg-1** with D_2_, a deuteride signal was observed by ^2^H NMR (*vide infra*). Notably, ethane was detected by ^1^H NMR and GC, in line with the formation of a magnesium hydride species. DOSY NMR experiments suggest that the species is a dimer (Figures S43 and S44), in line with previously reported dimeric magnesium hydride complexes.^73,74^ Finally, the new structure was determined as a dearomatized magnesium hydride **Mg-5**. However, the NMR signals of **Mg-5** gradually disappeared and converted to the free ligand under high temperature and H_2_ pressure.

Formation of the free ligand and **Mg-5** upon treatment of **Mg-1** with H_2_ might derive from an unstable aromatized magnesium hydride intermediate through the addition of H_2_ to the dearomatized side arm and the magnesium center (that is, activation of H_2_ via the MLC process). The active intermediate partially decomposed to generate the free ligand and the rest converted to **Mg-5** via the elimination of the ethyl group and a proton on the side arm. To prove the assumption that H_2_ addition to **Mg-1** occurs via an MLC process, 5 bar of D_2_ was added to a toluene solution of **Mg-1** in a J. Young NMR tube (Figure 4b). After heating at 65 °C for 16 h, partial decomposition of the complex to the free ligand was detected by ^31^P{^1^H} NMR (Figure S45). Interestingly, the phosphorus signals of the free ligand and **Mg-1** were split into multiplets. A more remarkable split could be observed upon prolonging the reaction time to 36 h. Such a phenomenon implies the incorporation of deuterium into both side arms of the ligand of **Mg-1**, leading to the formation of different types of phosphorus nuclei. **Mg-1-D** was consequently formed as **Mg-1** with the deuterium-labeled pincer ligand under the current conditions. **Mg-5-D**, with deuteride coordinated to the magnesium center and incorporated into both side arms of **Mg-5**, was also generated, as determined by ^31^P{^1^H} NMR and ^2^H NMR spectroscopy. Notably, after the solution was heated at the same temperature for 3 days, **Mg-1-D** was completely converted to **Mg-5-D** together with the elimination of CH_3_CH_2_D. A Mg-D signal appears at 1.32 ppm in the ^2^H NMR spectrum, further confirming the structure of **Mg-5** and **Mg-5-D**. These results suggest that D_2_ (H_2_) is reversibly activated by **Mg-1** via the MLC process. To determine the deuterated ratio of the side arm at the end of the reaction, higher temperature and longer reaction time were employed to ensure the full decomposition of **Mg-5-D** to the free ligand (PNP)^*t*^Bu-D. Integration of the methylene protons in the ^1^H NMR spectrum confirmed that 95% deuterium was incorporated into the side arms of the free ligand. This result further supports the reversible activation of D_2_ (H_2_) via the MLC process.

The generation of **Mg-1-D** and the free ligand (PNP)^*t*^Bu-D from **Mg-1** likely proceeds via the highly active aromatized intermediate **Int-I**, which is derived from the heterolytic cleavage of D_2_ (Figure 4b). The instability of **Int-I** results in partial decomposition to generate the free ligand, and the rest of **Int-I** regenerates the dearomatized structure to enter the next cycle of D_2_ activation. Notably, the multiplet of the free ligand in the ^31^P{^1^H} NMR spectrum was finally converted to a major singlet upon increasing the temperature to 120 °C, suggesting that some free ligands may regenerate **Mg-1** (**Mg-1-D**), which further incorporates deuterium into its side arms (Figure S45). It should be mentioned that the reaction of **Mg-2** with D_2_ also afforded the free ligand with 94% of deuterium incorporated into the side arms under similar conditions, indicating its capability in the activation of D_2_ (H_2_) via the MLC process (Figure S51). Likewise, **Mg-5** can reversibly activate D_2_ (H_2_) via the MLC, as supported by the observation of **Mg-5-D** formation when **Mg-5** was pressurized with D_2_ (Figure 4c and also see Figure S53). Finally, to further confirm that deuterium incorporation proceeds via the MLC process, the free ligand (PNP)^*t*^Bu was treated with D_2_ under similar conditions in the absence of the magnesium precursor, resulting in no incorporation of deuterium into the side arms (Figure S55). This result is also in line with our previous work involving zinc pincer complexes.^51^

### Catalytic Semihydrogenation of Alkynes

As the reversible activation of H_2_ (D_2_) by **Mg-1**, **Mg-2**, and **Mg-5** via the MLC process was shown to be feasible, the hydrogenation of C–C multiple bonds catalyzed by the new magnesium complexes might be possible. We first tested the semihydrogenation of internal alkynes hept-1-yn-1-ylbenzene (**1a**) with 3 mol % of **Mg-1** as a catalyst. Alkene was generated in 98% yield and 29:1 Z/E selectivity after heating at 120 °C for 24 h under 5 bar of H_2_. The over-reduction byproduct amounted to less than 2%, according to ^1^H NMR. The catalytic activities of the complexes we obtained were evaluated under the above conditions (*vide infra* and also see Table S10). **Mg-1** showed the highest efficiency. A series of other reaction conditions, including temperature, H_2_ pressure, and solvent, were screened, but no further improvements in the reaction yield and selectivity were observed (Table S11). With the optimal conditions in hand, we explored the scope of semihydrogenation of alkynes (Table 1). Arylalkyl alkynes with different chain lengths successfully afforded the desired *Z*-alkenes with excellent yields and selectivities (**2a**–**2d**). The steric hindrance of the alkyl substituent was explored under the optimal conditions. Cyclopropyl, cyclopentyl, and cyclohexyl substituted arylalkyl alkynes had no impact on the reaction yield and stereoselectivity. The desired products (**2e**–**2g**) were smoothly produced with excellent results (99% yield, 38:1–>99:1 Z/E). However, the bulky tertiary butyl substituted alkyne **1h** generated product **2h** with only 83% yield and 25:1 Z/E selectivity. Next, diaryl alkynes were further evaluated. Hydrogenation of diphenylacetylene **1i** gave *cis*-stilbene in 55% yield. Increasing the pressure of H_2_ to 8 bar and prolonging the reaction time to 36 h, **2i** was obtained in 88% yield and 8:1 Z/E stereoselectivity. The conditions were also suitable for the semihydrogenation of F- and ^*t*^Bu-substituted diaryl alkynes **1j** and **1k**, generating products in 82 and 85% yields, respectively. Surprisingly, bis(trimethylsilyl)acetylene, a challenging substrate under most other homogeneous catalyses, smoothly converts to *Z*-bis(trimethylsilyl)ethene **2l** in 99% yield and >99:1 stereoselectivity. In addition, the semihydrogenation reaction can proceed with dialkyl alkynes, generating the desired alkenes **2m**–**2o** in up to >99% yield and >99:1 stereoselectivity. Next, the substituents on the phenyl ring of arylalkyl alkynes were evaluated. The position of the substituent did not influence the reaction yield but had an impact on the selectivity. For example, all of the *ortho*-, *meta-*, and *para*-methyl substituted alkynes successfully generated the desired alkenes **2p**–**2r** with excellent yields under the standard conditions, but the stereoselectivity was affected when the methyl group was placed at the *ortho* position of the triple bond. The electron density of the alkynes has no impact on the yield and selectivity of the reaction. Both electron-rich alkynes (**1w**) and electron-deficient alkynes (**1s**–**1u**) can generate desired products with excellent yields. It is worth mentioning that electron-rich naphthyl substituted alkyne efficiently transformed into product **2x** in 99% yield and 29:1 stereoselectivity. These results indicate the compatibility of the developed catalysis for different types of alkynes and different functional groups. However, the current catalysis is not suitable for the hydrogenation of terminal alkynes due to the incompatibility of the magnesium catalyst with the acidic proton of terminal alkynes.

**Table 1:**
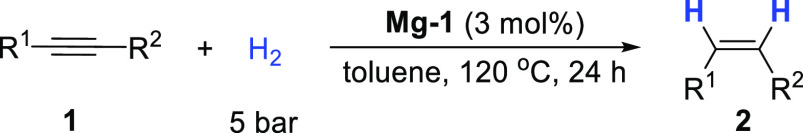

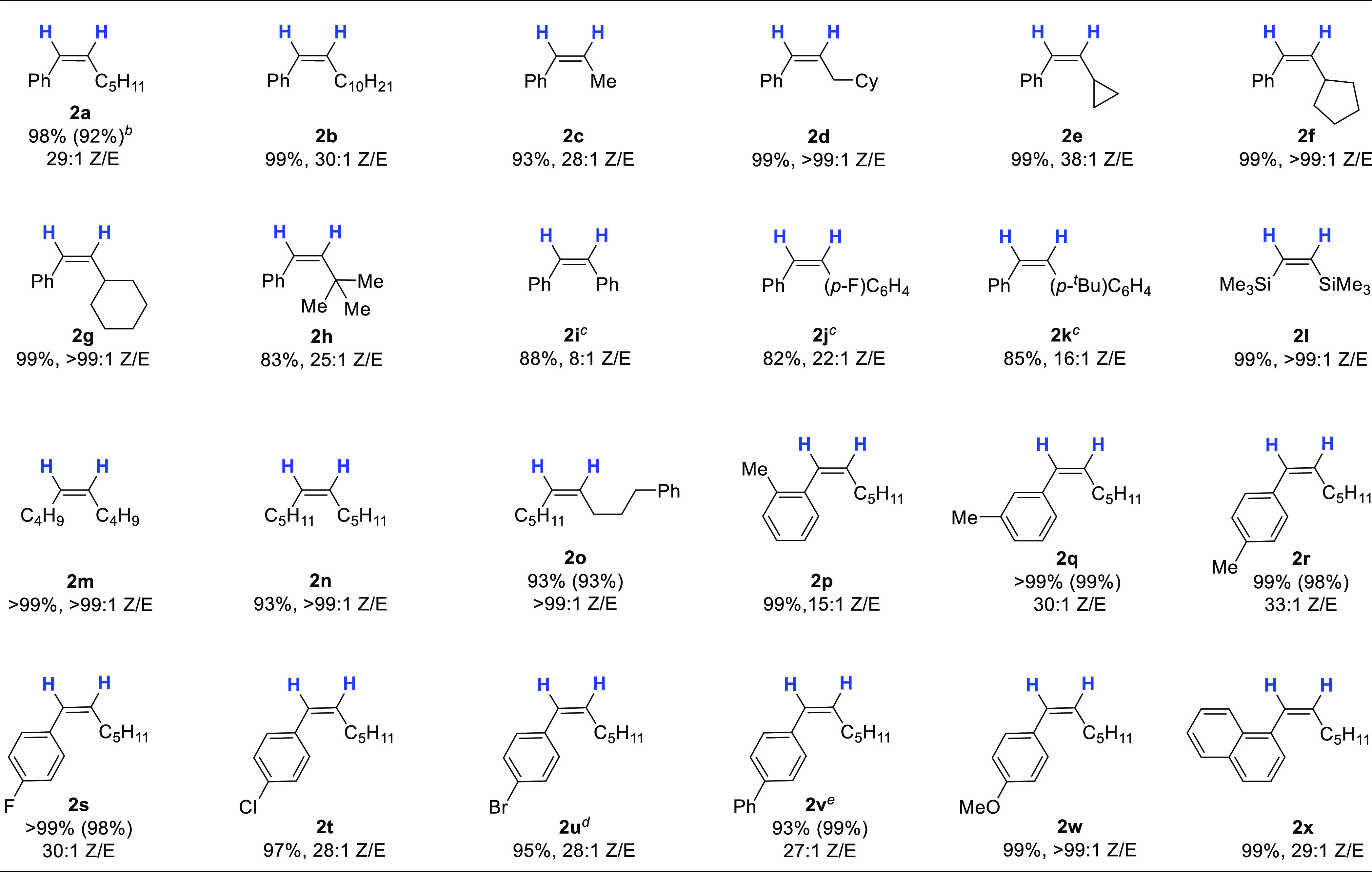
Catalytic Semihydrogenation of Alkynes^a^

a Reaction conditions: **1** (0.3 mmol), **Mg-1** (3 mol %), H_2_ (5 bar), toluene (0.5 mL), 120 °C, 24 h. Reaction yields were determined by ^1^H NMR using 1,3,5-trimethoxybenzene as the internal standard. Isolated yields are in parentheses. The Z/E ratio was determined by ^1^H NMR of the crude solution. The over-reduction products were less than 3% in all cases unless noted otherwise.

b Yield in parenthesis was obtained from a 6 mmol scale reaction.

c 5 mol % catalyst, 8 bar of H_2_, and 36 h reaction time.

d With 5% over-reduction product.

e With 7% over-reduction product, the isolated yield in parenthesis contains 7% over-reduction product.

### Catalytic Hydrogenation of Alkenes

To the best of our knowledge, magnesium-catalyzed hydrogenation of alkenes remains challenging,^27,30,31^ which may be due to the lack of efficient catalysts for H_2_ splitting. Considering the efficiency of the developed complexes in the semihydrogenation of alkynes, we explored if they are also suitable for the hydrogenation of alkenes. Indeed, styrene **3a** was smoothly converted to the desired product **4a** in >99% yield using 5 bar of H_2_ and 4 mol % **Mg-1** at 120 °C for 24 h. Other substituted styrenes were also tested under the optimal conditions (Table 2). The sterically hindered *ortho*-methyl styrene **3b** required longer heating, 48 h, to generate the product in high yield (98%). When a variety of styrenes bearing functional groups, i.e., OMe–, Me–, ^*t*^Bu–, and F–, at the *meta-* and *para*-position of the phenyl ring were utilized, the reaction gave the corresponding products **4c**–**4g** in high yields (83–>99%). Interestingly, the reaction yield was slightly affected by the electron density of the double bonds. For example, fluoro-substituted alkenes **3g** gave a lower yield than the neutral (**3a**) and electron-rich (**3c** and **3e**) alkenes. As expected, electron-rich 2-vinyl naphthalene **3h** smoothly generated the desired product **4h** in 95% yield. Aliphatic terminal alkenes were further evaluated under the optimal conditions. Monosubstituted alkenes with different chain lengths were efficiently transformed into saturated products (**4i**–**4m**) in excellent yields (87–>99%) catalyzed by **Mg-1**. Finally, we turned our attention to the more challenging internal alkene **3n**. It was not surprising that only 10% yield was obtained under the optimal conditions. After increasing the pressure of H_2_ to 7 bar and **Mg-1** loading to 5 mol % and extending the reaction time to 48 h, the reaction yield was improved to 70%. The above results indicate that the developed magnesium complexes are not only suitable for the semihydrogenation of alkynes but also efficient in the hydrogenation of alkenes.

**Table 2:**
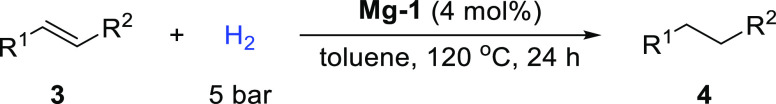

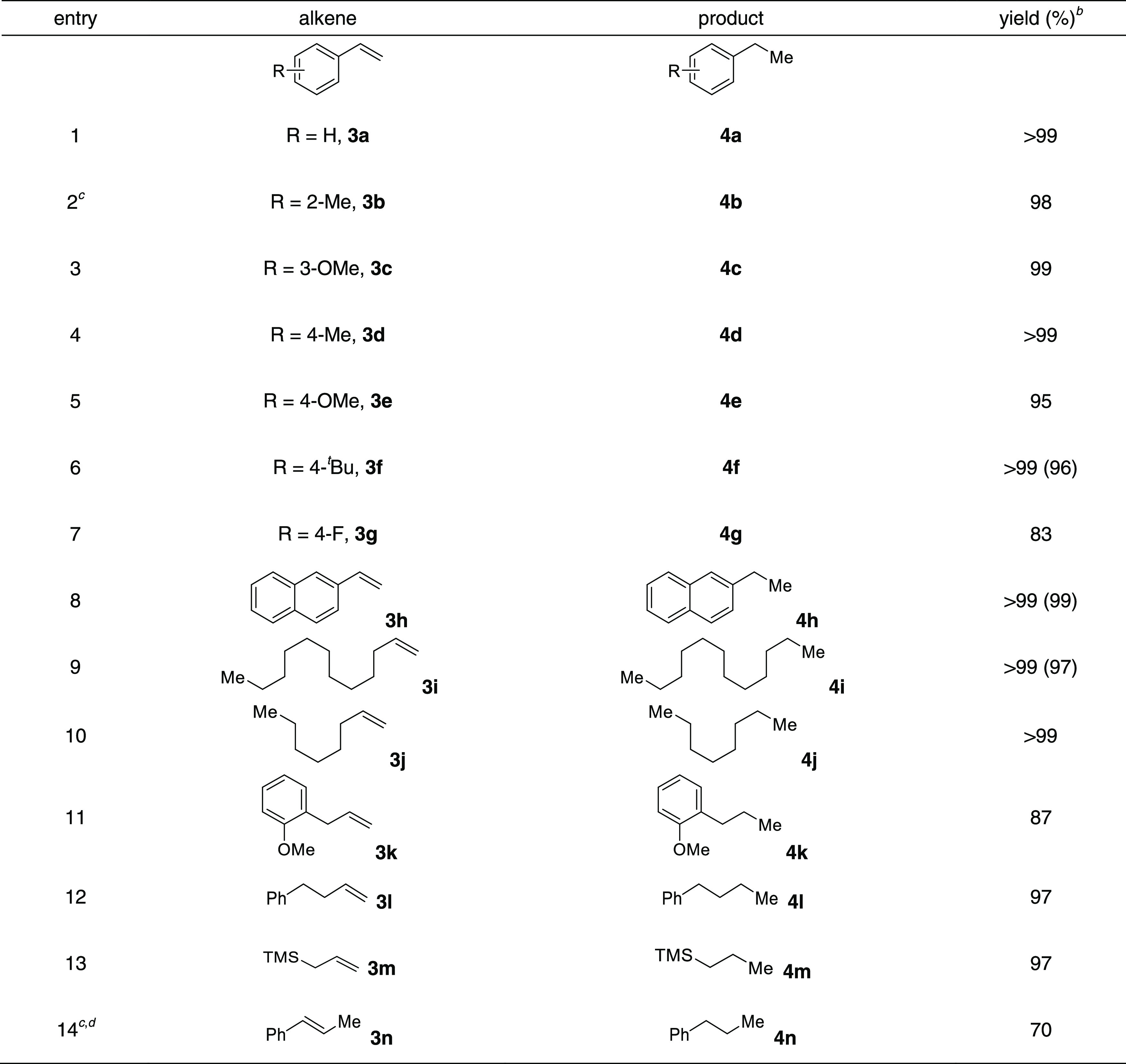
Catalytic Hydrogenation of Alkenes^a^

a Reaction conditions: **3** (0.3 mmol), **Mg-1** (4 mol %), H_2_ (5 bar), toluene (0.5 mL), 120 °C, 24 h.

b Reaction yields were determined by ^1^H NMR using benzyl benzoate as the internal standard. Isolated yields are in parentheses.

c Reaction time was 48 h.

d 7 bar of H_2_ and 5 mol % **Mg-1** were used.

### Mechanistic Studies

**Figure 5:**
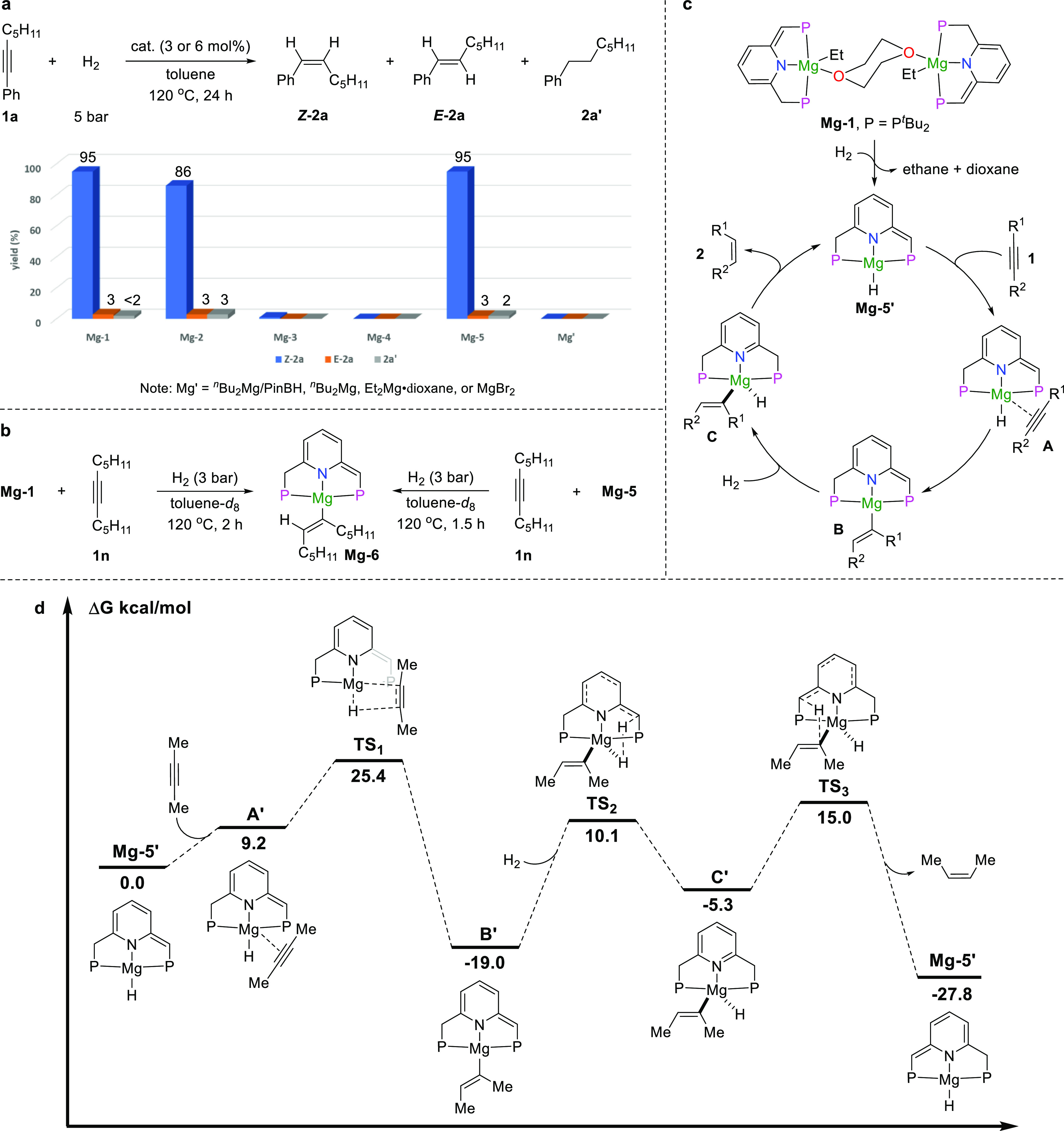
Mechanistic insight. (a) Effect of different catalysts in the semihydrogenation of alkynes (Note: the loading of **Mg-1**, **Mg-2**, and **Mg-3** is 3 mol %, and the loading of other catalysts is 6 mol %; **Mg-5** contains about 8% free ligand). (b) Control experiments to determine the reaction intermediate of semihydrogenation of alkynes. (c) Proposed mechanism. (d) DFT studies.

The new magnesium complexes were first tested to compare their effect on the catalytic semihydrogenation of **1a** (Figure 5a and also see Table S10). **Mg-2** gave a slightly lower yield than **Mg-1**, probably because the Mg-phenyl group led to a less stable complex. **Mg-3** and **Mg-4** supported by PNN- and NNN-type ligands generated a trace amount of the product since they are unstable at high temperatures. Interestingly, using the dearomatized hydride complex **Mg-5** as a catalyst, the alkene product was obtained in 98% yield and 32:1 stereoselectivity, a similar result to that of using **Mg-1**. Likewise, the hydrogenation of styrene **3a** catalyzed by **Mg-5** also generated the desired product in >99% yield (Table S12). Considering the fact that **Mg-5** is derived from **Mg-1** upon treatment with H_2_, **Mg-5** may act as the active catalyst in the hydrogenation reaction. Next, to demonstrate the importance of the dearomatized PNP ligand to the hydrogenation reaction, various simple magnesium compounds were further evaluated. According to previous reports, a magnesium hydride intermediate can be formed as active catalytic species in the combination of ^*n*^Bu_2_Mg with PinBH.^22,23^ However, the generated magnesium hydride species was unable to catalyze the hydrogenation of alkyne **1a**. Other magnesium compounds, including ^*n*^Bu_2_Mg, Et_2_Mg·dioxane, and MgBr_2_, were also examined as catalysts in the semihydrogenation of alkyne **1a**, but no conversion of **1a** was observed. These results suggest that the dearomatized ligands are essential for the hydrogenation reaction.

To investigate the active catalytic species and reaction intermediates, **Mg-1** was dissolved in toluene-*d*_8_ and combined with 1-phenylpropyne **1c** in a 1:6 ratio in a J. Young NMR tube and pressured with 3 bar of H_2_ to monitor the reaction (Figure S56). Interestingly, after only 10 min at 120 °C, two new singlets appear at 24.89 and −3.51 ppm in the ^31^P{^1^H} NMR spectrum in a 1:1 ratio, suggesting the formation of a new complex as the reaction intermediate. The complete conversion of **Mg-1** to the new species was observable when the reaction time was prolonged to 2 h. We then replaced **1c** with the aliphatic alkyne 6-dodecyne (**1n**) to avoid the overlap of arene signals in the ^1^H NMR spectrum and increased the concentration of alkyne to determine the structure of the generated intermediate (Figure 5b, left). The ^1^H NMR spectrum supported the structure of the generated intermediate as **Mg-6**, with a *cis*-alkene bound to the magnesium center considering the *Z*-configuration of the generated alkene product. In addition, two singlets at 26.54 and −2.42 ppm in the ^31^P{^1^H} NMR spectrum suggest two different phosphorus nuclei of the formed complex. The protons of methine and methylene of the side arms were observed by ^1^H NMR, indicating that the dearomatized structure still remained. A triplet (*J* = 6.2 Hz) assigned to the alkene proton, derived from the addition of magnesium hydride to the alkyne, was detected at 5.87 ppm. H–H COSY spectrum exhibits the correlation with its vicinal C*H*_2_ protons. An sp^2^ carbon bound to the Mg center appears at 172.7 ppm (t, *J* = 23.0 Hz) in the DEPT-Q spectrum. The HMBC spectrum further confirms the observation (see the SI for more characterization data). Meanwhile, the semihydrogenation of alkyne **1n** catalyzed by **Mg-5** was also investigated to further confirm the reaction intermediate (Figure 5b, right, and also see Figure S71). The same species **Mg-6** was formed upon combining **Mg-5** with 6-dodecyne **1n** in a 1:6 ratio under 3 bar of H_2_. Notably, **Mg-6** was also formed in the absence of H_2_. Upon consumption of the alkyne, the regeneration of **Mg-5** was observed. Thus, we can conclude that **Mg-5** is generated *in situ* from **Mg-1** as the active catalyst, and **Mg-6** is formed as a reaction intermediate, which originates from the addition of the Mg–H bond to the triple bond.^75^

Similarly, the hydrogenation of styrene catalyzed by **Mg-1** was also monitored under similar conditions (Figure S72). The formation of an active intermediate via the addition of the Mg–H bond to the styrene double bond was detected as well, indicating an analogous mechanism in the hydrogenation of alkenes. However, internal alkenes exhibit lower reactivity, and their hydrogenation is more challenging. As noticed, the internal alkene **3n** can convert to the alkane in 70% yield when increasing the loading of **Mg-1** and the pressure of H_2_ and prolonging the reaction time. This result prompted us to investigate why *E*-alkenes and alkanes were generated with low yields in the catalytic semihydrogenation of alkynes. We thus prolonged the reaction time for the hydrogenation of **1c** to 48 h (page S65). It was found that the yield of *E*-alkene and alkane increased to 9 and 17%, respectively. Prolonging the reaction time to 60 h did not significantly improve their yields. These results suggest that **Mg-1** can slowly catalyze the isomerization of *Z*-alkenes to the more stable *E*-alkenes and the hydrogenation of internal alkenes to alkanes. However, the gradual decomposition of **Mg-1** to the free ligand led to catalyst deactivation. Therefore, in most cases of the semihydrogenation of alkynes, the *E*-alkenes and over-reduction products can be avoided if the reactions are terminated after 24 h.

On the basis of the control experiments and previous studies on bond activation via MLC involving the aromatization/dearomatization,^51,66−70^ a mechanistic cycle is proposed (Figure 5c). Initially, **Mg-1** is converted to **Mg-5** as the active catalytic species by reaction with H_2_. **Mg-5** can further dissociate into the monomer **Mg-5′**,^17,76^ which provides a vacant site for alkyne coordination to generate intermediate **A**. The coordinated alkyne then inserts into the Mg–H bond to generate a magnesium vinyl intermediate **B**. Next, addition of H_2_ to intermediate **B** via the MLC process results in the aromatization of the pincer ligand to form the vinyl hydride intermediate **C**. Deprotonation of the side arm by the vinyl ligand generates the *Z*-alkene products and the magnesium hydride intermediate (**Mg-5′**) to propagate the cycle. Notably, no change in the metal oxidation state is involved in the overall process. It should be mentioned that the reaction of intermediate **B** with H_2_ via σ-bond metathesis to give the *Z*-alkene product and the intermediate **Mg-5′** was also considered. However, a higher energy barrier of the H_2_ activation step is required (*vide infra*).

The proposed mechanism for the catalytic semihydrogenation of alkynes by the magnesium complexes was calculated by DFT at the ωB97M-V/def2-TZVPP/RIJCOSX/SMD//M06-L/def2-TZVP/GD3/W06/SMD level of theory to get a deeper understanding of the reaction mechanism (Figure 5d). The calculation starts from the active intermediate **Mg-5′** using 2-butyne as a substrate. Coordination of the alkyne to the magnesium center to produce the intermediate **A′** is an endergonic process with Δ*G* = 9.2 kcal/mol. Insertion of the alkyne into the Mg–H bond to generate intermediate **B′** proceeds with an energy barrier of 25.4 kcal/mol in an exergonic step (Δ*G* = −19.0 kcal/mol). This result is in line with our observation that **B′** is a relatively stable intermediate. Activation of H_2_ by intermediate **B′** via MLC affords intermediate **C′** with hydride and vinyl ligands located *trans* to each other. **C′** formation overcomes an energy barrier of 29.1 kcal/mol, and that step is endergonic with Δ*G* = 13.7 kcal/mol, suggesting the high reactivity of intermediate **C′**. Proton transfer from the side arm to the vinyl ligand producing the alkene product and **Mg-5′** is an exergonic process with Δ*G* = −22.5 kcal/mol, and the associated energy barrier is 34.0 kcal/mol. In addition, the hydrogenolysis of intermediate **B′** by H_2_ to produce the alkene product and **Mg-5′** via a sigma-bond metathesis pathway was also considered. A 36.0 kcal/mol energy barrier is required to overcome, which is 6.9 kcal/mol higher in the H_2_ activation step and 2.0 kcal/mol higher in the whole transformation than the MLC pathway (Figure S75). The different energy barriers indicate that the activation of H_2_ via the sigma-bond metathesis pathway is less possible. Finally, the barrier for opening one side arm of intermediate **B′** to form a three-coordinate intermediate for the activation of H_2_ was also calculated, resulting in energy barriers of 33.7 and 42.6 kcal/mol to overcome in the activation of H_2_ via MLC and sigma-bond metathesis pathways, respectively (Figures S76 and S77). These results suggest that the activation of H_2_ via MLC without opening the side arm is a more favorable pathway.

## Conclusions

In summary, we have prepared a series of dearomatized magnesium pincer complexes supported by PNP, PNN, and NNN types of ligands. The complexes were well characterized by NMR and X-ray single-crystal diffraction. The reversible activation of H_2_ by the dearomatized complexes via the MLC process was shown to be feasible. The obtained magnesium pincer complexes catalyze the semihydrogenation of internal alkynes, affording *Z*-alkenes in excellent yields and stereoselectivities. To the best of our knowledge, this is the first example of homogeneous main-group metal-catalyzed semihydrogenation of alkynes, representing significant advances in semihydrogenation of alkynes and main-group metal catalysis. Besides, the developed complexes are suitable for the hydrogenation of alkenes, which have long been dominated by transition metals. Mechanistic studies indicate that a magnesium hydride complex is first formed as the active catalyst for the transformation. The aromatization/dearomatization MLC process is likely to play a significant role in achieving the reaction, as supported by control experiments and DFT studies. We believe that this study will provide new routes for the hydrogenation of other compounds catalyzed by main-group metal complexes.

## Supporting Information Available

Experimental procedures, NMR spectra, and computational details and X-ray crystallographic coordinates for the structures reported in this study have been deposited at the Cambridge Crystallographic Data Centre (CCDC) under deposition numbers 2174062, 2174063, and 2174064 (PDF)
