# Supplementary material for: Magnesium Pincer Complexes and Their Applications in Catalytic Semihydrogenation of Alkynes and Hydrogenation of Alkenes: Evidence for Metal–Ligand Cooperation
Source: J Am Chem Soc. 2022 Oct 4;144(41):19115–26. doi: 10.1021/jacs.2c08491 (PMC9585592; doi:10.1021/jacs.2c08491)
Supplement: Supplementary file 1 — ja2c08491_si_001.pdf [file ja2c08491_si_001.pdf]

Supporting Information

Magnesium Pincer Complexes and Their Applications in Catalytic Semihydrogenation of Alkynes and Hydrogenation of Alkenes: Evidence for Metal-Ligand Cooperation

Yaoyu Liang,[1,§] Uttam Kumar Das,[1,§] Jie Luo,[1] Yael Diskin-Posner,[2] Liat Avram,[2] David Milstein[1]*

[1]Department of Molecular Chemistry and Materials Science, Weizmann Institute of Science, Rehovot 7610001, Israel
[2]Department of Chemical Research Support, Weizmann Institute of Science, Rehovot 7610001, Israel
[§]These authors contributed equally to this work
*e-mail: david.milstein@weizmann.ac.il

Table of Contents

1 General considerations 2

2 Synthesis and characterization of magnesium complexes 3

3 Hydrogen activation by magnesium pincer complexes 32

4 Catalytic semihydrogenation of alkynes 48

5 Catalytic hydrogenation of alkenes 53

6 Mechanistic studies 53

7 Computational details 65

8 NMR spectra 95

9 References 122

1 General considerations

All the experiments were carried out under an atmosphere of purified nitrogen in a Vacuum Atmosphere glovebox equipped with a MO 40-2 inert gas purifier or using standard Schlenk techniques. Toluene, benzene, pentane, THF, and dioxane were refluxed over sodium/benzophenone, distilled under argon or nitrogen atmosphere, and stored over activated 4Å molecular sieves (MS). Other solvents were used after degassing with nitrogen. Deuterated benzene and toluene were degassed with nitrogen and stored in the glovebox over 4Å MS. NMR spectra were recorded on Bruker AVANCE III (300 MHz or 400 MHz) or AVANCE III HD (500 MHz) spectrometers and are reported in ppm (δ). ^{1}H NMR spectra are referred to the residual solvent peaks (toluene-d_8: 2.08 ppm, benzene-d_6: 7.15 ppm, and $CHCl_3$: 7.26 ppm), and ^{13}C NMR spectra are referred to the residual solvent peaks (toluene-d_8: 20.43 ppm, benzene-d_6: 128.06 ppm, and $CHCl_3$: 77.16 ppm). ^{31}P NMR chemical shifts are referenced with respect to an external solution of 85% phosphoric acid in D_2O. NMR spectroscopy abbreviations: s, singlet; d, doublet; t, triplet; q, quartet; m, multiplet; br, broad. The ^{1}H diffusion NMR measurements were performed on an 11.7 T (500 MHz) AVANCE III HD NMR spectrometer (Bruker, Germany) equipped with a 50 gauss/cm Z gradient system. For both compounds (**Mg-4** and **Mg-5**) a LED (longitudinal eddy current delay) sequence was used at 298 K. The diffusion experiments were performed with smoothed square (SMSQ.10.100) gradients, incremented from 2% to 98% in 32 linear steps and 16 scans were acquired for each gradient. The gradient duration was 2 ms and the diffusion time was 40 ms. GC-MS analyses were carried out on HP 6890/5973 (MS detector) instruments equipped with a 30 m column (Restek 5MS, 0.32 mm internal diameter) with a 5% phenylmethylsilicone coating (0.25 mm) with He as carrier gas.

All ligands were prepared according to the literature procedures.[1-3] All commercial reagents were purchased from Strem, Sigma Aldrich, Acros, or Alfa Aesar, and used as received without any further purification.

2 Synthesis and characterization of magnesium complexes

2.1 Synthesis and characterization of Mg-1

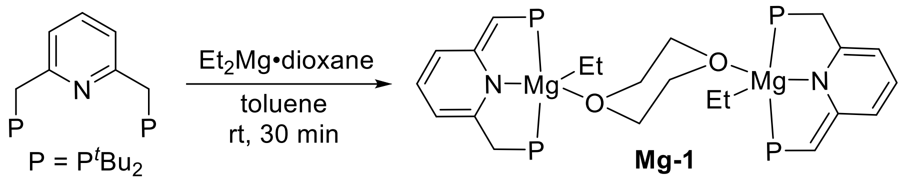

Et_2Mg·dioxane was prepared according to the literature procedure.[4] In a N_2 glovebox, the PNP ligand (158.1 mg, 0.4 mmol) and Et_2Mg·dioxane (102.1 mg, 0.6 mmol) were added to a 20 mL vial and then dissolved with toluene (5 mL). The color changed immediately from colorless to light yellow. After stirring at room temperature for 30 min, an orange solution was obtained. The solvent was removed under vacuum to give an orange solid. To further purify the complex, 0.5 mL of benzene was added to dissolve the solid, and pentane (about 1 mL) was slowly added. The resulting solution was stored in the freezer (-32ºC) until orange crystals were formed. The crystals were suitable for X-ray diffraction. Pentane was decanted, and the solid was dried under vacuum to give **Mg-1** as an orange solid (172.9 mg, 88% yield).

^{1}H NMR (400 MHz, C_6D_6) δ 6.48 – 6.41 (m, 1H, Py***H***), 6.23 (d, *J* = 8.8 Hz, 1H, Py***H***), 5.56 (d, *J* = 6.4 Hz, 1H, Py***H***), 3.59 (d, *J* = 4.7 Hz, 1H, PyC***H***P), 3.44 (s, 4H, OC***H***$_2$), 2.55 (s, 2H, PyC***H***$_2$P), 1.84 (t, *J* = 8.2 Hz, 3H, MgCH$_2$C***H***$_3$), 1.24 (d, *J* = 13.0 Hz, 18H, PC(C***H***$_3$)$_3$), 1.09 (d, *J* = 11.5 Hz, 18H, PC(C***H***$_3$)$_3$), 0.13 (q, *J* = 8.2 Hz, 2H, MgC***H***$_2$CH$_3$).

^{13}C NMR (101 MHz, C_6D_6) δ 168.74 (d, *J* = 17.7 Hz, Py***C***), 153.59 (dd, *J* = 5.9, 2.6 Hz, Py***C***), 133.13 (d, *J* = 2.8 Hz, Py***C***), 119.08 (dd, *J* = 10.3, 1.9 Hz, Py***C***), 102.40 (d, *J* = 8.4 Hz, Py***C***), 67.43 (s, O***C***H$_2$), 59.95 (d, *J* = 34.9 Hz, Py***C***HP), 32.99 (d, *J* = 11.6 Hz, P***C***(CH$_3$)$_3$), 31.88 (d, *J* = 12.4 Hz, P***C***(CH$_3$)$_3$), 30.19 (d, *J* = 11.6 Hz, Py***C***H$_2$P), 29.98 (d, *J* = 9.9 Hz, PC(***C***H$_3$)$_3$), 29.87 (d, *J* = 8.2 Hz, PC(***C***H$_3$)$_3$), 13.48 (s, MgCH$_2$***C***H$_3$), 3.87 (dd, *J* = 27.3, 22.2 Hz, Mg***C***H$_2$CH$_3$).

^{31}P NMR (162 MHz, C_6D_6) δ 23.54 (d, *J* = 3.1 Hz, PyCH$_2$***P***), -2.67 (d, *J* = 3.1 Hz, PyCH***P***).

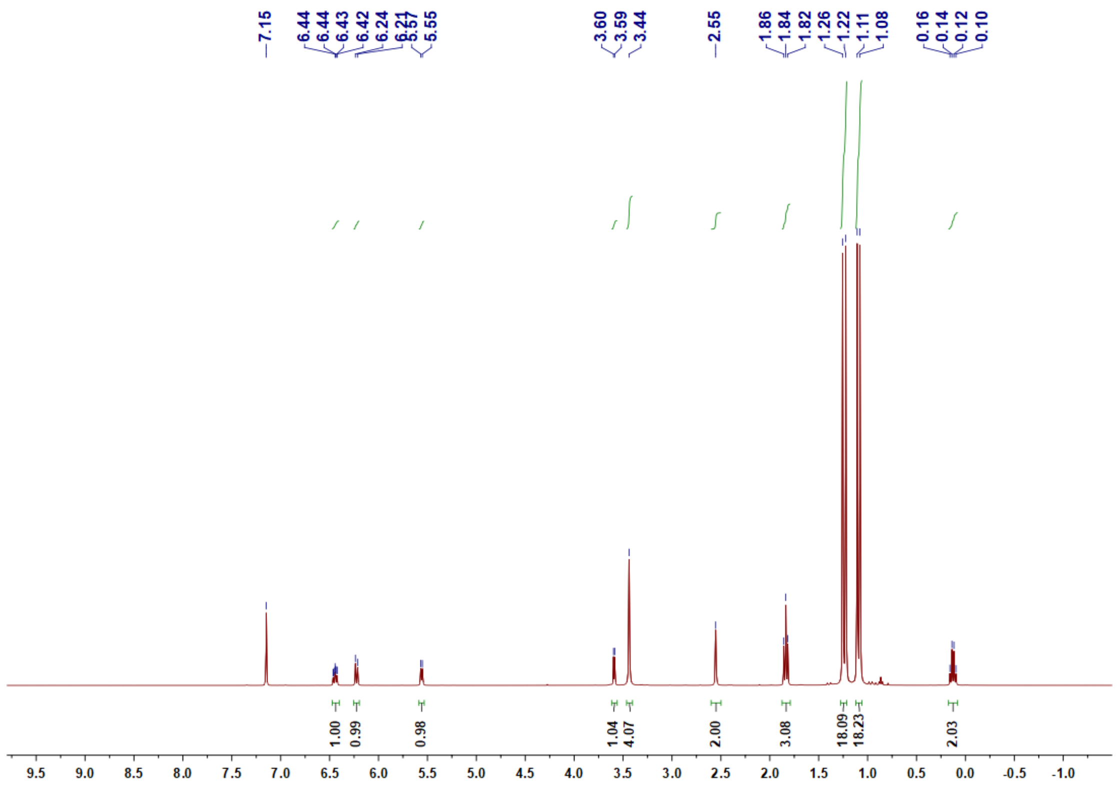

Figure S1. ^{1}H NMR spectrum of **Mg-1** in C_6D_6

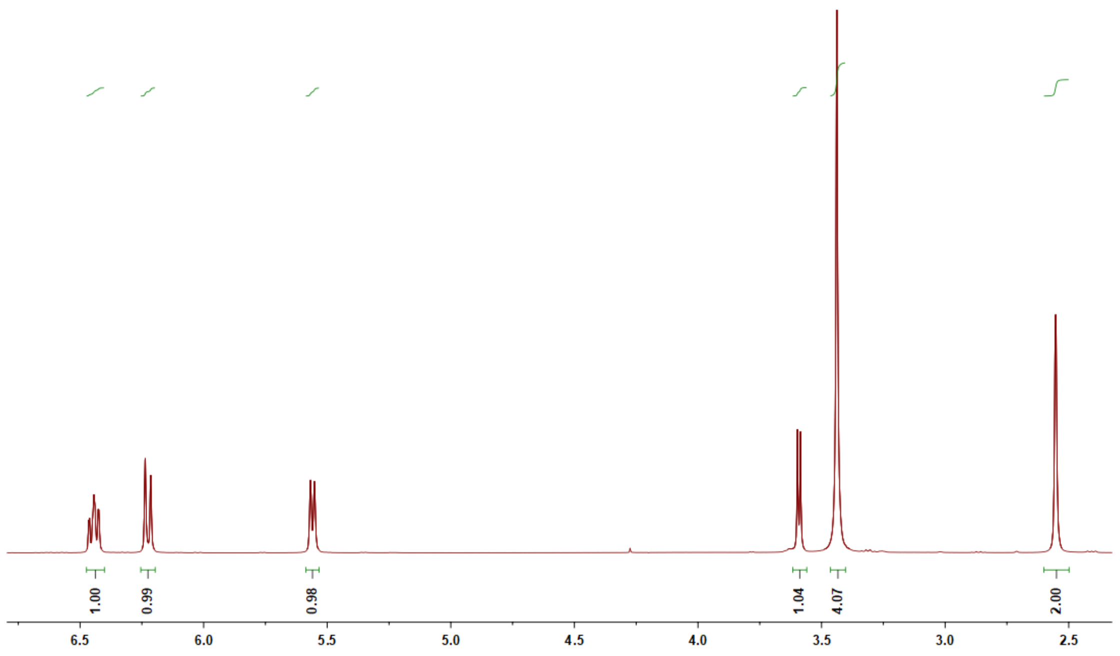

Figure S2. ^{1}H NMR spectrum (zoom in) of **Mg-1** in C_6D_6

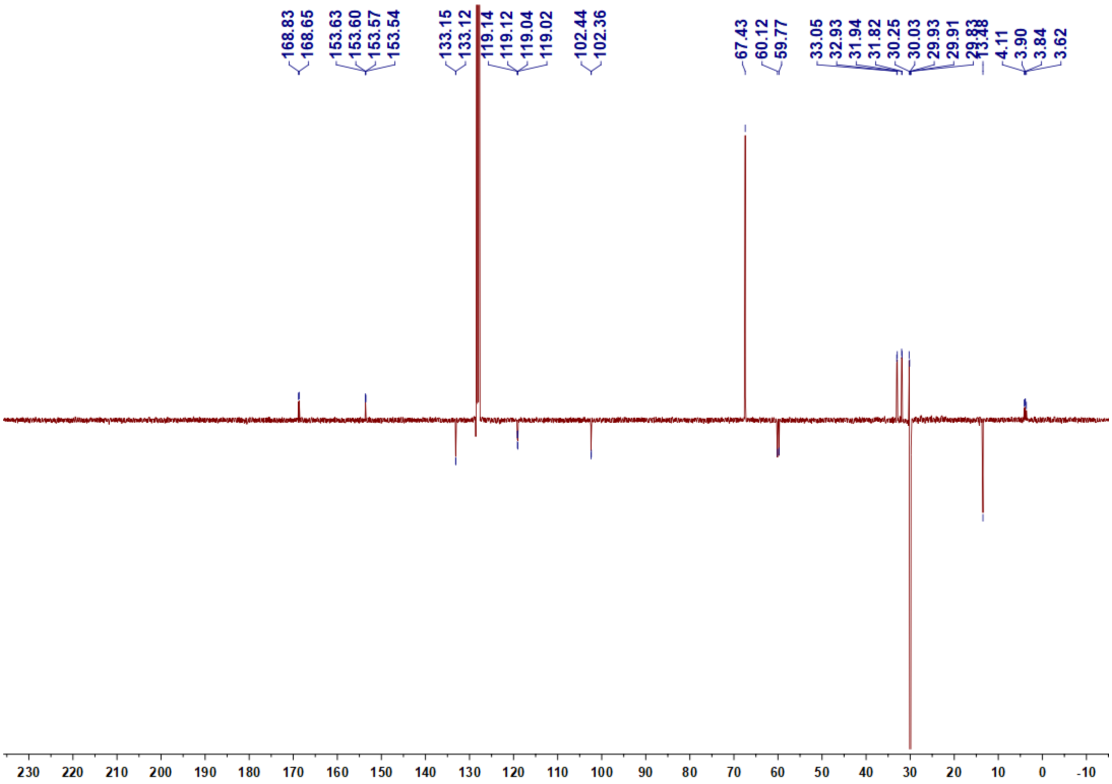

Figure S3. ^{13}C-DEPTQ NMR spectrum of **Mg-1** in C_6D_6

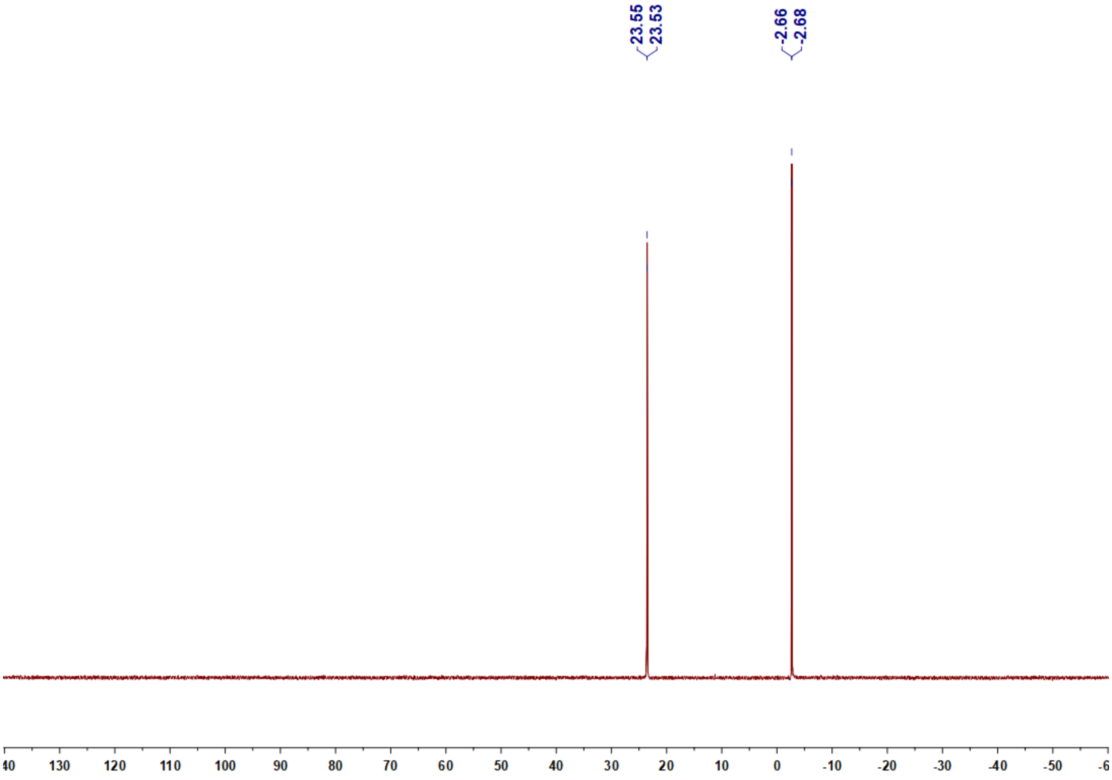

Figure S4. ^{31}P NMR spectrum of **Mg-1** in C_6D_6

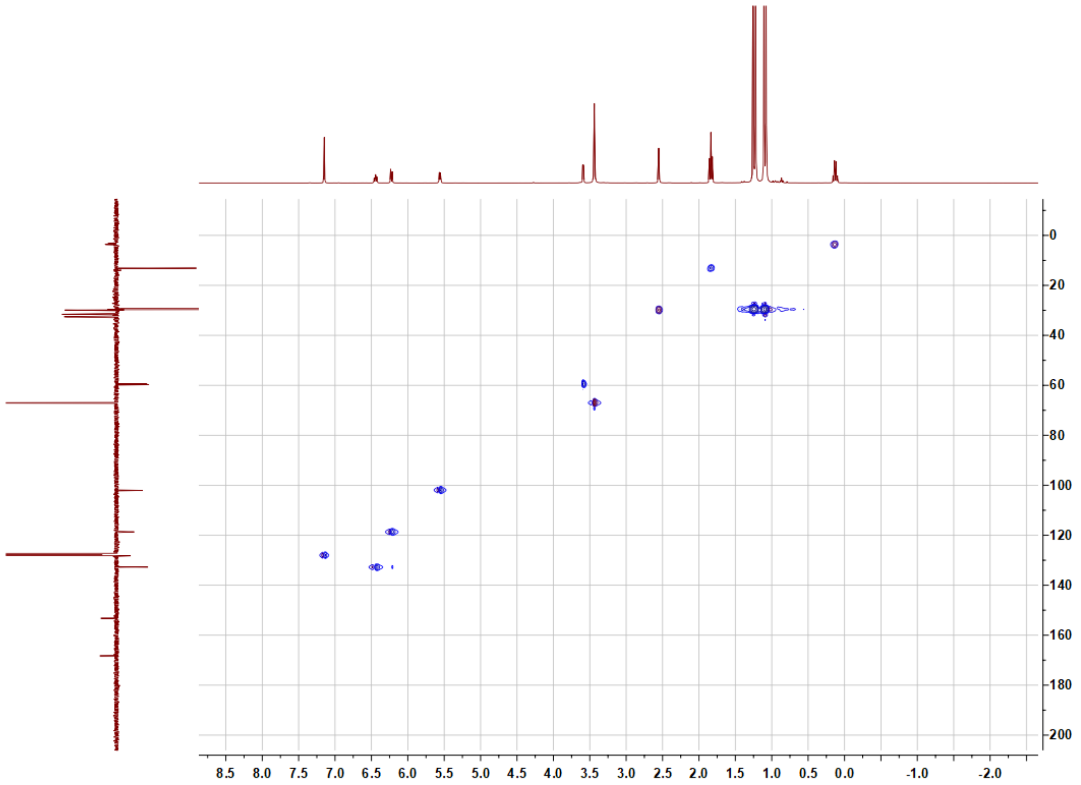

Figure S5. HSQC spectrum of **Mg-1** in C_6D_6

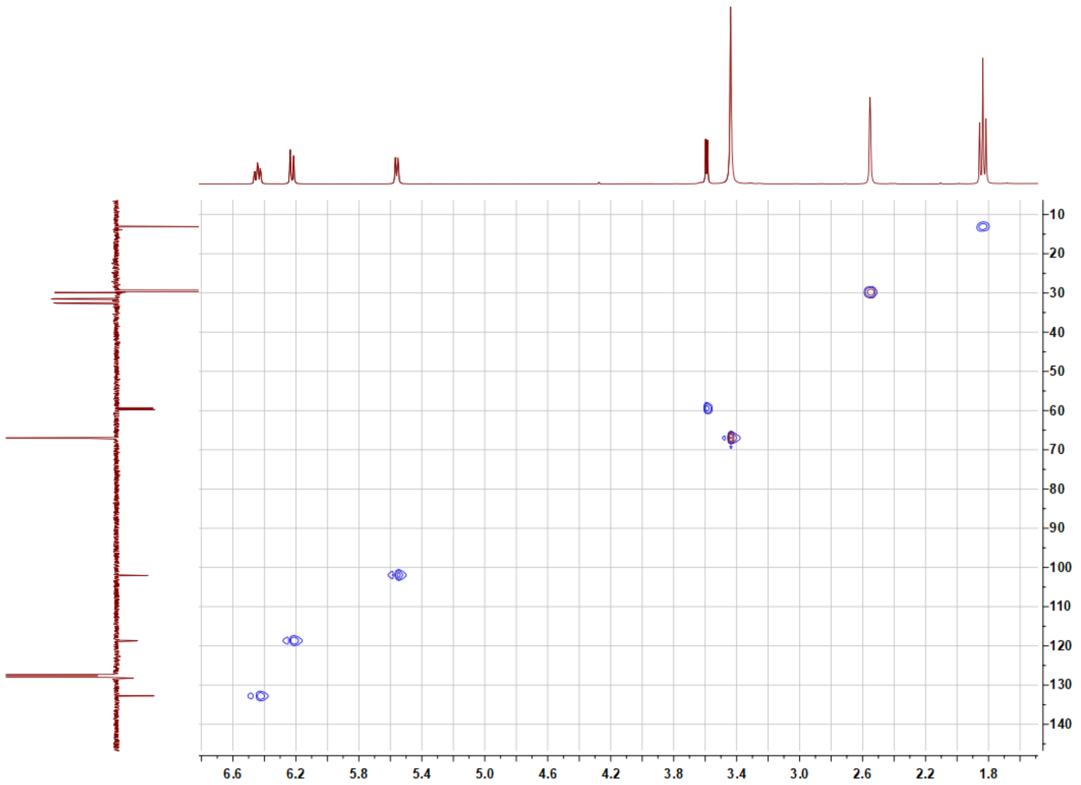

Figure S6. HSQC spectrum (zoom in) of **Mg-1** in C_6D_6

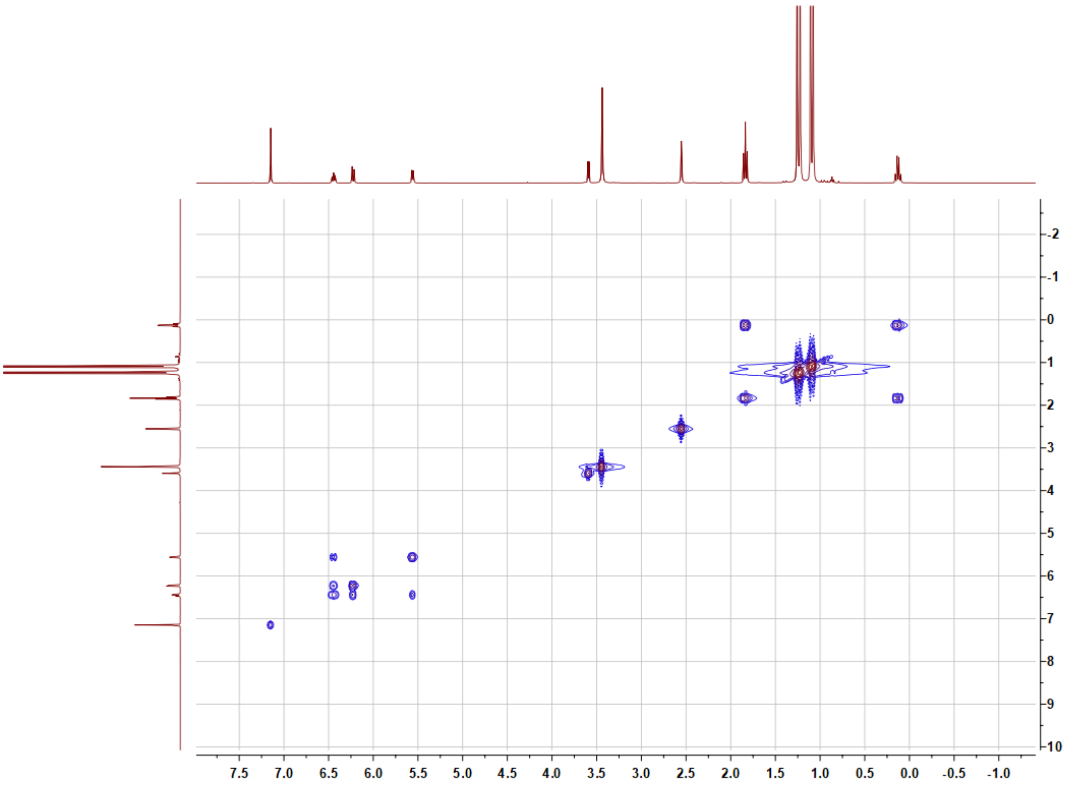

Figure S7. H-H COSY spectrum of **Mg-1** in C_6D_6

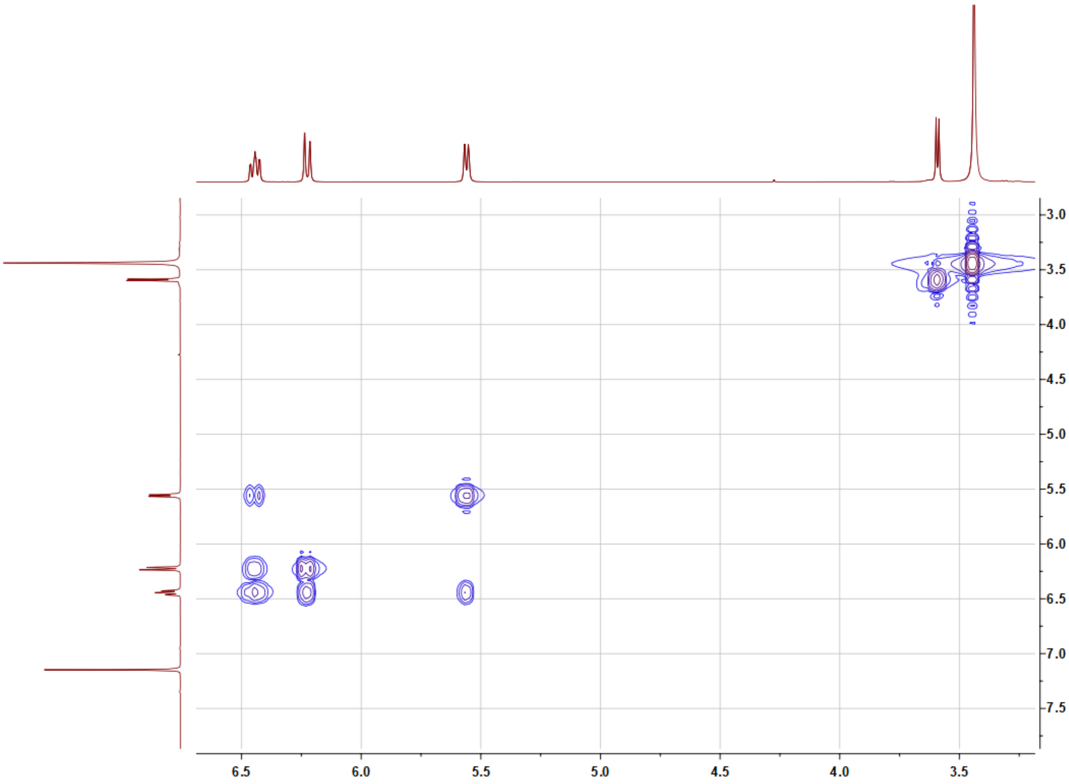

Figure S8. H-H COSY spectrum (zoom in) of **Mg-1** in C_6D_6

The diffraction data from single crystals of **Mg-1** were collected on Rigaku Xtalab PRO diffractometer dual source equipped with Dectris Pilatus 200K detector and microfocus, with CuKα (λ=1.54184 Å). The data were processed with CrysAlis^{PRO} [5]. The structures were solved with SHELXT[6]. Full matrix least-squares and refined based on F^2 with SHELXL[7]. All structure solution and refinement programs are implemented in Olex-2 GUI[8]. All non-hydrogen atoms were refined with anisotropic displacement coefficients. Hydrogens were placed in calculated positions and refined in a riding mode. Supplementary crystallographic data have been deposited at the Cambridge Crystallographic Data Center (CCDC 2174062).

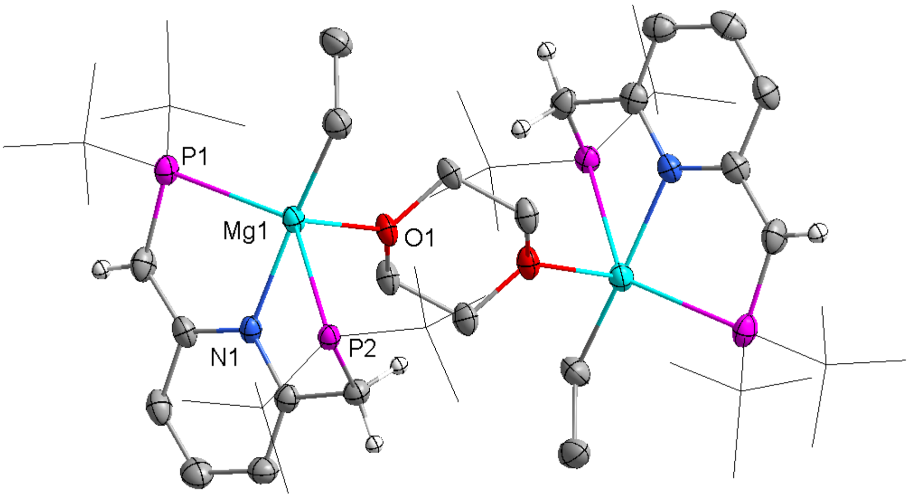

Figure S9. X-ray crystal structure of **Mg-1**

Table S1. Crystal data and structure refinement for Mg-1

Empirical formula	$C_{54}H_{102}Mg_2N_2O_2P_4$
Crystal description	Yellow block
Crystal size (mm^3)	0.147 × 0.115 × 0.035
Formula weight (g/mol)	983.87
T (K)	100.0(2)
Wavelength (Å)	1.54184
Crystal system	Orthorhombic
Space group	*Pna21*

a (Å)	14.9550(1)
b (Å)	13.3228(1)
c (Å)	29.9756(2)
α (°)	90
β (°)	90
γ (°)	90
Volume ($Å^3$)	5972.41(7)
Z	4
ρ_{cal} (mg/m^3)	1.094
μ (mm^{-1})	1.649
No. of reflection collected (Unique)	96084(12137)
R_{int}	0.1150
Completeness to θ (%)	100.0
Limiting indices	-18<=h<=15, -16<=k<=16, -37<=l<=36
Data\restraints\ parameters	12137/1/597
Goodness-of-fit on F^2	1.028
Final R_1 and wR_2 indices [I>2σ(I)]	R_1 = 0.0681, wR_2 = 0.1854
R_1 and wR_2 indices (all data)	R_1 = 0.0715, wR_2 = 0.1899
Largest diff. peak and hole ($e/Å^3$)	1.493 and -0.576

Table S2. Bond lengths for Mg-1

Atom	Atom	Length/Å	Atom	Atom	Length/Å
P1	Mg1	2.656(2)	C6	C7	1.509(8)
P1	C1	1.770(7)	C8	C9	1.538(11)
P1	C8	1.879(6)	C8	C10	1.518(11)
P1	C12	1.883(6)	C8	C11	1.521(11)
P2	Mg1	2.928(2)	C12	C13	1.524(8)
P2	C7	1.852(6)	C12	C14	1.513(9)
P2	C16	1.900(6)	C12	C15	1.549(9)
P2	C20	1.893(6)	C16	C17	1.516(9)
P3	Mg2	2.639(2)	C16	C18	1.526(9)
P3	C26	1.773(7)	C16	C19	1.546(9)
P3	C33	1.896(9)	C20	C21	1.529(8)

P3	C37	1.905(7)	C20	C22	1.528(9)
P4	Mg2	2.909(2)	C20	C23	1.530(9)
P4	C32	1.832(6)	C24	C25	1.531(9)
P4	C41	1.880(6)	C26	C27	1.406(9)
P4	C45	1.900(6)	C27	C28	1.446(9)
Mg1	O1	2.171(4)	C28	C29	1.349(10)
Mg1	N1	2.140(5)	C29	C30	1.416(10)
Mg1	C24	2.155(6)	C30	C31	1.357(9)
Mg2	O2	2.178(4)	C31	C32	1.510(9)
Mg2	N2	2.151(5)	C33	C34	1.519(14)
Mg2	C49	2.152(7)	C33	C35	1.532(14)
O1	C51	1.442(7)	C33	C36	1.506(16)
O1	C54	1.444(6)	C37	C38	1.515(9)
O2	C52	1.443(7)	C37	C39	1.532(11)
O2	C53	1.447(7)	C37	C40	1.523(10)
N1	C2	1.404(7)	C41	C42	1.526(10)
N1	C6	1.363(7)	C41	C43	1.549(9)
N2	C27	1.376(8)	C41	C44	1.530(8)
N2	C31	1.378(8)	C45	C46	1.536(9)
C1	C2	1.393(9)	C45	C47	1.531(9)
C2	C3	1.446(9)	C45	C48	1.553(10)
C3	C4	1.335(10)	C49	C50	1.522(9)
C4	C5	1.406(10)	C51	C52	1.510(8)
C5	C6	1.380(8)	C53	C54	1.507(9)

Table S3. Bond angles for Mg-1

Atom	Atom	Atom	Angle/°	Atom	Atom	Atom	Angle/°
C1	P1	Mg1	93.5(2)	C6	C7	P2	109.6(4)
C1	P1	C8	105.1(3)	C9	C8	P1	113.7(6)
C1	P1	C12	105.9(3)	C10	C8	P1	112.8(5)
C8	P1	Mg1	123.4(2)	C10	C8	C9	105.0(7)
C8	P1	C12	111.7(3)	C10	C8	C11	110.6(8)
C12	P1	Mg1	113.58(18)	C11	C8	P1	105.9(5)
C7	P2	Mg1	80.12(18)	C11	C8	C9	109.0(7)
C7	P2	C16	102.5(3)	C13	C12	P1	114.7(4)
C7	P2	C20	103.1(3)	C13	C12	C15	108.0(6)
C16	P2	Mg1	117.1(2)	C14	C12	P1	112.4(5)
C20	P2	Mg1	130.5(2)	C14	C12	C13	109.1(5)
C20	P2	C16	110.5(3)	C14	C12	C15	109.0(6)
C26	P3	Mg2	94.1(2)	C15	C12	P1	103.3(4)

C26	P3	C33	103.9(4)	C17	C16	P2	115.8(5)
C26	P3	C37	105.2(3)	C17	C16	C18	110.8(5)
C33	P3	Mg2	124.5(4)	C17	C16	C19	108.1(5)
C33	P3	C37	111.7(4)	C18	C16	P2	106.7(4)
C37	P3	Mg2	113.2(2)	C18	C16	C19	108.6(5)
C32	P4	Mg2	80.4(2)	C19	C16	P2	106.6(4)
C32	P4	C41	103.7(3)	C21	C20	P2	106.8(4)
C32	P4	C45	103.1(3)	C21	C20	C23	107.5(5)
C41	P4	Mg2	130.7(2)	C22	C20	P2	114.4(4)
C41	P4	C45	110.0(3)	C22	C20	C21	108.7(5)
C45	P4	Mg2	116.9(2)	C22	C20	C23	109.8(5)
P1	Mg1	P2	136.09(8)	C23	C20	P2	109.3(4)
O1	Mg1	P1	107.66(13)	C25	C24	Mg1	116.6(4)
O1	Mg1	P2	105.18(13)	C27	C26	P3	122.8(5)
N1	Mg1	P1	80.70(13)	N2	C27	C26	121.9(6)
N1	Mg1	P2	72.41(13)	N2	C27	C28	118.5(6)
N1	Mg1	O1	87.49(17)	C26	C27	C28	119.5(6)
N1	Mg1	C24	165.5(2)	C29	C28	C27	120.9(6)
C24	Mg1	P1	107.88(18)	C28	C29	C30	119.6(6)
C24	Mg1	P2	93.72(18)	C31	C30	C29	118.4(6)
C24	Mg1	O1	100.5(2)	N2	C31	C32	115.7(5)
P3	Mg2	P4	137.46(9)	C30	C31	N2	123.7(6)
O2	Mg2	P3	107.34(13)	C30	C31	C32	120.6(6)
O2	Mg2	P4	104.05(13)	C31	C32	P4	110.2(4)
N2	Mg2	P3	80.34(15)	C34	C33	P3	112.1(8)
N2	Mg2	P4	72.73(14)	C34	C33	C35	110.2(9)
N2	Mg2	O2	88.06(18)	C35	C33	P3	110.0(6)
N2	Mg2	C49	165.2(3)	C36	C33	P3	105.0(6)
C49	Mg2	P3	107.7(2)	C36	C33	C34	108.1(9)
C49	Mg2	P4	93.5(2)	C36	C33	C35	111.2(11)
C49	Mg2	O2	100.9(2)	C38	C37	P3	112.5(5)
C51	O1	Mg1	127.7(3)	C38	C37	C39	110.2(6)
C51	O1	C54	109.8(4)	C38	C37	C40	109.4(6)
C54	O1	Mg1	121.0(3)	C39	C37	P3	113.3(6)
C52	O2	Mg2	119.8(4)	C40	C37	P3	103.4(5)
C52	O2	C53	109.0(4)	C40	C37	C39	107.6(7)
C53	O2	Mg2	129.3(3)	C42	C41	P4	106.9(4)
C2	N1	Mg1	119.9(4)	C42	C41	C43	108.4(5)
C6	N1	Mg1	121.9(4)	C42	C41	C44	107.8(6)
C6	N1	C2	118.2(5)	C43	C41	P4	114.4(5)

C27	N2	Mg2	120.7(4)	C44	C41	P4	111.3(4)
C27	N2	C31	118.7(5)	C44	C41	C43	107.9(5)
C31	N2	Mg2	120.6(4)	C46	C45	P4	115.9(5)
C2	C1	P1	123.5(4)	C46	C45	C48	110.6(5)
N1	C2	C3	117.8(5)	C47	C45	P4	107.3(4)
C1	C2	N1	122.1(5)	C47	C45	C46	107.2(6)
C1	C2	C3	120.1(5)	C47	C45	C48	108.0(6)
C4	C3	C2	121.7(6)	C48	C45	P4	107.5(4)
C3	C4	C5	120.1(6)	C50	C49	Mg2	118.9(5)
C6	C5	C4	118.0(6)	O1	C51	C52	110.8(5)
N1	C6	C5	124.1(5)	O2	C52	C51	110.4(5)
N1	C6	C7	115.9(5)	O2	C53	C54	111.0(5)
C5	C6	C7	120.1(5)	O1	C54	C53	109.8(5)

2.2 Synthesis and characterization of Mg-2

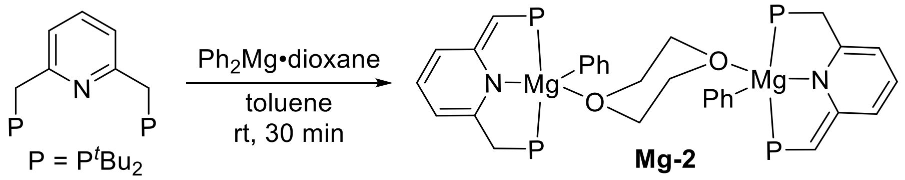

Ph_2Mg·dioxane was prepared according to the literature procedure.[9] In a N_2 glovebox, the PNP ligand (79.1 mg, 0.2 mmol) and Ph_2Mg·dioxane (53.2 mg, 0.2 mmol) were added to a 20 mL vial and then dissolved with toluene (2 mL). The color changed immediately from colorless to light yellow. After stirring at room temperature for 30 min, an orange solution was obtained. The solvent was removed under vacuum to give the pure complex **Mg-2** (80.9 mg, 75% yield).

^{1}H NMR (400 MHz, C_6D_6) δ 8.21 (d, *J* = 6.3 Hz, 2H, Ph***H***), 7.49 (t, *J* = 7.3 Hz, 2H, Ph***H***), 7.33 (t, *J* = 7.3 Hz, 1H, Ph***H***), 6.53 – 6.44 (m, 1H, Py***H***), 6.28 (d, *J* = 8.8 Hz, 1H, Py***H***), 5.56 (d, *J* = 6.3 Hz, 1H, Py***H***), 3.76 (s, 4H, OC$\boldsymbol{H}_2$), 3.68 (d, *J* = 4.0 Hz, 1H, PyC***H***P), 2.58 (s, 2H, PyC$\boldsymbol{H}_2$P), 1.22 (d, *J* = 12.6 Hz, 18H, PC(C$\boldsymbol{H}_3$)$_3$), 1.07 (d, *J* = 11.4 Hz, 18H, PC(C$\boldsymbol{H}_3$)$_3$).

^{13}C NMR (101 MHz, C_6D_6) δ 171.44 (dd, *J* = 30.3, 24.5 Hz, Ph***C***), 168.96 (dd, *J* = 18.4, 1.3 Hz, Py***C***), 154.48 (dd, *J* = 6.2, 2.4 Hz, Py***C***), 141.22 (d, *J* = 1.4 Hz, Ph***C***), 132.75 (d, *J* = 2.7 Hz, Py***C***), 126.54 (s, Ph***C***), 124.87 (s, Ph***C***), 119.43 (dd, *J* = 9.9, 2.2 Hz, Py***C***), 102.21 (d, *J* = 7.3 Hz, Py***C***), 69.86 (s, O***C***H_2), 60.69 (d, *J* = 31.9 Hz,

Py***C***HP), 33.14 (d, J = 8.5 Hz, P***C***(CH_3)$_3$), 32.06 (d, J = 12.8 Hz, P***C***(CH_3)$_3$), 30.12 (d, J = 10.5 Hz, Py***C***H_2P), 29.91 (d, J = 2.3 Hz, PC(***C***H_3)$_3$), 29.82 (d, J = 3.7 Hz, PC(***C***H_3)$_3$).

^{31}P NMR (162 MHz, C_6D_6) δ 16.41 (s, PyCH_2***P***), -6.34 (s, PyCH***P***).

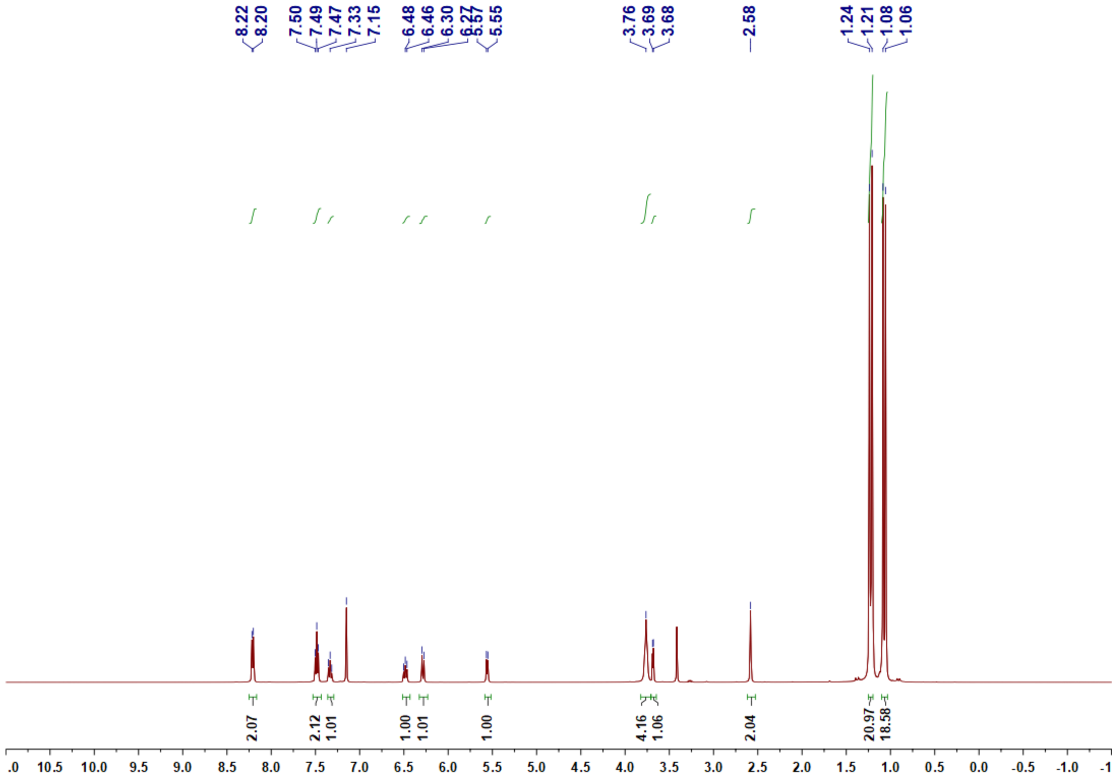

Figure S10. ^{1}H NMR spectrum of **Mg-2** in C_6D_6

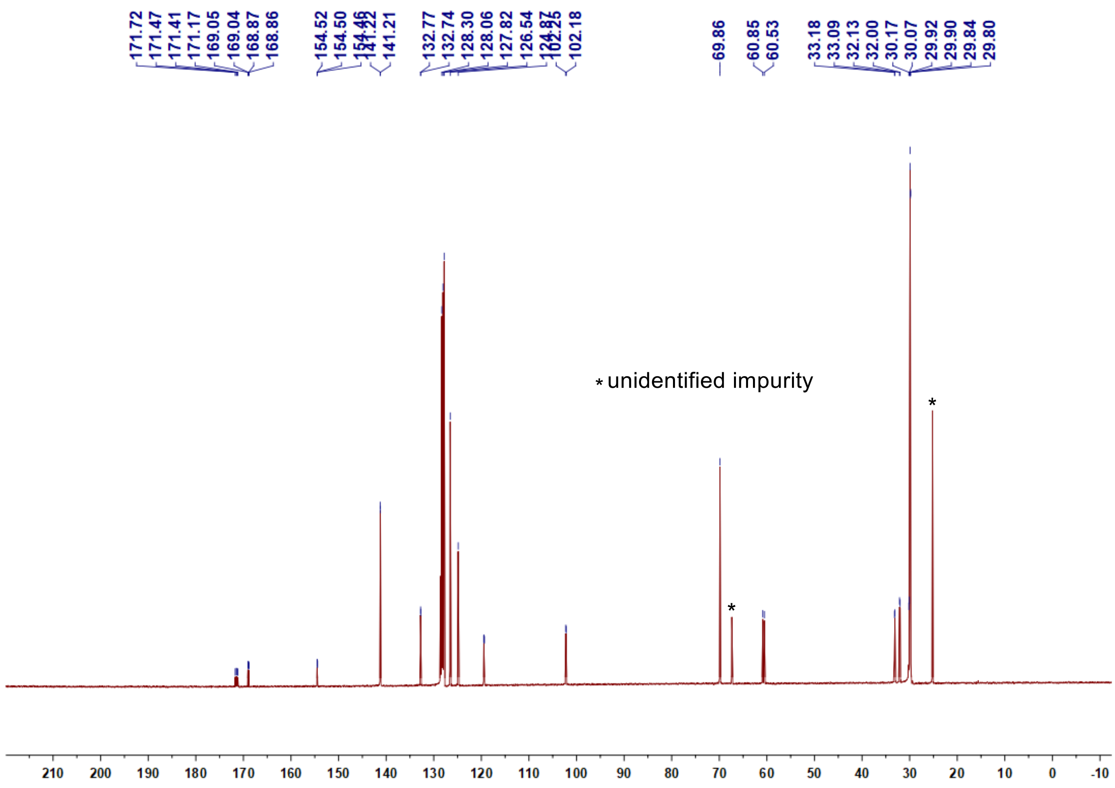

Figure S11. ^{13}C NMR spectrum of **Mg-2** in C_6D_6

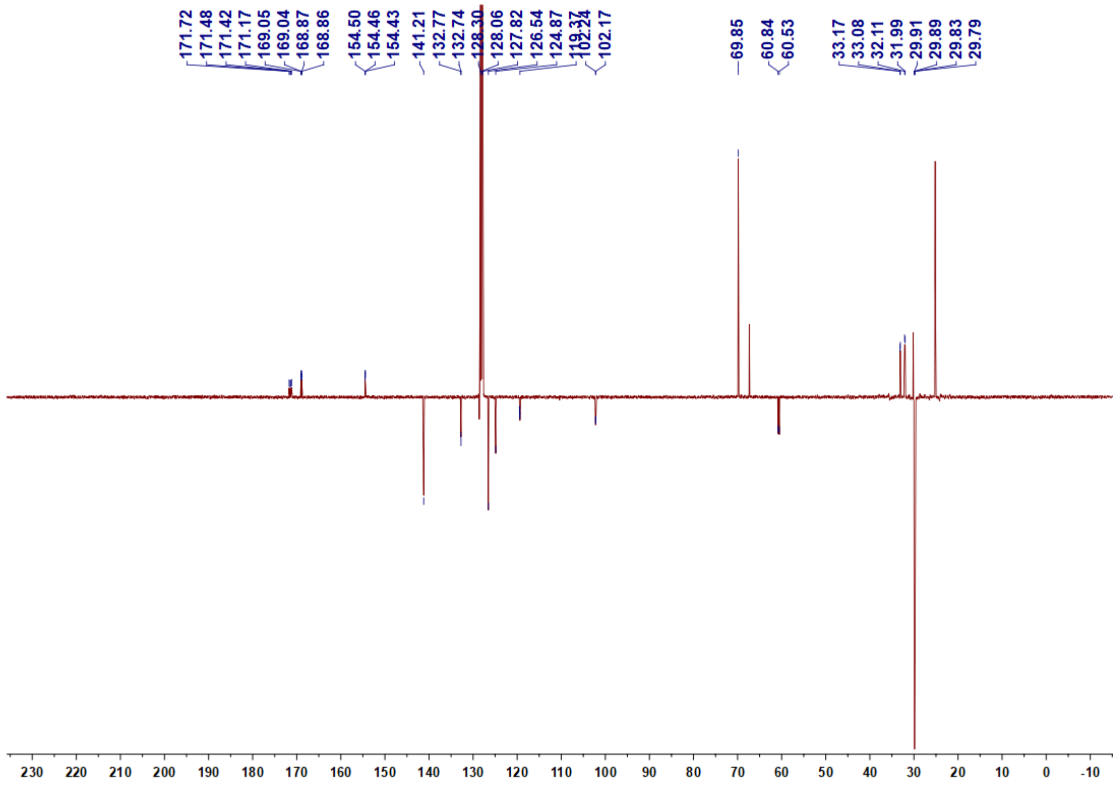

Figure S12. ^{13}C-DEPTQ NMR spectrum of **Mg-2** in C_6D_6

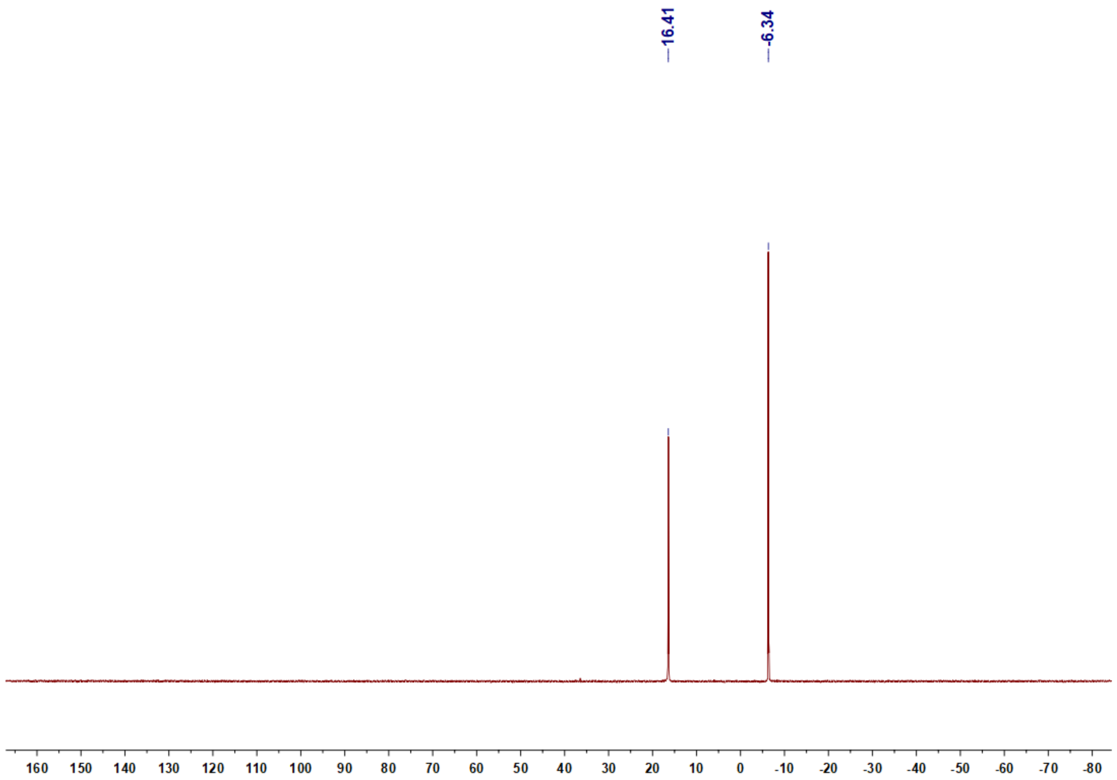

Figure S13. ^{31}P NMR spectrum of **Mg-2** in C_6D_6

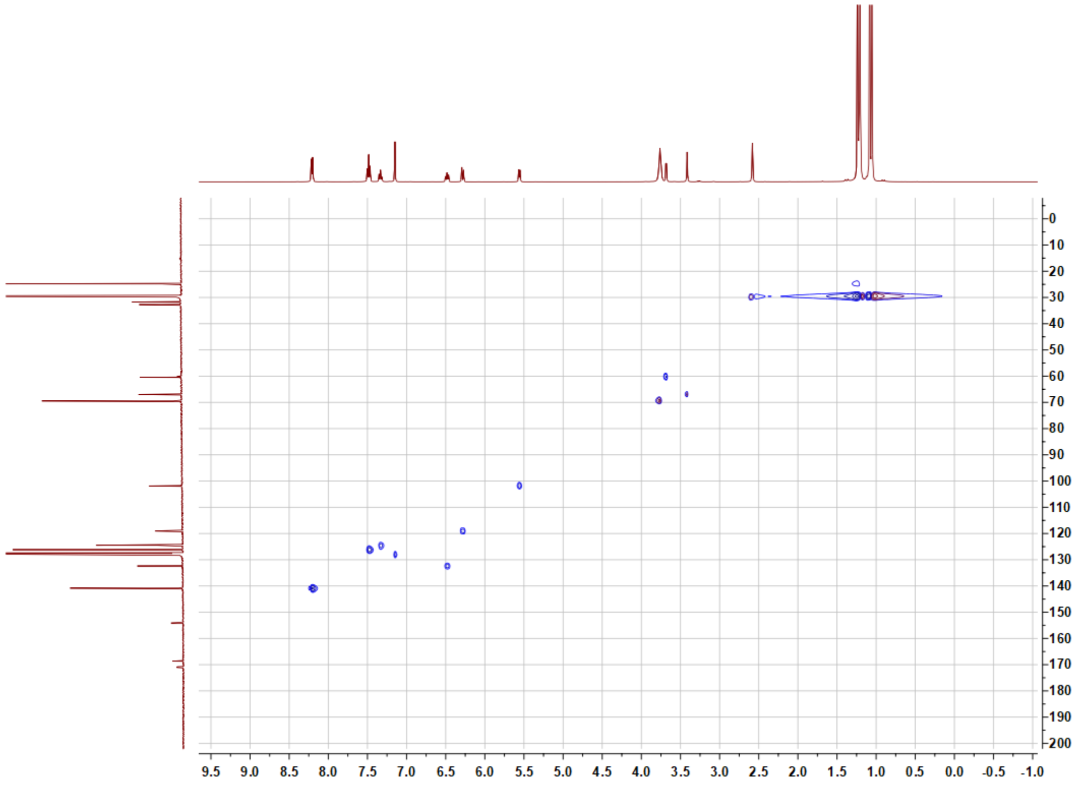

Figure S14. HSQC spectrum of **Mg-2** in C_6D_6

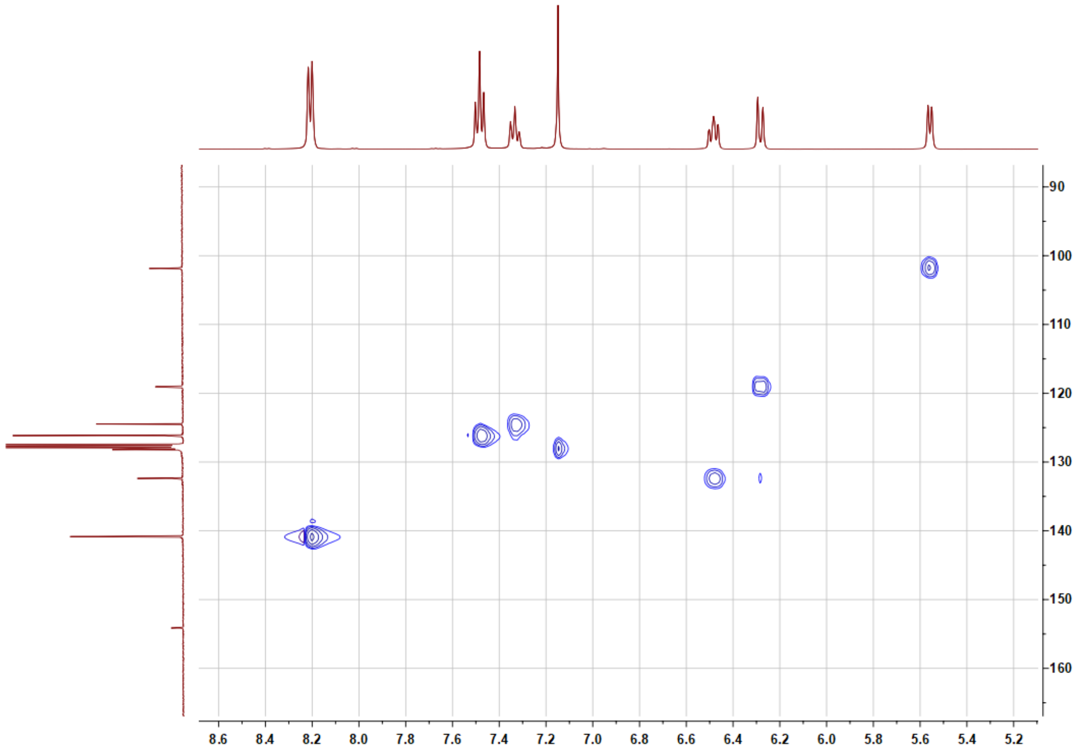

Figure S15. HSQC spectrum (zoom in) of **Mg-2** in C_6D_6

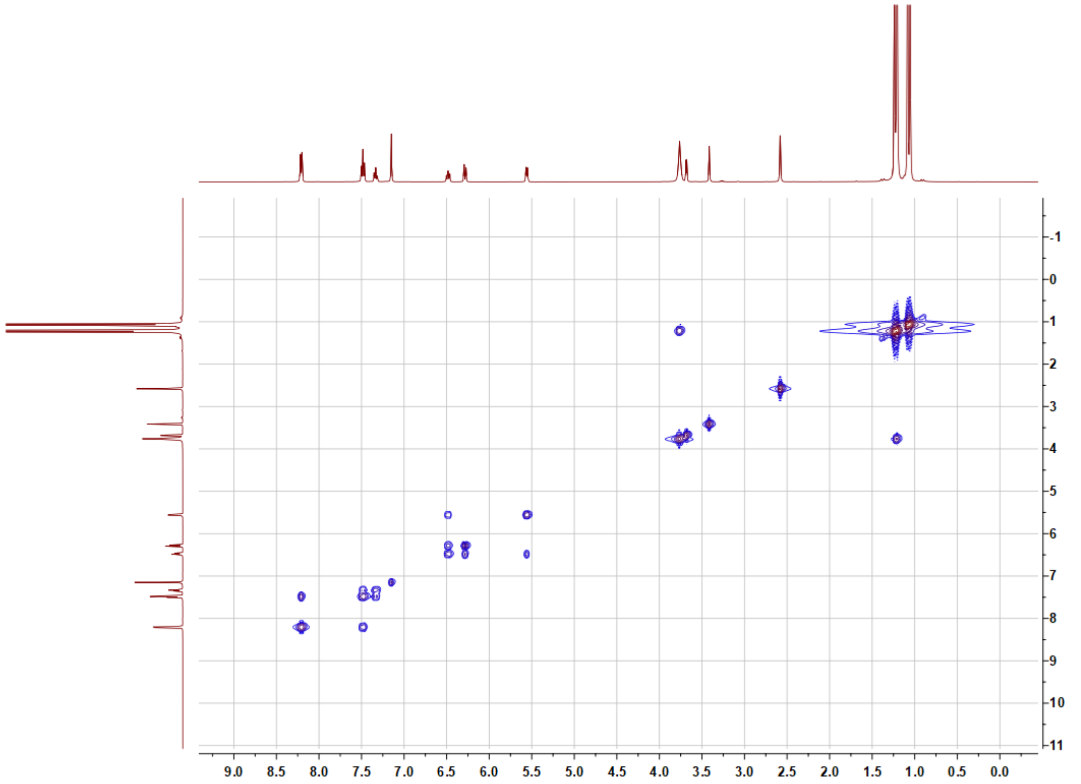

Figure S16. H-H COSY spectrum of **Mg-2** in C_6D_6

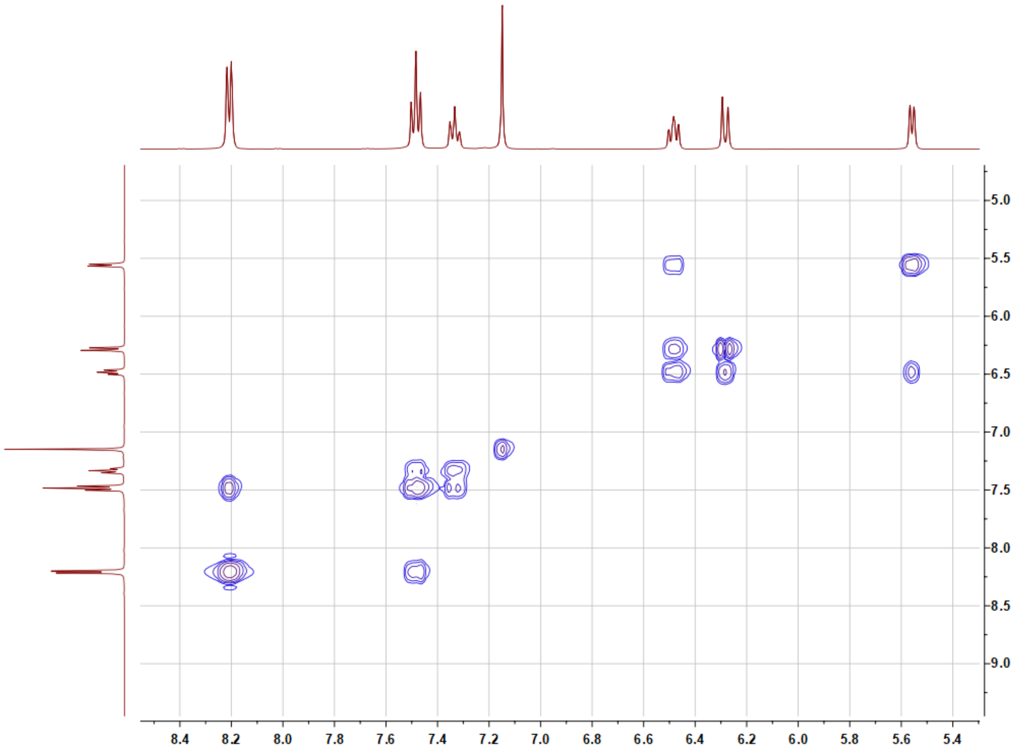

Figure S17. H-H COSY spectrum (zoom in) of **Mg-2** in C_6D_6

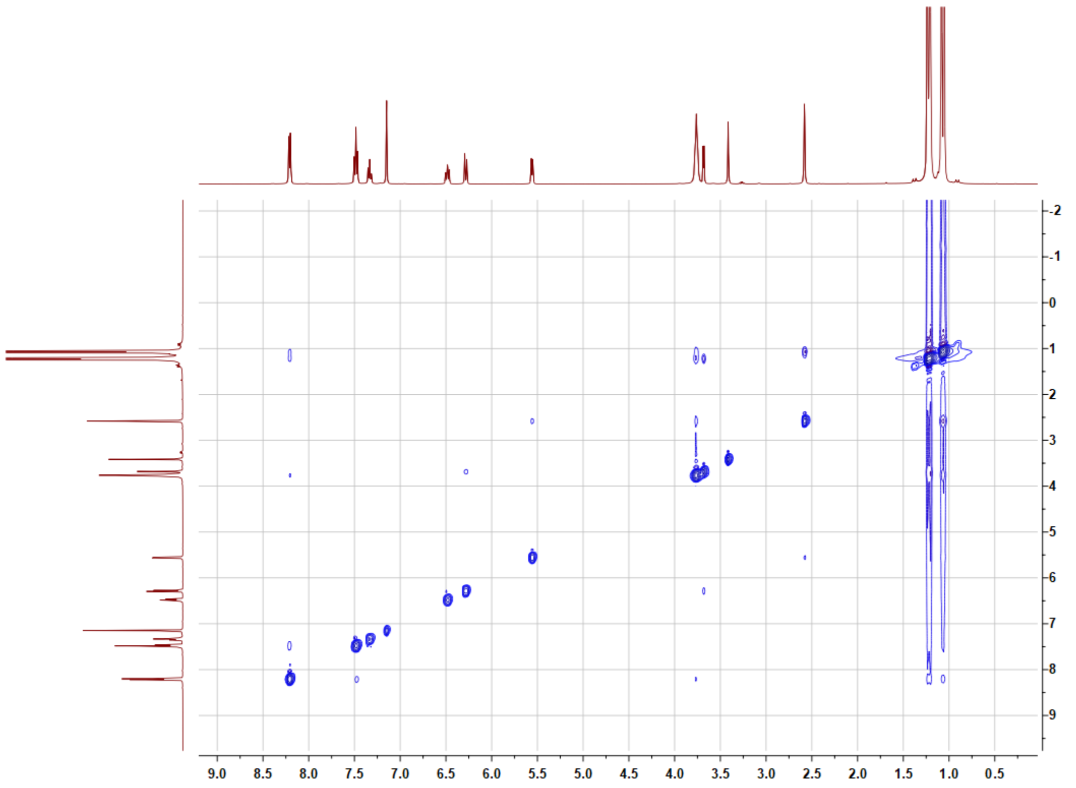

Figure S18. H-H NOSY spectrum of **Mg-2** in C_6D_6

2.3 Synthesis and characterization of Mg-3

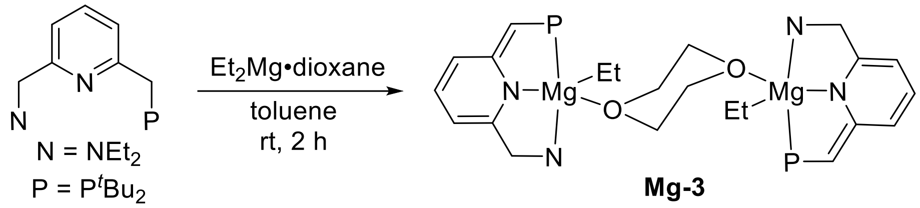

In a N_2 glovebox, the PNN ligand (96.7 mg, 0.3 mmol) and Et_2Mg·dioxane (76.6 mg, 0.45 mmol) were added to a 20 mL vial and then dissolved with toluene (2 mL). After stirring at room temperature for 2 h, an orange solution was obtained. The solvent was removed under vacuum to give an orange solid. To further purify the complex, 0.3 mL of benzene was added to dissolve the solid, and then pentane (about 1 mL) was slowly added. The resulting solution was stored in the freezer (-32ºC) until orange crystals were formed. The crystals were suitable for X-ray diffraction. Solvent was decanted, and the crystals were dried under vacuum to give **Mg-3** as an orange solid (77.8 mg, 62% yield).

^{1}H NMR (400 MHz, C_6D_6) δ 6.43 (ddd, *J* = 8.7, 6.3, 1.4 Hz, 1H, Py***H***), 6.22 (d, *J* = 8.9 Hz, 1H, Py***H***), 5.13 (d, *J* = 6.2 Hz, 1H, Py***H***), 3.66 (d, *J* = 4.7 Hz, 1H, PyC***H***P), 3.46 (s, 4H, OC***H***2), 2.80 (s, 2H, PyC***H***2P), 2.41 (br, 4H, NC***H***2CH3), 1.78 (t, *J* = 8.2 Hz, 3H, MgCH2C***H***3), 1.28 (d, *J* = 12.4 Hz, 18H, PC(C***H***3)3), 0.69 (t, *J* = 7.1 Hz, 6H, NCH2C***H***3), -0.08 (q, *J* = 8.2 Hz, 2H, MgC***H***2CH3).

^{13}C NMR (101 MHz, C_6D_6) δ 167.61 (d, *J* = 17.9 Hz, Py***C***), 150.69 (d, *J* = 3.1 Hz, Py***C***), 132.83 (d, *J* = 2.3 Hz, Py***C***), 119.38 (d, *J* = 9.0 Hz, Py***C***), 98.64 (s, Py***C***), 67.44 (s, O***C***H2), 62.62 (d, *J* = 30.8 Hz, Py***C***HP), 57.76 (s, Py***C***H2N), 44.78 (s, N***C***H2CH3), 32.82 (d, *J* = 5.6 Hz, P***C***(CH3)3), 29.86 (d, *J* = 9.1 Hz, PC(***C***H3)3), 14.39 (s, MgCH2***C***H3), 9.20 (s, NCH2***C***H3), 2.76 (d, *J* = 35.9 Hz, Mg***C***H2CH3).

^{31}P NMR (162 MHz, C_6D_6) δ 0.43 (s).

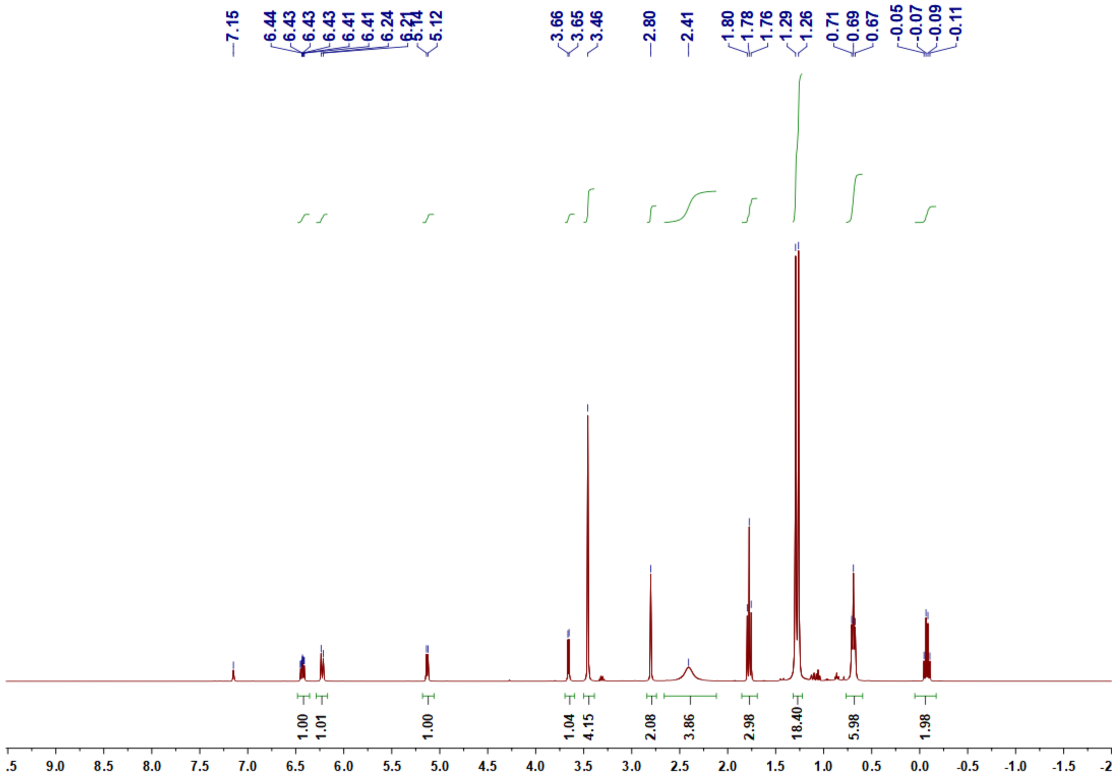

Figure S19. ^{1}H NMR spectrum of **Mg-3** in C_6D_6

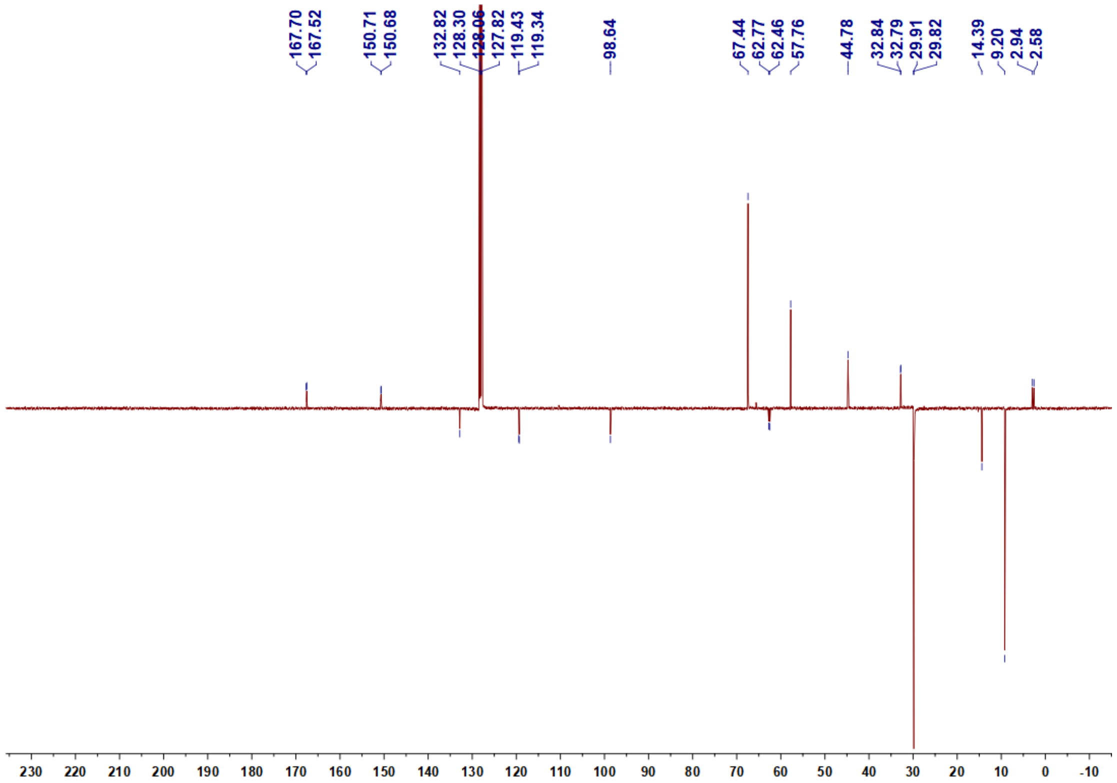

Figure S20. ^{13}C-DEPTQ NMR spectrum of **Mg-3** in C_6D_6

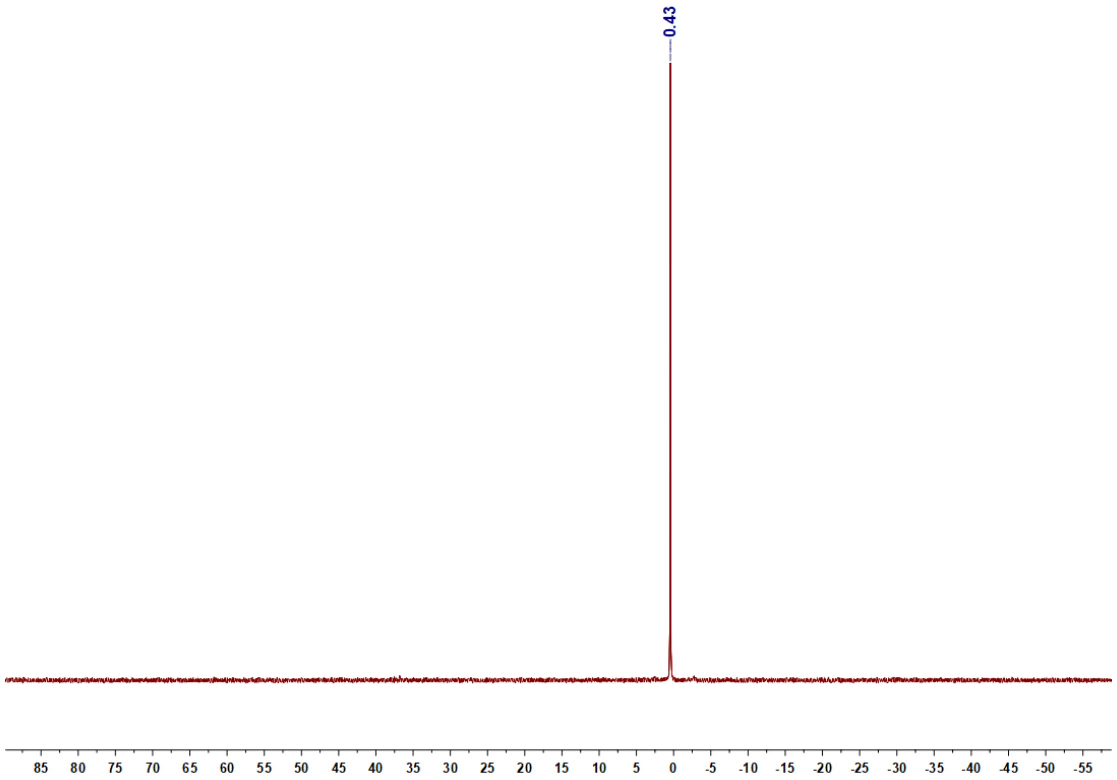

Figure S21. ^{31}P NMR spectrum of **Mg-3** in C_6D_6

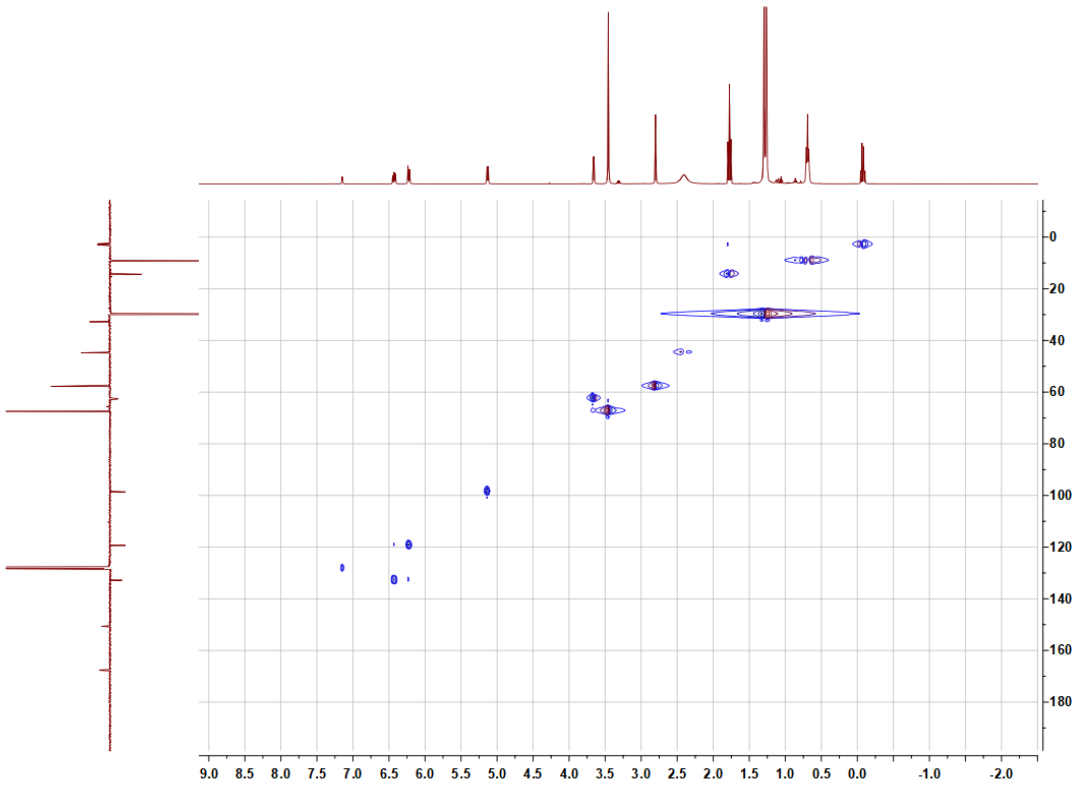

Figure S22. HSQC spectrum of **Mg-3** in C_6D_6

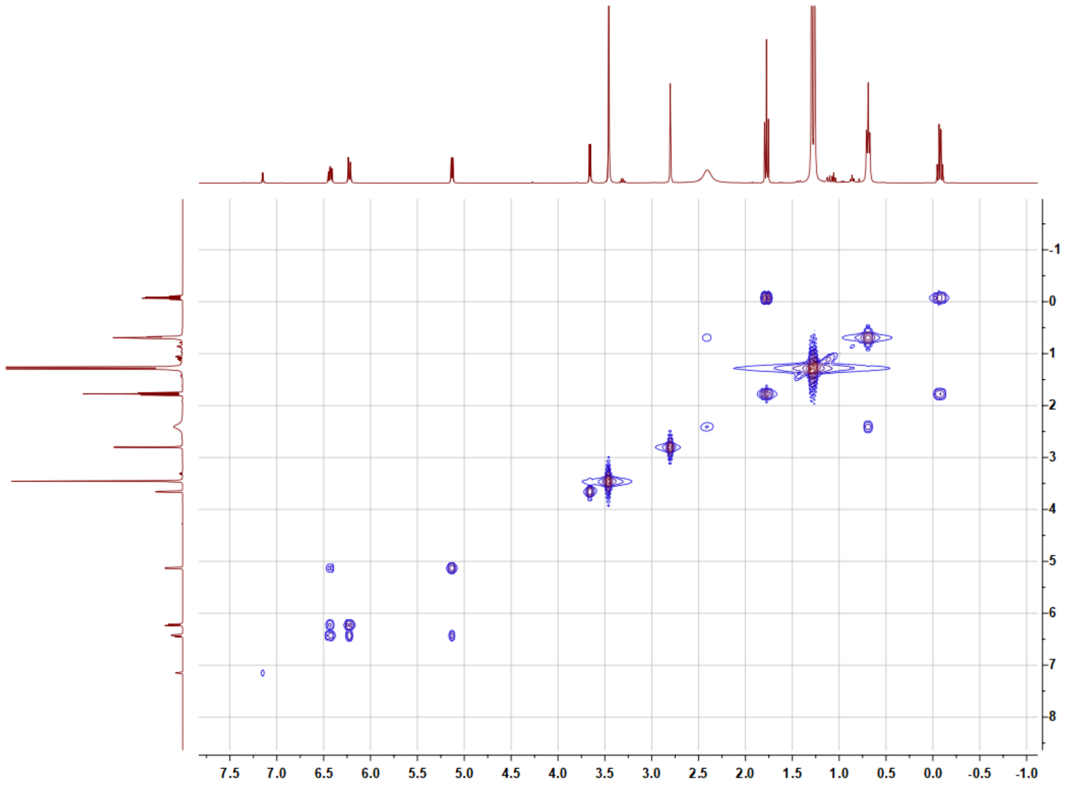

Figure S23. H-H COSY spectrum of **Mg-3** in C_6D_6

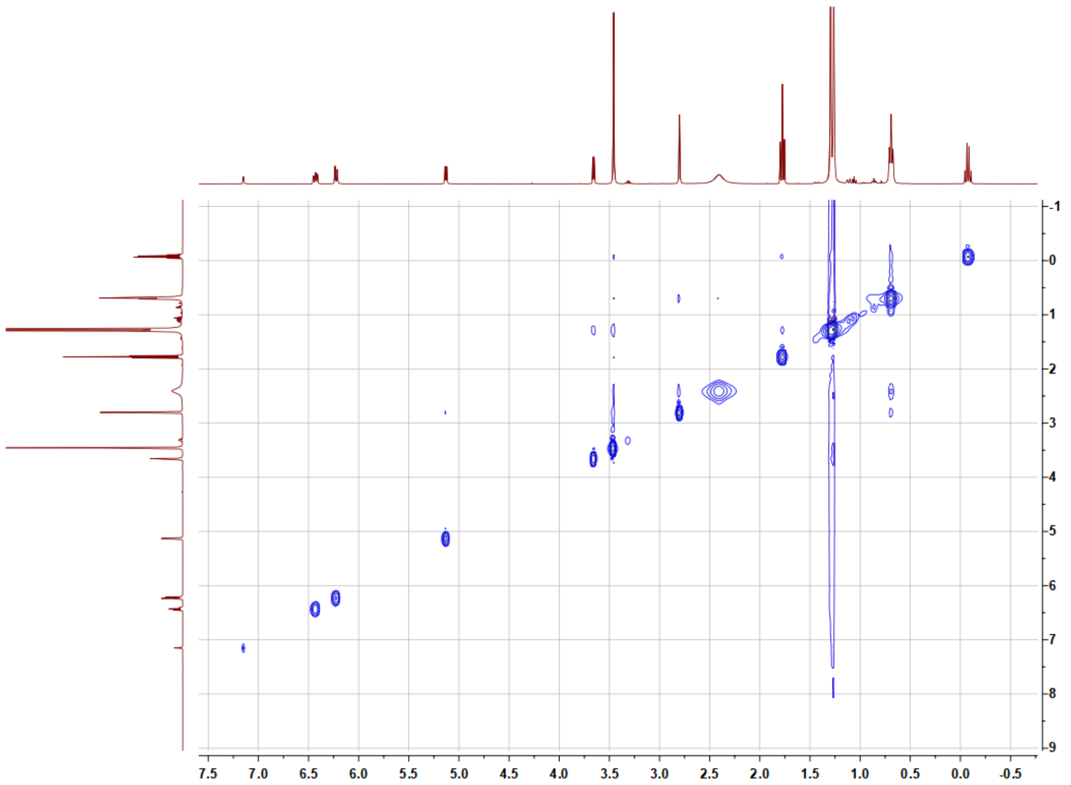

Figure S24. H-H NOSY spectrum of **Mg-3** in C_6D_6

The diffraction data from single crystals of **Mg-3** were collected on Rigaku Xtalab PRO diffractometer dual source equipped with Dectris Pilatus 200K detector and microfocus, with CuKα (λ=1.54184 Å). The data were processed with CrysAlis^{PRO} [5]. The structures were solved with SHELXT[6]. Full matrix least-squares and refined based on F^2 with SHELXL[7]. All structure solution and refinement programs are implemented in Olex-2 GUI[8]. All non-hydrogen atoms were refined with anisotropic displacement coefficients. Hydrogens were placed in calculated positions and refined in a riding mode. Supplementary crystallographic data have been deposited at the Cambridge Crystallographic Data Center (CCDC 2174063).

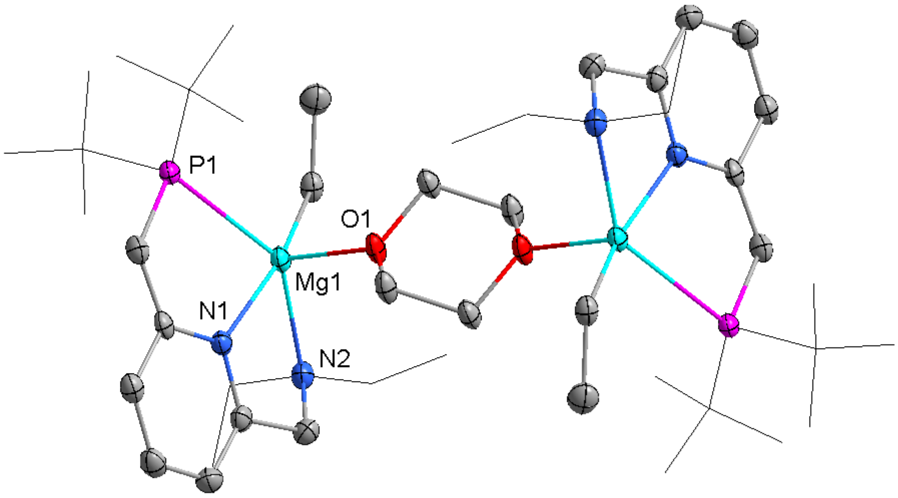

Figure S25. X-ray crystal structure of **Mg-3**

Table S4. Crystal data and structure refinement for Mg-3

Empirical formula	$C_{46}H_{86}Mg_2N_4O_2P_2$
Crystal description	Orange prism
Crystal size (mm³)	0.146 × 0.054 × 0.040
Formula weight (g/mol)	837.74
T (K)	120.0(2)
Wavelength (Å)	1.54184
Crystal system	Monoclinic
Space group	*P2₁/c*

a (Å)	15.49834(14)
b (Å)	11.80010(9)
c (Å)	14.41709(14)
α (°)	90
β (°)	111.2785(10)
γ (°)	90
Volume ($Å^3$)	2456.88(4)
Z	2
ρ_{cal} (mg/m^3)	1.341
μ (mm^{-1})	1.132
No. of reflection collected (Unique)	27541(5303)
R_{int}	0.0427
Completeness to θ (%)	99.9
Limiting indices	-18<=h<=17, -15<=k<=14, -18<=l<=18
Data\restraints\ parameters	5303/0/263
Goodness-of-fit on F^2	1.110
Final R_1 and wR_2 indices [I>2σ(I)]	R_1 = 0.0459, wR_2 = 0.1343
R_1 and wR_2 indices (all data)	R_1 = 0.0475, wR_2 = 0.1361
Largest diff. peak and hole ($e/Å^3$)	0.391 and -0.411

Table S5. Bond lengths for Mg-3

Atom	Atom	Length/Å	Atom	Atom	Length/Å
P1	Mg1	2.7516(6)	C2	C3	1.4465(19)
P1	C1	1.7690(15)	C3	C4	1.358(2)
P1	C8	1.8898(15)	C4	C5	1.421(2)
P1	C12	1.8987(14)	C5	C6	1.370(2)
Mg1	O1	2.1288(10)	C6	C7	1.510(2)
Mg1	N1	2.1440(12)	C8	C9	1.531(2)
Mg1	N2	2.3011(13)	C8	C10	1.534(2)
Mg1	C22	2.1783(14)	C8	C11	1.528(2)
O1	C20	1.4427(16)	C12	C13	1.536(2)
O1	C21	1.4456(16)	C12	C14	1.537(2)
N1	C2	1.3954(18)	C12	C15	1.530(2)

N1	C6	1.3582(18)	C16	C17	1.522(2)
N2	C7	1.4723(18)	C18	C19	1.525(2)
N2	C16	1.4875(18)	C20	C21[1]	1.5103(19)
N2	C18	1.4940(17)	C21	C20[1]	1.5104(19)
C1	C2	1.382(2)	C22	C23	1.512(2)

[1]1-X,1-Y,1-Z

Table S6. Bond angles for Mg-3

Atom	Atom	Atom	Angle/°	Atom	Atom	Atom	Angle/°
C1	P1	Mg1	94.62(5)	C2	C1	P1	121.95(11)
C1	P1	C8	105.30(7)	N1	C2	C3	117.46(12)
C1	P1	C12	104.28(6)	C1	C2	N1	121.93(12)
C8	P1	Mg1	125.89(5)	C1	C2	C3	120.60(13)
C8	P1	C12	110.24(6)	C4	C3	C2	121.40(13)
C12	P1	Mg1	112.59(5)	C3	C4	C5	119.93(13)
O1	Mg1	P1	100.43(3)	C6	C5	C4	117.14(13)
O1	Mg1	N1	93.49(4)	N1	C6	C5	124.91(13)
O1	Mg1	N2	104.30(4)	N1	C6	C7	114.75(12)
O1	Mg1	C22	104.98(5)	C5	C6	C7	120.28(13)
N1	Mg1	P1	76.67(4)	N2	C7	C6	111.49(11)
N1	Mg1	N2	75.90(5)	C9	C8	P1	106.28(10)
N1	Mg1	C22	161.07(5)	C9	C8	C10	107.54(13)
N2	Mg1	P1	143.85(4)	C10	C8	P1	109.67(10)
C22	Mg1	P1	103.46(4)	C11	C8	P1	114.44(10)
C22	Mg1	N2	95.18(5)	C11	C8	C9	109.10(14)
C20	O1	Mg1	127.64(8)	C11	C8	C10	109.55(14)
C20	O1	C21	110.40(10)	C13	C12	P1	113.91(10)
C21	O1	Mg1	120.18(8)	C13	C12	C14	108.40(12)
C2	N1	Mg1	124.31(9)	C14	C12	P1	102.73(9)
C6	N1	Mg1	116.66(9)	C15	C12	P1	113.47(10)
C6	N1	C2	119.02(12)	C15	C12	C13	109.09(12)
C7	N2	Mg1	103.32(9)	C15	C12	C14	108.87(13)
C7	N2	C16	110.36(11)	N2	C16	C17	113.32(12)
C7	N2	C18	111.26(11)	N2	C18	C19	116.57(13)
C16	N2	Mg1	119.29(9)	O1	C20	C21[1]	109.99(11)
C16	N2	C18	109.87(11)	O1	C21	C20[1]	110.47(12)
C18	N2	Mg1	102.35(8)	C23	C22	Mg1	120.36(10)

[1]1-X,1-Y,1-Z

2.4 Synthesis and characterization of Mg-4

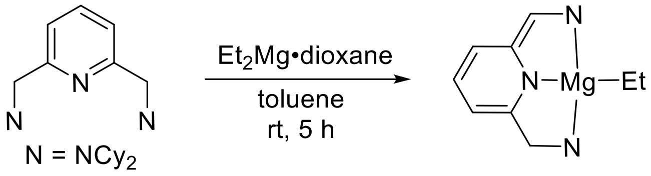

In a N_2 glovebox, the NNN ligand (232.7 mg, 0.5 mmol) and Et_2Mg·dioxane (127.6 mg, 0.75 mmol) were added to a 20 mL vial and then dissolved with toluene (5 mL). After stirring at room temperature for 5 h, the solvent was removed under vacuum to give an orange-red solid. To further purify the complex, the solid was dissolved in Et_2O and pentane. The solution was kept at room temperature until crystals were formed. The crystals were suitable for X-ray diffraction. Solvent was removed, and solid was dried under vacuum to give **Mg-4** as an orange-red solid (238.0 mg, 92% yield).

^{1}H NMR (400 MHz, C_6D_6) δ 6.41 (dd, *J* = 9.1, 6.0 Hz, 1H, Py***H***), 5.97 (d, *J* = 9.1 Hz, 1H, Py***H***), 4.95 (d, *J* = 6.0 Hz, 1H, Py***H***), 4.09 (s, 1H, PyC***H***N), 3.02 (s, 2H, PyC$\boldsymbol{H}_2$N), 2.83 – 2.74 (m, 2H, NC***H***$(CH_2)_2$), 2.73 – 2.65 (m, 2H, NC***H***$(CH_2)_2$), 2.12 (d, *J* = 11.9 Hz, 2H, Cy***H***), 2.06 – 1.90 (m, 2H, Cy***H***), 1.87 – 1.78 (m, 6H, Cy***H***), 1.78 (t, *J* = 8.2 Hz, 3H, $MgCH_2C\boldsymbol{H}_3$), 1.73 – 1.39 (m, 12H, Cy***H***), 1.37 – 1.24 (m, 4H, Cy***H***), 1.18 – 0.98 (m, 12H, Cy***H***), 0.94 – 0.80 (m, 2H, Cy***H***), 0.03 (q, *J* = 8.2 Hz, 2H, $MgC\boldsymbol{H}_2CH_3$).

^{13}C NMR (101 MHz, C_6D_6) δ 154.98 (Py***C***), 152.90 (Py***C***), 131.12 (Py***C***), 112.53 (Py***C***), 91.11 (Py***C*** and Py***C***HN), 61.91 (N***C***H$(CH_2)_2$), 61.04 (N***C***H$(CH_2)_2$), 53.08 (Py***C***H_2N), 31.42 (Cy***C***), 30.68 (Cy***C***), 29.06 (Cy***C***), 26.48 (Cy***C***), 26.45 (Cy***C***), 26.33 (Cy***C***), 26.28 (Cy***C***), 26.06 (Cy***C***), 14.12 ($MgCH_2\boldsymbol{C}H_3$), 3.37 ($Mg\boldsymbol{C}H_2CH_3$).

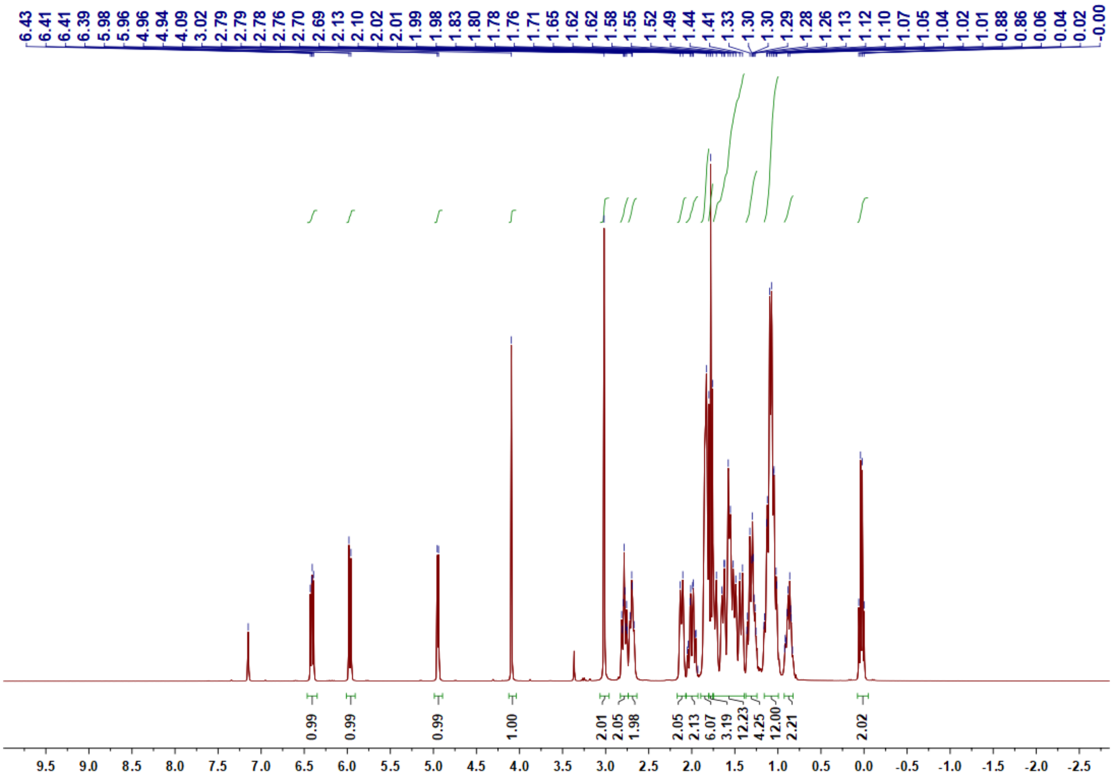

Figure S26. ^{1}H NMR spectrum of **Mg-4** in C_6D_6

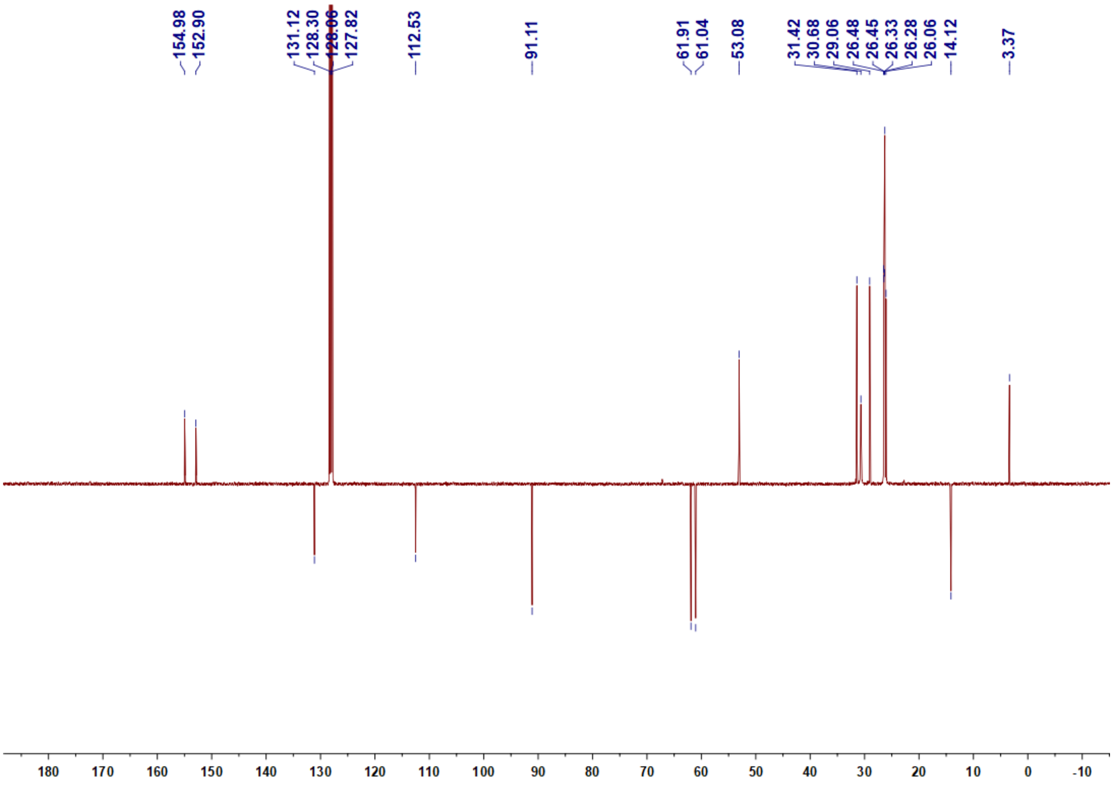

Figure S27. ^{13}C-DEPTQ NMR spectrum of **Mg-4** in C_6D_6

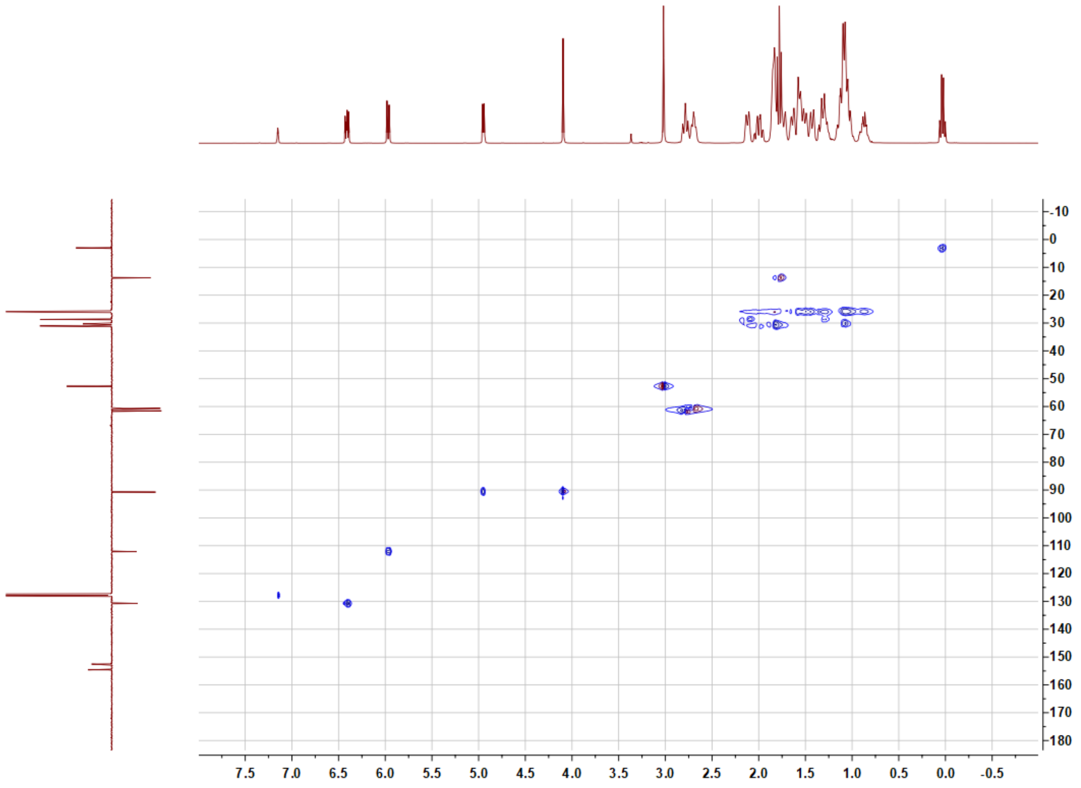

Figure S28. HSQC spectrum of **Mg-4** in C_6D_6

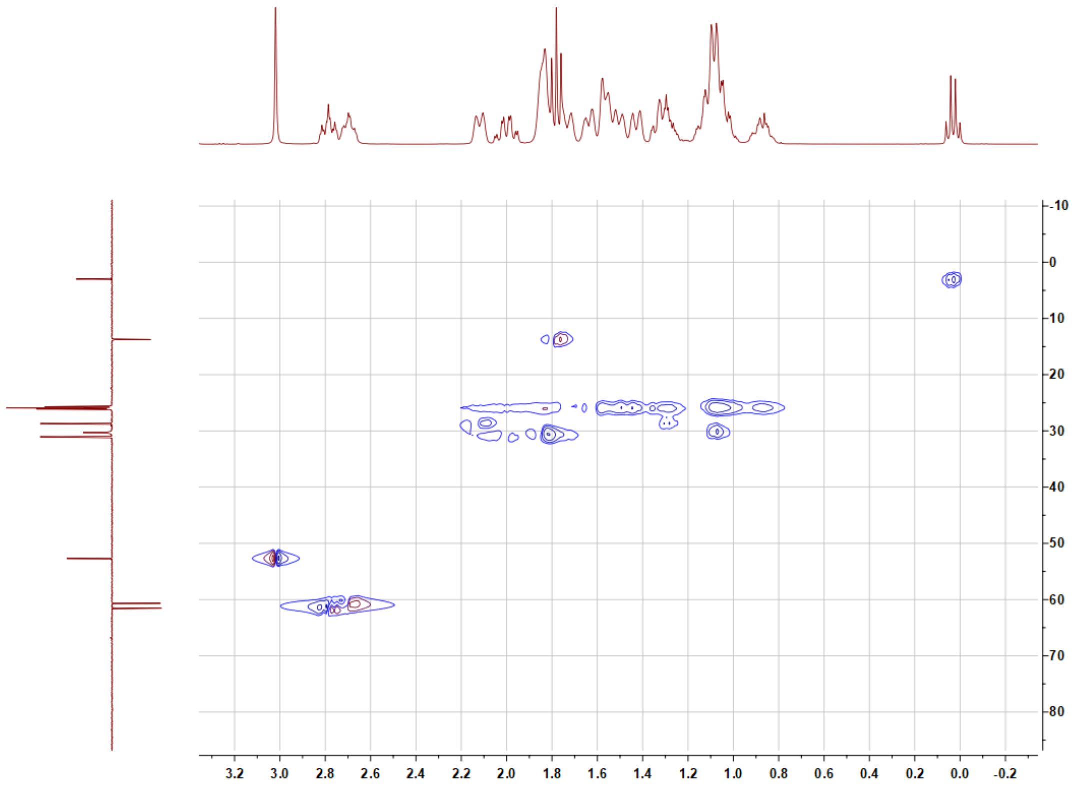

Figure S29. HSQC spectrum (zoom in) of **Mg-4** in C_6D_6

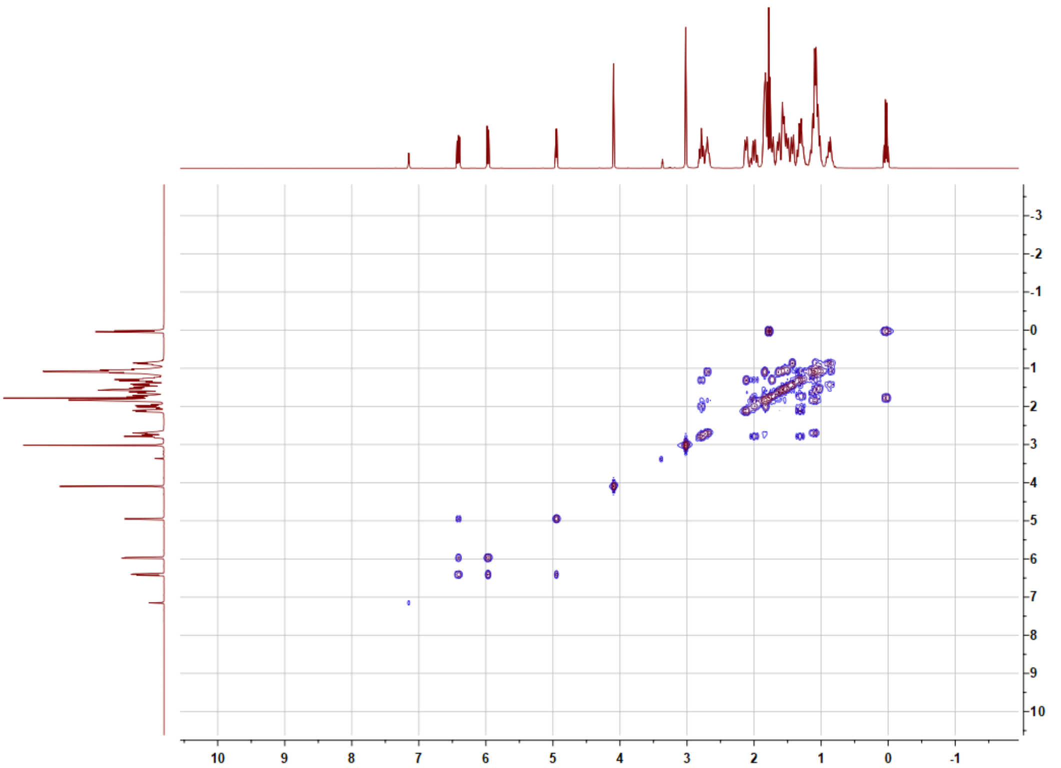

Figure S30. H-H COSY spectrum of **Mg-4** in C_6D_6

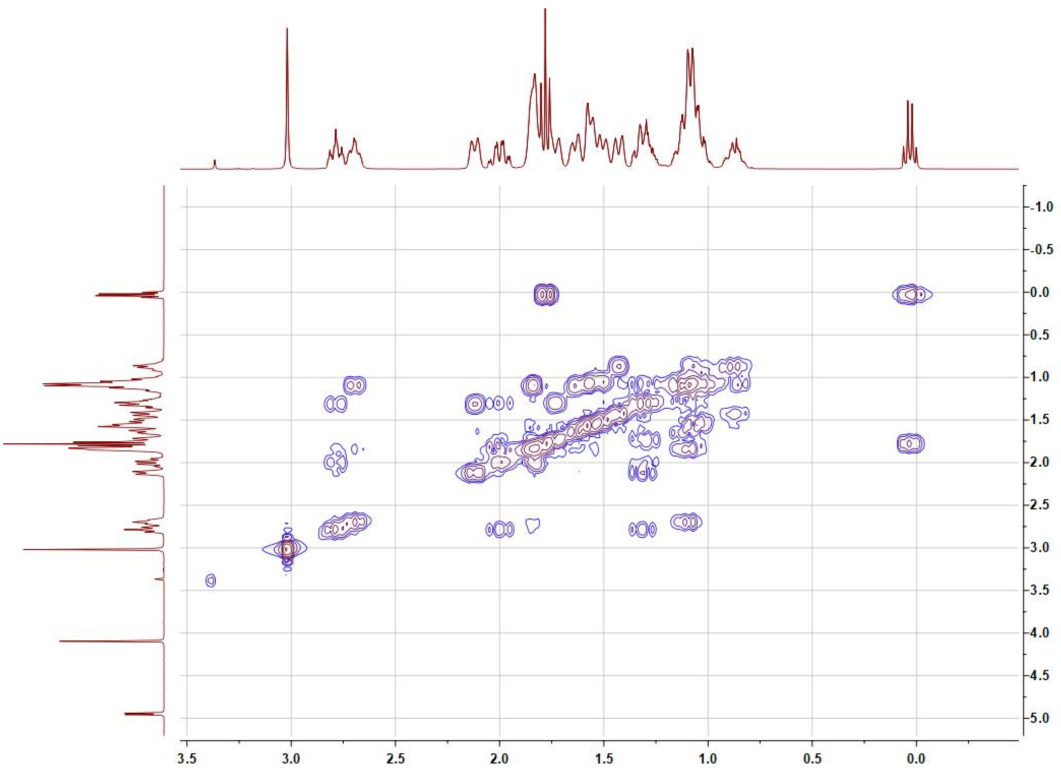

Figure S31. H-H COSY spectrum (zoom in) of **Mg-4** in C_6D_6

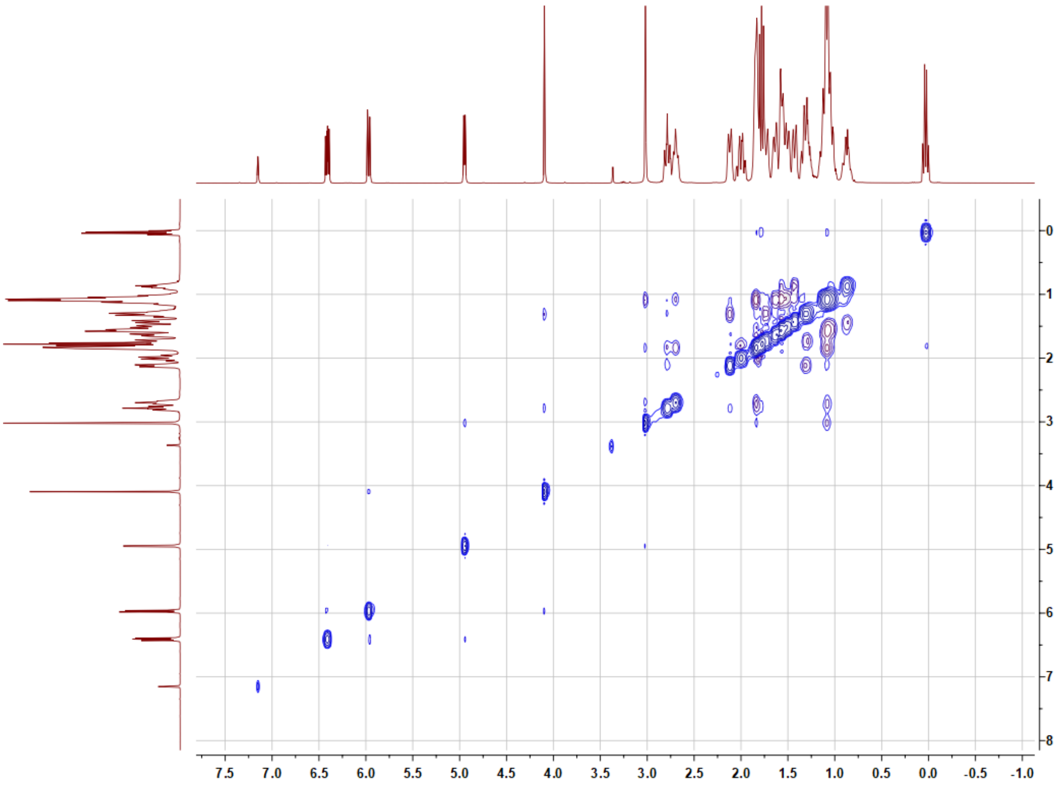

Figure S32. H-H NOSY spectrum of **Mg-4** in C_6D_6

The diffraction data from single crystals of **Mg-4** were collected on Rigaku Xtalab PRO diffractometer dual source equipped with Dectris Pilatus 200K detector and microfocus, with CuKα (λ=1.54184 Å). The data were processed with CrysAlis^{PRO} [5]. The structures were solved with SHELXT[6]. Full matrix least-squares and refined based on F^2 with SHELXL[7]. All structure solution and refinement programs are implemented in Olex-2 GUI[8]. All non-hydrogen atoms were refined with anisotropic displacement coefficients. Hydrogens were placed in calculated positions and refined in a riding mode. Supplementary crystallographic data have been deposited at the Cambridge Crystallographic Data Center (CCDC 2174064).

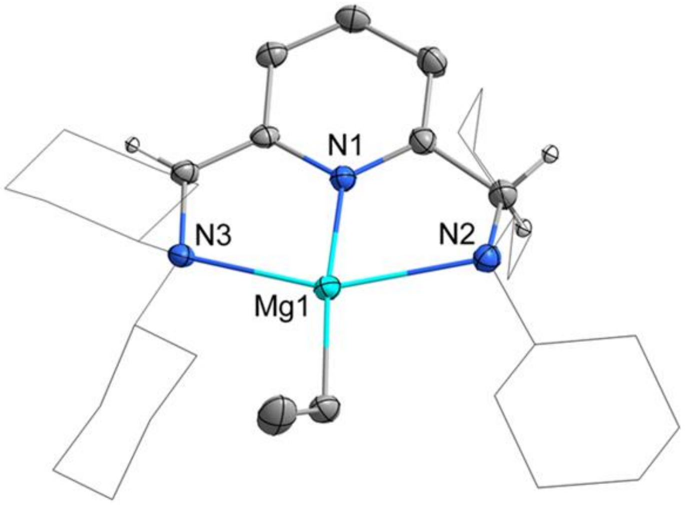

Figure S33. X-ray crystal structure of **Mg-4**

Table S7. Crystal data and structure refinement for Mg-4

Empirical formula	$C_{33}H_{55}MgN_3$
Crystal description	Orange chunk
Crystal size (mm^3)	0.085 × 0.056 × 0.034
Formula weight (g/mol)	518.11
T (K)	120.0(2)
Wavelength (Å)	1.54184
Crystal system	Triclinic
Space group	*P-1*
a (Å)	9.12514(13)
b (Å)	9.42476(14)
c (Å)	19.5034(2)
α (°)	99.5105(11)
β (°)	90.9695(10)
γ (°)	110.5742(13)
Volume ($Å^3$)	1543.65(4)
Z	2
ρ_{cal} (mg/m^3)	1.115
μ (mm^{-1})	0.665

No. of reflection collected (Unique)	25923(6569)
R_{int}	0.0515
Completeness to θ (%)	99.7
Limiting indices	-10<=h<=11, -11<=k<=12, -24<=l<=24
Data\restraints\ parameters	6569/0/335
Goodness-of-fit on F^2	1.113
Final R_1 and wR_2 indices [I>2σ(I)]	R_1 = 0.0504, wR_2 = 0.1382
R_1 and wR_2 indices (all data)	R_1 = 0.0546, wR_2 = 0.1419
Largest diff. peak and hole (e/Å^3)	0.340 and -0.436

Table S8. Bond lengths for Mg-4

Atom	Atom	Length/Å	Atom	Atom	Length/Å
Mg1	N1	2.0333(12)	C11	C12	1.526(2)
Mg1	N2	2.4354(12)	C12	C13	1.534(2)
Mg1	N3	2.2943(11)	C14	C15	1.533(2)
Mg1	C33	2.1507(14)	C14	C19	1.530(2)
N1	C2	1.3532(17)	C15	C16	1.522(2)
N1	C6	1.3941(16)	C16	C17	1.516(3)
N2	C1	1.4883(18)	C17	C18	1.523(3)
N2	C8	1.5011(17)	C18	C19	1.528(2)
N2	C14	1.4991(18)	C21	C22	1.531(2)
N3	C7	1.4617(16)	C21	C26	1.5260(18)
N3	C26	1.5011(16)	C22	C23	1.524(2)
N3	C27	1.5056(16)	C23	C24	1.522(2)
C1	C2	1.5023(18)	C24	C25	1.525(2)
C2	C3	1.3701(19)	C25	C26	1.5301(18)
C3	C4	1.427(2)	C27	C28	1.5280(19)
C4	C5	1.356(2)	C27	C32	1.5248(18)
C5	C6	1.4450(18)	C28	C29	1.528(2)
C6	C7	1.3658(18)	C29	C30	1.522(2)
C8	C9	1.531(2)	C30	C31	1.520(2)
C8	C13	1.526(2)	C31	C32	1.5309(19)
C9	C10	1.531(2)	C33	C34	1.533(2)
C10	C11	1.515(3)			

Table S9. Bond angles for Mg-4

Atom	Atom	Atom	Angle/°	Atom	Atom	Atom	Angle/°
N1	Mg1	N2	75.52(4)	N2	C8	C13	111.96(11)
N1	Mg1	N3	79.91(4)	C13	C8	C9	110.30(12)
N1	Mg1	C33	173.71(5)	C10	C9	C8	110.84(13)
N3	Mg1	N2	154.91(4)	C11	C10	C9	111.62(13)
C33	Mg1	N2	99.57(5)	C10	C11	C12	111.09(13)
C33	Mg1	N3	105.28(5)	C11	C12	C13	111.18(14)
C2	N1	Mg1	124.28(9)	C8	C13	C12	110.64(12)
C2	N1	C6	119.54(11)	N2	C14	C15	110.24(11)
C6	N1	Mg1	115.79(8)	N2	C14	C19	113.02(12)
C1	N2	Mg1	105.13(8)	C19	C14	C15	109.87(13)
C1	N2	C8	110.15(11)	C16	C15	C14	112.31(13)
C1	N2	C14	109.53(11)	C17	C16	C15	111.75(15)
C8	N2	Mg1	108.14(8)	C16	C17	C18	109.88(15)
C14	N2	Mg1	111.18(8)	C17	C18	C19	111.89(15)
C14	N2	C8	112.46(10)	C18	C19	C14	112.14(14)
C7	N3	Mg1	104.42(7)	C26	C21	C22	111.75(12)
C7	N3	C26	111.36(10)	C23	C22	C21	111.54(12)
C7	N3	C27	108.00(9)	C24	C23	C22	110.47(12)
C26	N3	Mg1	105.55(7)	C23	C24	C25	110.60(12)
C26	N3	C27	113.03(10)	C24	C25	C26	111.32(12)
C27	N3	Mg1	114.18(7)	N3	C26	C21	110.06(10)
N2	C1	C2	114.45(11)	N3	C26	C25	114.03(11)
N1	C2	C1	115.21(11)	C21	C26	C25	110.51(11)
N1	C2	C3	123.89(12)	N3	C27	C28	109.07(10)
C3	C2	C1	120.79(12)	N3	C27	C32	115.52(11)
C2	C3	C4	117.53(12)	C32	C27	C28	109.35(11)
C5	C4	C3	120.50(12)	C29	C28	C27	111.73(12)
C4	C5	C6	120.12(12)	C30	C29	C28	111.08(12)
N1	C6	C5	118.33(11)	C31	C30	C29	110.92(13)
C7	C6	N1	119.06(11)	C30	C31	C32	112.36(12)
C7	C6	C5	122.61(12)	C27	C32	C31	110.21(11)
C6	C7	N3	120.56(11)	C34	C33	Mg1	122.38(11)
N2	C8	C9	114.86(12)				

3 Hydrogen activation by magnesium pincer complexes

3.1 Reaction of Mg-1 with H_2

A toluene-d_8 solution of **Mg-1** was charged with 5 bar of H_2, only a trace amount of

free ligand was detected by ^{31}P NMR after 24 h at room temperature (with rotation).

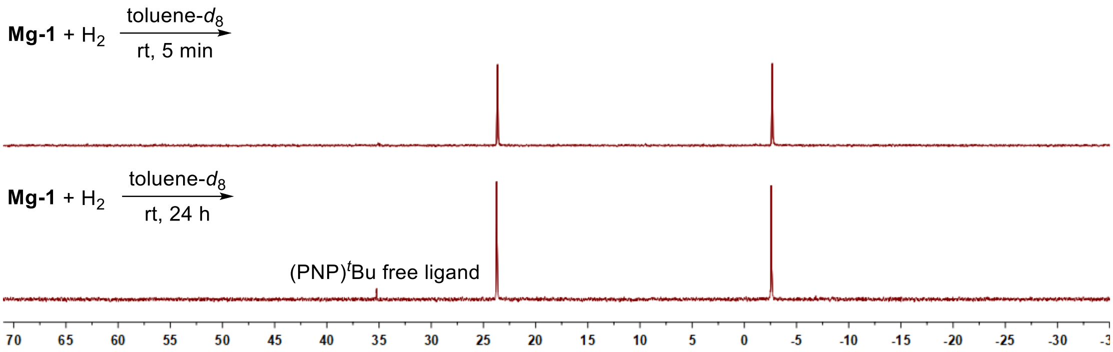

Figure S34. ^{31}P NMR spectra of the reaction of **Mg-1** with H_2 at room temperature

Next, the temperature was increased to 65 °C. Gradually decomposition of **Mg-1** to the free ligand was observed by ^{31}P NMR, and **Mg-5** was also gradually formed. Increasing the temperature to 120 °C, **Mg-1** was completely transformed into **Mg-5** and the free ligand after 36 h. After heating the solution at 120 °C for 4 d, the majority of **Mg-1** and **Mg-5** disappeared, and the free ligand was formed as the major product.

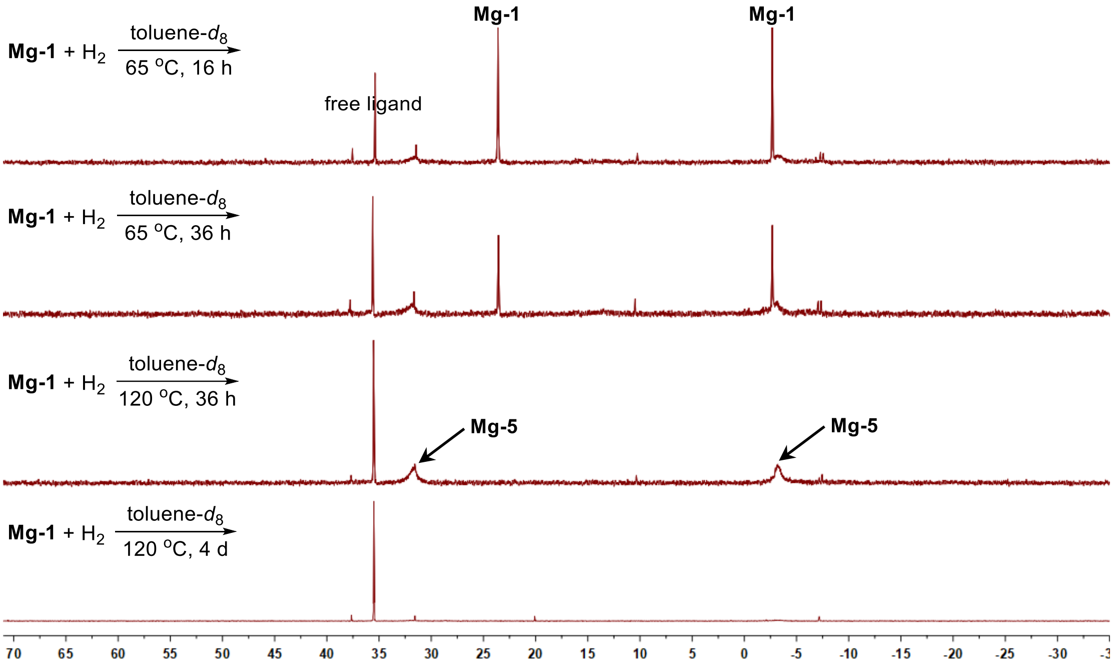

Figure S35. ^{31}P NMR spectra of the reaction of **Mg-1** with H_2 at different temperatures

It should be noted that CH_3CH_3 was detected by ^{1}H NMR during the reaction (0.81 ppm, s). And a triplet (J = 6.6 Hz) at 0.88 ppm in the ^{1}H NMR spectrum was also

detected. The signal may be assigned to the C***H***$_3$ of EtMgH species, which is derived from the decomposition of **Mg-1**.

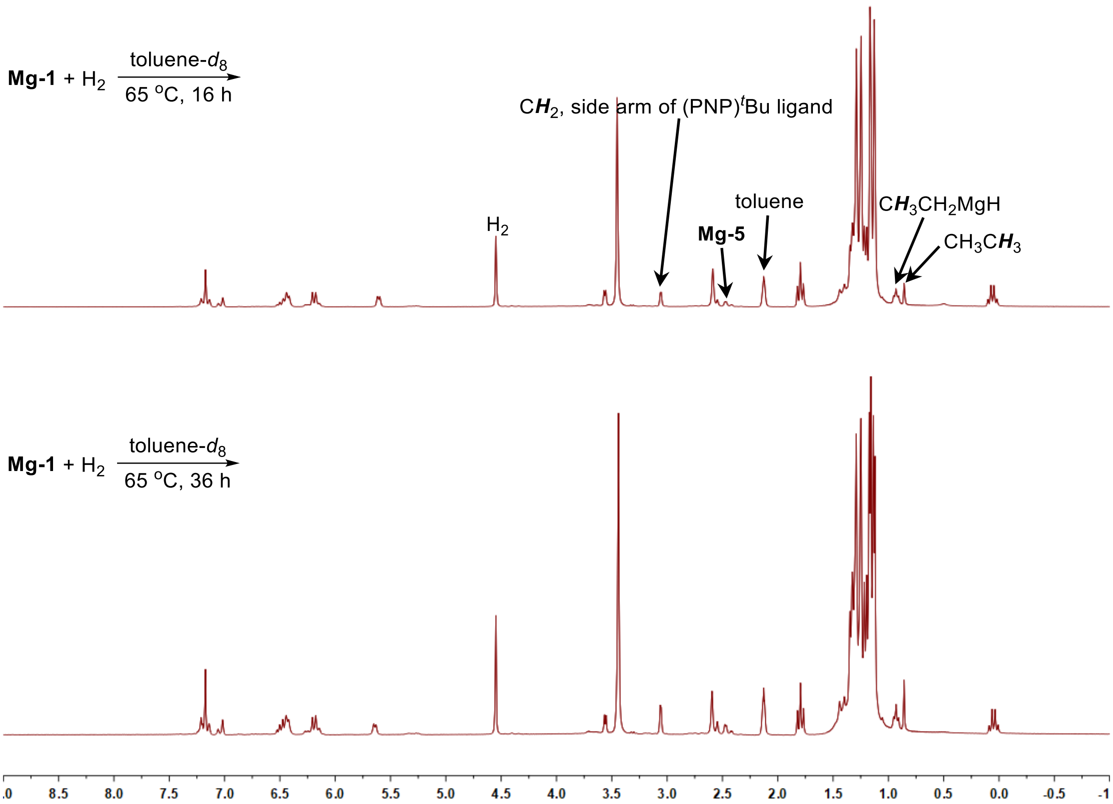

Figure S36. ^{1}H NMR spectra of the reaction of **Mg-1** with H_2

In order to determine the structure of **Mg-5**, 1 bar of H_2 and 80 °C of the reaction temperature were employed to reduce the decomposition.

*Procedures to obtain **Mg-5** in high yield:*

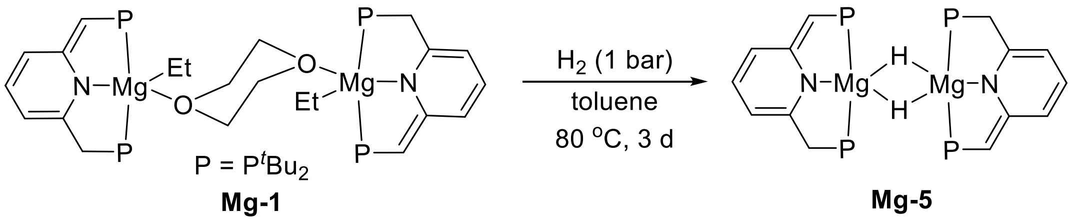

In a N_2 glovebox, **Mg-1** (19.6 mg, 0.02 mmol) was dissolved by toluene (0.7 mL) in a J. Young NMR tube. And then, the NMR tube was taken out from the glovebox and pressurized with 1 bar of H_2. The solution was heated at 80 °C until the full consumption of **Mg-1**. The NMR tube was put into the glovebox, and the solvent was

removed under the vacuum. The residue was dissolved by benzene-d_6, and the solution was measured by NMR to determine the structure.

^{1}H NMR (400 MHz, C_6D_6) δ 6.51 – 6.46 (m, 2H, Py***H***), 6.17 (s, 1H, Py***H***), 3.46 (s, 1H, PyC***H***P), 2.59 (d, *J* = 15.8 Hz, 1H, PyC$\boldsymbol{H}_2$P), 2.46 (dd, *J* = 15.8, 4.3 Hz, 1H, PyC$\boldsymbol{H}_2$P), 1.35 – 1.27 (m, 18H, PC(C$\boldsymbol{H}_3$)$_3$), 1.22 (d, *J* = 11.1 Hz, 9H, PC(C$\boldsymbol{H}_3$)$_3$), 1.17 (d, *J* = 10.6 Hz, 9H, PC(C$\boldsymbol{H}_3$)$_3$).

^{13}C NMR (101 MHz, C_6D_6) δ 169.95 – 169.28 (m, Py***C***), 156.82 (d, *J* = 16.2 Hz, Py***C***), 134.21 (s, Py***C***), 118.77 – 118.55 (m, Py***C***), 105.66 (d, *J* = 27.8 Hz, Py***C***), 57.40 (d, *J* = 32.5 Hz, Py***C***HP), 31.94 (d, *J* = 25.4 Hz, Py***C***H$_2$P), 31.50 (d, *J* = 21.9 Hz, Py***C***H$_2$P), 31.04 (d, *J* = 26.0 Hz, P***C***(CH$_3$)$_3$), 30.74 – 30.58 (m, PC(***C***H$_3$)$_3$), 30.40 (d, *J* = 11.6 Hz, PC(***C***H$_3$)$_3$), 30.26 (d, *J* = 12.3 Hz, PC(***C***H$_3$)$_3$).

^{31}P NMR (162 MHz, C_6D_6) δ 31.77 (br, PyCH$_2$***P***), -3.14 (br, PyCH***P***).

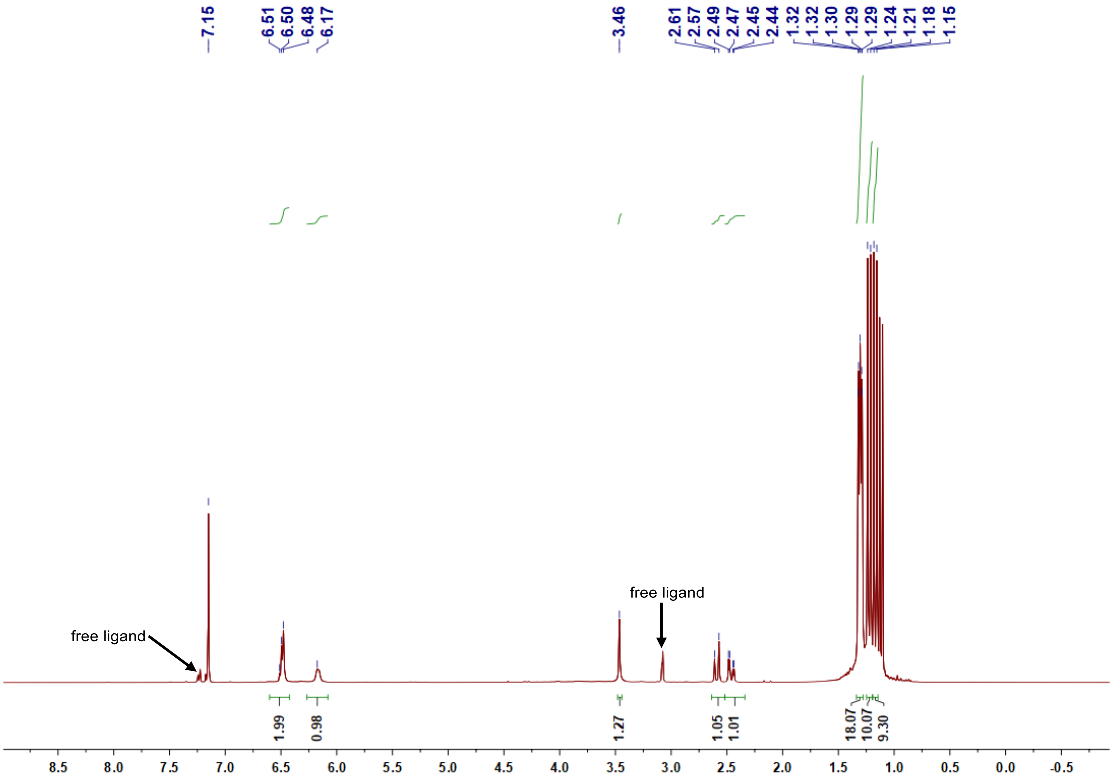

Figure S37. ^{1}H NMR spectrum of **Mg-5** in C_6D_6

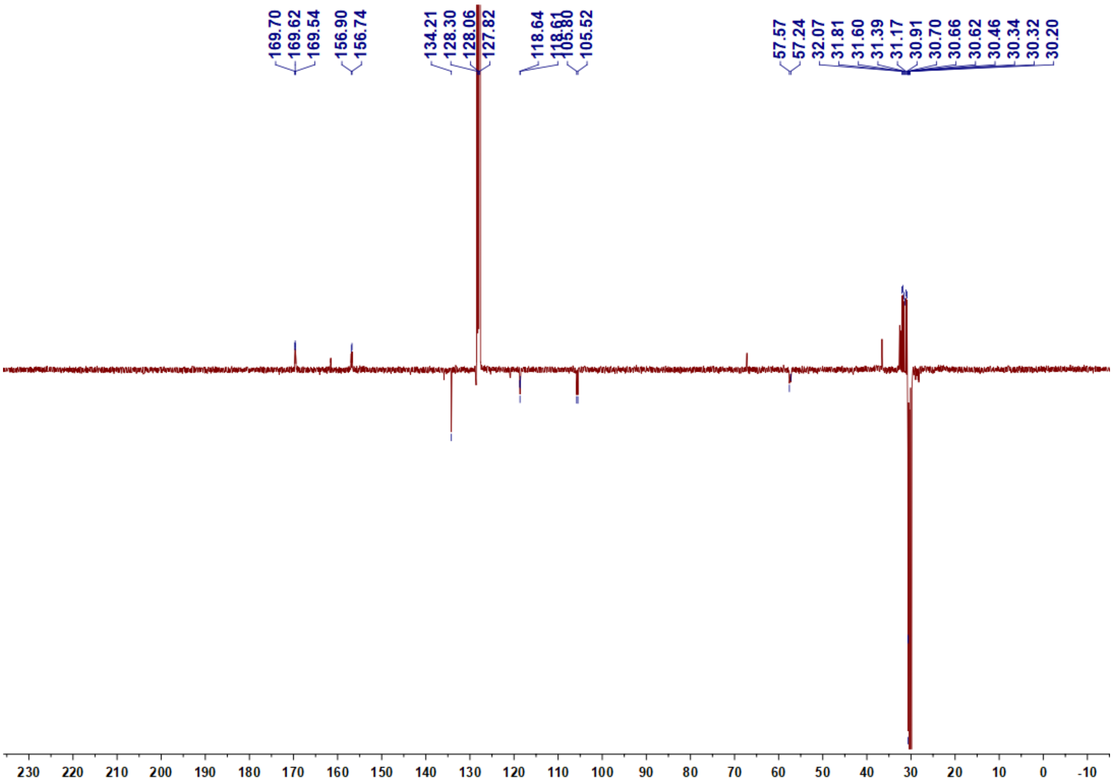

Figure S38. ^{13}C-DEPTQ NMR spectrum of **Mg-5** in C_6D_6

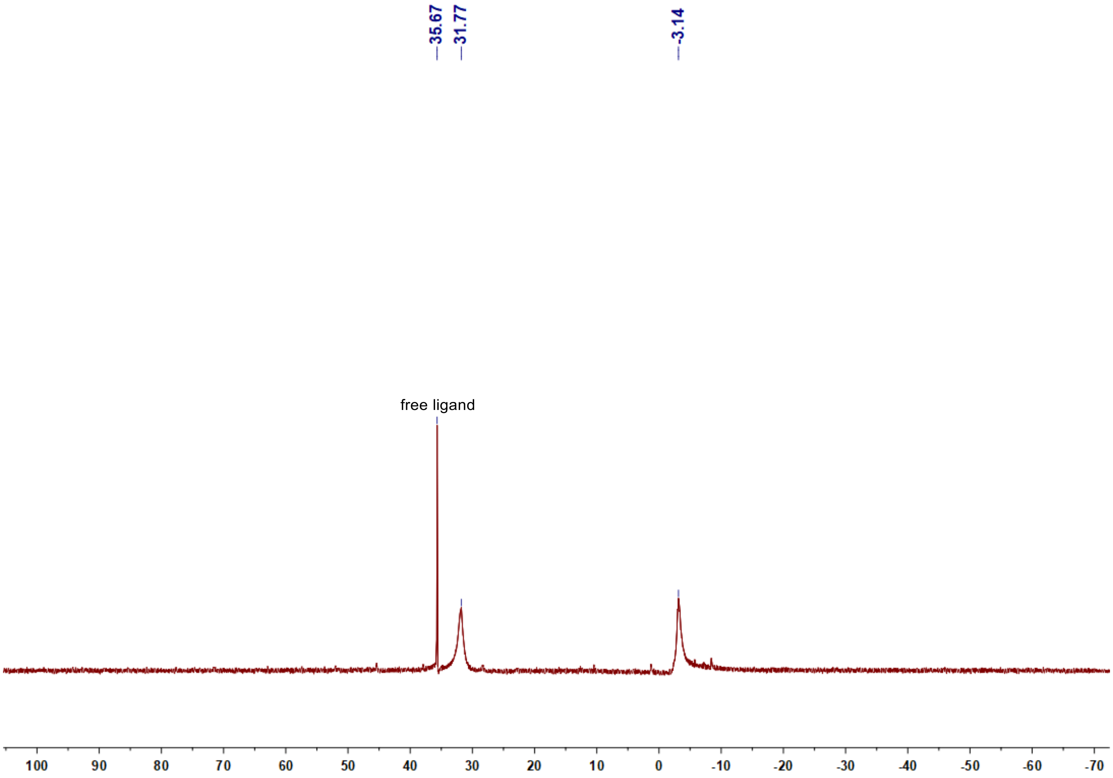

Figure S39. ^{31}P NMR spectrum of **Mg-5** in C_6D_6

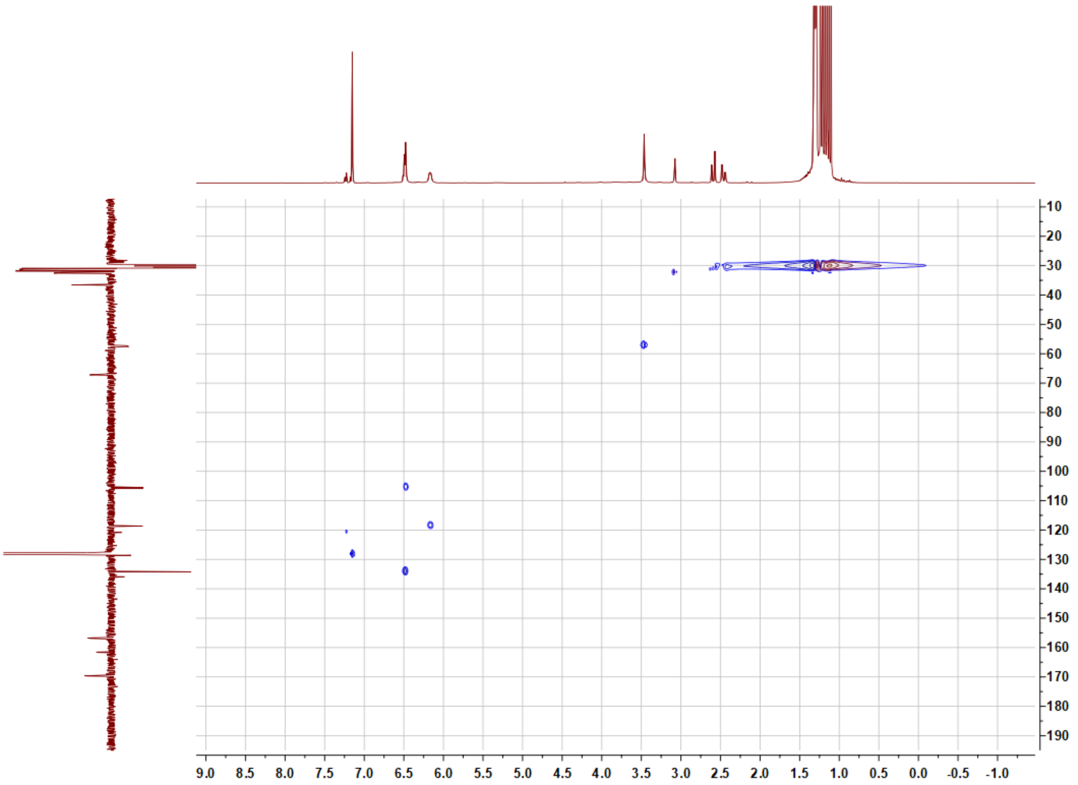

Figure S40. HSQC spectrum of **Mg-5** in C_6D_6

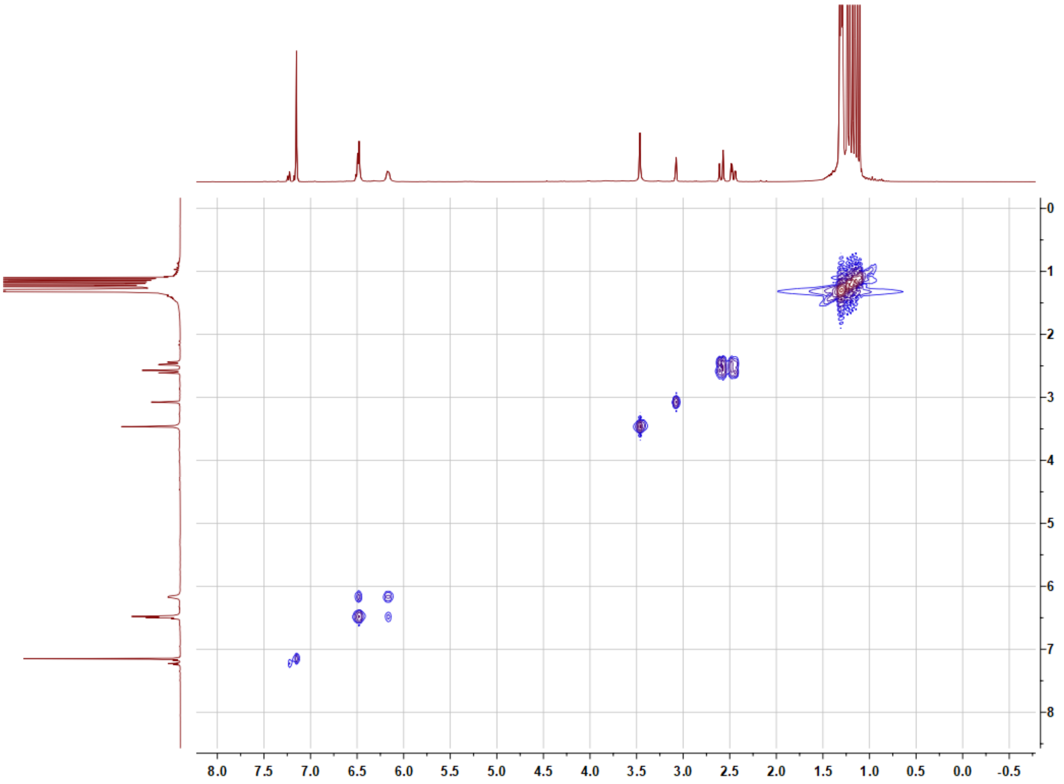

Figure S41. H-H COSY spectrum of **Mg-5** in C_6D_6

Another parallel experiment was carried out to determine the byproducts of the reaction. After the consumption of **Mg-1**, the gas in the NMR tube was directly analyzed by GC. An ethane signal was observed in the GC trace.

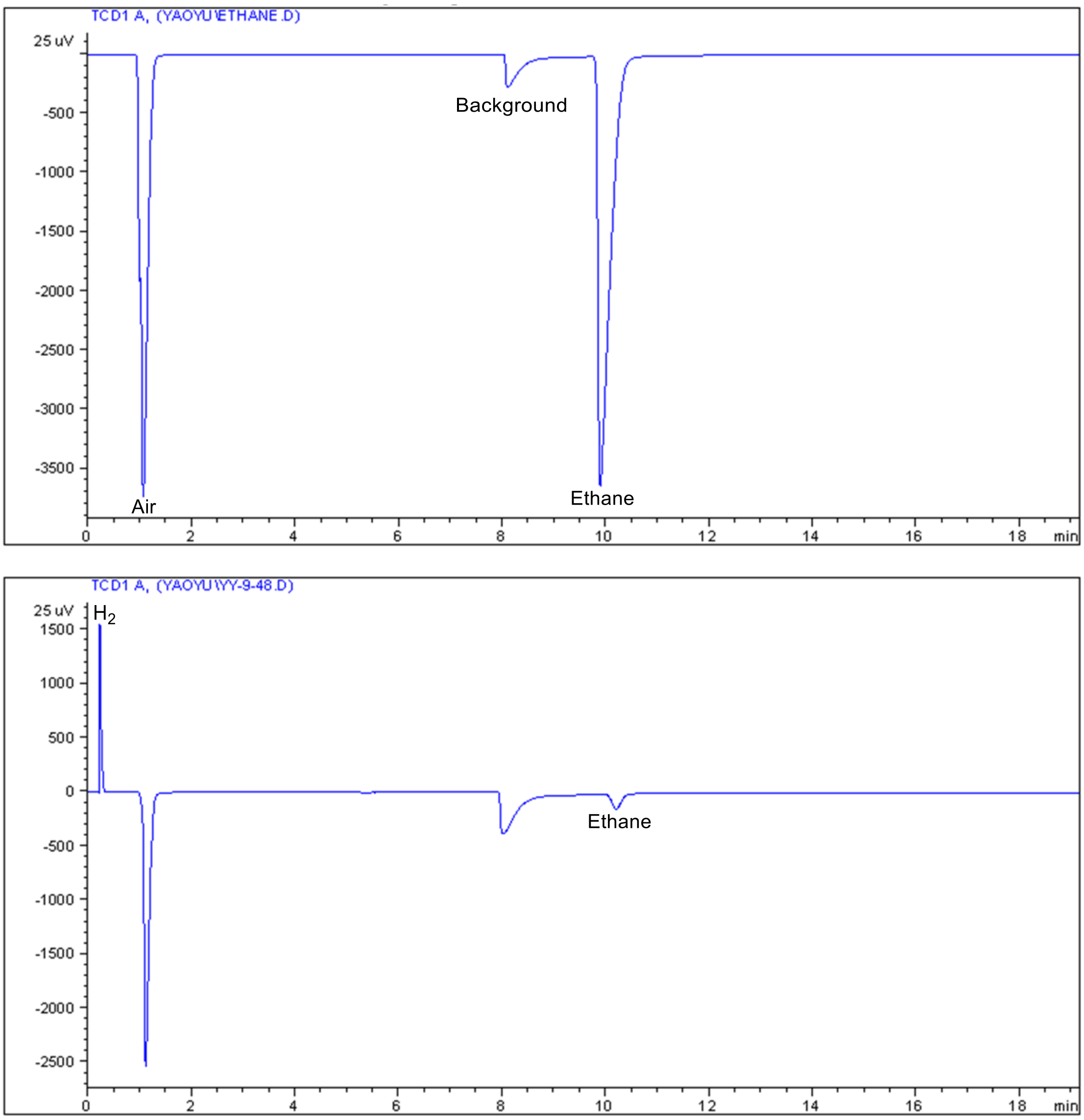

Figure S42. GC-gas trace of collected gas of the reaction (top, trace of standard ethane; bottom, trace of the collected gas from the reaction), N_2 was used as the carrier gas

To make clear that the formation of **Mg-5** is a monomer or a dimer, a DOSY NMR experiment was performed to compare the diffusion coefficients of **Mg-5** with the monomeric **Mg-4**. The results showed that **Mg-5** diffuses slower than **Mg-4**, indicating the larger structure of **Mg-5**. The diffusion coefficients of **Mg-5** (0.661×10^{5} $cm^{2} \cdot s^{-1}$) and **Mg-4** (0.717×10^{5} $cm^{2} \cdot s^{-1}$) suggest that **Mg-5** is a dimer.

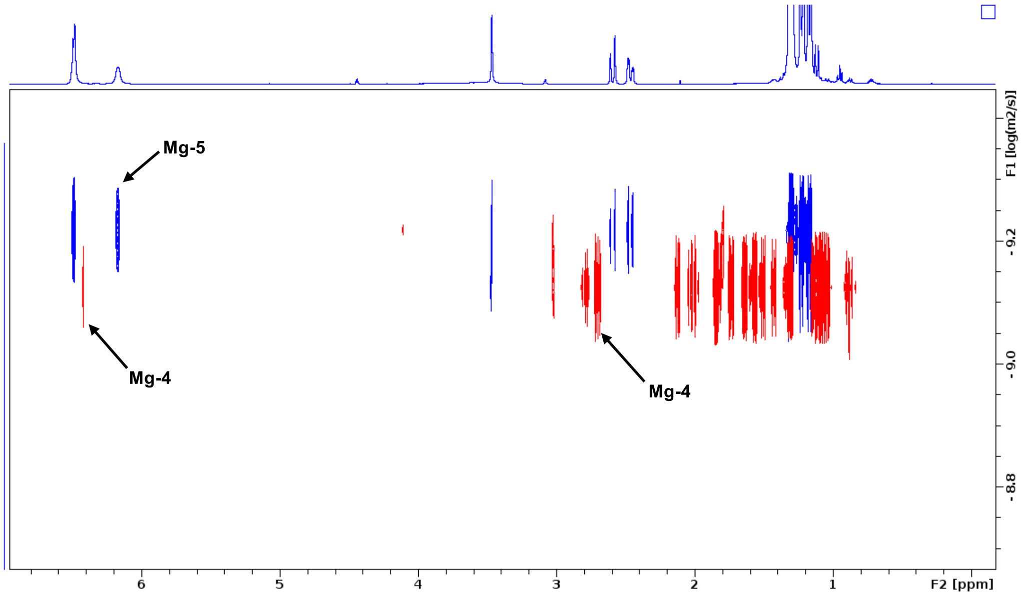

Figure S43. 2D DOSY ^{1}H NMR spectrum of **Mg-4** and **Mg-5**

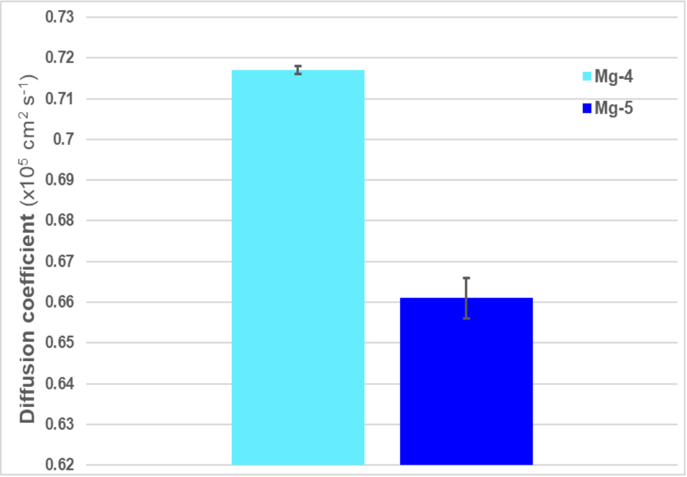

Figure S44. Diffusion coefficients of **Mg-4** and **Mg-5**

3.2 Reaction of Mg-1 with D_2

To prove the assumption that H_2 addition to **Mg-1** can proceed via the MLC process, 5 bar of D_2 was added to the toluene solution of **Mg-1** in a J. Young NMR tube. After heating at 65 °C for 16 h, partial decomposition of the complex to free ligand was detected in the $^{31}P\{^{1}H\}$ NMR spectrum. The phosphorus signals of free ligand and **Mg-1** were split into multiplets. Such a phenomenon implies the incorporation of deuterium into both side arms of the free ligand and **Mg-1**, leading to the formation of different types of phosphorus nuclei. **Mg-5-D**, with deuterium

coordinated to the magnesium center and incorporated into both side arms of **Mg-5**, is also generated, as determined by ^{31}P NMR and ^{2}H NMR spectroscopy. Notably, After the solution was heated at the same temperature for 3 days, **Mg-1-D** was completely converted to **Mg-5-D** together with the elimination of CH_3CH_2D. An Mg-*D* signal appears at 1.32 ppm in the ^{2}H NMR spectrum with a similar chemical shift to the tertiary butyl groups of the ligand, further confirming the structure of **Mg-5** and **Mg-5-D**. These results suggest that D_2 (H_2) is reversibly activated by **Mg-1** via the MLC process.

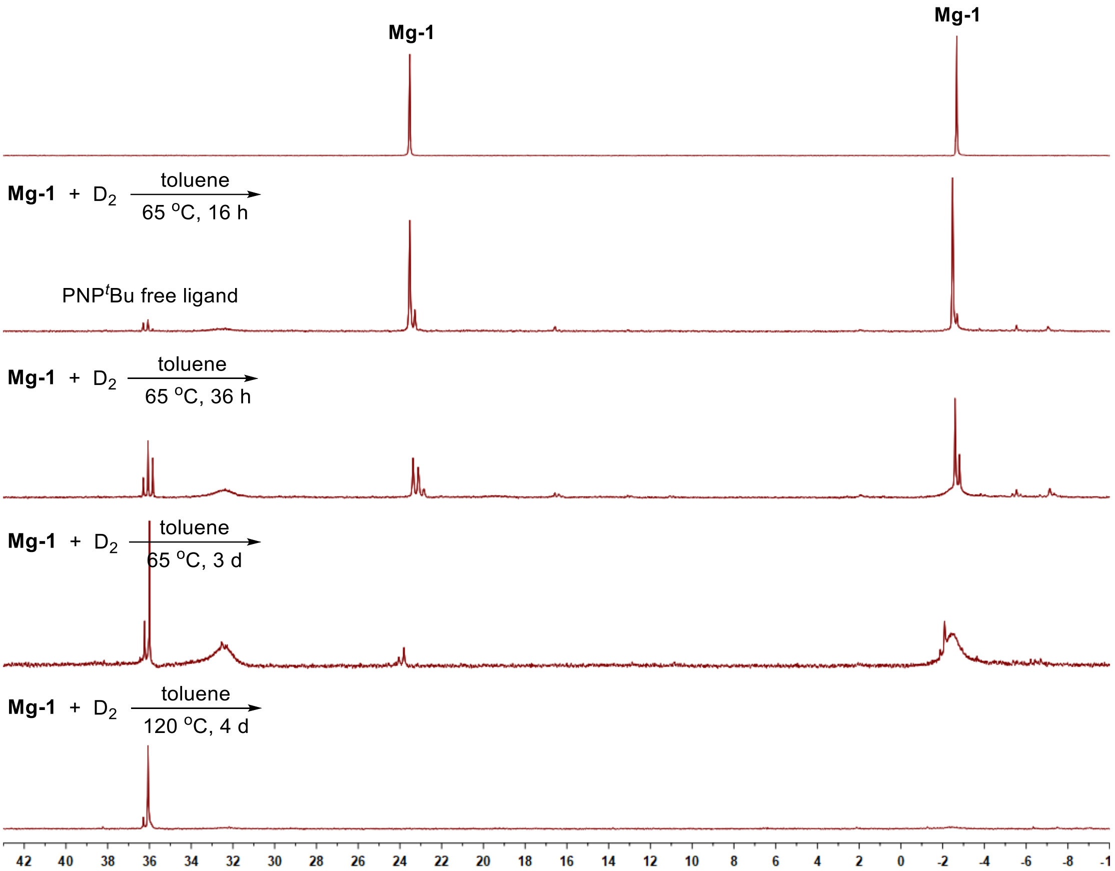

Figure S45. ^{31}P NMR spectra of the reaction of **Mg-1** and D_2 at different conditions

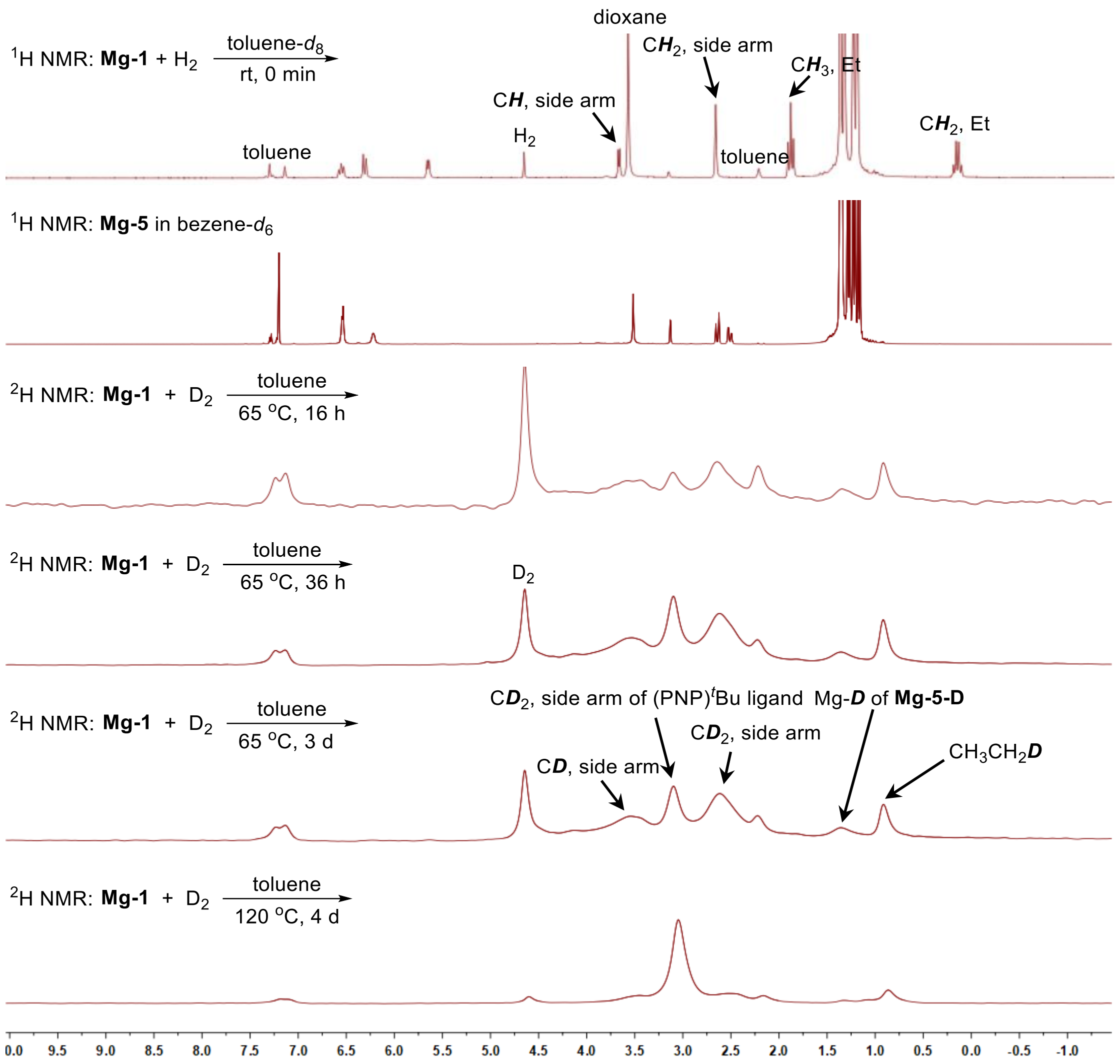

Figure S46. ^{2}H NMR spectra of the reaction of **Mg-1** and D_2

Note: the ^{2}H NMR signals of **Mg-1-D** overlap with the signals of **Mg-5-D** because of the similar chemical shifts.

After heating the above solution at 120 °C for 4 d, the toluene was removed under vacuum and redissolved by benzene-d_6. The ^{31}P NMR spectrum exhibits one major peak of the free ligand.

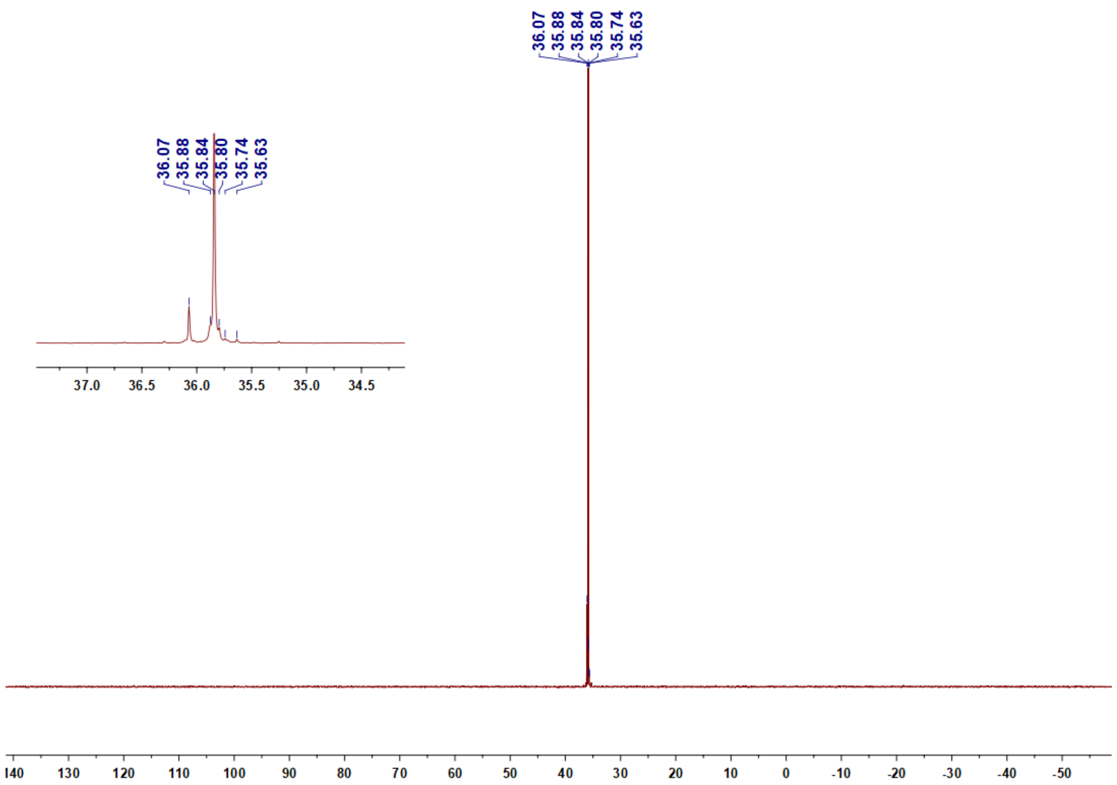

Figure S47. ^{31}P NMR spectrum after removing toluene and redissolving in C_6D_6

The integration of the methylene protons in the ^{1}H NMR spectrum confirmed that 95% deuterium was incorporated into the side arm of the free ligand. This result further supports the activation of D_2 (H_2) via the MLC process, and most importantly, the activation process is reversible.

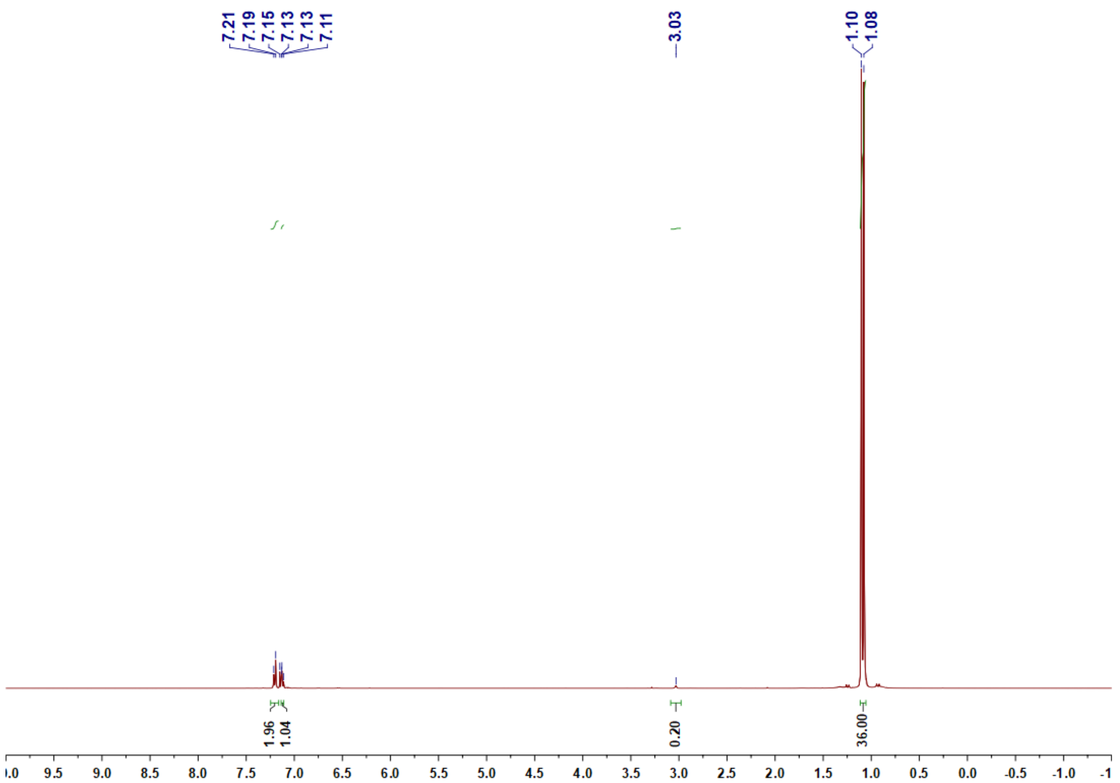

Figure S48. ^{1}H NMR spectrum after removing toluene and redissolving in C_6D_6

3.3 Reaction of Mg-2 with D_2

Reaction of **Mg-2** with D_2 (5 bar) at 120 °C for 3 days also afforded the free ligand with 94% of deuterium incorporated into the side arms. Next, the solvent was removed and then redissolved in benzene-d_6. It was confirmed by ^{1}H NMR that 94% of deuterium was incorporated into the side arm of the free ligand. This result suggests that **Mg-2** can also reversibly activate D_2 (H_2).

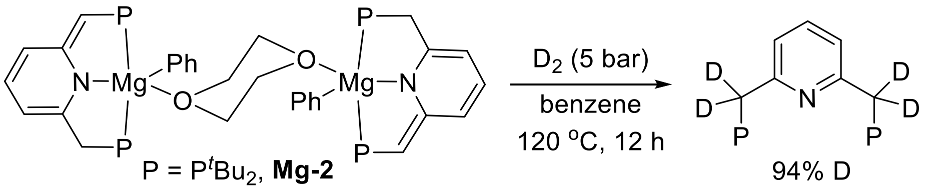

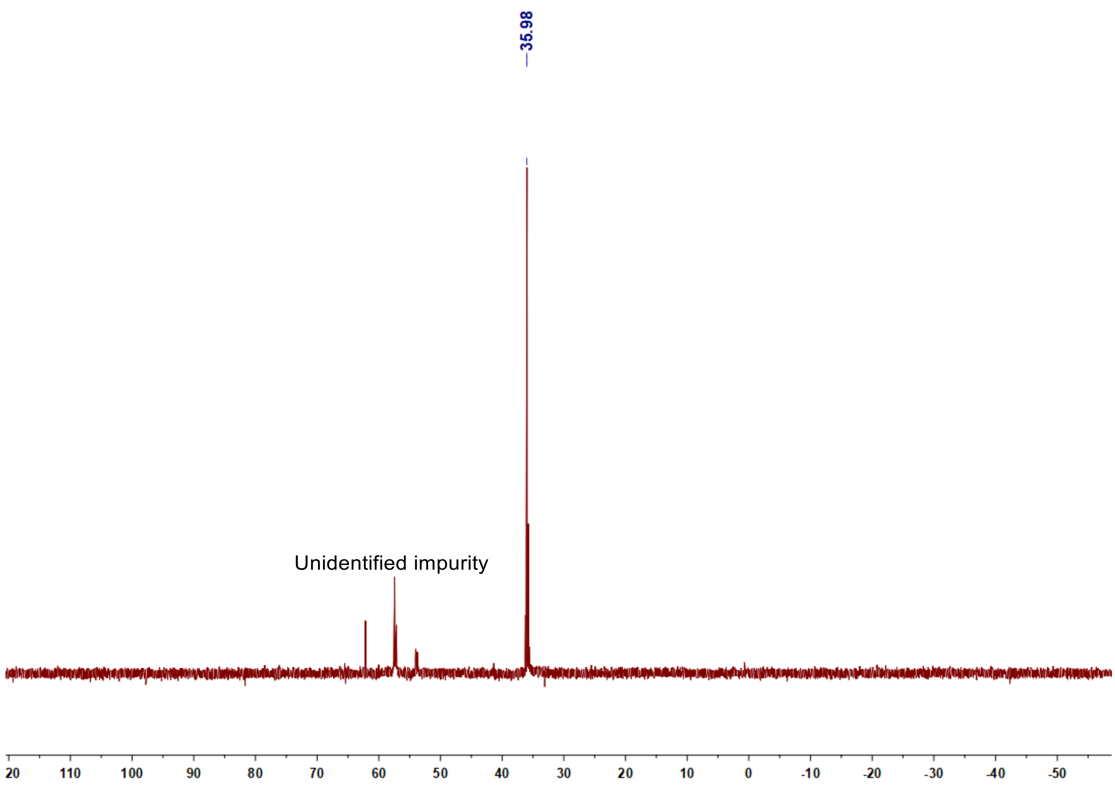

Figure S49. ^{31}P NMR spectrum of the reaction mixture in benzene after 3 days

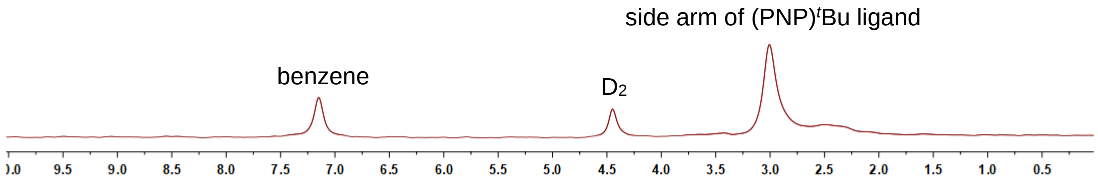

Figure S50. ^{2}H NMR spectrum of the reaction mixture in benzene after 3 days

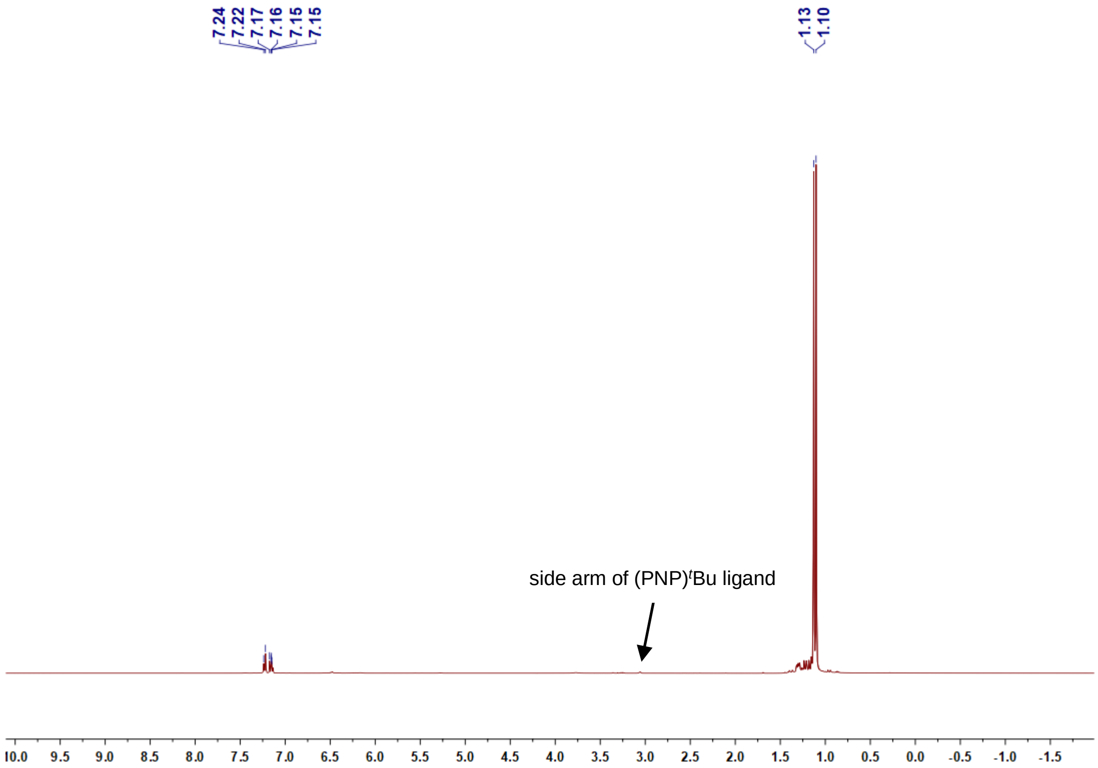

Figure S51. ^{1}H NMR spectrum after removing benzene and redissolving in benzene-d_6

3.4 Reaction of Mg-5 with D_2

To prove that **Mg-5** can reversibly activate H_2, a toluene solution of **Mg-5** was pressurized with D_2 (1 bar). After heating at 80 °C for 36 h, **Mg-5** was partially decomposed to the free ligand. The signals of the free ligand in the ^{31}P NMR spectrum split into multiplets, suggesting deuterium was incorporated into the side arm of the ligand (Figure S52). Deuterium incorporated into both side arms of **Mg-5** was also observed by ^{2}H NMR, confirming the reversible activation of D_2 (H_2) by **Mg-5** (Figure S53). Next, the solvent was removed, and the residue was dissolved in toluene-d_8. It was found that 70% of deuterium was already incorporated into both side arms at this stage, according to the integration of ^{1}H NMR (Figure S54). The result further confirms the reversible activation of D_2 (H_2) by **Mg-5**.

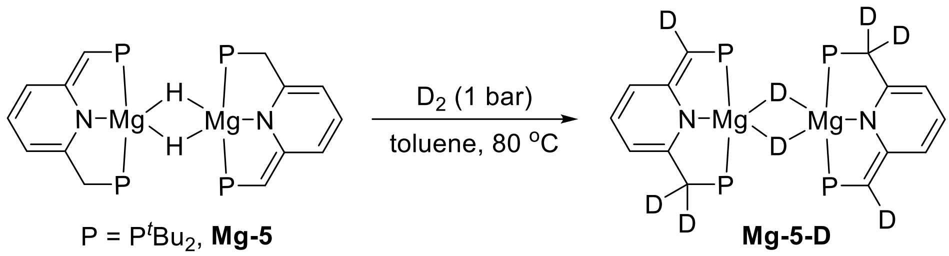

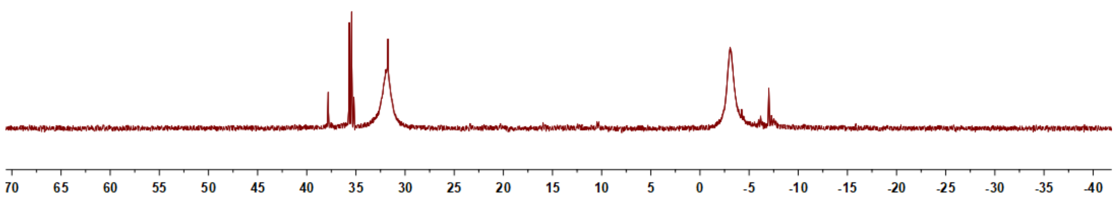

Figure S52. ^{31}P NMR spectrum of the reaction of **Mg-5** with D_2 in toluene

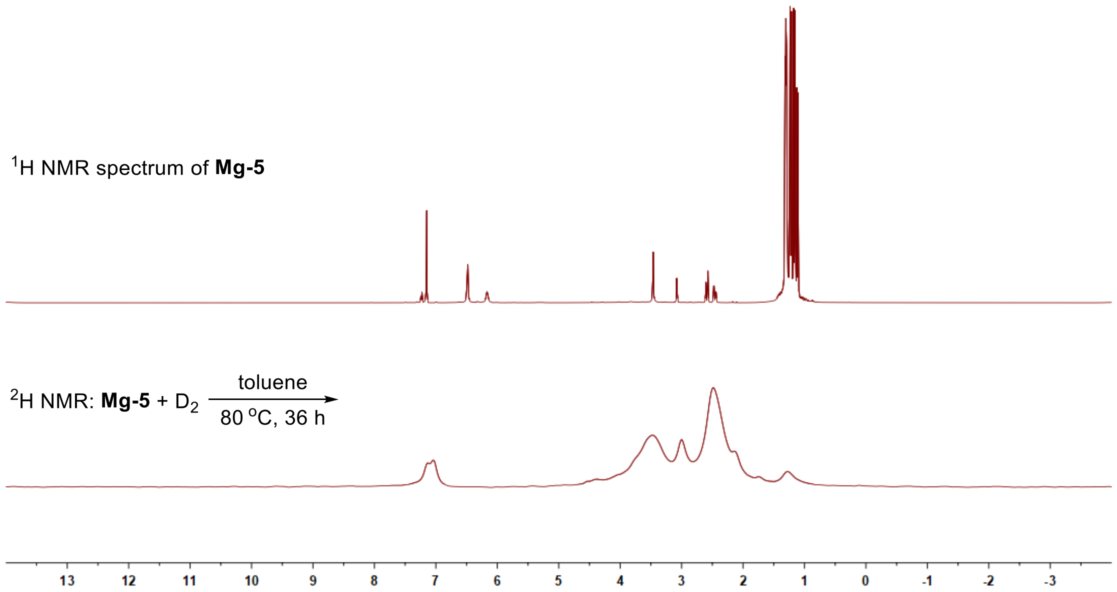

Figure S53. ^{2}H NMR spectrum of the reaction of **Mg-5** with D_2 in toluene

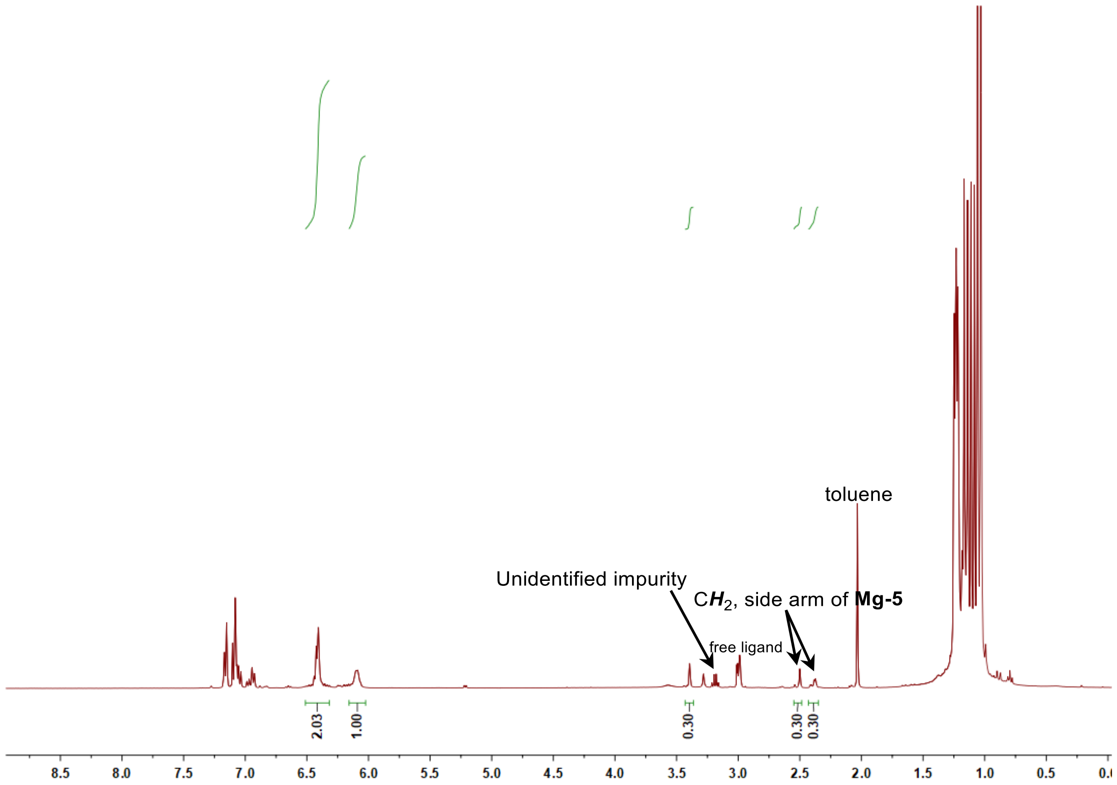

Figure S54. ^{1}H NMR spectrum of the reaction of **Mg-5** with D_2 in toluene-d_8

3.5 Reaction of (PNP)^{t}Bu free ligand with D_2

To further confirm deuterium incorporation proceeds via the metal-ligand corporation process, the free ligand (PNP)^{t}Bu was treated with D_2 (5 bar) at 80 °C in the absence of magnesium precursor. Incorporation of deuterium into the side arm was not observed.

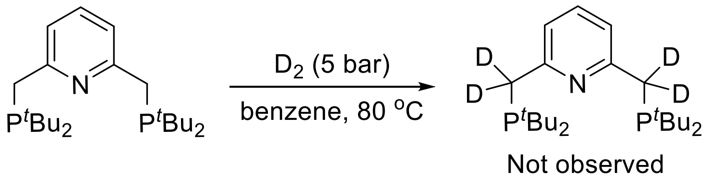

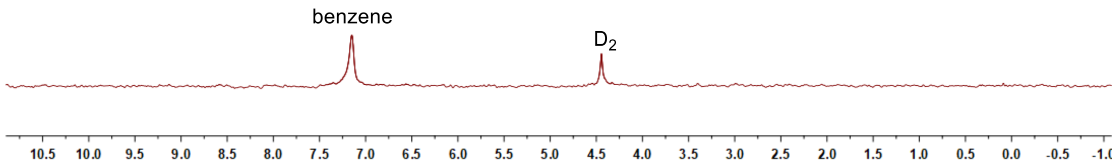

Figure S55. ^{2}H NMR spectrum of the reaction of (PNP)^{t}Bu free ligand with D_2

4 Catalytic semihydrogenation of alkynes

4.1 Conditions optimization

Table S10. Catalysts optimization for semihydrogenation of 1a

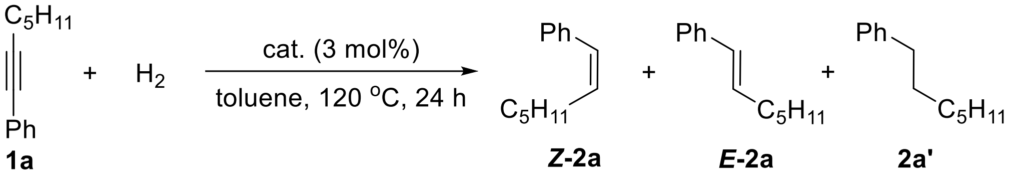

entry	cat.	yield of ***Z*-2a**/***E*-2a**/**2a′** (%)
1	**Mg-1**	95/3/<2
2	**Mg-2**	86/3/3
3	**Mg-3**	1/0/0
4	**Mg-4**	nr
5	**Mg-5**	95/3/2
6[a,b]	${}^{n}Bu_2Mg$/pinBH	nr
7[a]	${}^{n}Bu_2Mg$	nr
8[a]	Et_2Mg•dioxane	nr
9[a]	$MgBr_2$	nr

Reaction conditions: **1a** (0.3 mmol), cat. (3 mol%), H_2 (5 bar), toluene (0.5 mL), 120 °C, 24 h, reaction yields were determined by ^{1}H NMR using 1,3,5-trimethoxybenzene as the internal standard. [a]The loading of the catalyst is 6 mol%. [b]pinBH and ${}^{n}Bu_2Mg$ were firstly mixed together at room temperature in a 1:1 ratio. nr = no reaction.

Table S11. Conditions optimization for semihydrogenation of 1a

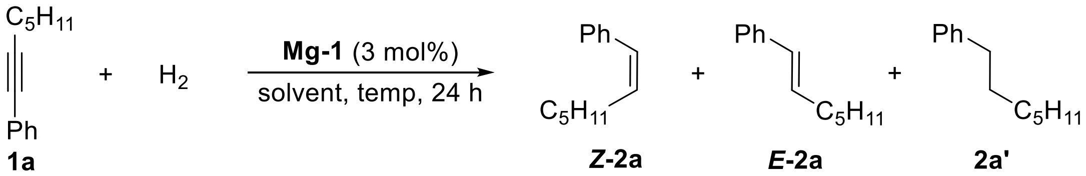

entry	solvent	H_2 (bar)	temp (°C)	yield (%) ***Z*-2a**/*E*-**2a**/**2a′**
1	toluene	5	120	95/3/<2
2	benzene	5	120	91/2/<2
3	xylene	5	120	95/3/2
4	THF	5	120	nr
5	dioxane	5	120	nr
6	toluene	3	120	60/2/nd
7	toluene	7	120	94/4/2
8	toluene	5	80	71/<2/<2

9	toluene	5	135	90/6/4

Reaction conditions: **1a** (0.3 mmol), **Mg-1** (3 mol%), solvent (0.5 mL), 24 h, reaction yields were determined by ^{1}H NMR using 1,3,5-trimethoxybenzene as the internal standard. nr = no reaction, nd = not detected.

4.2 Synthesis and characterization of alkyne substrates

Alkynes **1c**, **1i**, and **1l-1m** were commercially available. Alkynes **1a-1b**, **1d-1h**, **1j-1k**, and **1p-1x** were prepared according to the literature procedures.[10] **1o** was prepared as described below:

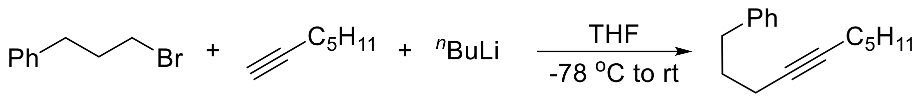

To a 50 mL Schlenk flask was added THF (20 mL) and 1-heptyne (480.9 mg, 5 mmol) under nitrogen atmosphere. The solution was cooled to -78 °C, and ^{n}BuLi (3.8 mL, 6 mmol, 1.6 M) was slowly dropped into the reaction. The resulting solution was stirred at the same temperature for 1 h. Next, a THF solution (5 mL) of 1-bromo-3-phenylpropane (995.5 mg, 5 mmol) was dropped into the reaction via a syringe over 10 min. The solution was gradually warmed to room temperature and stirred for 24 h. A saturated NH_4Cl solution was added to quench the reaction. The resulting mixture was extracted with Et_2O (30 mL × 3). The combined organic solvent was dried over Na_2SO_4, filtered, and concentrated by vacuum. The residue was purified by column chromatography using a flash silica gel column (eluent: hexane) to yield the alkyne **1o** as a colorless oil (706.8 mg, 66% yield).

^{1}H NMR (300 MHz, $CDCl_3$) δ 7.39 – 7.29 (m, 2H), 7.29 – 7.19 (m, 3H), 2.77 (t, J = 7.6 Hz, 2H), 2.22 (t, J = 6.4 Hz, 4H), 1.93 – 1.76 (m, 2H), 1.63 – 1.47 (m, 2H), 1.47 – 1.25 (m, 4H), 0.96 (t, J = 6.9 Hz, 3H).

^{13}C NMR (75 MHz, $CDCl_3$) δ 142.04, 128.66, 128.43, 125.92, 81.02, 79.78, 34.94, 31.24, 30.91, 29.01, 22.39, 18.88, 18.37, 14.17.

GC-MS m/z calcd. for $C_{15}H_{22}$ $[M]^+$: 214.2, found: 214.2.

4.3 Procedures of catalytic semihydrogenation of alkynes and characterization of alkene products

General procedures:

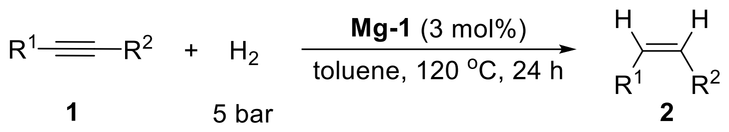

In a N_2 glovebox, alkyne **1** (0.3 mmol) was added to a 9 mL high-pressure tube and dissolved by toluene (0.2 mL). The catalyst was dissolved in a small vial with toluene (0.3 mL) and then transferred into the pressure tube by syringe. The tube was sealed, taken out of the glovebox, and charged with 5 bar of H_2. The resulting solution was heated at 120 °C for 24 h. The reaction was cooled to room temperature, and the gas was released. The solution was concentrated under reduced pressure. The resulting residue was transferred to the NMR tube to determine the reaction yield using 1,3,5-trimethoxybenzene as the internal standard. The unreported products were purified by column chromatography using a short flash silica gel column (eluent: hexane) to obtain the alkene products.

Procedure for the gram-scale reaction: In a N_2 glovebox, **1a** (1032.8 mg, 6 mmol), **Mg-1** (3 mol%), and toluene (6 mL) were added to a 8 mL small tube. The small tube was put into a 90 mL Fischer-Porter tube containing 5 mL toluene. The tubes were taken out of the glovebox and charged with 5 bar of H_2. The resulting solution was heated at 120 °C for 24 h. The reaction was cooled to room temperature, and the gas was released. The solution was concentrated under reduced pressure and the resulting residue was purified by column chromatography using a short flash silica gel column (eluent: hexane) to obtain the alkene products (92% yield, 25:1 Z/E, with 3% over-reduction product).

Characterization data of unreported products:

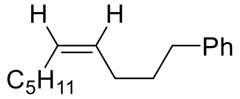

(*Z*)-Dec-4-en-1-ylbenzene (2o). Colorless oil, 60.3 mg, 93% isolated yield, and >99:1

Z/E.

^{1}H NMR (300 MHz, $CDCl_3$) δ 7.38 – 7.30 (m, 2H), 7.25 (d, *J* = 6.8 Hz, 3H), 5.46 (t, *J* = 5.2 Hz, 2H), 2.73 – 2.58 (m, 2H), 2.23 – 2.00 (m, 4H), 1.84 – 1.66 (m, 2H), 1.51 – 1.23 (m, 6H), 0.96 (t, *J* = 6.4 Hz, 3H).

^{13}C NMR (75 MHz, $CDCl_3$) δ 142.72, 130.65, 129.38, 128.56, 128.38, 125.76, 35.63, 31.67, 31.65, 29.56, 27.38, 26.96, 22.73, 14.24.

GC-MS *m/z* calcd. for $C_{16}H_{24}$ $[M]^+$: 216.2, found: 216.2.

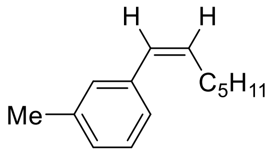

(*Z*)-1-(Hept-1-en-1-yl)-3-methylbenzene (2q). Colorless oil, 55.9 mg, 99% isolated yield, and 30:1 Z/E.

^{1}H NMR (400 MHz, $CDCl_3$) δ 7.36 (t, *J* = 7.8 Hz, 1H), 7.24 (d, *J* = 6.5 Hz, 2H), 7.18 (d, *J* = 7.5 Hz, 2H), 6.52 (d, *J* = 11.6 Hz, 1H), 5.79 (dt, *J* = 11.6, 7.3 Hz, 1H), 2.53 – 2.43 (m, 5H), 1.65 – 1.54 (m, 2H), 1.51 – 1.41 (m, 4H), 1.04 (t, *J* = 7.0 Hz, 3H).

^{13}C NMR (101 MHz, $CDCl_3$) δ 137.92, 137.72, 133.25, 129.67, 128.88, 128.12, 127.31, 125.93, 31.72, 29.84, 28.80, 22.71, 21.60, 14.20.

GC-MS *m/z* calcd. for $C_{14}H_{20}$ $[M]^+$: 188.2, found: 188.2.

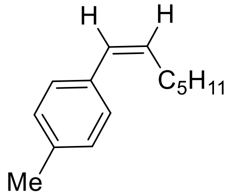

(*Z*)-1-(Hept-1-en-1-yl)-4-methylbenzene (2r). Colorless oil, 55.3 mg, 98% isolated yield, and 33:1 Z/E.

^{1}H NMR (300 MHz, $CDCl_3$) δ 7.32 – 7.16 (m, 4H), 6.44 (d, *J* = 11.6 Hz, 1H), 5.69 (dt, *J* = 11.6, 7.2 Hz, 1H), 2.48 – 2.32 (m, 5H), 1.59 – 1.46 (m, 2H), 1.43 – 1.32 (m, 4H), 0.96 (t, *J* = 6.8 Hz, 3H).

^{13}C NMR (75 MHz, $CDCl_3$) δ 136.17, 135.09, 132.72, 128.93, 128.80, 128.63, 31.74, 29.87, 28.82, 22.72, 21.29, 14.21.

GC-MS *m/z* calcd. for $C_{14}H_{20}$ $[M]^+$: 188.2, found: 188.1.

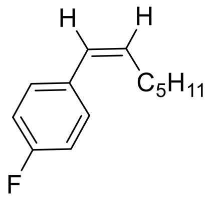

(*Z*)-1-Fluoro-4-(hept-1-en-1-yl)benzene (2s). Colorless oil, 56.5 mg, 98% isolated yield, and 30:1 Z/E.

^{1}H NMR (300 MHz, $CDCl_3$) δ 7.38 – 7.21 (m, 2H), 7.05 (t, *J* = 8.7 Hz, 2H), 6.39 (d, *J* = 11.6 Hz, 1H), 5.69 (dt, *J* = 11.6, 7.2 Hz, 1H), 2.40 – 2.23 (m, 2H), 1.57 – 1.42 (m, 2H), 1.40 – 1.29 (m, 4H), 0.93 (t, *J* = 6.2 Hz, 3H).

^{13}C NMR (75 MHz, $CDCl_3$) δ 161.54 (d, *J* = 245.7 Hz), 133.95 (d, *J* = 3.3 Hz), 133.26 (d, *J* = 1.1 Hz), 130.38 (d, *J* = 7.8 Hz), 127.70 (s), 115.07 (d, *J* = 21.3 Hz). 31.70, 29.76, 28.64, 22.69, 14.19.

^{19}F NMR (282 MHz, $CDCl_3$) δ -116.91.

GC-MS *m/z* calcd. for $C_{13}H_{27}F$ $[M]^+$: 192.1, found: 192.1.

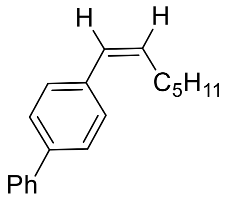

(*Z*)-4-(Hept-1-en-1-yl)-1,1'-biphenyl (2v). Colorless oil, 75.8 mg, 99% isolated yield, and 28:1 Z/E with 7% over-reduction product.

^{1}H NMR (400 MHz, $CDCl_3$) δ 7.81 – 7.74 (m, 4H), 7.61 (t, *J* = 7.6 Hz, 2H), 7.55 (d, *J* = 8.2 Hz, 2H), 7.51 (t, *J* = 7.3 Hz, 1H), 6.63 (d, *J* = 11.7 Hz, 1H), 5.89 (dt, *J* = 11.7, 7.3 Hz, 1H), 2.58 (qd, *J* = 7.5, 1.6 Hz, 2H), 1.72 – 1.63 (m, 2H), 1.56 – 1.50 (m, 4H), 1.10 (t, *J* = 7.0 Hz, 3H).

^{13}C NMR (101 MHz, $CDCl_3$) δ 141.00, 139.28, 137.02, 133.66, 129.33, 128.88, 128.40, 127.30, 127.09, 126.92, 31.74, 29.85, 28.94, 22.72, 14.21.

GC-MS *m/z* calcd. for $C_{19}H_{22}$ $[M]^+$: 250.2, found: 250.2.

5 Catalytic hydrogenation of alkenes

5.1 Conditions optimization for hydrogenation of styrene

Table S12. Optimization for hydrogenation of 3a

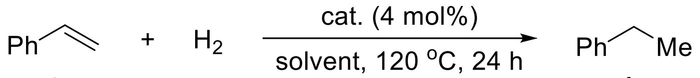

3a **4a**

entry	cat.	solvent	H_2 (bar)	yield (%)
1[a]	**Mg-1**	toluene	5	80
2	**Mg-1**	toluene	5	>99
3	**Mg-5**	toluene	5	>99
4	**Mg-1**	toluene	3	87
5	**Mg-1**	toluene	7	>99
6	**Mg-1**	benzene	5	96
7	**Mg-1**	xylene	5	>99
8	**Mg-1**	PhCl	5	89

Reaction conditions: **3a** (0.3 mmol), toluene (0.5 mL), 24 h, reaction yields were determined by ^{1}H NMR using benzyl benzoate as the internal standard. [a]With 3 mol% of **Mg-1**.

5.2 Procedures of catalytic hydrogenation of alkenes

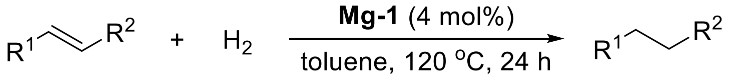

3 **4**

In a N_2 glovebox, alkene **3** (0.3 mmol) was added to a 9 mL high-pressure tube and dissolved by toluene (0.2 mL). The catalyst was dissolved in a small vial with toluene (0.3 mL) and then transferred into the pressure tube by syringe. The tube was sealed, taken out of the glovebox, and charged with 5 bar of H_2. The resulting solution was heated at 120 °C for 24 h. The reaction was cooled to room temperature, and the gas was released. The resulting residue was transferred to an NMR tube by $CDCl_3$ to determine the reaction yield using benzyl benzoate as the internal standard. The isolated products were purified by column chromatography using a short flash silica gel column (eluent: hexane).

6 Mechanistic studies

To investigate the active catalytic species and reaction intermediates, **Mg-1** was

dissolved in toluene and combined with 1-phenylpropyne **1c** in a 1:6 ratio in a J. Young NMR tube. The solution was pressurized with 3 bar of H_2 to monitor the reaction. Two new singlets gradually appeared at 24.89 and -3.51 ppm in the $^{31}P\{^1H\}$ NMR spectrum in a 1:1 ratio. The complex **Int-S$_1$** is proposed as a reaction intermediate. But the structure is unclear because of the overlapped signals in the ^1H NMR spectrum.

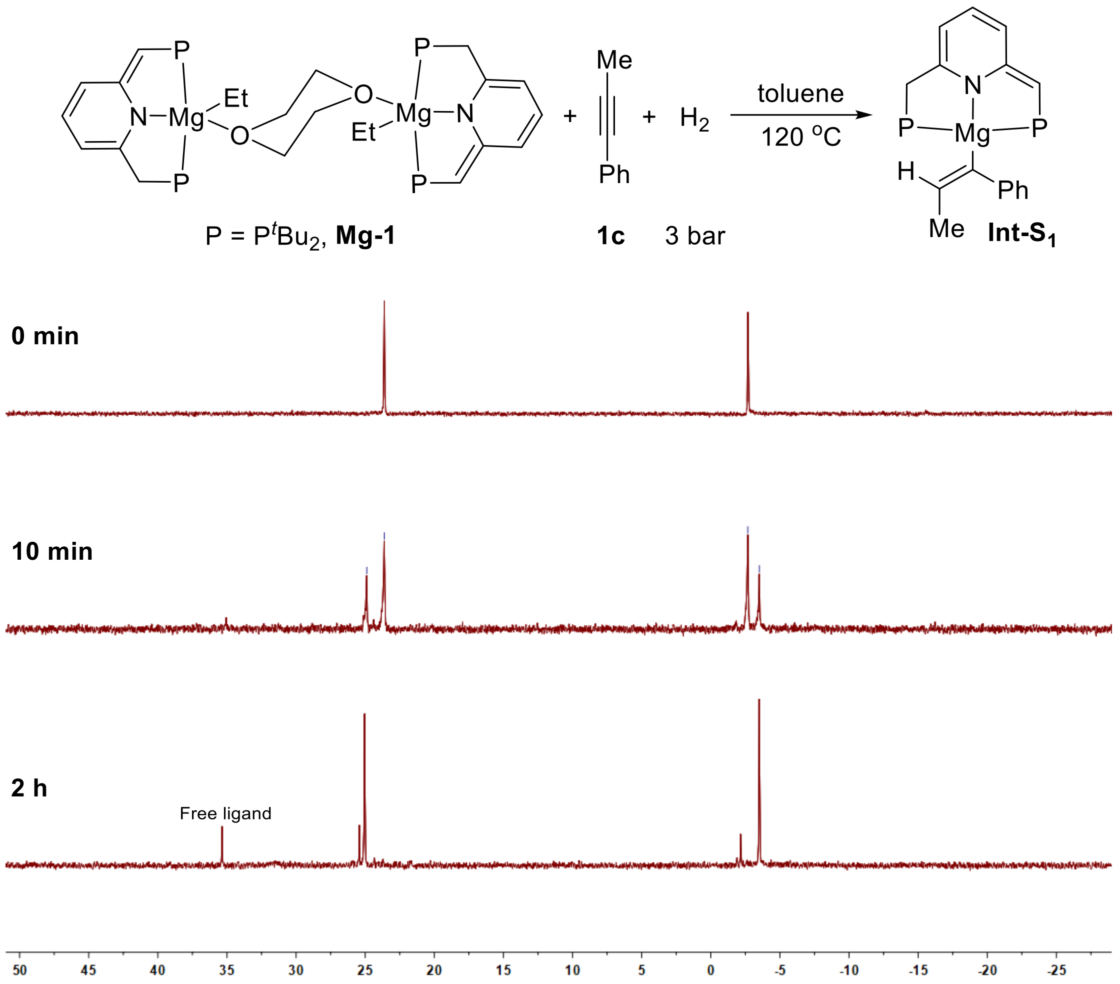

Figure S56. ^{31}P NMR spectrum of monitoring the formation of **Int-S$_1$**

To determine the structure of the intermediate, 6-dodecyne **1n** (0.16 mmol) and **Mg-1** (0.02 mmol) were dissolved by toluene-d_8 in a J. Young NMR tube. The solution was charged with 3 bar of H_2 and then heated at 120 °C. **Mg-1** gradually disappeared, and the reaction intermediate **Mg-6** was formed. The structure was

determined by NMR.

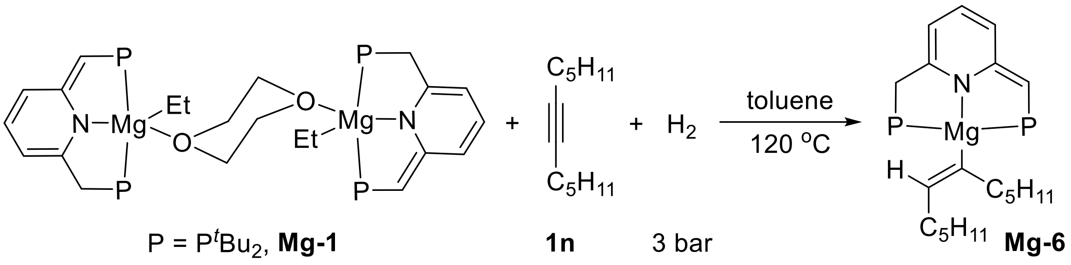

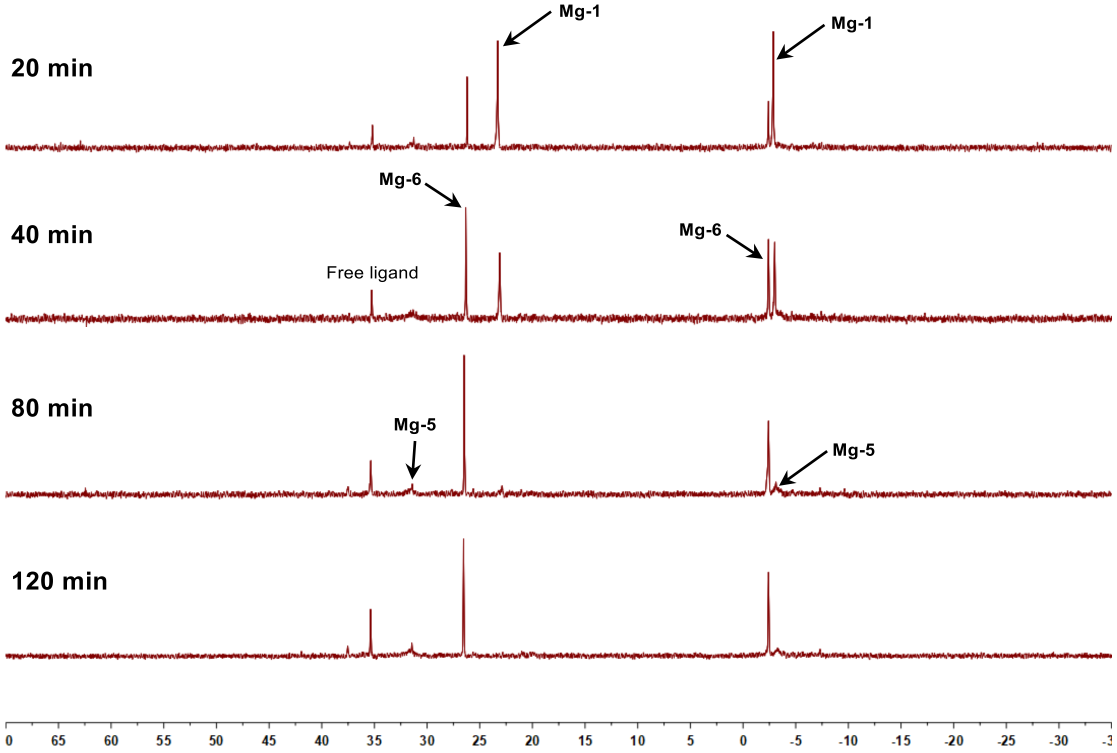

Figure S57. ^{31}P NMR spectrum of monitoring the formation of **Mg-6**

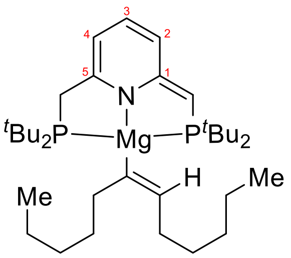

Characteristic NMR data of **Mg-6**:

^{1}H NMR (400 MHz, Tol-d_8) δ 6.41 (t, J = 8.9 Hz, 1H, Py$\boldsymbol{H}^3$), 6.17 (d, J = 8.9 Hz, 1H, Py$\boldsymbol{H}^2$), 5.87 (t, J = 6.2 Hz, 1H, MgCC$\boldsymbol{H}$), 5.48 (d, 1H, Py$\boldsymbol{H}^4$, overlapping with alkene

product), 3.55 (d, J = 4.9 Hz, 1H, PyC***H***P), 2.72 (s, 2H, PyC$\boldsymbol{H}_2$P), 2.60 (t, J = 10.0 Hz, 2H, MgCC$\boldsymbol{H}_2$), 2.35 – 2.30 (m, 2H, MgCCHC$\boldsymbol{H}_2$), 1.82 – 1.74 (m, 2H, $CH_2C\boldsymbol{H}_2CH_2$), 1.13 (d, J = 11.6 Hz, 18H, $PC(C\boldsymbol{H}_3)_3$), other aliphatic protons are overlapped with starting material and hydrogenation product.

^{13}C NMR (101 MHz, Tol-d_8) δ 172.7 (t, J = 23.0 Hz, Mg***C***CH), 168.67 (d, J = 16.8 Hz, Py***C***^{1}), 152.97 (s, Py***C***^{5}), 138.20 (s, MgC***C***H), 133.14 (s, Py***C***^{3}), 119.21 (d, J = 10.3 Hz, Py***C***^{2}), 102.33 (d, J = 6.5 Hz, Py***C***^{4}), 59.59 (d, J = 36.3 Hz, Py***C***HP), 35.86 (s, MgC$\boldsymbol{C}H_2$), 34.54 (s, $CH_2\boldsymbol{C}H_2CH_2$), 33.17 (d, J = 12.7 Hz, $P\boldsymbol{C}(CH_3)_3$), 32.27 (d, J = 8.9 Hz, $P\boldsymbol{C}(CH_3)_3$), 31.03 (d, J = 26.3 Hz, Py$\boldsymbol{C}H_2$P), 30.15 (d, J = 8.5 Hz, $PC(\boldsymbol{C}H_3)_3$), 29.95 (d, J = 8.3 Hz, $PC(\boldsymbol{C}H_3)_3$), 29.43 (s, MgCCH$\boldsymbol{C}H_2$), other aliphatic carbons are overlapped with starting material and hydrogenation product.

^{31}P NMR (162 MHz, Tol-d_8) δ 26.76 (s, $PyCH_2\boldsymbol{P}$), -2.42 (s, PyCH***P***).

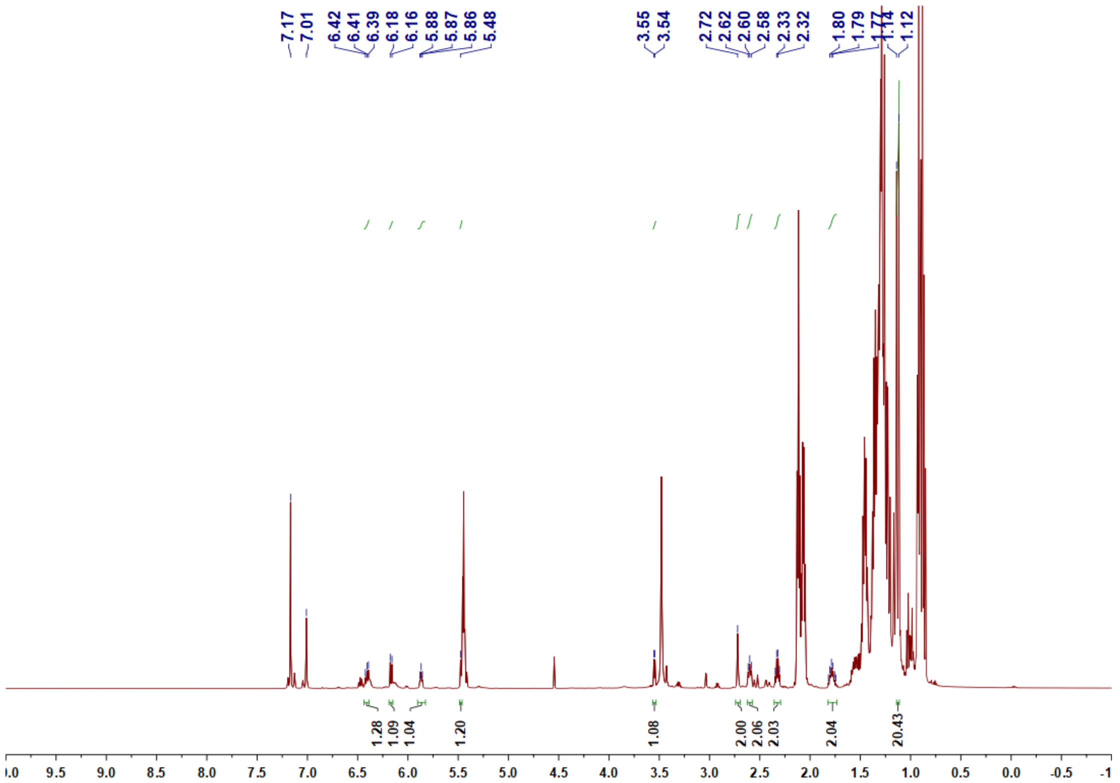

Figure S58. ^{1}H NMR spectrum of **Mg-6** in toluene-d_8

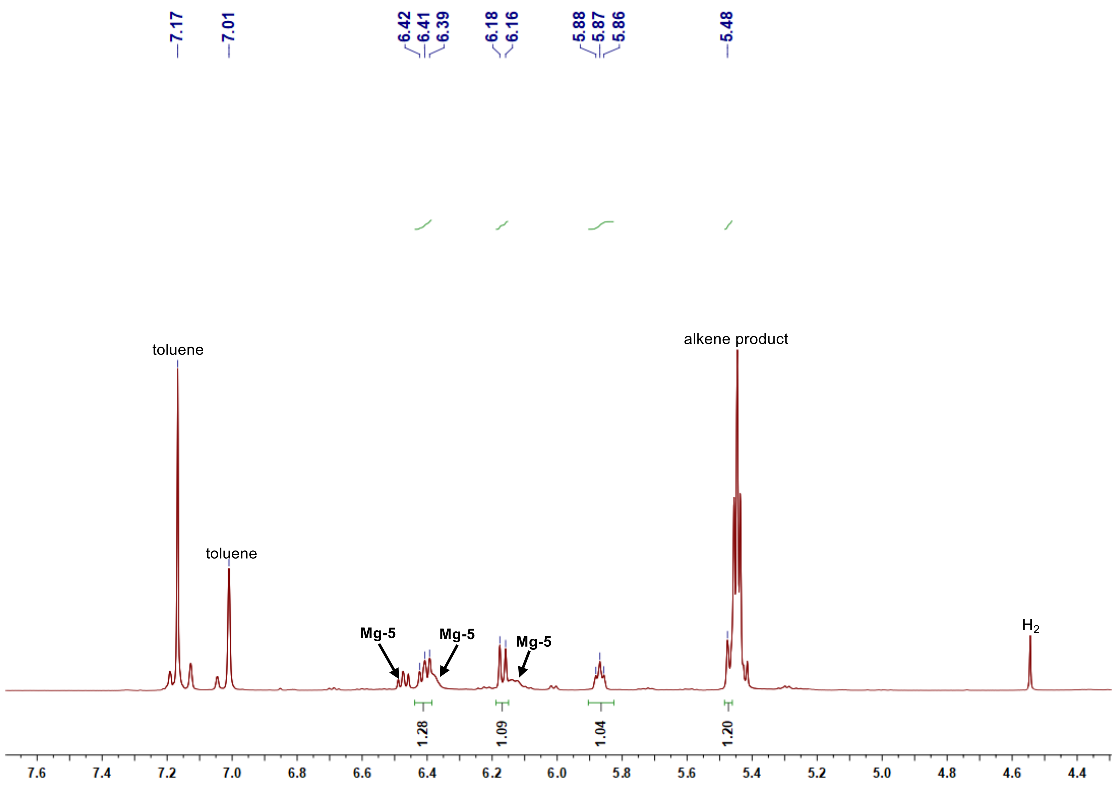

Figure S59. ^{1}H NMR spectrum (zoom in 1) of **Mg-6** in toluene-d_8

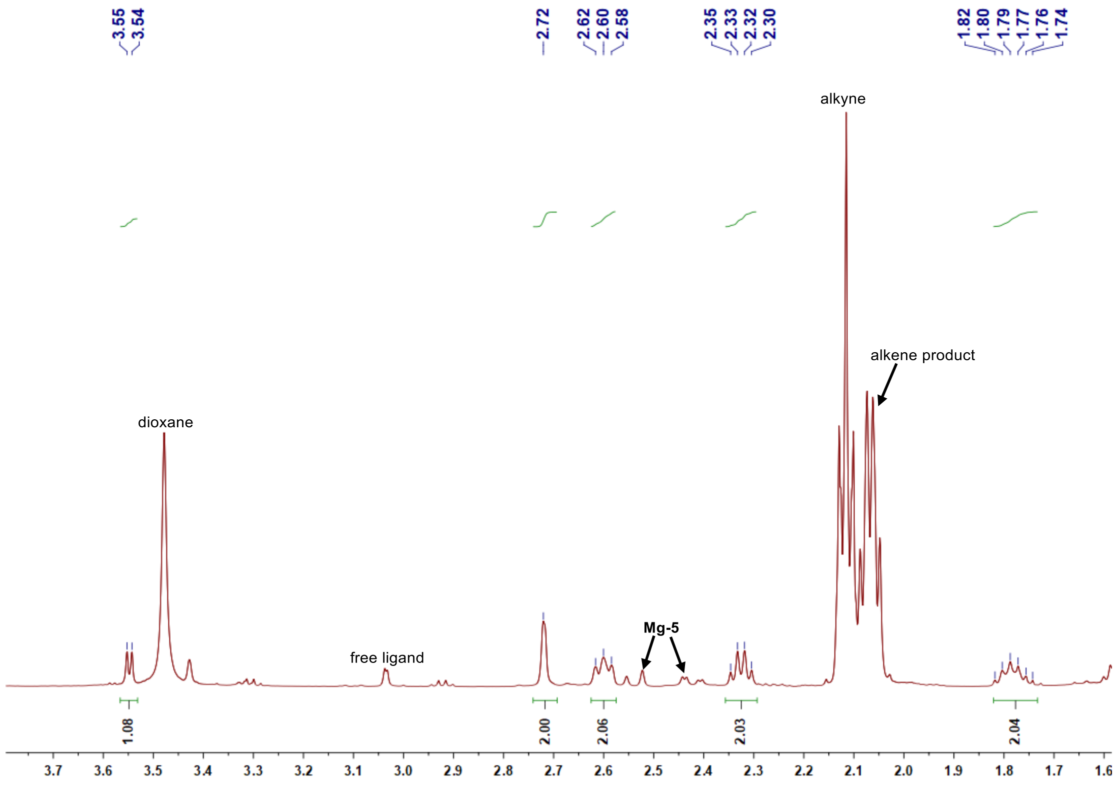

Figure S60. ^{1}H NMR spectrum (zoom in 2) of **Mg-6** in toluene-d_8

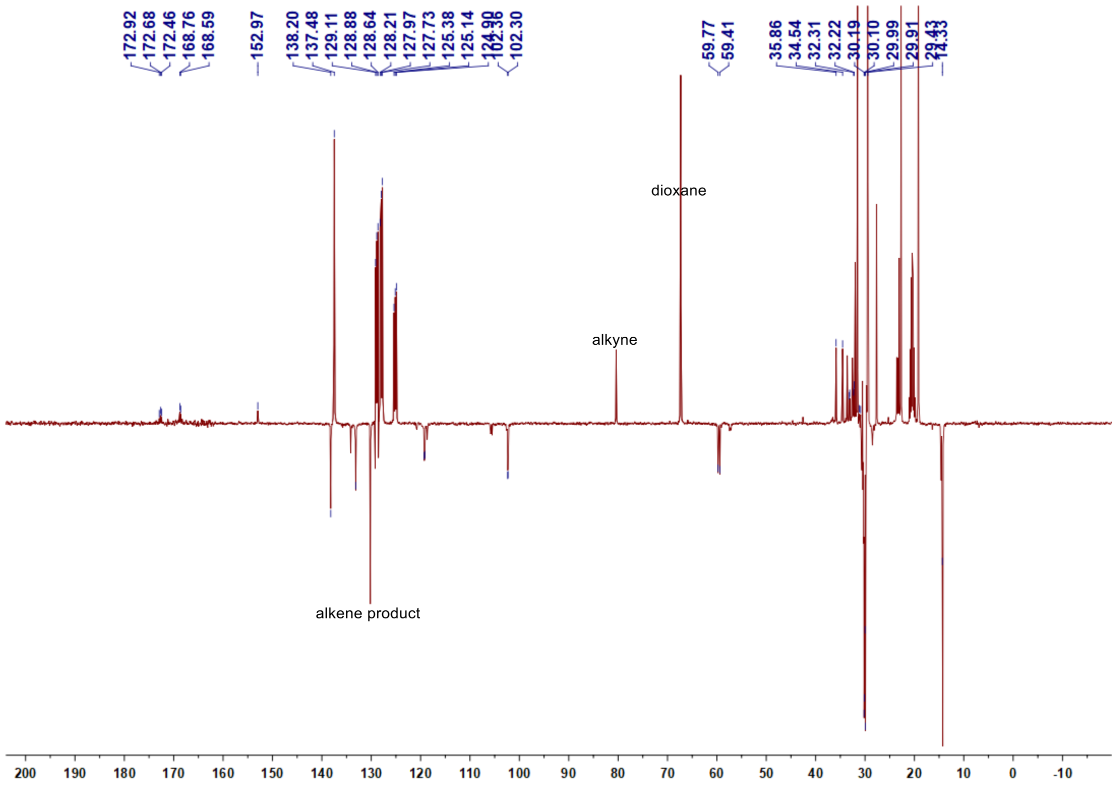

Figure S61. ^{13}C-DEPTQ NMR spectrum of **Mg-6** in toluene-d_8

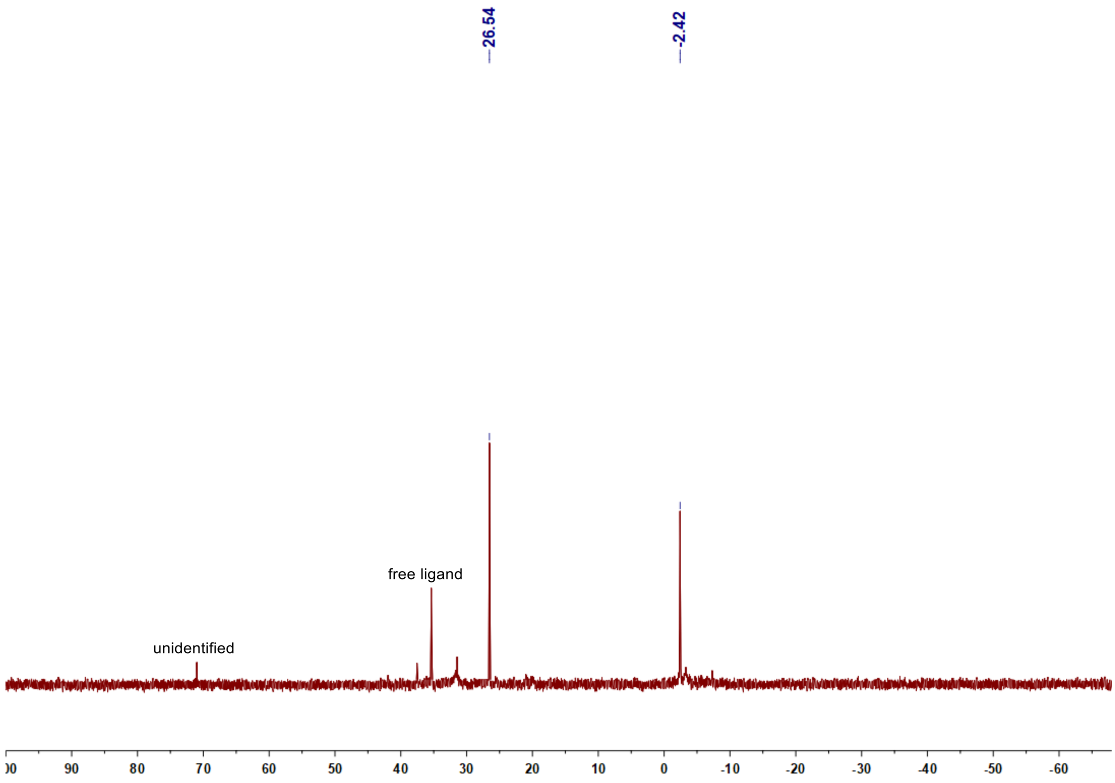

Figure S62. ^{31}P NMR spectrum of **Mg-6** in toluene-d_8

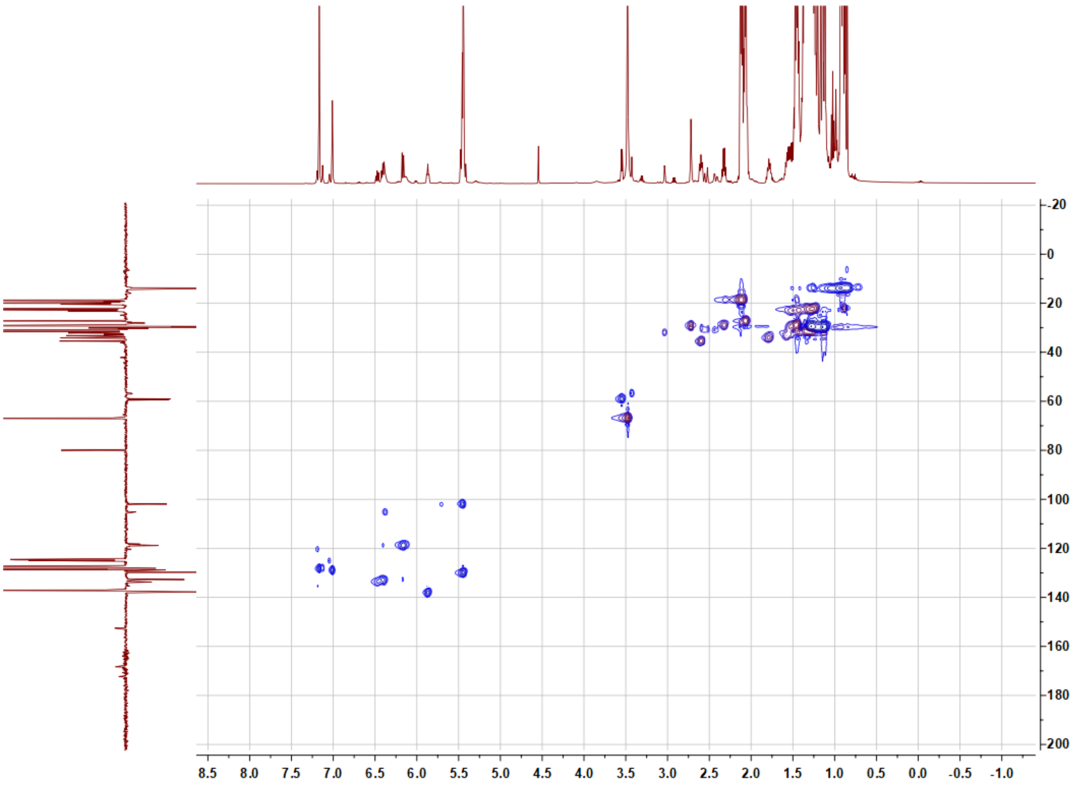

Figure S63. HSQC spectrum of **Mg-6** in toluene-d_8

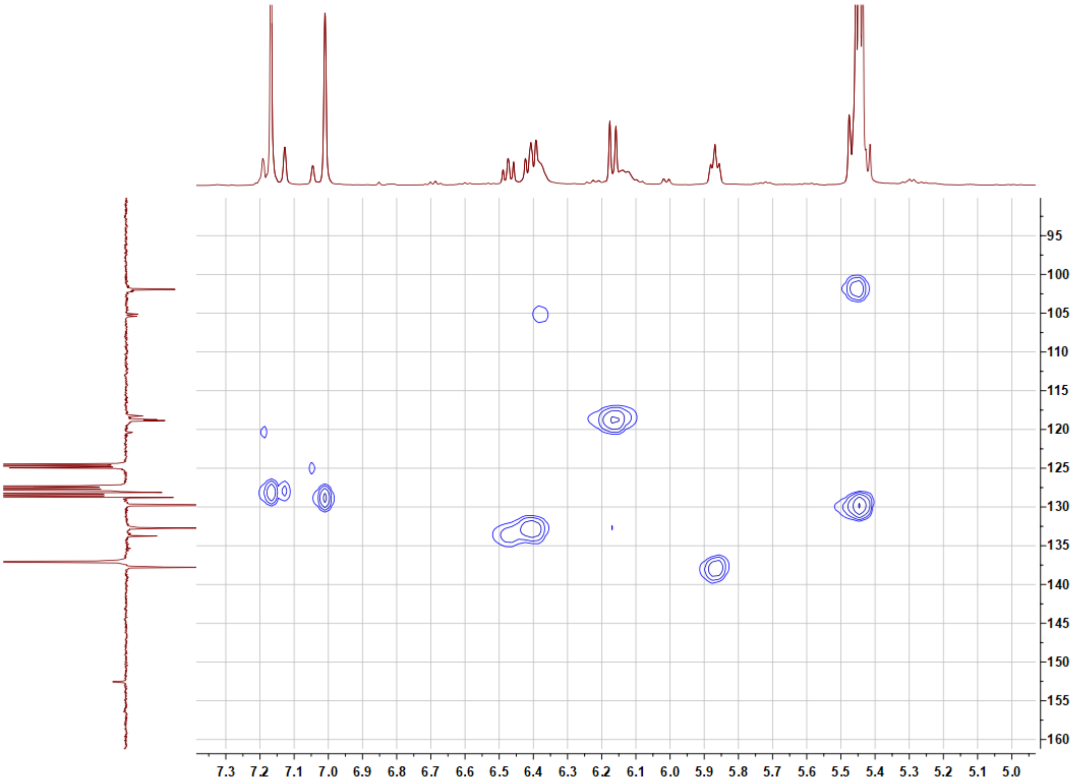

Figure S64. HSQC spectrum (zoom in 1) of **Mg-6** in toluene-d_8

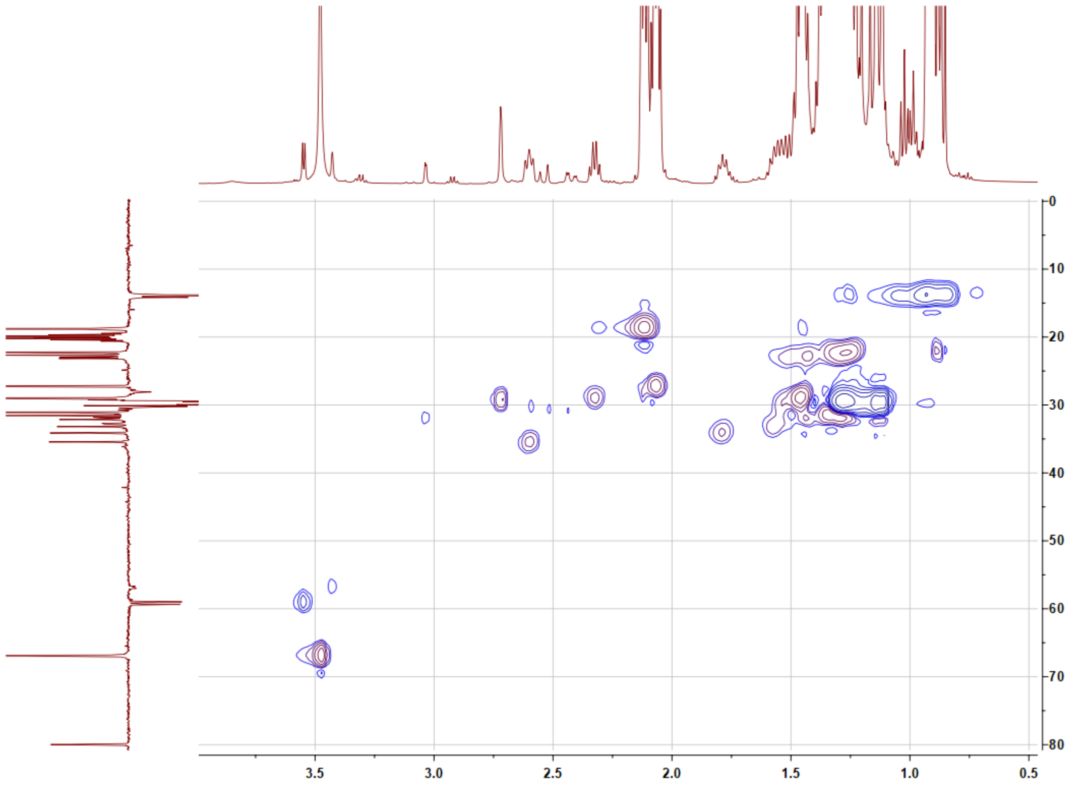

Figure S65. HSQC spectrum (zoom in 2) of **Mg-6** in toluene-d_8

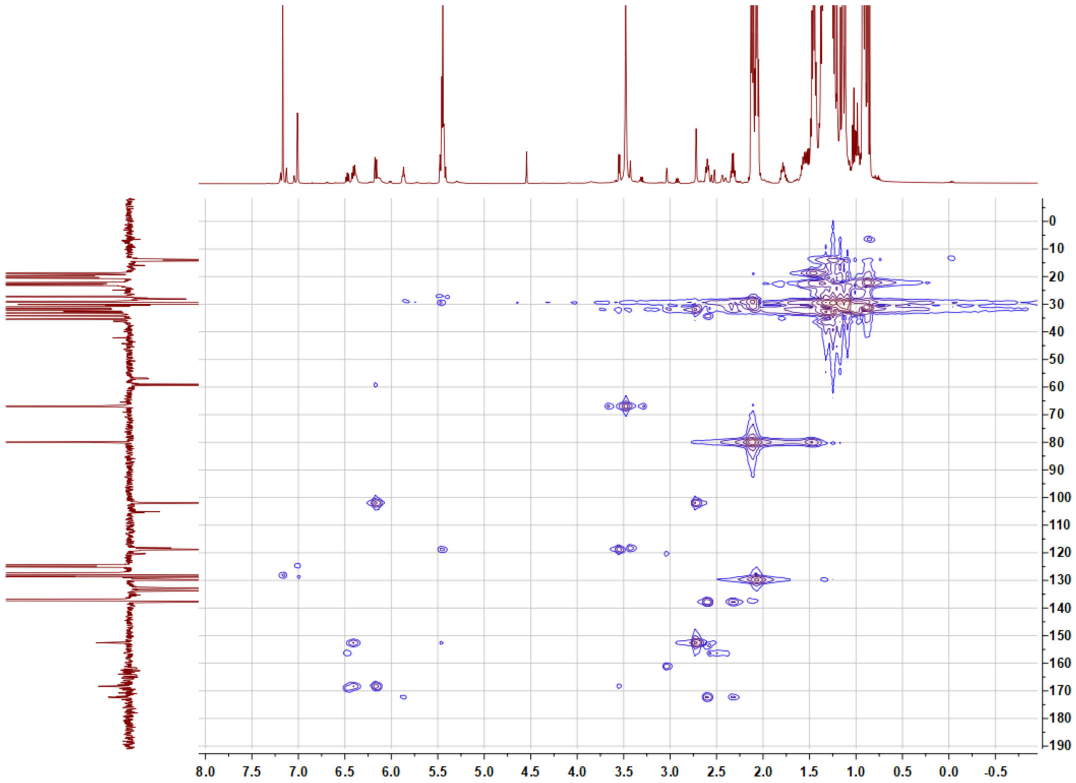

Figure S66. HMBC spectrum of **Mg-6** in toluene-d_8

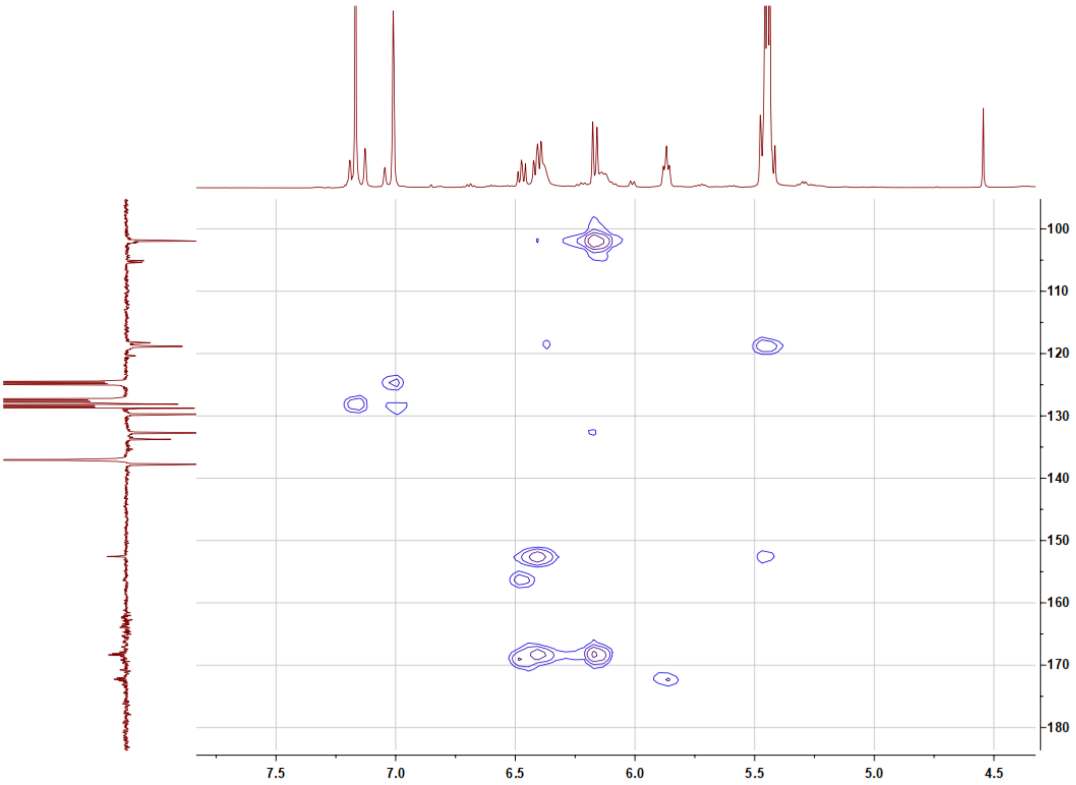

Figure S67. HMBC spectrum (zoom in 1) of **Mg-6** in toluene-d_8

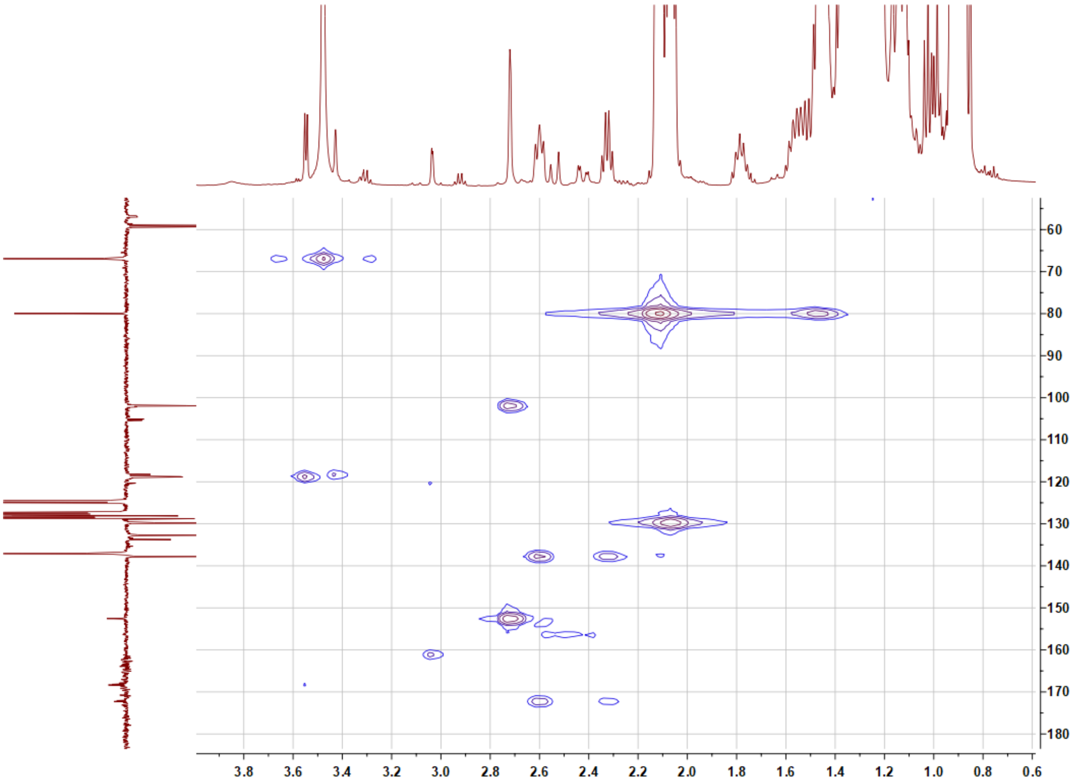

Figure S68. HMBC spectrum (zoom in 2) of **Mg-6** in toluene-d_8

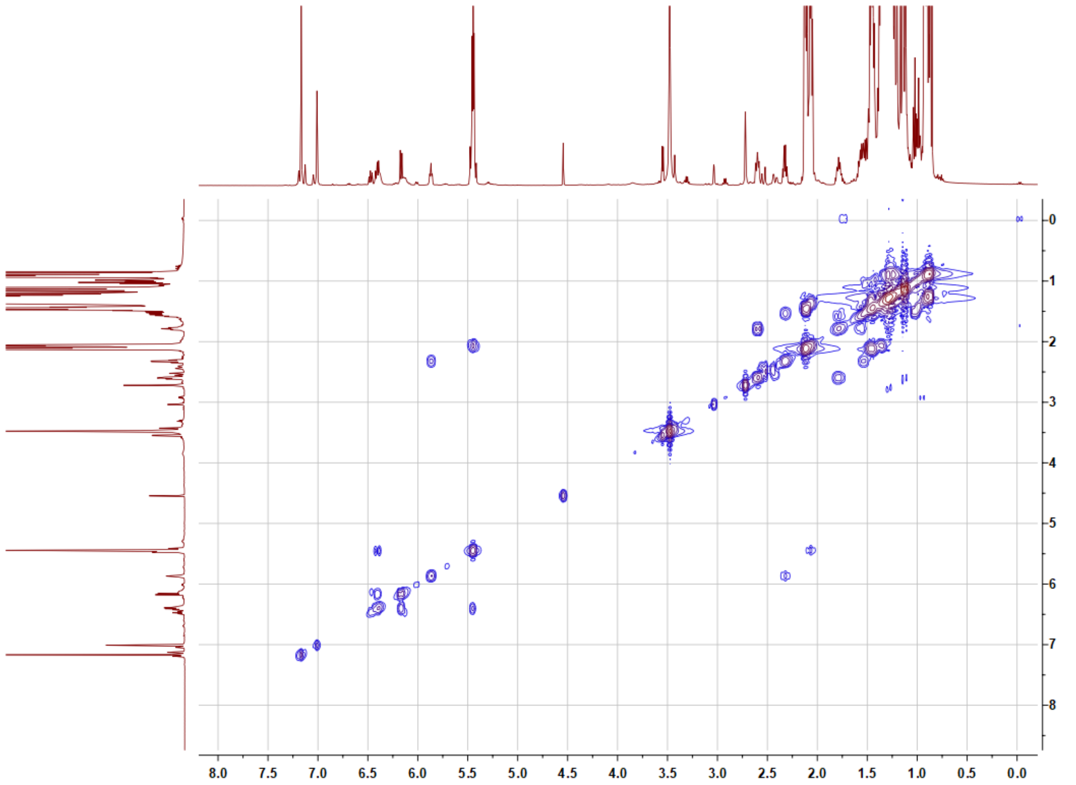

Figure S69. H-H COSY spectrum of **Mg-6** in toluene-d_8

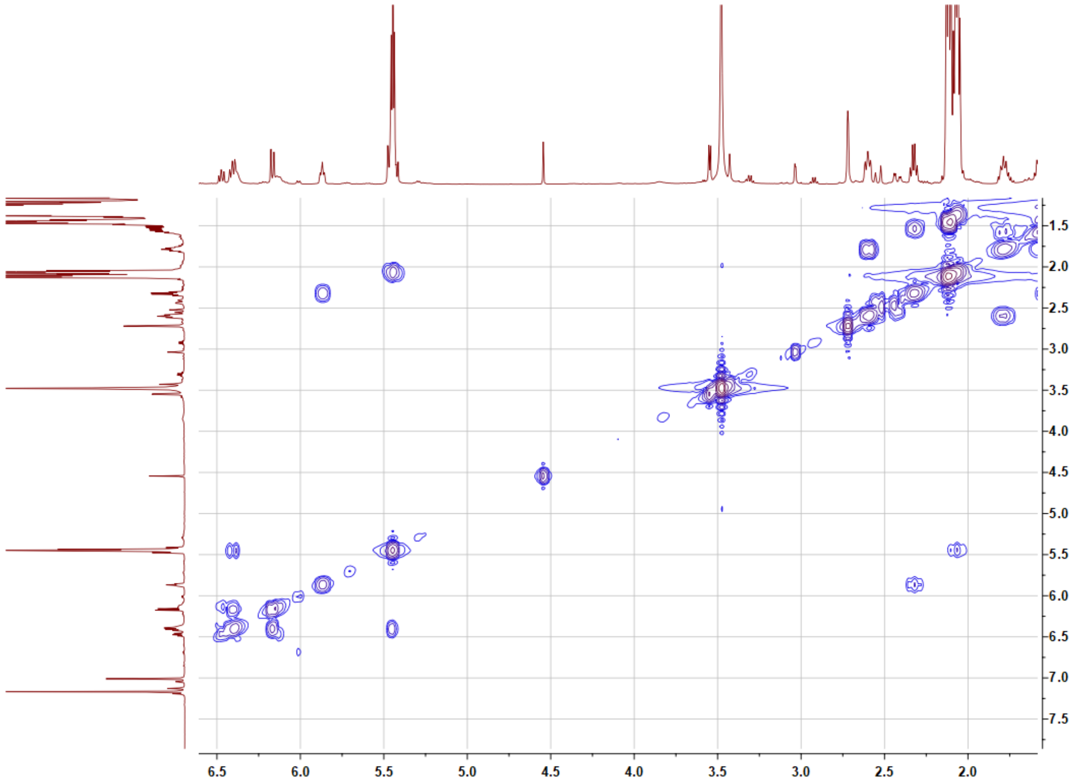

Figure S70. H-H COSY spectrum (zoom in) of **Mg-6** in toluene-d_8

Mg-5 catalyzed semihydrogenation of 6-dodecyne **1n** was also monitored under similar conditions. **1n** (0.12 mmol) and **Mg-5** (0.02 mmol) were dissolved by toluene-d_8 in a J. Young NMR tube and charged with 3 bar of H_2. The solution was heated at 120 °C, **Mg-5** gradually disappeared, and the reaction intermediate **Mg-6** was formed. As the consumption of the alkyne, **Mg-5** gradually regenerated. These results further confirm that **Mg-5** is the active catalyst.

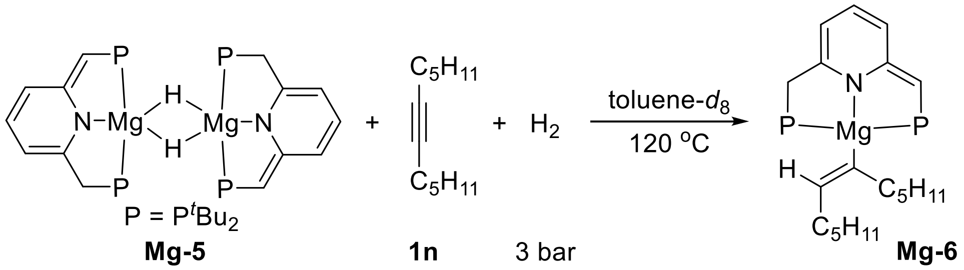

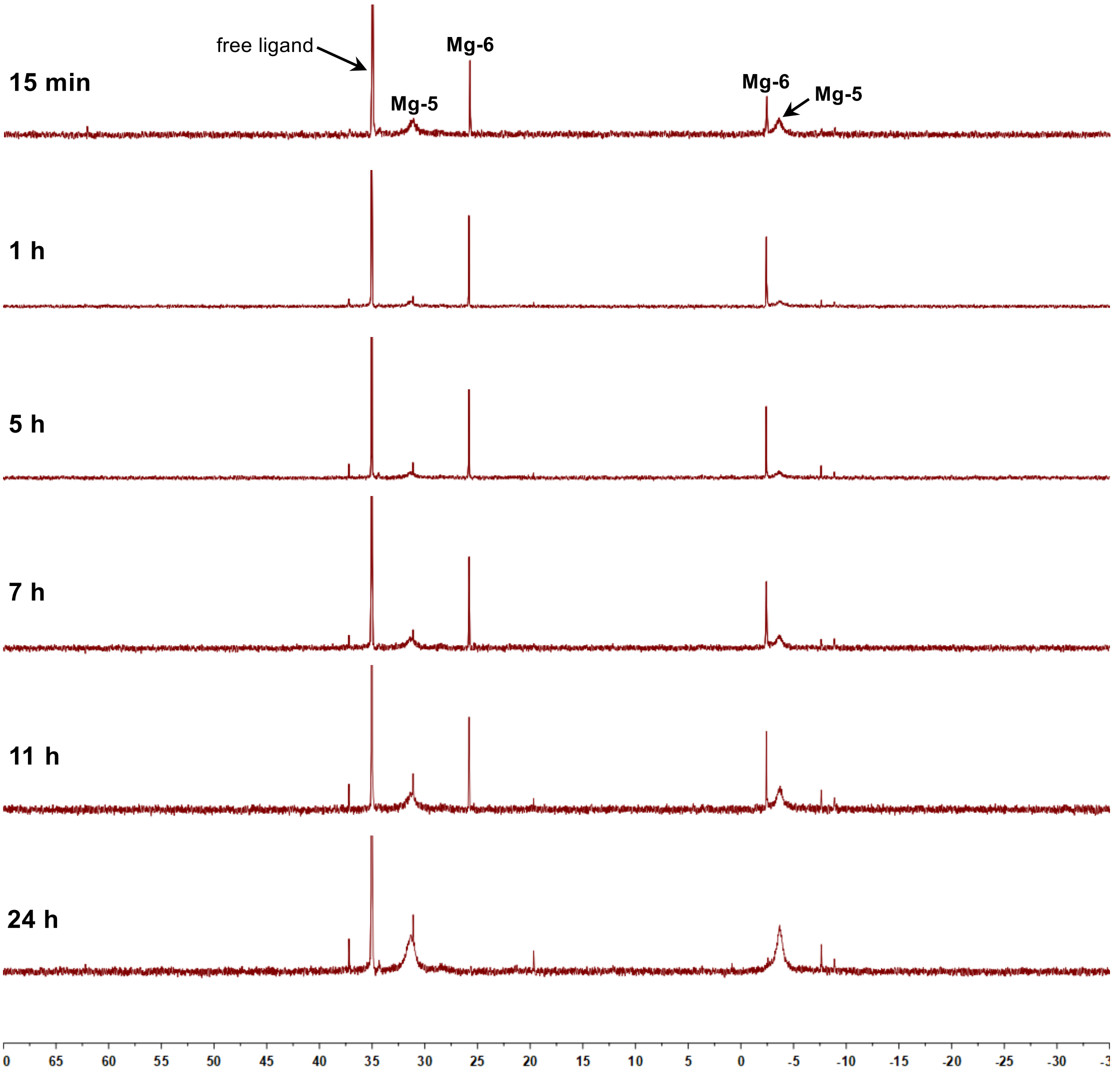

Figure S71. ^{31}P NMR spectrum of monitor experiment of **Mg-5** catalyzed semihydrogenation

Hydrogenation of styrene catalyzed by **Mg-1** was also monitored. Styrene **3a** (0.2 mmol) and **Mg-1** (0.02 mmol) were dissolved by toluene-d_8 in a J. Young NMR tube. The solution was charged with 3 bar of H_2 and then heated at 120 °C. **Mg-1** gradually disappeared, and the reaction intermediate **Int-S2** was formed. As the consumption of the styrene, **Mg-5** was gradually generated.

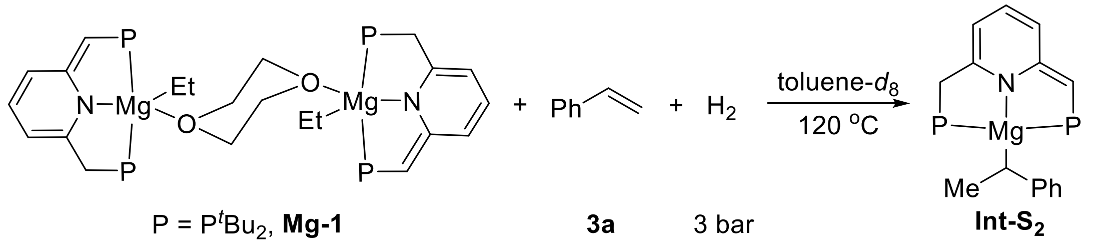

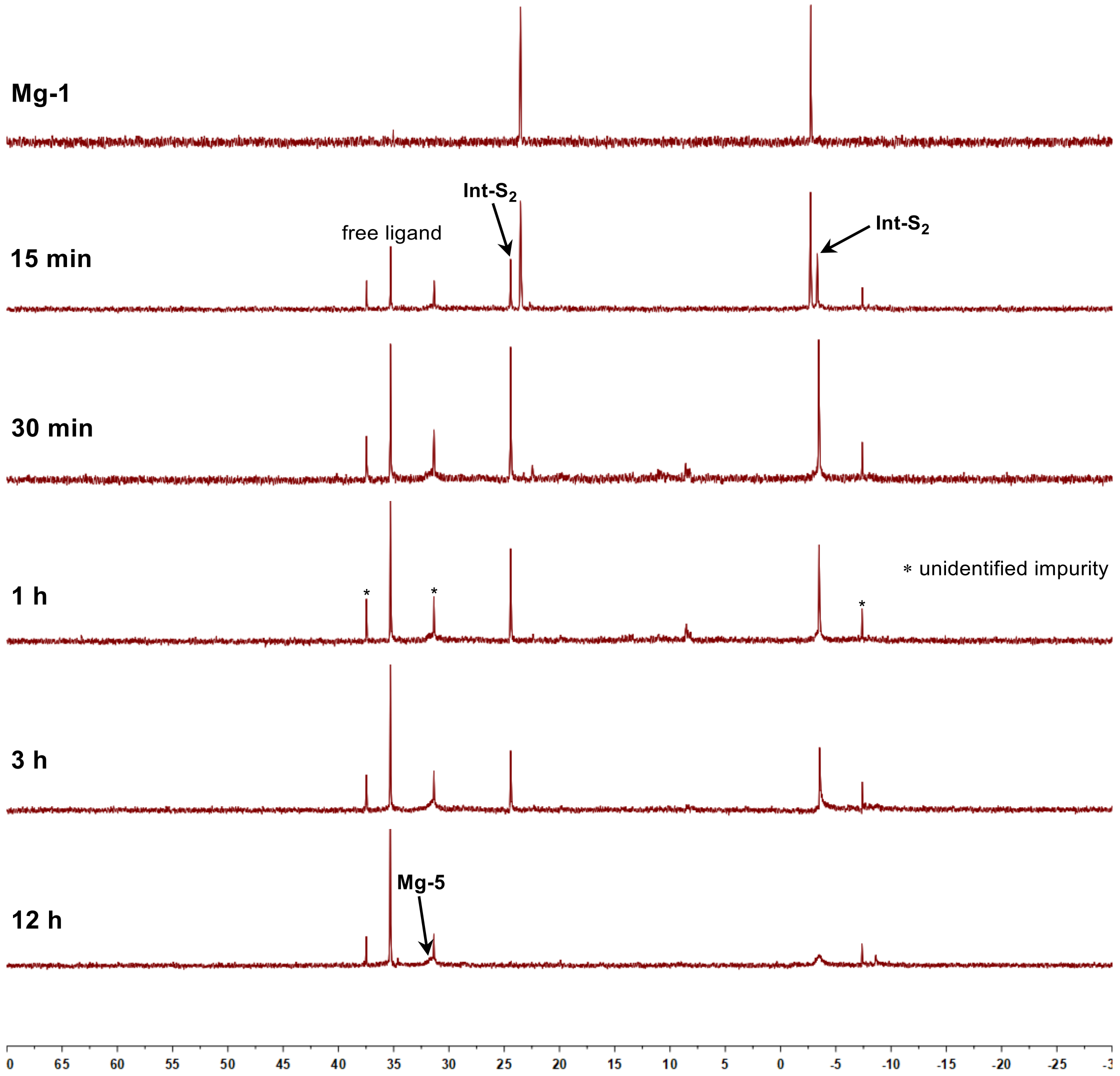

Figure S72. ^{31}P NMR spectrum of gradually formation of **Int-S2**

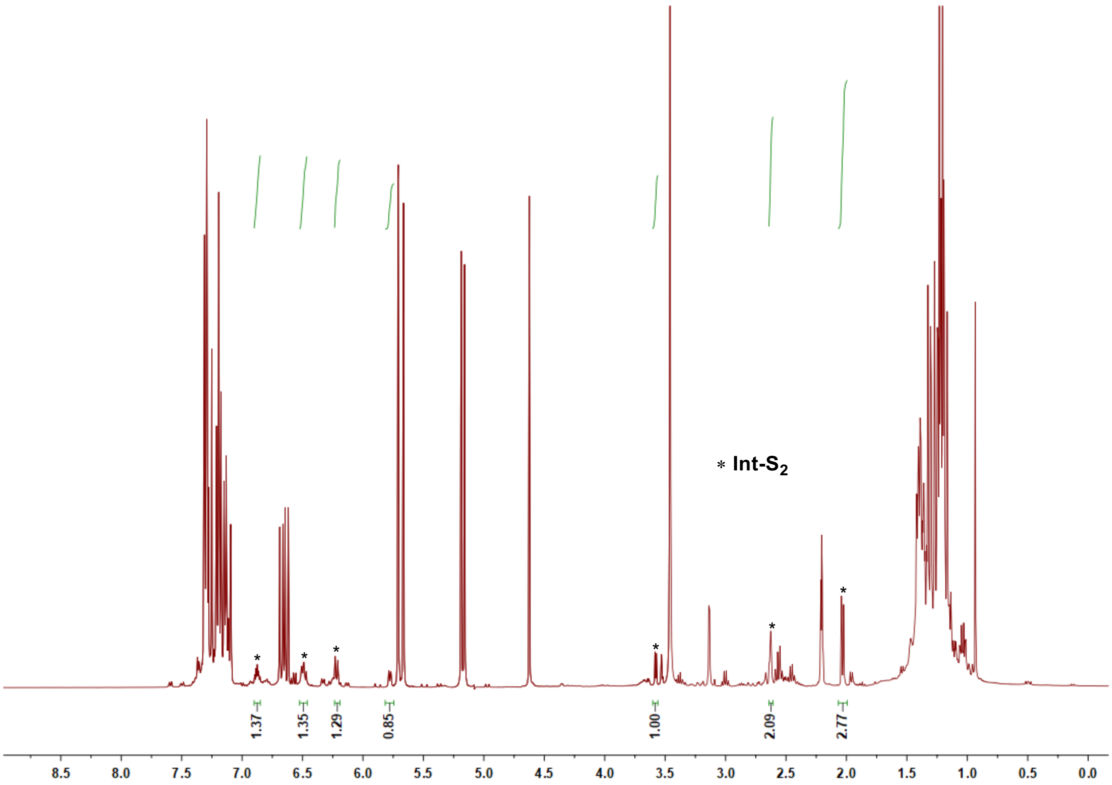

Figure S73. ^{1}H NMR of the solution at 30 min

Next, the reaction time for the hydrogenation of **1c** was prolonged to 48 h. It was found that the yield of *E*-alkene and alkane increased to 9% and 17%, respectively. The result suggests that **Mg-1** can slowly catalyze the isomerization of *Z*-alkenes to more stable *E*-alkenes and hydrogenation of internal alkenes to alkanes. However, the gradual decomposition of **Mg-1** to the free ligand led to the catalyst deactivation. Therefore, in most cases of the semihydrogenation of alkynes, the *E*-alkenes and over-reduction products could be avoided if the reactions were terminated after 24 h.

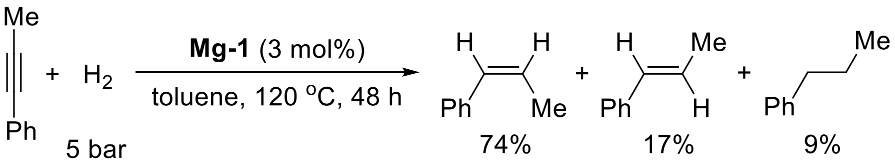

7 Computational details

DFT calculations were performed with Gaussian 16 (C.01 revision)[11] using Truhlar's M06-L functional,[12] the triple-ξ def2-TZVP basis set,[13] W06 density

fitting,[14] and Grimme's D3(0) empirical dispersion correction.[15] Frequency calculations at this level of theory were run to confirm stationary points and transition states, as well as to obtain thermodynamic corrections. Single point energies of the M06-L optimized structures were computed with ORCA (4.2.1)[16] using the range-separated meta-GGA hybrid functional ωB97M-V of the Head-Gordon group[17] including dispersion correction,[18,19] together with the triple-ξ def2-TZVPP basis set[13] and the corresponding auxiliary basis sets, def2/J[14] and def2-TZVPP/C[20] for RIJCOSX density fitting. The functional and basis set selections are based on recent benchmark studies.[21] The polarizable continuum model (IEFPCM) was used in all calculations (optimization and single point) with the SMD solvation (toluene) model of Truhlar and co-workers.[22] Gibbs free energies were computed by adding the free energy correction term from the frequency calculation to the single point energy in toluene, according to

$$\mathbf{G^{o\omega B97M\text{-}V}_{(toluene)} = E^{\omega B97M\text{-}V}_{toluene} + corr^{\mathit{M06\text{-}L}}_{\mathit{freq}(toluene)}}$$

where $\mathbf{E^{\omega B97M\text{-}V}_{Toluene}}$ is the single point energy, and where $\mathbf{corr^{\mathit{M06\text{-}L}}_{\mathit{freq}}}$ is the thermal correction to the Gibbs free energy from the frequency calculation.

Free energy values (G^o) were then corrected to account for changes in standard states ($G^o \rightarrow G$).

Standard state corrections[23] were employed such that all species are treated as 1M (using an ideal gas approximation), with the exception of H_2 (maintained as 5 atm).[24-26] Other than these standard state corrections, the transformation of hydrogen from the condensed phase to the gas phase is not additionally corrected in the free energy quantities provided.

2-Butyne was studied as a minimal model for alkynes in the system. Directionality of ΔG and ΔG_{TS} values are indicated by the ordering of X,Y, and all energies are reported in kcal/mol.

Several pathways for the semihydrogenation of alkynes are presented as follow:

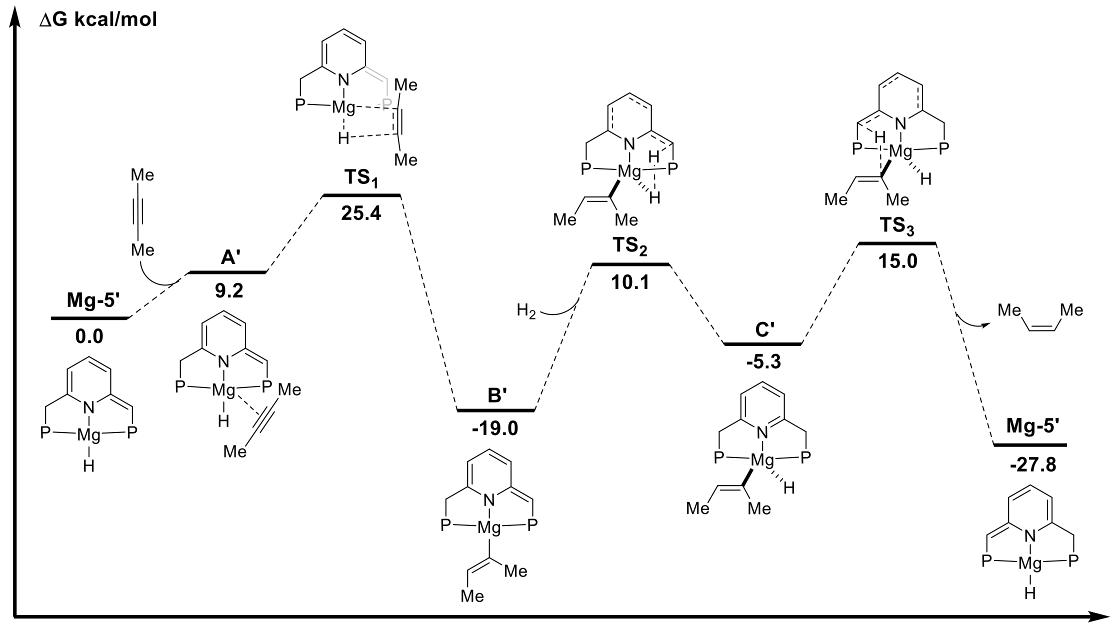

Figure S74. Pathway via the MLC process without opening the side arm

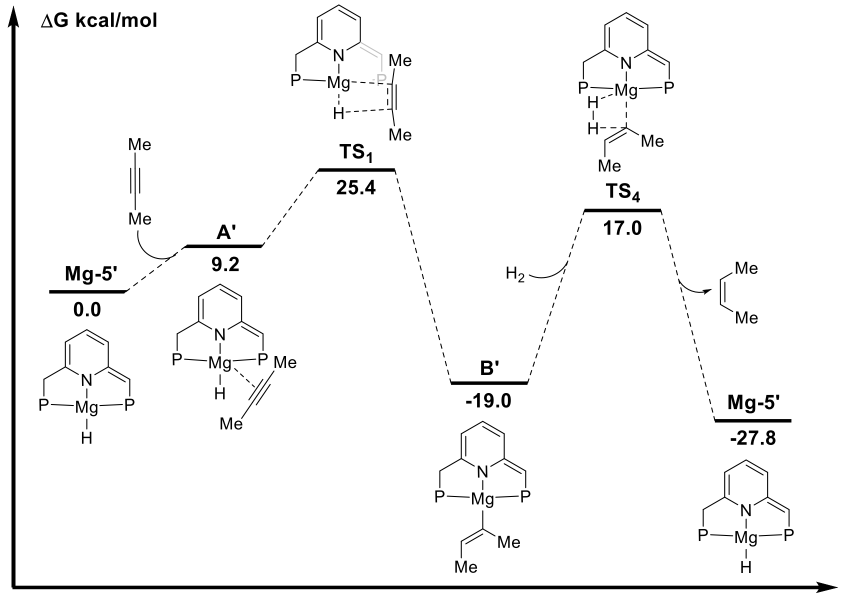

Figure S75. Pathway via the sigma bond metathesis without opening the side arm

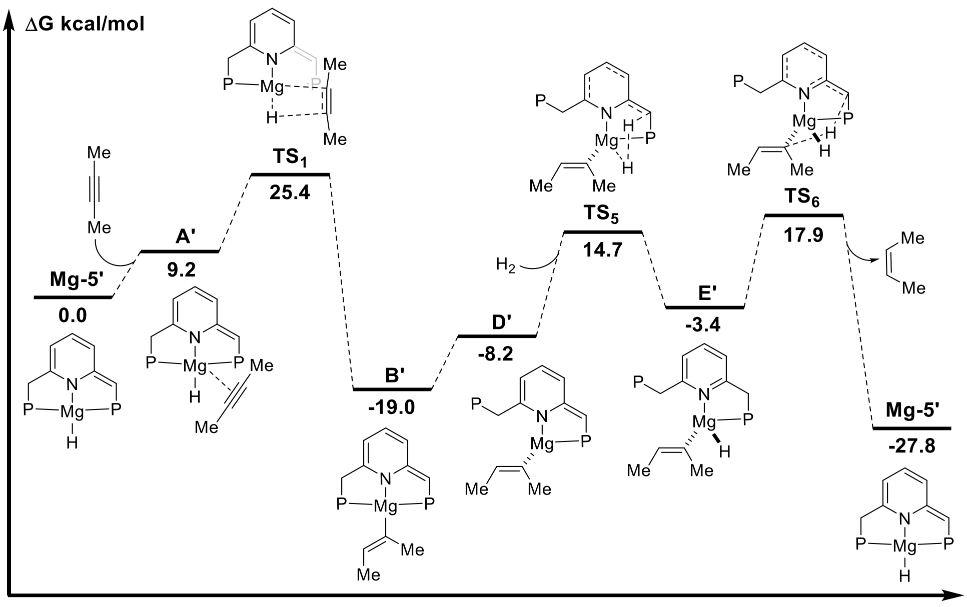

Figure S76. Pathway via the MLC process with opening a side arm

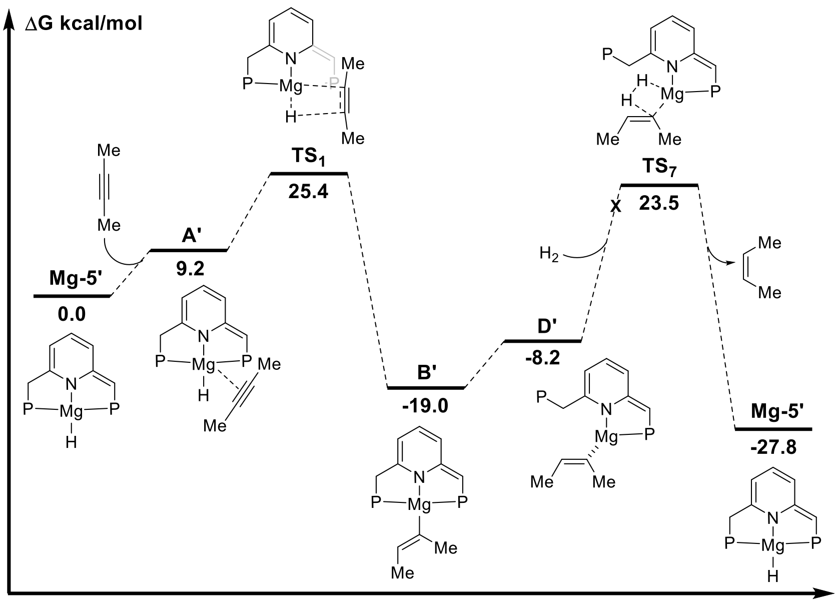

Figure S77. Pathway via the sigma bond metathesis with opening a side arm

Table S13. Energies of computed structures in energy diagrams

**Structure**	**$E^{\omega B97M\text{-}V}_{toluene}$**	**$G^{o\omega B97M\text{-}V}_{toluene}$**	**G**
Energy Unit	Hartree	Hartree	kcal
**Mg-5′**	-1839.781565	-1839.2617	-1154154.146
**A′**	-1995.745618	-1995.1502	-1251975.644
**TS_1**	-1995.719883	-1995.1244	-1251959.483
**B′**	-1995.792798	-1995.1952	-1252003.915
**TS_2**	-1996.926094	-1996.3148	-1252706.473
**C′**	-1996.955977	-1996.3393	-1252721.832
**TS_3**	-1996.921660	-1996.3069	-1252701.489
**TS_4**	-1996.918054	-1996.3038	-1252699.547
**D′**	-1995.775566	-1995.1779	-1251993.050
**TS_5**	-1996.918862	-1996.3075	-1252701.895
**E′**	-1996.952076	-1996.3363	-1252719.948
**TS_6**	-1996.913106	-1996.3024	-1252698.691
**TS_7**	-1996.904911	-1996.2934	-1252693.009
**Butyne**	-155.9525539	-155.90321	-97830.73997
**Butene**	-157.1850087	-157.11348	-98590.19451
**H_2**	-1.161490729	-1.1659877	-731.6683465

Cartesian Coordinates

Mg-5′
P 6.4900440000 8.2621460000 19.3387380000
P 8.0365060000 3.6468000000 18.6763290000
Mg 7.2045730000 6.1417130000 18.0703500000
N 8.9886400000 6.4711220000 19.1434460000
C 8.0351140000 8.4640360000 20.1378920000
H 8.2106220000 9.3053390000 20.8003270000
C 9.0938800000 7.5706940000 19.9773000000
C 10.3277950000 7.7481530000 20.6801590000
H 10.4316660000 8.6168790000 21.3175100000
C 11.3372300000 6.8428170000 20.5628170000
H 12.2631460000 6.9929650000 21.1058010000
C 11.1751380000 5.7002500000 19.7566610000
H 11.9475770000 4.9496400000 19.6675230000
C 9.9874140000 5.5653050000 19.0777800000
C 9.6943520000 4.3563010000 18.2398960000
H 10.5011630000 3.6247350000 18.2993930000
H 9.6108700000 4.6563730000 17.1911430000
C 6.2993660000 9.7601260000 18.1996460000
C 6.7333880000 11.0654730000 18.8563940000
H 6.0918950000 11.3605300000 19.6843940000

H 6.7018510000 11.8725040000 18.1196730000
H 7.7569310000 11.0031650000 19.2258060000
C 4.8838700000 9.8911430000 17.6543960000
H 4.5106780000 8.9491210000 17.2481060000
H 4.8673140000 10.6226480000 16.8425270000
H 4.1814740000 10.2410500000 18.4106550000
C 7.2533330000 9.4693330000 17.0388180000
H 8.2738590000 9.3044030000 17.3904020000
H 7.2766820000 10.3209930000 16.3545740000
H 6.9464330000 8.5934090000 16.4642590000
C 5.1386740000 8.2420660000 20.6572400000
C 4.9838830000 9.5664240000 21.3925880000
H 5.9322770000 9.9261290000 21.7939610000
H 4.2987240000 9.4475370000 22.2363300000
H 4.5682330000 10.3434370000 20.7517060000
C 3.8021210000 7.8202910000 20.0491150000
H 3.3729070000 8.5751880000 19.3952960000
H 3.0773130000 7.6370050000 20.8463770000
H 3.8932120000 6.8964460000 19.4745700000
C 5.5571700000 7.1598160000 21.6503610000
H 5.6289420000 6.1831520000 21.1659920000
H 4.8070060000 7.0739080000 22.4399950000
H 6.5199660000 7.3733810000 22.1139330000
C 8.2586990000 2.9714090000 20.4252000000
C 8.1604450000 4.1581490000 21.3893940000
H 9.0122510000 4.8308980000 21.3159530000
H 8.1336730000 3.7803720000 22.4143140000
H 7.2544900000 4.7432940000 21.2332060000
C 9.5948950000 2.2767290000 20.6634330000
H 9.7421690000 1.3998770000 20.0378220000
H 9.6462950000 1.9444810000 21.7032620000
H 10.4340390000 2.9552390000 20.5075380000
C 7.1032540000 2.0233440000 20.7387440000
H 6.1332290000 2.4894050000 20.5562280000
H 7.1379130000 1.7519110000 21.7960600000
H 7.1465860000 1.0975550000 20.1677390000
C 7.8021180000 2.2532140000 17.4290000000
C 8.6402230000 1.0053870000 17.6776840000
H 9.7061450000 1.2293150000 17.7288860000
H 8.4990470000 0.3073500000 16.8485410000
H 8.3592770000 0.4781540000 18.5877350000
C 8.1659410000 2.8110810000 16.0503780000
H 7.6935800000 3.7744790000 15.8516300000
H 7.8224870000 2.1111180000 15.2857740000

H 9.2420810000 2.9226100000 15.9228780000
C 6.3145440000 1.8962180000 17.4091320000
H 5.9601060000 1.4948650000 18.3566320000
H 6.1316590000 1.1367700000 16.6457060000
H 5.7030060000 2.7646620000 17.1614760000
H 6.8544110000 5.9628610000 16.3627510000

A′
P 6.7404940000 8.5081950000 19.0563890000
P 8.4885840000 3.6031330000 18.4434940000
Mg 7.1780710000 5.9776330000 18.4321050000
N 9.1202480000 6.4668970000 19.3194310000
C 8.1722500000 8.5951140000 20.0609470000
H 8.3063850000 9.4221600000 20.7518100000
C 9.1585700000 7.6155000000 20.1083580000
C 10.2624620000 7.7835630000 21.0079260000
H 10.2596460000 8.6577190000 21.6463420000
C 11.2781500000 6.8857900000 21.0556760000
H 12.1049890000 7.0305660000 21.7411850000
C 11.2512650000 5.7650770000 20.2056430000
H 12.0504270000 5.0373840000 20.1976460000
C 10.1693790000 5.6055960000 19.3720520000
C 10.1378270000 4.4127390000 18.4590170000
H 10.9582840000 3.7345680000 18.6983100000
H 10.3021560000 4.7509700000 17.4331810000
C 6.7912910000 10.0749830000 17.9881120000
C 7.2186460000 11.3185910000 18.7604680000
H 6.4999250000 11.6226690000 19.5176080000
H 7.3305570000 12.1564870000 18.0667210000
H 8.1827510000 11.1717630000 19.2473930000
C 5.4616410000 10.3228140000 17.2880440000
H 5.1015860000 9.4324210000 16.7685880000
H 5.5771010000 11.1111080000 16.5392630000
H 4.6836140000 10.6496890000 17.9769110000
C 7.8569430000 9.7950340000 16.9290260000
H 8.8120340000 9.5262840000 17.3844970000
H 8.0214450000 10.6883340000 16.3207760000
H 7.5546680000 8.9892000000 16.2613330000
C 5.2550240000 8.5807000000 20.2324970000
C 5.0520840000 9.9242490000 20.9191510000
H 5.9585090000 10.2746370000 21.4152560000
H 4.2789140000 9.8327140000 21.6874690000
H 4.7205050000 10.6986190000 20.2279390000
C 3.9860690000 8.1715180000 19.4864460000

H 3.7091610000 8.8723110000 18.7006870000
H 3.1458250000 8.1243940000 20.1846750000
H 4.0984140000 7.1807840000 19.0422080000
C 5.5303880000 7.5216860000 21.3006060000
H 5.6991910000 6.5375690000 20.8565480000
H 4.6613840000 7.4316900000 21.9574140000
H 6.3954330000 7.7726390000 21.9136380000
C 8.3509950000 2.8116130000 20.1549190000
C 7.9059270000 3.9169910000 21.1200020000
H 8.6593560000 4.6921500000 21.2509170000
H 7.7214340000 3.4776890000 22.1034080000
H 6.9741010000 4.3854800000 20.7957090000
C 9.6540030000 2.2170600000 20.6763440000
H 10.0498860000 1.4260850000 20.0429530000
H 9.4834140000 1.7847960000 21.6656300000
H 10.4254280000 2.9790470000 20.7938920000
C 7.2507960000 1.7538040000 20.1365160000
H 6.3189650000 2.1469330000 19.7270050000
H 7.0460190000 1.4324320000 21.1602690000
H 7.5287090000 0.8650240000 19.5721020000
C 8.6367690000 2.2873020000 17.1018520000
C 9.5533890000 1.1182660000 17.4395860000
H 10.5572090000 1.4471090000 17.7109410000
H 9.6525930000 0.4682050000 16.5661950000
H 9.1669540000 0.5032320000 18.2504410000
C 9.1757010000 2.9813070000 15.8490730000
H 8.6024290000 3.8748640000 15.5978350000
H 9.0964560000 2.2970810000 15.0016210000
H 10.2263930000 3.2539750000 15.9414570000
C 7.2311060000 1.7798940000 16.7734710000
H 6.7813320000 1.2161690000 17.5868440000
H 7.2764860000 1.1178050000 15.9056710000
H 6.5564380000 2.6015280000 16.5300610000
H 5.5674090000 5.2642040000 18.6745560000
C 7.8663410000 6.4248220000 15.8462940000
C 6.6795720000 6.1962570000 15.8047190000
C 9.2756830000 6.7562910000 15.8008590000
H 9.4478550000 7.5896900000 15.1184500000
H 9.6542980000 7.0522360000 16.7807920000
H 9.8724370000 5.9137090000 15.4482590000
C 5.2549160000 5.9756330000 15.6907040000
H 4.7055390000 6.5723990000 16.4198530000
H 4.9105250000 6.2496530000 14.6922290000
H 4.9919520000 4.9342150000 15.8709460000

TS$_1$
P 6.8240190000 8.5049910000 19.1501200000
P 8.4312740000 3.7334420000 18.5338970000
Mg 7.4142840000 6.1849790000 18.1756170000
N 9.2103180000 6.5468650000 19.3346590000
C 8.2226290000 8.5976020000 20.1966220000
H 8.3475300000 9.4158330000 20.8985880000
C 9.2467320000 7.6524370000 20.1732700000
C 10.3769240000 7.7988590000 21.0407690000
H 10.3982740000 8.6551250000 21.7028600000
C 11.3906040000 6.8938380000 21.0353030000
H 12.2379430000 7.0217410000 21.6989660000
C 11.3339480000 5.7858020000 20.1703380000
H 12.1195360000 5.0439920000 20.1401340000
C 10.2341600000 5.6643060000 19.3526100000
C 10.1135130000 4.4833830000 18.4324400000
H 10.9222800000 3.7725010000 18.6087030000
H 10.2078550000 4.8280050000 17.3989720000
C 6.8409120000 10.1014130000 18.1380740000
C 7.2101970000 11.3259720000 18.9675580000
H 6.4800120000 11.5500460000 19.7431110000
H 7.2725360000 12.2035030000 18.3182080000
H 8.1828510000 11.2055070000 19.4441190000
C 5.5189090000 10.3363640000 17.4199110000
H 5.1737820000 9.4434270000 16.8945290000
H 5.6373230000 11.1281160000 16.6754710000
H 4.7315470000 10.6565720000 18.1016560000
C 7.9359800000 9.8800850000 17.0936380000
H 8.8852880000 9.6079000000 17.5593690000
H 8.0987310000 10.7974990000 16.5220290000
H 7.6636520000 9.0928970000 16.3904200000
C 5.2874180000 8.4224020000 20.2482220000
C 5.0739850000 9.6526890000 21.1179270000
H 5.9682120000 9.9118820000 21.6869070000
H 4.2717260000 9.4708060000 21.8387290000
H 4.7805510000 10.5220770000 20.5295920000
C 4.0390200000 8.1363700000 19.4151190000
H 3.7097530000 8.9937020000 18.8332560000
H 3.2117990000 7.8622570000 20.0752570000
H 4.1999820000 7.3023890000 18.7277800000
C 5.5306280000 7.2049460000 21.1391220000
H 5.6530990000 6.2992200000 20.5370110000
H 4.6695020000 7.0410160000 21.7914780000
H 6.4175160000 7.3185790000 21.7625160000

C 8.3551750000 2.9715450000 20.2606990000
C 8.0145520000 4.1015110000 21.2379750000
H 8.8112900000 4.8370880000 21.3295240000
H 7.8502060000 3.6755170000 22.2307370000
H 7.0994380000 4.6226890000 20.9537880000
C 9.6582560000 2.3235880000 20.7150070000
H 9.9764220000 1.5030660000 20.0759360000
H 9.5284360000 1.9182540000 21.7216140000
H 10.4696870000 3.0501250000 20.7692600000
C 7.2095270000 1.9634640000 20.3143780000
H 6.2719120000 2.3953690000 19.9597130000
H 7.0528460000 1.6533660000 21.3499170000
H 7.4085580000 1.0627060000 19.7366100000
C 8.4466950000 2.3907190000 17.2105110000
C 9.2986360000 1.1701520000 17.5361290000
H 10.3336410000 1.4369870000 17.7525720000
H 9.3136680000 0.4998910000 16.6727180000
H 8.9136870000 0.5958980000 18.3767410000
C 8.9861910000 3.0218890000 15.9254940000
H 8.4632050000 3.9446970000 15.6745710000
H 8.8352660000 2.3240870000 15.0990370000
H 10.0546060000 3.2285190000 15.9791500000
C 6.9984220000 1.9752160000 16.9448540000
H 6.5393780000 1.4685810000 17.7906570000
H 6.9665320000 1.2863520000 16.0975740000
H 6.3777850000 2.8377800000 16.6978460000
H 5.8189120000 5.6659140000 17.4632190000
C 7.8328820000 6.4051900000 16.0005580000
C 6.6183340000 6.1334160000 15.9015860000
C 9.0957570000 6.8069680000 15.3612250000
H 8.9399820000 7.3907810000 14.4498350000
H 9.7375780000 7.3885770000 16.0250130000
H 9.6681000000 5.9203960000 15.0781360000
C 5.3572090000 5.9735890000 15.1793970000
H 4.5842240000 6.6342030000 15.5725360000
H 5.5089870000 6.2094220000 14.1221750000
H 4.9751710000 4.9554870000 15.2541190000

B′

P 6.8913190000 8.4339660000 19.1695660000
P 8.4901320000 3.8185120000 18.5289000000
Mg 7.8573050000 6.3951440000 17.9061240000
N 9.3665660000 6.5938520000 19.3498960000
C 8.2498500000 8.5368500000 20.2726880000

H 8.3011480000 9.3277780000 21.0139140000
C 9.3124840000 7.6340680000 20.2609820000
C 10.3845980000 7.7383200000 21.2034350000
H 10.3686380000 8.5619030000 21.9057010000
C 11.3879580000 6.8194490000 21.2272080000
H 12.1891710000 6.9133290000 21.9510580000
C 11.3777380000 5.7332890000 20.3309030000
H 12.1441000000 4.9714960000 20.3481730000
C 10.3476330000 5.6694260000 19.4229080000
C 10.2092700000 4.5216360000 18.4659210000
H 10.9825610000 3.7701720000 18.6309760000
H 10.3325980000 4.8886680000 17.4421300000
C 6.9346090000 10.0380020000 18.1639320000
C 7.2709100000 11.2589980000 19.0126910000
H 6.5123800000 11.4831080000 19.7601140000
H 7.3587270000 12.1371440000 18.3675050000
H 8.2247610000 11.1362700000 19.5253160000
C 5.6418030000 10.2732440000 17.3944260000
H 5.3322680000 9.3892800000 16.8333590000
H 5.7855270000 11.0808440000 16.6720270000
H 4.8207370000 10.5723740000 18.0455270000
C 8.0688910000 9.8255490000 17.1589780000
H 9.0017230000 9.5457830000 17.6544580000
H 8.2563300000 10.7502340000 16.6074280000
H 7.8198310000 9.0498650000 16.4341670000
C 5.3034980000 8.3369110000 20.1896530000
C 5.0100920000 9.5982440000 20.9906050000
H 5.8579890000 9.8969730000 21.6085580000
H 4.1646600000 9.4258500000 21.6623290000
H 4.7419450000 10.4381670000 20.3504790000
C 4.1144950000 7.9936860000 19.2936170000
H 3.8274460000 8.8074030000 18.6328510000
H 3.2438060000 7.7595510000 19.9114860000
H 4.3165930000 7.1181370000 18.6730820000
C 5.5076250000 7.1685050000 21.1511220000
H 5.6626890000 6.2329840000 20.6091200000
H 4.6157650000 7.0389190000 21.7688330000
H 6.3621720000 7.3187310000 21.8104690000
C 8.3631660000 3.0552420000 20.2532060000
C 8.0733020000 4.1930780000 21.2370040000
H 8.9223450000 4.8596020000 21.3720140000
H 7.8391030000 3.7650200000 22.2146560000
H 7.2182380000 4.7938080000 20.9296020000
C 9.6248870000 2.3366940000 20.7190380000

H 9.8964060000 1.4903700000 20.0929970000
H 9.4660120000 1.9530270000 21.7299720000
H 10.4774240000 3.0143570000 20.7700640000
C 7.1690160000 2.1038150000 20.2847820000
H 6.2558580000 2.5835950000 19.9277330000
H 6.9876660000 1.7896800000 21.3149670000
H 7.3294570000 1.2012710000 19.6978960000
C 8.5090760000 2.4774290000 17.2015520000
C 9.3010640000 1.2240930000 17.5520930000
H 10.3350980000 1.4494630000 17.8149140000
H 9.3284060000 0.5627720000 16.6822890000
H 8.8585200000 0.6561260000 18.3686020000
C 9.1158460000 3.0952700000 15.9401150000
H 8.6426000000 4.0431710000 15.6818310000
H 8.9599470000 2.4117780000 15.1026860000
H 10.1901790000 3.2529830000 16.0295110000
C 7.0577480000 2.1136600000 16.8822670000
H 6.5375950000 1.6566660000 17.7214680000
H 7.0350840000 1.3978410000 16.0575620000
H 6.4891630000 2.9907540000 16.5705470000
C 7.8105510000 6.4450100000 15.7874290000
C 6.5644340000 6.2611680000 15.3132370000
C 8.9569210000 6.7348650000 14.8625220000
H 8.7132880000 6.7957450000 13.7961040000
H 9.4434750000 7.6789950000 15.1284160000
H 9.7440420000 5.9791550000 14.9590990000
C 6.0540420000 6.2845870000 13.9046500000
H 5.2824460000 7.0482130000 13.7713290000
H 6.8341040000 6.4806720000 13.1711870000
H 5.5843540000 5.3344250000 13.6341940000
H 5.7618660000 6.0609500000 16.0348690000

TS_2

P 8.4451530000 3.8620170000 18.4685590000
Mg 7.3871450000 6.4304430000 18.2525140000
N 9.1508830000 6.6604470000 19.5373930000
C 9.0672440000 7.6714390000 20.4500040000
C 9.9641060000 7.7102580000 21.5331390000
H 9.8630220000 8.4985550000 22.2676070000
C 10.9652550000 6.7763000000 21.6237960000
H 11.6791780000 6.8195320000 22.4372310000
C 11.0636880000 5.7727980000 20.6598910000
H 11.8479000000 5.0292700000 20.6980020000
C 10.1249130000 5.7359200000 19.6470650000

C 10.1181900000 4.6547480000 18.6143890000
H 10.9181640000 3.9350250000 18.7914890000
H 10.3049760000 5.1106030000 17.6387770000
C 8.2979470000 2.8566480000 20.0627620000
C 7.7730880000 3.8247660000 21.1280360000
H 8.4646450000 4.6410870000 21.3351720000
H 7.6245480000 3.2826290000 22.0652720000
H 6.8179050000 4.2651770000 20.8421660000
C 9.6065320000 2.2497890000 20.5551650000
H 10.0636390000 1.5747220000 19.8342990000
H 9.4188160000 1.6737220000 21.4647990000
H 10.3373750000 3.0162870000 20.8139180000
C 7.2516630000 1.7606370000 19.8800590000
H 6.3166390000 2.1518950000 19.4756200000
H 7.0230590000 1.3163460000 20.8515290000
H 7.5942040000 0.9559050000 19.2310270000
C 8.6754310000 2.7361050000 16.9732230000
C 9.6465540000 1.5817100000 17.1826860000
H 10.6272590000 1.9210470000 17.5188110000
H 9.7959920000 1.0541480000 16.2369590000
H 9.2764150000 0.8505920000 17.8999850000
C 9.1903660000 3.6190330000 15.8324320000
H 8.6010830000 4.5321110000 15.7263580000
H 9.1091730000 3.0686220000 14.8928950000
H 10.2386370000 3.8894290000 15.9533340000
C 7.3052550000 2.2041390000 16.5503420000
H 6.8624170000 1.5330610000 17.2822970000
H 7.4085010000 1.6433940000 15.6184550000
H 6.6060230000 3.0209180000 16.3684170000
C 6.5748080000 6.3405410000 16.2846920000
C 5.5051560000 5.5397200000 16.1346600000
C 7.0353350000 7.2324140000 15.1681290000
H 6.3673640000 7.3114610000 14.3023820000
H 7.2069750000 8.2531210000 15.5291920000
H 8.0074430000 6.9048790000 14.7813360000
C 4.6118440000 5.3364540000 14.9486160000
H 3.5617190000 5.5076770000 15.2007210000
H 4.8573730000 5.9887890000 14.1119980000
H 4.6660030000 4.3067090000 14.5807780000
H 5.2141080000 4.9106700000 16.9840030000
P 7.6624720000 9.1679900000 18.6092230000
C 9.7095570000 11.0746060000 19.2835070000
C 8.8324980000 11.1188300000 16.9436870000
C 10.2506410000 9.2437610000 17.7047030000

C 9.1613550000 10.2305710000 18.1388030000
C 5.5455270000 10.5006940000 17.3930900000
C 6.3363510000 11.5518480000 19.5245810000
C 5.0886270000 9.4062210000 19.5497950000
C 6.1209050000 10.2370930000 18.7860130000
C 7.9704960000 8.5902480000 20.2978410000
H 9.9850680000 10.4590150000 20.1402790000
H 10.6129920000 11.5969870000 18.9563710000
H 9.0071890000 11.8311350000 19.6272070000
H 8.1428950000 11.9226550000 17.1973560000
H 9.7484680000 11.5866650000 16.5738750000
H 8.4030640000 10.5505170000 16.1169070000
H 9.9055960000 8.5736230000 16.9142930000
H 11.1103670000 9.7971300000 17.3180370000
H 10.6088420000 8.6331260000 18.5324810000
H 5.3436240000 9.5674930000 16.8655420000
H 4.5981890000 11.0375180000 17.4862490000
H 6.1966090000 11.1055060000 16.7659390000
H 6.9660960000 12.2448090000 18.9678990000
H 5.3745870000 12.0487520000 19.6791410000
H 6.7829680000 11.4028410000 20.5084790000
H 5.3759790000 9.2309040000 20.5855000000
H 4.1361410000 9.9412670000 19.5556020000
H 4.9163220000 8.4364950000 19.0806390000
H 7.8962950000 9.3233580000 21.0999700000
H 6.4612360000 6.7288890000 19.9170940000
H 6.9722420000 7.5734870000 20.2228100000

C′

P 8.1686710000 3.7232210000 18.4605330000
Mg 7.0133400000 6.3325290000 18.2508220000
N 8.9246870000 6.5548880000 19.4960140000
C 9.0074100000 7.5393030000 20.4123790000
C 10.1102520000 7.6633590000 21.2494190000
H 10.1356030000 8.4607270000 21.9798320000
C 11.1559020000 6.7681800000 21.1284600000
H 12.0270820000 6.8515160000 21.7657210000
C 11.0736460000 5.7657560000 20.1803250000
H 11.8730340000 5.0487300000 20.0500560000
C 9.9395590000 5.6775730000 19.3826330000
C 9.8032790000 4.5993690000 18.3612120000
H 10.6610930000 3.9257640000 18.4001810000
H 9.8010770000 5.0647280000 17.3712580000
C 8.2287550000 2.8487370000 20.1376990000

C 7.8717070000 3.9034630000 21.1937880000
H 8.6889900000 4.5998280000 21.3765330000
H 7.6676840000 3.4013780000 22.1428080000
H 6.9875920000 4.4830290000 20.9226710000
C 9.5674040000 2.2284350000 20.5205180000
H 9.8701610000 1.4145110000 19.8667200000
H 9.5009250000 1.8202800000 21.5327640000
H 10.3694830000 2.9678370000 20.5346680000
C 7.1286860000 1.7895000000 20.1583780000
H 6.1619850000 2.2064770000 19.8707950000
H 7.0238900000 1.3946260000 21.1715410000
H 7.3434710000 0.9453780000 19.5048350000
C 8.3313200000 2.4653420000 17.0556010000
C 9.2672520000 1.2941370000 17.3243130000
H 10.2719480000 1.6209250000 17.5955950000
H 9.3609350000 0.6922390000 16.4163220000
H 8.9018710000 0.6315880000 18.1072260000
C 8.8381990000 3.2124060000 15.8191980000
H 8.2737680000 4.1270930000 15.6348290000
H 8.7190980000 2.5683970000 14.9447400000
H 9.8973490000 3.4601280000 15.8891860000
C 6.9249430000 1.9522330000 16.7412860000
H 6.4828540000 1.3942960000 17.5644790000
H 6.9635490000 1.2835010000 15.8779450000
H 6.2527490000 2.7747070000 16.4954780000
C 6.9667600000 6.2928570000 16.1124980000
C 5.9242420000 5.5614590000 15.6778910000
C 7.8738550000 6.9817380000 15.1348460000
H 7.6318820000 6.8539110000 14.0727440000
H 7.8948820000 8.0613370000 15.3204200000
H 8.9136190000 6.6545970000 15.2561760000
C 5.4803440000 5.2460110000 14.2808360000
H 4.4308610000 5.5070940000 14.1199000000
H 6.0629810000 5.7595160000 13.5173060000
H 5.5555300000 4.1724330000 14.0752490000
H 5.2897090000 5.0785200000 16.4297430000
P 7.3737820000 9.1130870000 18.8155150000
C 9.4912580000 11.0077450000 19.3652930000
C 8.4246780000 11.0434630000 17.1027890000
C 9.9175740000 9.1855160000 17.7440970000
C 8.8548770000 10.1587820000 18.2706840000
C 5.1470550000 10.4236350000 17.8702780000
C 6.1930270000 11.5556720000 19.8452640000
C 4.9659180000 9.4216720000 20.1231080000

C 5.8881860000 10.2136290000 19.1931350000
C 7.8695090000 8.4998840000 20.4934070000
H 9.8556340000 10.3969210000 20.1924770000
H 10.3578170000 11.5367370000 18.9592860000
H 8.8179300000 11.7585570000 19.7718140000
H 7.7412440000 11.8356580000 17.4034200000
H 9.3043910000 11.5245000000 16.6689520000
H 7.9465900000 10.4659710000 16.3099730000
H 9.5069530000 8.4691630000 17.0313210000
H 10.6994810000 9.7518600000 17.2320200000
H 10.4039440000 8.6283500000 18.5431420000
H 4.8799870000 9.4710250000 17.4105850000
H 4.2223800000 10.9764800000 18.0518560000
H 5.7265610000 10.9910080000 17.1443840000
H 6.7487600000 12.2281800000 19.1935640000
H 5.2544480000 12.0580380000 20.0947180000
H 6.7517590000 11.4437570000 20.7753150000
H 5.3440880000 9.3878010000 21.1447970000
H 3.9906210000 9.9121420000 20.1575530000
H 4.8148200000 8.3948640000 19.7861580000
H 8.0820590000 9.2902210000 21.2147220000
H 5.7282180000 6.2516570000 19.4835550000
H 6.9896130000 7.9511780000 20.8443200000

TS_3

Mg 15.1447520000 6.8390060000 4.5989750000
P 12.5369650000 6.3237130000 4.4013510000
P 17.4880920000 5.9735210000 3.3330250000
N 15.0261910000 4.6072130000 4.4887360000
H 15.2027060000 8.5639410000 4.1534440000
C 10.5244130000 6.1855300000 2.3439350000
C 11.2629140000 8.4392360000 3.1246190000
C 12.8106730000 7.0156250000 1.8052230000
C 11.7030190000 7.0022250000 2.8634320000
C 10.0003670000 6.0577180000 5.7221410000
C 11.5484560000 7.8799270000 6.4650250000
C 11.4554920000 6.4513620000 5.9267060000
C 18.8409270000 3.6462880000 2.3165440000
C 18.4727170000 5.5401960000 0.7281110000
C 16.5675270000 4.1605810000 1.4802490000
C 17.8999640000 4.7763990000 1.9168890000
C 18.9833120000 8.2261290000 2.6824600000
C 20.3159900000 6.3839600000 3.7339410000
C 18.6927360000 7.7054190000 5.0704450000

C 18.9641010000 7.0843260000 3.7003210000
C 12.6365020000 4.5163890000 4.0435950000
C 13.9018570000 3.8716950000 4.5373260000
C 13.9059370000 2.5529460000 4.9555020000
C 15.1187250000 1.9664620000 5.3154220000
C 16.2759070000 2.7024220000 5.2427200000
C 16.2231930000 4.0376080000 4.8120410000
C 17.3603200000 4.9293230000 4.8148840000
C 12.1042540000 5.5257840000 6.9568680000
H 9.6894520000 6.1583320000 3.0406000000
H 10.1560910000 6.6330470000 1.4173880000
H 10.7986720000 5.1570550000 2.1085970000
H 12.0651650000 9.0384960000 3.5576840000
H 10.9734160000 8.9096470000 2.1823780000
H 10.3987360000 8.4869730000 3.7869390000
H 13.2042790000 6.0176170000 1.6026660000
H 12.4153540000 7.4004750000 0.8625510000
H 13.6441150000 7.6551650000 2.1019850000
H 9.8950880000 5.0626330000 5.2869880000
H 9.4825620000 6.0446530000 6.6846060000
H 9.4715860000 6.7651450000 5.0842100000
H 11.0506570000 8.6062120000 5.8263410000
H 11.0728690000 7.9302090000 7.4472600000
H 12.5861800000 8.1966260000 6.5828440000
H 18.4319060000 3.0521780000 3.1341970000
H 18.9855180000 2.9708070000 1.4686550000
H 19.8247760000 3.9993290000 2.6186240000
H 19.4805280000 5.9077900000 0.9149770000
H 18.5285490000 4.8777910000 -0.1396060000
H 17.8472090000 6.3896200000 0.4488400000
H 15.8411850000 4.9239090000 1.1984990000
H 16.7306330000 3.5190530000 0.6102200000
H 16.1217770000 3.5403310000 2.2570820000
H 18.0252620000 8.7467910000 2.6565220000
H 19.7472220000 8.9538890000 2.9672760000
H 19.2155140000 7.8960550000 1.6725310000
H 20.6263470000 6.0262460000 2.7527530000
H 21.0838890000 7.0839440000 4.0750150000
H 20.3246500000 5.5378210000 4.4223440000
H 18.7672510000 6.9824440000 5.8813150000
H 19.4251450000 8.4940570000 5.2592280000
H 17.7022520000 8.1640470000 5.1150090000
H 12.6329600000 4.4261230000 2.9536360000
H 11.7594950000 3.9674630000 4.3909610000

H 12.9815360000 1.9915400000 4.9784000000
H 15.1471570000 0.9343190000 5.6423840000
H 17.2325430000 2.2756480000 5.5157660000
H 18.2803520000 4.4591320000 5.1630700000
H 13.1469030000 5.7968180000 7.1315630000
H 11.5791160000 5.6196920000 7.9098730000
H 12.0679140000 4.4744740000 6.6684060000
H 16.7969230000 5.8390460000 5.7069150000
C 15.8941210000 6.4235220000 6.7520880000
C 15.8086770000 7.6759010000 7.2354010000
C 15.7174640000 5.2237780000 7.6397550000
H 15.4271520000 5.4553000000 8.6669300000
H 14.9804400000 4.5173510000 7.2484160000
H 16.6525370000 4.6576150000 7.7015730000
C 15.4680340000 8.1330180000 8.6172200000
H 14.6999450000 8.9091820000 8.5939740000
H 15.1122580000 7.3326110000 9.2639980000
H 16.3346640000 8.5871920000 9.1065180000
H 15.9940200000 8.5045980000 6.5489320000

TS$_4$
P 6.8794350000 8.4669160000 19.3428780000
P 8.6206120000 3.8058010000 18.8863400000
Mg 7.5830100000 6.2243100000 18.3231100000
N 9.3681080000 6.6417670000 19.4274170000
C 8.2789810000 8.5955260000 20.3822820000
H 8.3869740000 9.4241230000 21.0756670000
C 9.3709580000 7.7338230000 20.2827300000
C 10.5380130000 7.9498790000 21.0831710000
H 10.5469280000 8.8066380000 21.7447210000
C 11.6021530000 7.1063370000 21.0170060000
H 12.4786100000 7.2895200000 21.6276500000
C 11.5571830000 5.9836870000 20.1698320000
H 12.3713990000 5.2749520000 20.1173910000
C 10.4244640000 5.7967670000 19.4131440000
C 10.2729030000 4.5686260000 18.5615440000
H 11.1118870000 3.8871770000 18.7124030000
H 10.2685260000 4.8421740000 17.5028410000
C 6.8440000000 10.0413520000 18.3069160000
C 7.0511210000 11.3146880000 19.1166640000
H 6.2335730000 11.5269210000 19.8004810000
H 7.1292180000 12.1667640000 18.4360540000
H 7.9777190000 11.2744250000 19.6900030000
C 5.5475200000 10.1019620000 17.5030850000

H 5.3375330000 9.1500250000 17.0115560000
H 5.6229590000 10.8627910000 16.7218190000
H 4.6810710000 10.3561420000 18.1132140000
C 8.0244730000 9.9114520000 17.3404610000
H 8.9703300000 9.8333580000 17.8793520000
H 8.0786590000 10.7982440000 16.7037640000
H 7.9318690000 9.0410160000 16.6933790000
C 5.3114060000 8.3532170000 20.3931190000
C 4.8040280000 9.6832290000 20.9398820000
H 5.5843460000 10.2127100000 21.4876290000
H 3.9883410000 9.5040120000 21.6458790000
H 4.4203270000 10.3508040000 20.1708470000
C 4.2257040000 7.6581180000 19.5737900000
H 3.8604220000 8.2833150000 18.7576530000
H 3.3672430000 7.4237550000 20.2087260000
H 4.5798500000 6.7230580000 19.1350770000
C 5.6699620000 7.4601530000 21.5775060000
H 6.0307510000 6.4816770000 21.2459530000
H 4.7820160000 7.2844100000 22.1893040000
H 6.4444280000 7.8961990000 22.2058680000
C 8.7041060000 3.1574330000 20.6502120000
C 8.5293510000 4.3392890000 21.6090530000
H 9.3821370000 5.0144710000 21.6065470000
H 8.4231160000 3.9547590000 22.6262530000
H 7.6344360000 4.9216660000 21.3874980000
C 10.0068350000 2.4518830000 21.0063060000
H 10.2116820000 1.5814260000 20.3895740000
H 9.9589940000 2.1102570000 22.0432860000
H 10.8573310000 3.1315590000 20.9356380000
C 7.4977860000 2.2364570000 20.8457500000
H 6.5665060000 2.7428520000 20.5845440000
H 7.4260270000 1.9489910000 21.8969040000
H 7.5507910000 1.3199920000 20.2605050000
C 8.4936160000 2.3620550000 17.6880210000
C 9.2659860000 1.1061180000 18.0782840000
H 10.3346190000 1.3051370000 18.1742970000
H 9.1542120000 0.3575160000 17.2896130000
H 8.9270150000 0.6499390000 19.0061060000
C 9.0337270000 2.8503840000 16.3440530000
H 8.5746360000 3.7946890000 16.0450460000
H 8.7926130000 2.1162070000 15.5726840000
H 10.1157340000 2.9738110000 16.3467840000
C 7.0056070000 2.0519920000 17.5111960000
H 6.5526710000 1.6525180000 18.4182700000

H 6.8775650000 1.3024330000 16.7267960000
H 6.4448630000 2.9418580000 17.2224240000
C 7.7575100000 6.4914690000 15.9967820000
C 6.7734380000 7.1687020000 15.3929840000
C 9.1932050000 6.5826790000 15.5742210000
H 9.3611050000 7.2098970000 14.6927800000
H 9.8208080000 6.9944500000 16.3726030000
H 9.6146830000 5.6011740000 15.3435320000
C 6.8341990000 8.1362780000 14.2645710000
H 6.4230100000 9.1052930000 14.5604970000
H 7.8461090000 8.3076850000 13.8999120000
H 6.2282050000 7.7997130000 13.4197890000
H 5.7632910000 6.9976750000 15.7847680000
H 6.1223200000 5.3254580000 17.3897900000
H 6.8964680000 5.6117060000 16.7469160000

D′
P 7.2165810000 8.3422090000 19.2919230000
P 11.9103960000 3.7216660000 18.4443650000
Mg 8.4850660000 6.9700740000 17.6077570000
N 10.0605830000 7.1412940000 18.9487670000
C 8.6912380000 8.7718640000 20.1260940000
H 8.6776850000 9.5135780000 20.9174980000
C 9.9176160000 8.1313800000 19.9107520000
C 11.0650190000 8.4720150000 20.6858240000
H 10.9669060000 9.2555520000 21.4258930000
C 12.2469010000 7.8181840000 20.5065110000
H 13.1082640000 8.0864270000 21.1072520000
C 12.3476380000 6.7823570000 19.5657270000
H 13.2560500000 6.2105590000 19.4407760000
C 11.2338000000 6.4755940000 18.8189070000
C 11.2533110000 5.3522590000 17.8244230000
H 11.9166870000 5.6132900000 16.9946890000
H 10.2630930000 5.2091990000 17.3802850000
C 6.4730890000 9.9565600000 18.6731090000
C 6.5823970000 11.0853800000 19.6921530000
H 6.0293060000 10.8804350000 20.6075140000
H 6.1742970000 12.0051140000 19.2655820000
H 7.6192700000 11.2836680000 19.9608620000
C 5.0227470000 9.7878850000 18.2400800000
H 4.8859960000 8.9340170000 17.5741180000
H 4.6950200000 10.6788270000 17.6990870000
H 4.3530960000 9.6681970000 19.0910390000
C 7.3175860000 10.3198010000 17.4493830000

H 8.3787380000 10.3824220000 17.6994260000
H 7.0155790000 11.2942750000 17.0586810000
H 7.1942100000 9.5941220000 16.6417970000
C 6.0706850000 7.4413070000 20.4941060000
C 5.6374650000 8.3129020000 21.6637620000
H 6.4910100000 8.7710910000 22.1659450000
H 5.1069590000 7.7118850000 22.4070410000
H 4.9588720000 9.1065070000 21.3504260000
C 4.8523040000 6.8621920000 19.7794460000
H 4.1243840000 7.6181040000 19.4965480000
H 4.3407080000 6.1581710000 20.4403160000
H 5.1306120000 6.3139680000 18.8771860000
C 6.9117680000 6.2726290000 21.0106310000
H 7.2194290000 5.6058320000 20.1991700000
H 6.3223480000 5.6740350000 21.7088400000
H 7.8110130000 6.6083420000 21.5263610000
C 12.0518670000 2.8355740000 16.7722690000
C 13.4050940000 3.2862600000 16.2151020000
H 13.4618950000 4.3688060000 16.0877460000
H 13.5720370000 2.8379360000 15.2324090000
H 14.2284580000 2.9875020000 16.8639770000
C 10.9725910000 3.1662600000 15.7445250000
H 9.9615540000 2.9763370000 16.1008840000
H 11.1209180000 2.5556390000 14.8493080000
H 11.0183290000 4.2057540000 15.4240740000
C 12.1074250000 1.3270590000 16.9874380000
H 12.8138560000 1.0496570000 17.7717380000
H 12.4331410000 0.8362190000 16.0668520000
H 11.1340730000 0.9103090000 17.2437900000
C 10.4439000000 3.0168610000 19.4144460000
C 9.1669140000 2.7691110000 18.6241950000
H 8.8417630000 3.6512710000 18.0677640000
H 8.3528620000 2.5007110000 19.3049060000
H 9.2700660000 1.9495240000 17.9132210000
C 10.1480210000 4.0296430000 20.5236300000
H 9.4399270000 3.5942990000 21.2337760000
H 9.6998450000 4.9452860000 20.1422690000
H 11.0451270000 4.3066940000 21.0802260000
C 10.8965830000 1.7220880000 20.0904040000
H 11.0594350000 0.9088410000 19.3869650000
H 10.1294560000 1.3904020000 20.7947220000
H 11.8192850000 1.8663560000 20.6541040000
C 8.1176490000 5.8641710000 15.8693020000
C 7.2248620000 4.8897460000 16.1284050000

C 8.8173070000 5.9415810000 14.5440610000
H 8.7431990000 5.0478650000 13.9156010000
H 8.4221000000 6.7729650000 13.9519340000
H 9.8828120000 6.1603100000 14.6610800000
C 6.7799160000 3.7432900000 15.2753560000
H 5.7154640000 3.8116480000 15.0350920000
H 7.3208170000 3.6778300000 14.3329580000
H 6.9086610000 2.7910450000 15.7977140000
H 6.7294690000 4.8865000000 17.1070710000

TS_5

P 7.0301870000 8.4620250000 19.0933490000
P 11.2271860000 3.2021280000 18.7634650000
Mg 8.5951480000 7.3223820000 17.3119980000
N 9.7365640000 6.9352400000 19.0951140000
C 8.6469120000 8.9752260000 19.7188520000
H 8.6682320000 9.7994600000 20.4266820000
C 9.6489170000 7.9671440000 19.9817510000
C 10.5880020000 8.0850730000 21.0158920000
H 10.5125600000 8.9176670000 21.7027630000
C 11.5772850000 7.1394690000 21.1432550000
H 12.3019600000 7.2157110000 21.9448330000
C 11.6370260000 6.0742420000 20.2504650000
H 12.3840180000 5.2980660000 20.3449180000
C 10.7002420000 5.9990210000 19.2369020000
C 10.6937060000 4.8989080000 18.2192410000
H 11.3936830000 5.1656030000 17.4198610000
H 9.7109010000 4.8572760000 17.7401800000
C 6.1999240000 10.1103660000 18.7039670000
C 6.4224520000 11.1846740000 19.7646730000
H 6.0732230000 10.8891700000 20.7520570000
H 5.8723610000 12.0857550000 19.4823130000
H 7.4708620000 11.4669640000 19.8462920000
C 4.7021940000 9.9069440000 18.4964110000
H 4.4873280000 9.0929870000 17.8025680000
H 4.2728840000 10.8178700000 18.0734710000
H 4.1770620000 9.7106690000 19.4300530000
C 6.7980540000 10.6057340000 17.3852180000
H 7.8646300000 10.8084100000 17.4626370000
H 6.3075580000 11.5387130000 17.0972750000
H 6.6493540000 9.8921510000 16.5734840000
C 6.0899210000 7.5188860000 20.4309040000
C 5.7605940000 8.3463440000 21.6647610000
H 6.6485220000 8.8230960000 22.0831330000

H 5.3396400000 7.7028890000 22.4418400000
H 5.0228020000 9.1209470000 21.4582540000
C 4.8181200000 6.9092440000 19.8384480000
H 4.0441460000 7.6430620000 19.6330990000
H 4.4000370000 6.1915920000 20.5482020000
H 5.0224810000 6.3694350000 18.9124590000
C 6.9967350000 6.3550310000 20.8321950000
H 7.2881690000 5.7572550000 19.9656360000
H 6.4572270000 5.6949660000 21.5154700000
H 7.9035920000 6.6805880000 21.3399210000
C 11.4318820000 2.4262700000 17.0459970000
C 12.8370980000 2.8427660000 16.6025990000
H 12.9506020000 3.9268700000 16.5466950000
H 13.0457630000 2.4466850000 15.6056950000
H 13.6030240000 2.4653880000 17.2803410000
C 10.4345570000 2.8777530000 15.9824390000
H 9.3979260000 2.6910520000 16.2555250000
H 10.6270860000 2.3389280000 15.0503060000
H 10.5266490000 3.9382940000 15.7556830000
C 11.4007940000 0.9060840000 17.1618860000
H 12.0398030000 0.5413700000 17.9681120000
H 11.7613640000 0.4590760000 16.2321370000
H 10.3931900000 0.5263610000 17.3279410000
C 9.6599030000 2.5300340000 19.5859300000
C 8.4260220000 2.4226980000 18.7012020000
H 8.1944730000 3.3609270000 18.1931010000
H 7.5536770000 2.1636720000 19.3090450000
H 8.5269090000 1.6459880000 17.9435490000
C 9.3585000000 3.4898850000 20.7382310000
H 8.5484180000 3.0839790000 21.3498600000
H 9.0367150000 4.4683650000 20.3873090000
H 10.2224070000 3.6355230000 21.3891060000
C 9.9817110000 1.1670560000 20.1992970000
H 10.1370290000 0.3914430000 19.4528210000
H 9.1501010000 0.8444960000 20.8309190000
H 10.8733280000 1.2097820000 20.8264720000
C 8.0900690000 5.9231680000 15.8143290000
C 7.1379090000 5.0767920000 16.2494480000
C 8.7015010000 5.7632610000 14.4530870000
H 8.5013160000 4.8108490000 13.9496650000
H 8.3478960000 6.5517780000 13.7812130000
H 9.7882080000 5.8865830000 14.4801630000
C 6.5278830000 3.8861330000 15.5743490000
H 5.4737010000 4.0567540000 15.3373250000

H 7.0267290000 3.6224900000 14.6429660000
H 6.5493470000 3.0046420000 16.2211660000
H 6.7089370000 5.2517600000 17.2445330000
H 9.4205770000 9.0462880000 17.4141070000
H 9.1247040000 9.2369760000 18.3981170000

E′
P 7.0704460000 8.7957140000 18.9706970000
P 11.2492920000 3.2723510000 18.2744680000
Mg 8.1580000000 6.9424180000 17.3680060000
N 9.4805850000 6.8665270000 19.1703620000
C 8.6393260000 9.0428910000 19.9080730000
H 8.4789270000 9.5617920000 20.8549990000
C 9.4545000000 7.8032290000 20.1330960000
C 10.2011890000 7.6700470000 21.2960240000
H 10.1436980000 8.4354820000 22.0585060000
C 11.0096900000 6.5612980000 21.4509430000
H 11.5976010000 6.4328440000 22.3510690000
C 11.0725410000 5.6237450000 20.4369070000
H 11.7140670000 4.7564020000 20.5107360000
C 10.3007400000 5.8016420000 19.2973700000
C 10.3563960000 4.8904320000 18.1062320000
H 10.8626930000 5.4519090000 17.3144370000
H 9.3372130000 4.7244490000 17.7316810000
C 6.7256640000 10.5258200000 18.3208070000
C 7.0207320000 11.6515920000 19.3073670000
H 6.4153120000 11.6009940000 20.2092880000
H 6.8075330000 12.6108030000 18.8295240000
H 8.0691230000 11.6751740000 19.6041950000
C 5.2753810000 10.6081320000 17.8534080000
H 5.0125470000 9.7835100000 17.1891320000
H 5.1263660000 11.5353730000 17.2960700000
H 4.5698380000 10.6151230000 18.6832540000
C 7.6349460000 10.6911290000 17.1008800000
H 8.6921510000 10.5774660000 17.3435080000
H 7.5026660000 11.6918070000 16.6833610000
H 7.4072150000 9.9676000000 16.3188190000
C 5.8360860000 8.2830760000 20.2912140000
C 5.4456390000 9.3774310000 21.2753190000
H 6.3130790000 9.8333380000 21.7550530000
H 4.8298490000 8.9475820000 22.0694330000
H 4.8581320000 10.1674400000 20.8098520000
C 4.5922810000 7.7346810000 19.5916910000
H 4.0423630000 8.5040630000 19.0530960000

H 3.9130180000 7.3109750000 20.3348540000
H 4.8444990000 6.9446640000 18.8828460000
C 6.4966290000 7.1429830000 21.0707360000
H 6.9257900000 6.3797960000 20.4181910000
H 5.7472180000 6.6513460000 21.6942910000
H 7.2839930000 7.5007660000 21.7342780000
C 11.8359720000 3.0555710000 16.4897260000
C 13.0834390000 3.9380020000 16.3901050000
H 12.8598100000 4.9924990000 16.5606250000
H 13.5135480000 3.8605250000 15.3886880000
H 13.8483390000 3.6403180000 17.1077390000
C 10.8642680000 3.4672800000 15.3876850000
H 9.9532150000 2.8743830000 15.3798380000
H 11.3444730000 3.3308130000 14.4147890000
H 10.5805980000 4.5178800000 15.4485570000
C 12.2570490000 1.6035840000 16.2851990000
H 12.9144610000 1.2526830000 17.0828730000
H 12.8032040000 1.5052130000 15.3438790000
H 11.4019360000 0.9305170000 16.2307670000
C 9.8215880000 2.0885210000 18.6446060000
C 8.8235310000 1.8538840000 17.5196330000
H 8.3850800000 2.7834890000 17.1539070000
H 7.9988680000 1.2350490000 17.8844650000
H 9.2672920000 1.3239080000 16.6776140000
C 9.0802060000 2.6958850000 19.8383780000
H 8.3499150000 1.9773350000 20.2185070000
H 8.5289340000 3.5979170000 19.5654370000
H 9.7542770000 2.9392170000 20.6621820000
C 10.4235580000 0.7554270000 19.0878450000
H 10.9534170000 0.2472010000 18.2836400000
H 9.6300240000 0.0846610000 19.4266770000
H 11.1230940000 0.8852870000 19.9146450000
C 6.9302490000 5.2021900000 17.0554770000
C 6.2589930000 4.5575190000 18.0268350000
C 6.9248860000 4.6637500000 15.6480110000
H 6.4576760000 3.6823770000 15.5043810000
H 6.4098110000 5.3557370000 14.9739520000
H 7.9404060000 4.5958100000 15.2443620000
C 5.4458990000 3.2993980000 17.9687380000
H 4.3875220000 3.4956700000 18.1669100000
H 5.5021150000 2.7910020000 17.0073880000
H 5.7649220000 2.5873750000 18.7353070000
H 6.2808520000 4.9787470000 19.0355470000
H 9.1885590000 7.7332210000 16.1540610000

H 9.2346930000 9.7204950000 19.2871830000

TS_6

Mg 15.3372380000 6.7656270000 3.9586470000
P 11.3617080000 3.9636860000 2.7852630000
P 17.7932310000 5.8870700000 3.3533930000
N 15.0428820000 4.6791710000 4.1834230000
H 14.3428200000 7.8811430000 3.0504700000
C 10.0868760000 6.5083070000 3.4202400000
C 8.7437960000 4.7609070000 2.2519420000
C 9.4150790000 4.4399510000 4.6154390000
C 9.8582950000 5.0076650000 3.2640060000
C 11.8185040000 5.8499310000 0.6130260000
C 11.1145590000 3.5190340000 0.0592830000
C 11.9140010000 4.3886190000 1.0284230000
C 18.8793700000 3.3863830000 2.4730970000
C 19.0080560000 5.2993510000 0.8670840000
C 16.8348240000 4.2461620000 1.3710340000
C 18.1834430000 4.6466650000 1.9712450000
C 19.6946100000 7.8775330000 2.8703460000
C 20.5722090000 5.8630710000 4.0686790000
C 19.0623190000 7.4645940000 5.2101760000
C 19.3678500000 6.7777360000 3.8819900000
C 12.6593240000 4.8280230000 3.7956370000
C 13.8645400000 4.0311640000 4.1956310000
C 13.7615420000 2.7279970000 4.6564510000
C 14.8981120000 2.1013260000 5.1550230000
C 16.0941740000 2.7806820000 5.1965080000
C 16.1611950000 4.0896070000 4.7013240000
C 17.3361050000 4.9290700000 4.8209220000
C 13.3805540000 3.9629670000 0.9624770000
H 10.3958170000 6.9921660000 2.4973390000
H 9.1551130000 6.9847280000 3.7375470000
H 10.8325300000 6.7422020000 4.1798070000
H 8.5837820000 3.6960770000 2.0727830000
H 7.8037800000 5.1744100000 2.6255790000
H 8.9448320000 5.2385240000 1.2934610000
H 10.1850540000 4.5445450000 5.3824770000
H 8.5320960000 4.9751090000 4.9732480000
H 9.1602330000 3.3821910000 4.5459030000
H 12.3449400000 6.5161730000 1.2980650000
H 12.2777370000 5.9809380000 -0.3710360000
H 10.7873000000 6.1909170000 0.5284230000
H 10.0563470000 3.7753410000 0.0413190000

H 11.4961310000 3.6482060000 -0.9567110000
H 11.1946190000 2.4608220000 0.3109650000
H 18.2883760000 2.8758410000 3.2335890000
H 19.0087100000 2.6878160000 1.6421930000
H 19.8661990000 3.5791650000 2.8880140000
H 20.0341730000 5.5001150000 1.1703180000
H 19.0551640000 4.6257410000 0.0079380000
H 18.5639380000 6.2336820000 0.5205520000
H 16.2763910000 5.1130110000 1.0153280000
H 16.9964850000 3.5844590000 0.5160150000
H 16.2098910000 3.7060270000 2.0803570000
H 18.8426490000 8.5393060000 2.7098330000
H 20.5165520000 8.4870410000 3.2529500000
H 20.0048670000 7.4934720000 1.9023510000
H 20.9257140000 5.4386440000 3.1306450000
H 21.4008060000 6.4373180000 4.4911060000
H 20.3661310000 5.0429830000 4.7572140000
H 18.8572130000 6.7599440000 6.0145860000
H 19.9232850000 8.0676770000 5.5073750000
H 18.2118670000 8.1380760000 5.1220710000
H 12.1557060000 5.1223580000 4.7214620000
H 12.9636330000 5.7634560000 3.3169630000
H 12.8045760000 2.2254650000 4.6248520000
H 14.8387040000 1.0833310000 5.5204790000
H 16.9873030000 2.3240480000 5.6022440000
H 18.1600960000 4.4324500000 5.3331560000
H 13.5375520000 2.9476760000 1.3345530000
H 13.7312430000 3.9921990000 -0.0721650000
H 14.0175740000 4.6353660000 1.5366750000
H 16.7045140000 5.9982280000 5.5385830000
C 15.7496150000 6.9559140000 6.1822270000
C 14.9752840000 6.2284460000 7.0076940000
C 16.0892750000 8.3832250000 6.5369050000
H 17.0099410000 8.4431080000 7.1243020000
H 16.2531550000 9.0109590000 5.6572260000
H 15.3114250000 8.8775570000 7.1236790000
C 14.3588640000 6.6186190000 8.3068830000
H 14.7850400000 6.0349210000 9.1278200000
H 14.4936890000 7.6701140000 8.5517830000
H 13.2874070000 6.4049560000 8.3164550000
H 14.7821860000 5.1884930000 6.7377300000

TS$_7$

P 7.5136220000 8.9631810000 18.9296220000

P 11.9038660000 3.5577020000 18.4786120000
Mg 8.5400250000 7.0069820000 17.7435020000
N 9.9055590000 7.0143680000 19.2795310000
C 8.7723430000 8.9910760000 20.1399880000
H 8.7612800000 9.7341690000 20.9304940000
C 9.7996890000 8.0435250000 20.2034000000
C 10.7894570000 8.1108360000 21.2290040000
H 10.7036770000 8.8955960000 21.9694440000
C 11.8173820000 7.2200300000 21.2646660000
H 12.5643030000 7.2891200000 22.0471160000
C 11.9191770000 6.2100470000 20.2924780000
H 12.7259950000 5.4921270000 20.2986380000
C 10.9439590000 6.1442450000 19.3274480000
C 10.9733460000 5.1397840000 18.2067210000
H 11.4418060000 5.6201850000 17.3417680000
H 9.9517000000 4.9015130000 17.8821710000
C 7.4911980000 10.6535840000 18.1064700000
C 7.6024600000 11.7971040000 19.1084420000
H 6.7426290000 11.8611060000 19.7725250000
H 7.6715810000 12.7481480000 18.5744980000
H 8.4986570000 11.7033810000 19.7215320000
C 6.2553550000 10.8328380000 17.2338350000
H 6.1192830000 10.0002840000 16.5403580000
H 6.3552680000 11.7409640000 16.6344370000
H 5.3436760000 10.9367770000 17.8212680000
C 8.7341130000 10.6649920000 17.2144220000
H 9.6412710000 10.4635790000 17.7868760000
H 8.8494050000 11.6448910000 16.7455040000
H 8.6677870000 9.9256050000 16.4134370000
C 5.8670350000 8.5708240000 19.7677820000
C 5.3049330000 9.7140450000 20.6004890000
H 6.0350590000 10.0920670000 21.3175580000
H 4.4392430000 9.3681960000 21.1718800000
H 4.9681400000 10.5481210000 19.9852220000
C 4.8464280000 8.1131560000 18.7275460000
H 4.5624300000 8.9010020000 18.0326750000
H 3.9328240000 7.7797330000 19.2267220000
H 5.2235160000 7.2709800000 18.1433970000
C 6.1737150000 7.3875800000 20.6868340000
H 6.6887980000 6.5819280000 20.1561660000
H 5.2421470000 6.9762050000 21.0830240000
H 6.8053410000 7.6767170000 21.5251270000
C 12.2147900000 3.0858520000 16.6710010000
C 13.4331670000 3.9206740000 16.2660990000

H 13.2426100000 4.9931590000 16.3322770000
H 13.7032220000 3.7061300000 15.2291760000
H 14.2991260000 3.7005720000 16.8905750000
C 11.0867710000 3.3691240000 15.6820690000
H 10.1787170000 2.8106480000 15.8960460000
H 11.4085980000 3.0841940000 14.6764390000
H 10.8250230000 4.4264890000 15.6371680000
C 12.6034490000 1.6123770000 16.5993600000
H 13.3764630000 1.3593600000 17.3272500000
H 13.0005510000 1.3812870000 15.6077700000
H 11.7536360000 0.9509310000 16.7653870000
C 10.5612910000 2.4394990000 19.2089410000
C 9.4236900000 2.0417010000 18.2788980000
H 8.9443230000 2.9016090000 17.8095300000
H 8.6514770000 1.5145980000 18.8474200000
H 9.7512390000 1.3667590000 17.4891090000
C 9.9779410000 3.2020480000 20.4000290000
H 9.3146430000 2.5426670000 20.9654270000
H 9.3867720000 4.0671740000 20.0940590000
H 10.7514220000 3.5566670000 21.0828460000
C 11.2463160000 1.1828730000 19.7458970000
H 11.6794560000 0.5720080000 18.9552900000
H 10.5203260000 0.5606430000 20.2755070000
H 12.0438640000 1.4309720000 20.4470610000
C 7.1839720000 5.5371670000 16.7391550000
C 6.9961550000 4.8437320000 17.8738630000
C 6.1394310000 5.7031040000 15.6887980000
H 5.1469290000 5.3266090000 15.9500870000
H 6.0261570000 6.7568440000 15.4138560000
H 6.4414070000 5.1987770000 14.7667690000
C 5.7901810000 4.0982030000 18.3463640000
H 6.0125680000 3.0342670000 18.4610170000
H 5.4657720000 4.4483810000 19.3290190000
H 4.9466410000 4.1869850000 17.6644880000
H 7.8261060000 4.7799340000 18.5921740000
H 8.4803240000 6.1653340000 16.1644510000
H 9.2698950000 6.7635720000 15.9764060000

Butyne

C 1.0256560000 -0.0459660000 -0.0471880000
C -0.1780400000 -0.0459660000 -0.0471880000
C 2.4703750000 -0.0454310000 -0.0469030000
C -1.6227580000 -0.0454310000 -0.0469030000
H 2.8699020000 0.9128490000 -0.3816600000

H 2.8708170000 -0.8141960000 -0.7092090000
H 2.8703040000 -0.2342550000 0.9502860000
H -2.0222850000 0.9128490000 -0.3816610000
H -2.0226870000 -0.2342540000 0.9502860000
H -2.0232000000 -0.8141970000 -0.7092080000

Butene
C 1.0926620000 0.0811750000 0.1473340000
C 0.4271510000 0.6374720000 1.1569820000
C 0.9945110000 1.3002830000 2.3594870000
C 2.5654870000 -0.0131310000 -0.0236600000
H 0.5050340000 -0.3597930000 -0.6530010000
H -0.6580300000 0.6123960000 1.1114540000
H 2.0825440000 1.3063550000 2.3700130000
H 0.6581360000 0.8090990000 3.2753250000
H 0.6578260000 2.3367910000 2.4333910000
H 3.1202710000 0.4383780000 0.7963730000
H 2.8865640000 0.4740130000 -0.9471230000
H 2.8864370000 -1.0540050000 -0.1057960000

H_2
H 1.2211930000 0.0262130000 0.0242980000
H 1.9655950000 0.0262130000 0.0242980000

8 NMR spectra

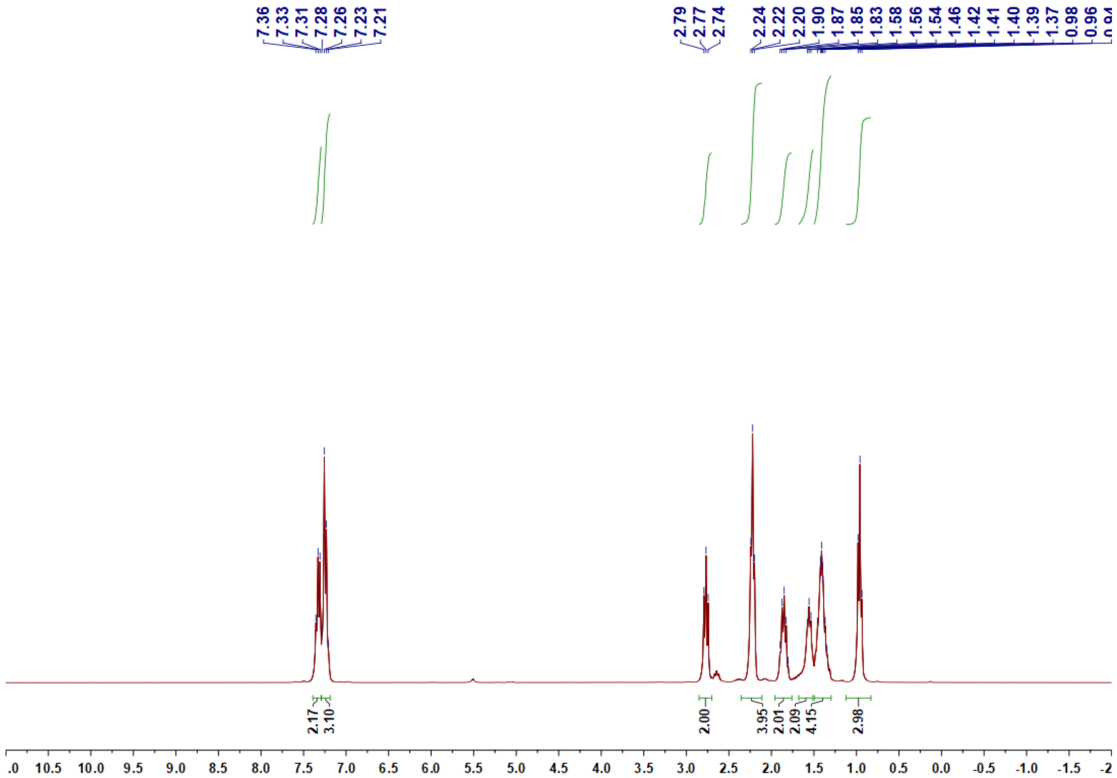

Figure S78. ^{1}H NMR (300 MHz, $CDCl_3$) spectrum of **1o**

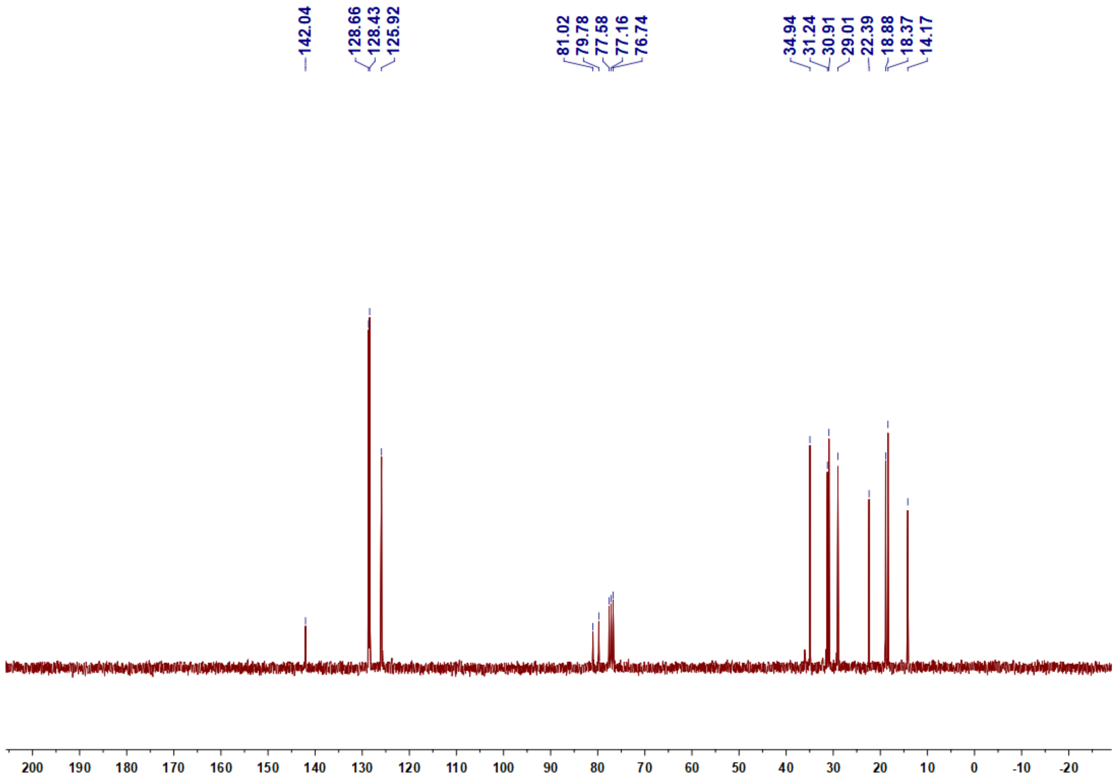

Figure S79. ^{13}C NMR (75 MHz, $CDCl_3$) spectrum of **1o**

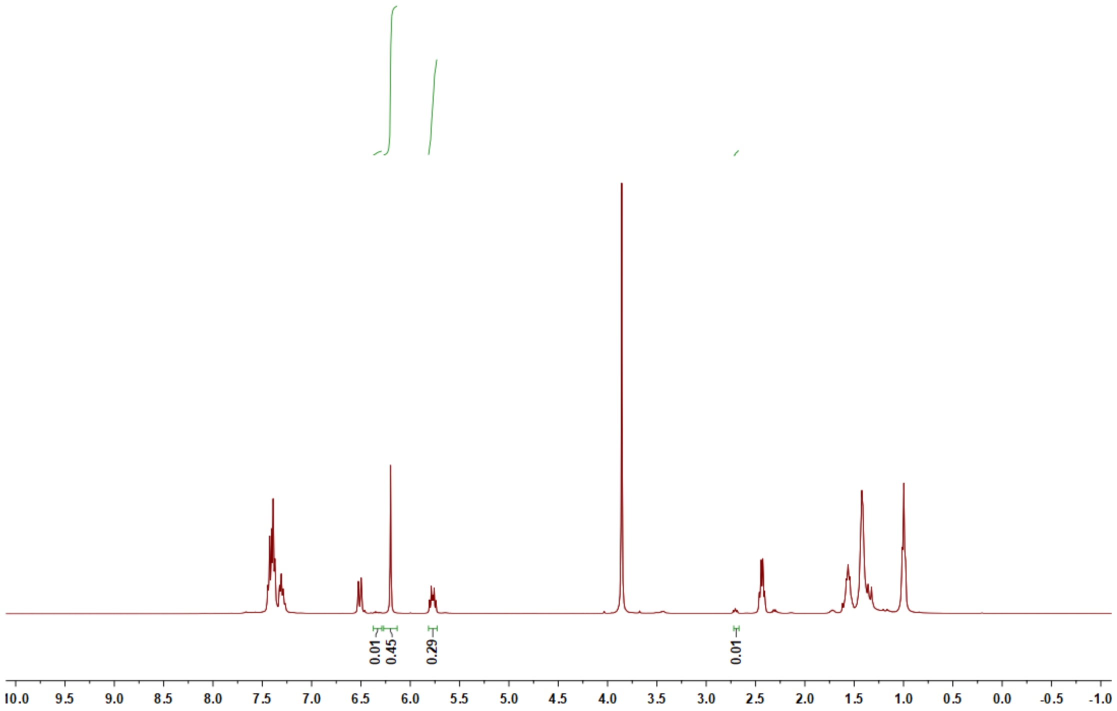

Figure S80. ^{1}H NMR (400 MHz, $CDCl_3$) spectrum of **2a**

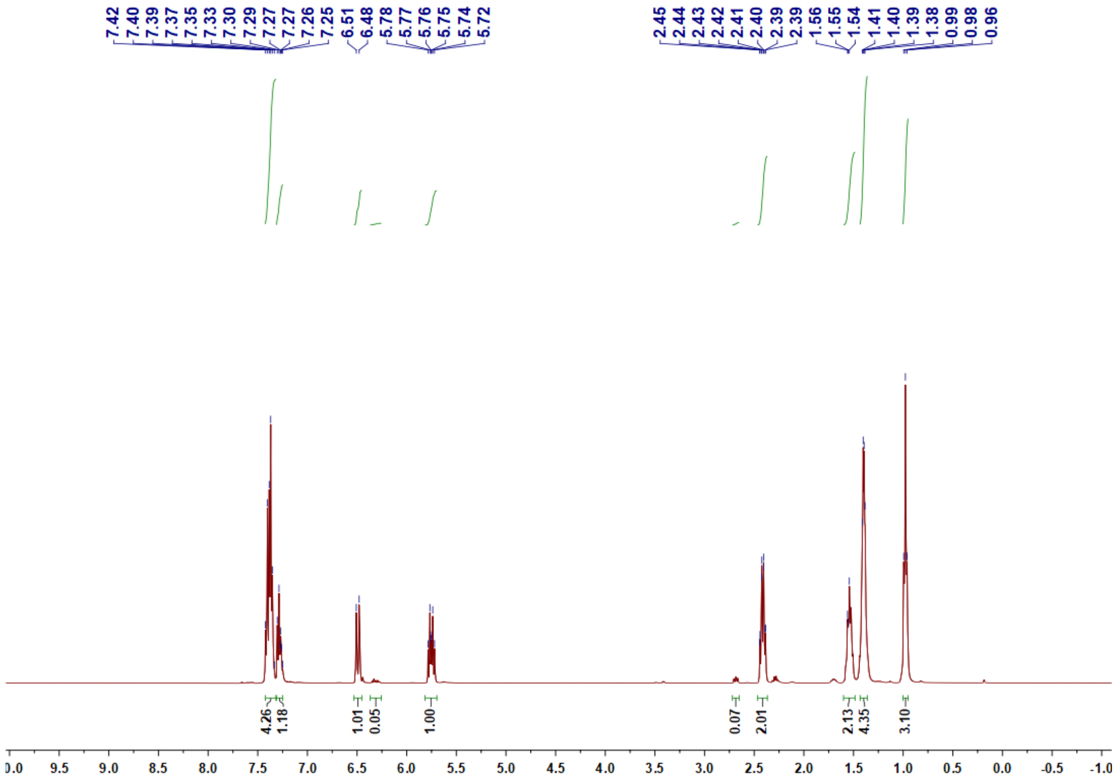

Figure S81. ^{1}H NMR (400 MHz, $CDCl_3$) spectrum of isolated **2a**

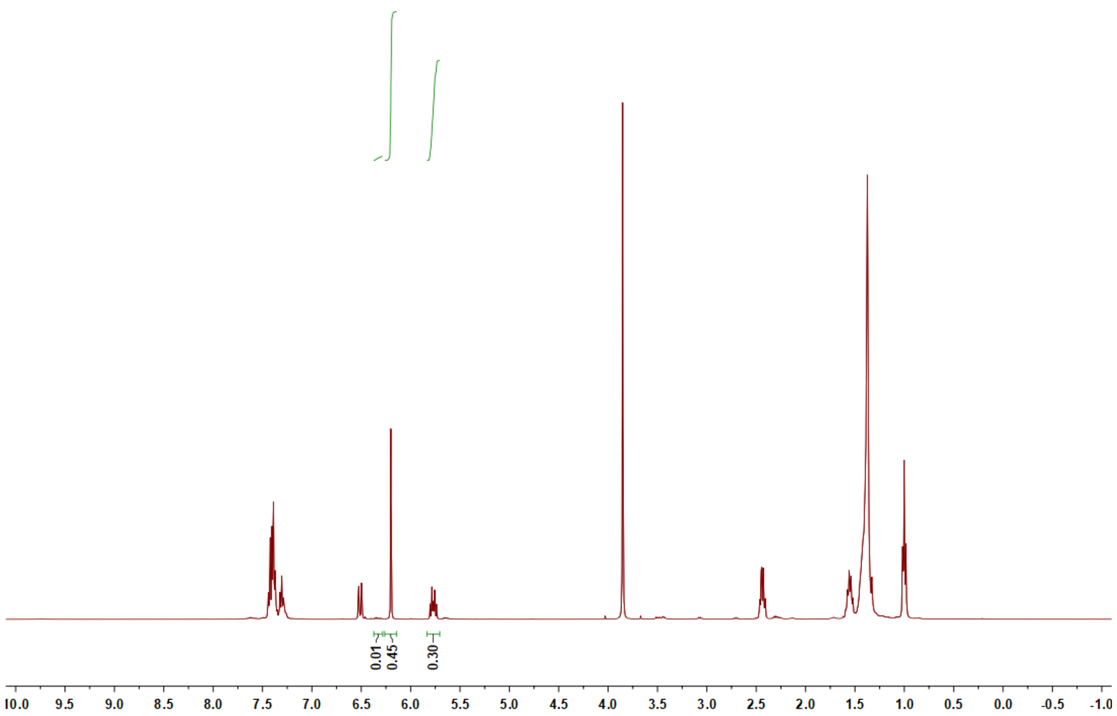

Figure S82. ^{1}H NMR (400 MHz, $CDCl_3$) spectrum of **2b**

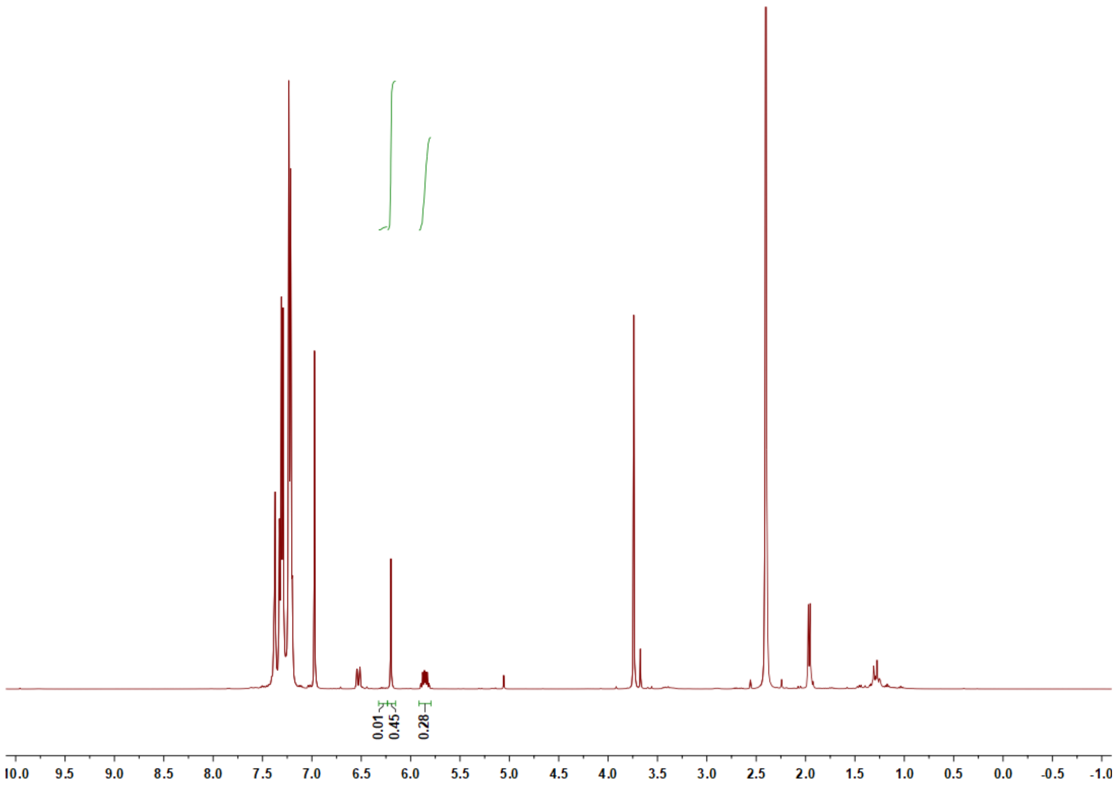

Figure S83. ^{1}H NMR (400 MHz, $CDCl_3$) spectrum of **2c**

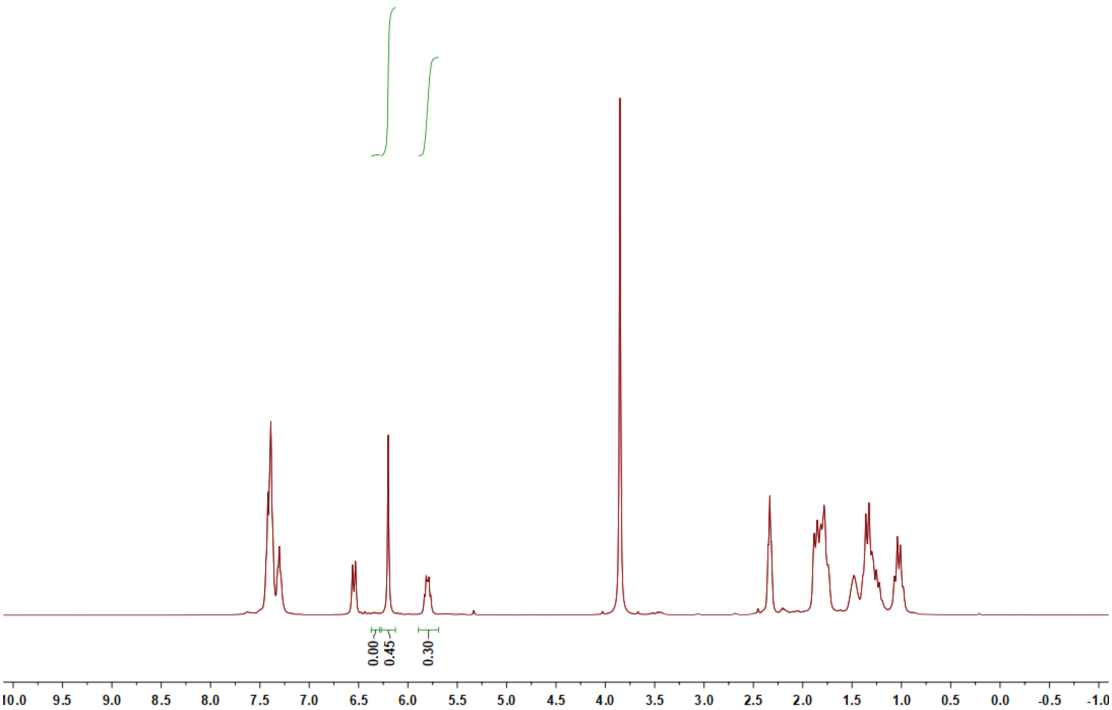

Figure S84. ^{1}H NMR (400 MHz, $CDCl_3$) spectrum of **2d**

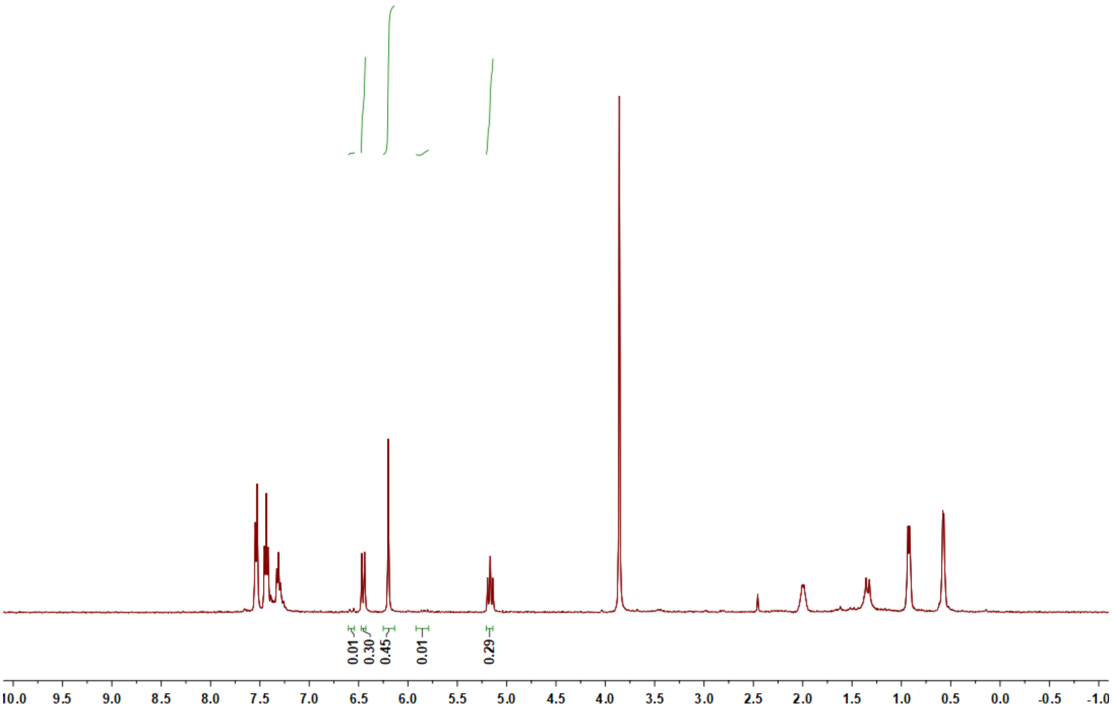

Figure S85. ^{1}H NMR (400 MHz, $CDCl_3$) spectrum of **2e**

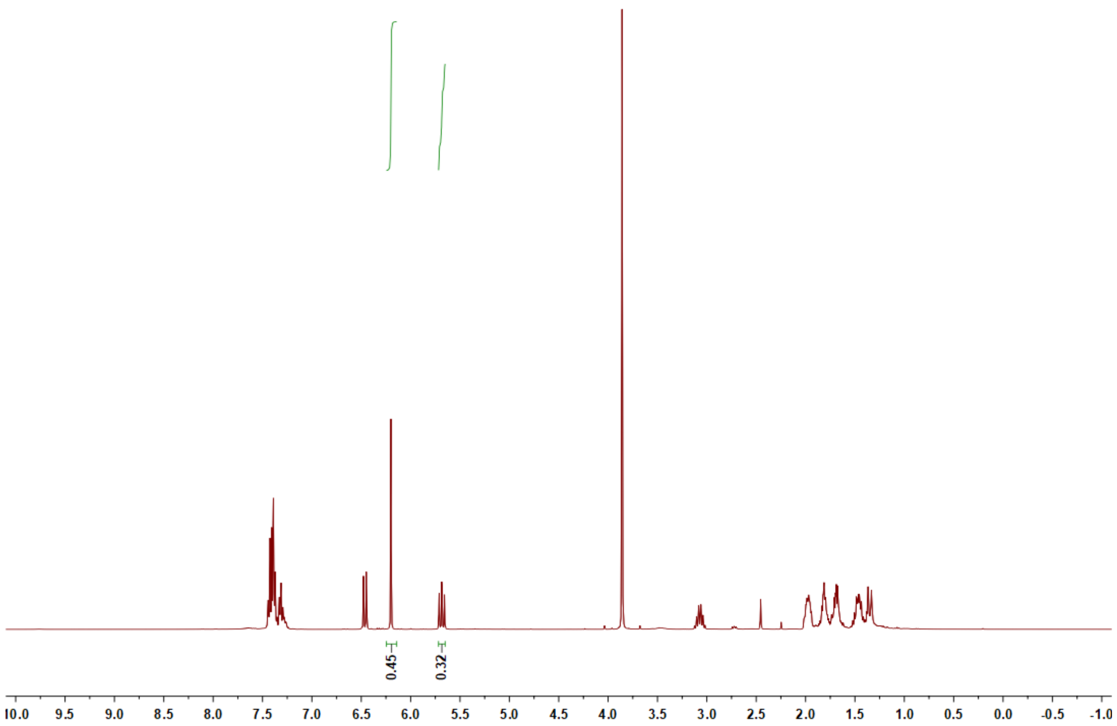

Figure S86. ^{1}H NMR (400 MHz, $CDCl_3$) spectrum of **2f**

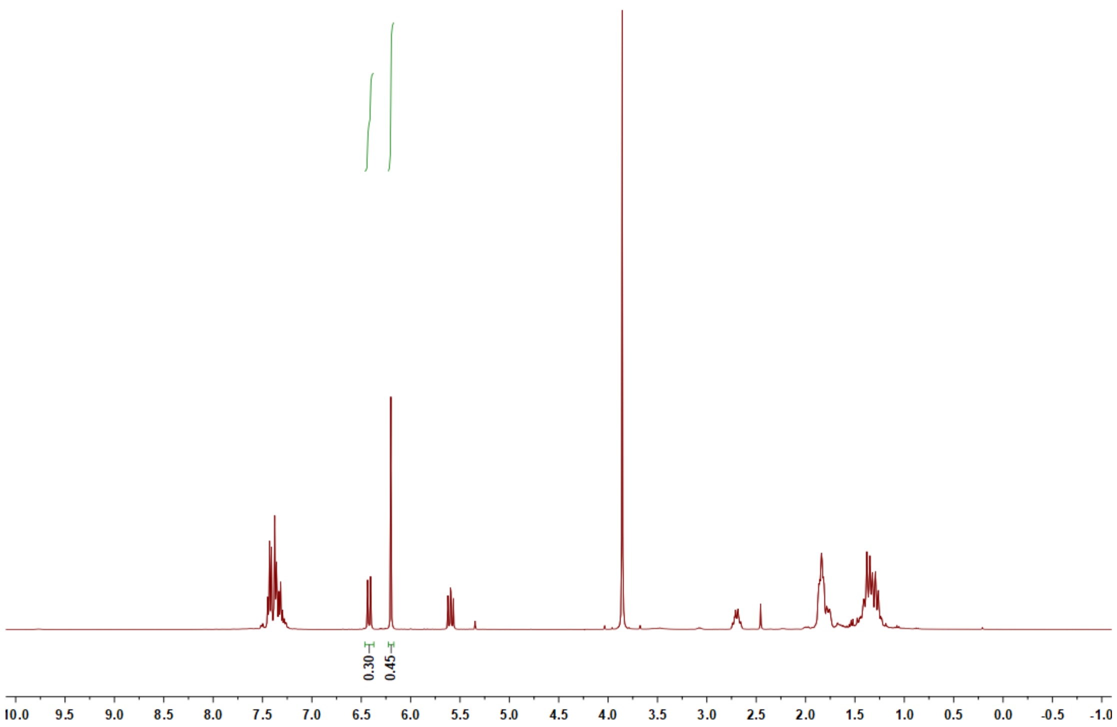

Figure S87. ^{1}H NMR (400 MHz, $CDCl_3$) spectrum of **2g**

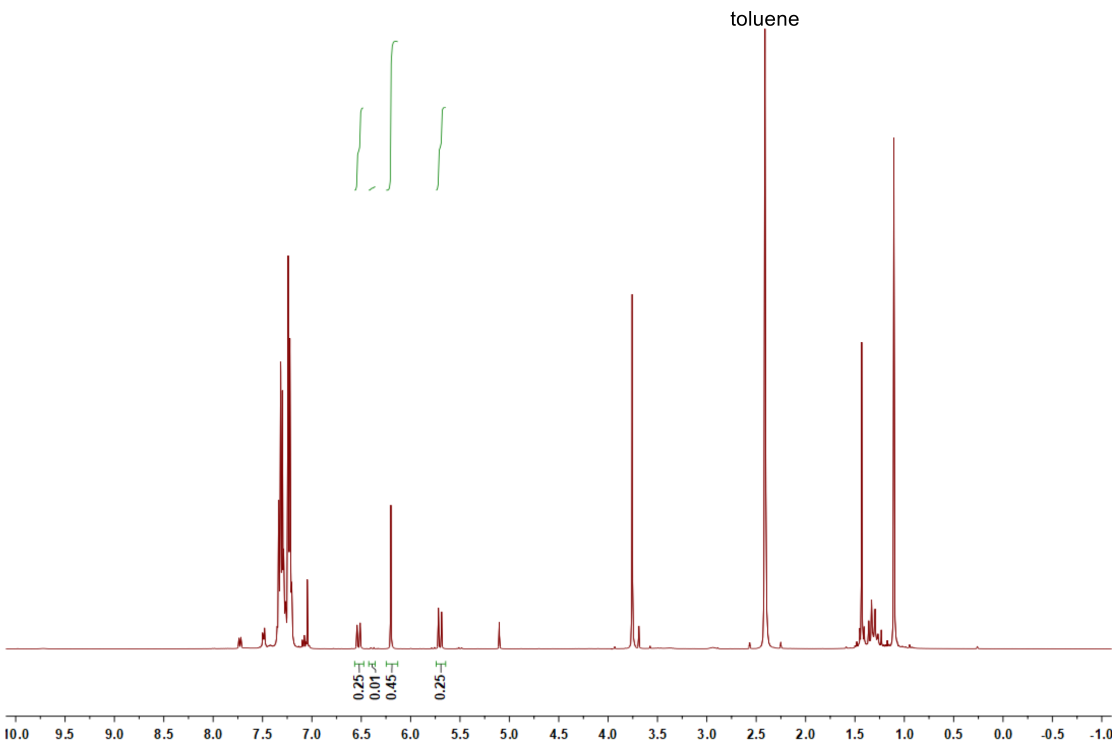

Figure S88. ^{1}H NMR (400 MHz, $CDCl_3$) spectrum of **2h**

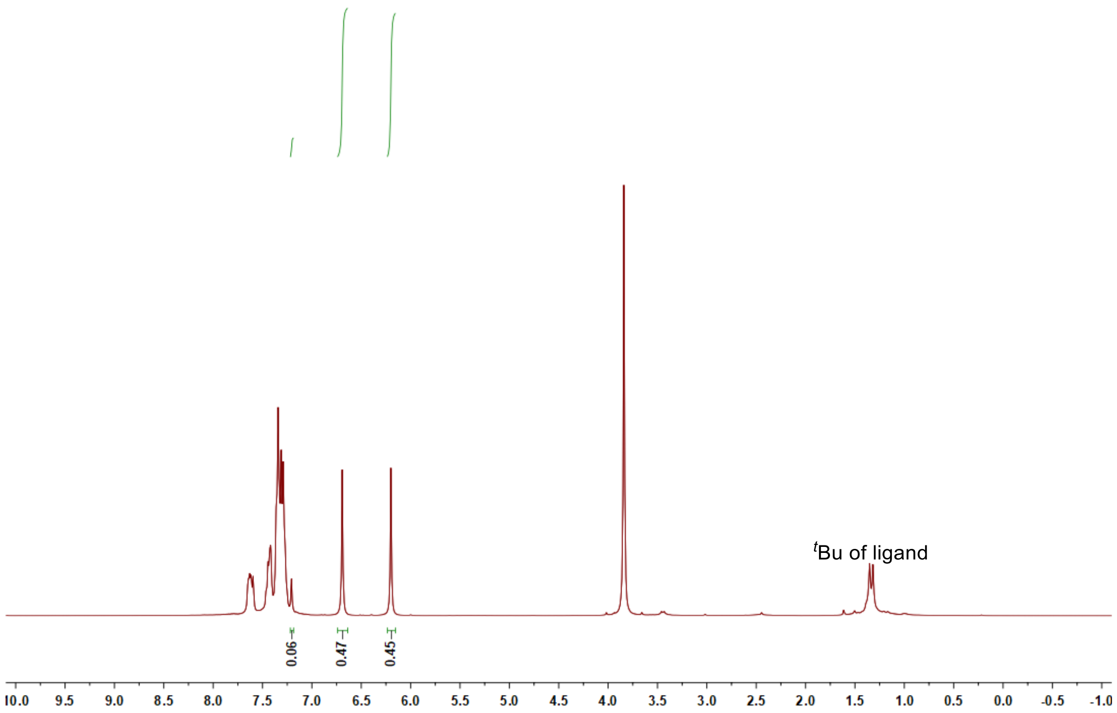

Figure S89. ^{1}H NMR (400 MHz, $CDCl_3$) spectrum of **2i**

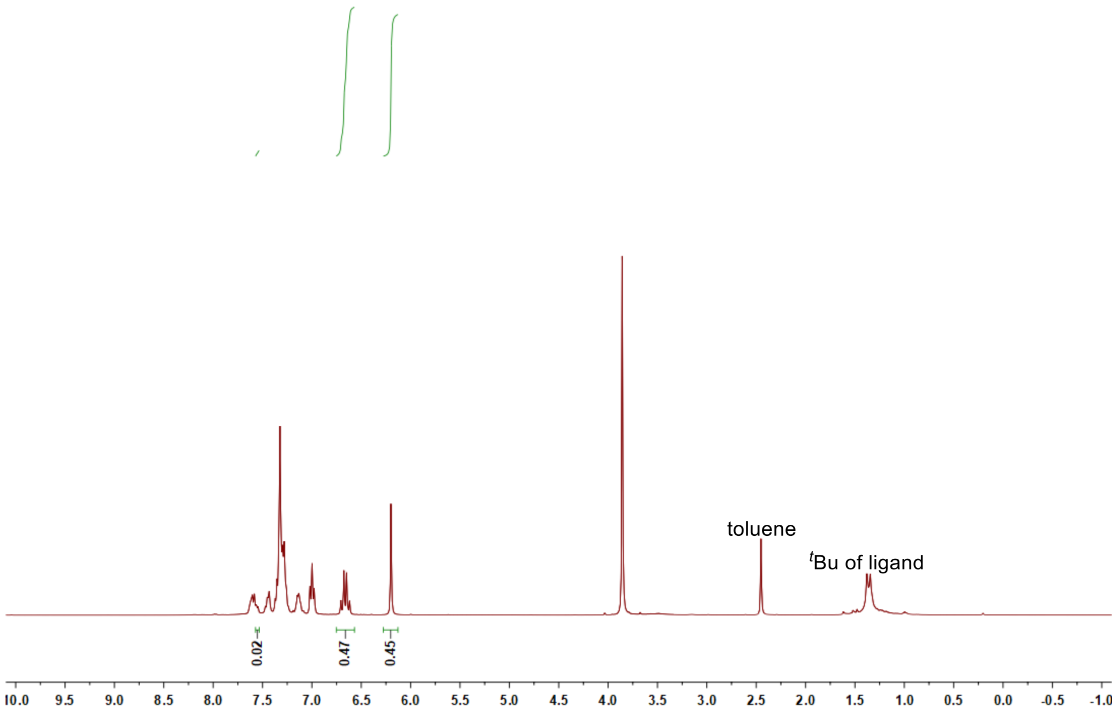

Figure S90. ^{1}H NMR (400 MHz, $CDCl_3$) spectrum of **2j**

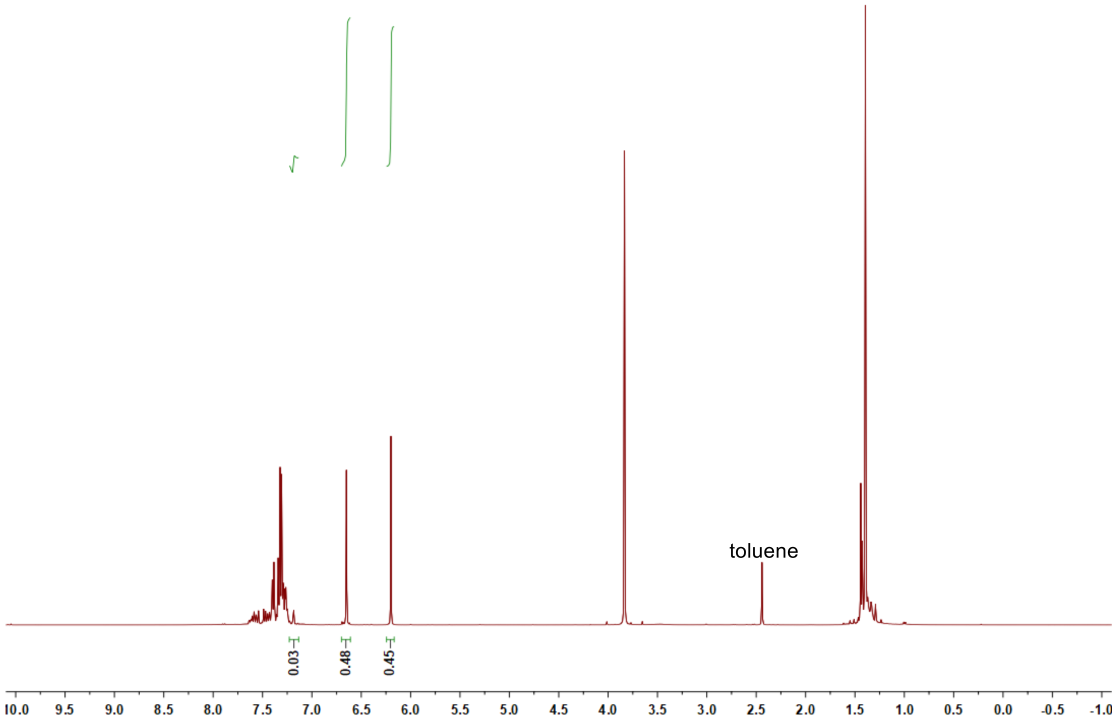

Figure S91. ^{1}H NMR (400 MHz, $CDCl_3$) spectrum of **2k**

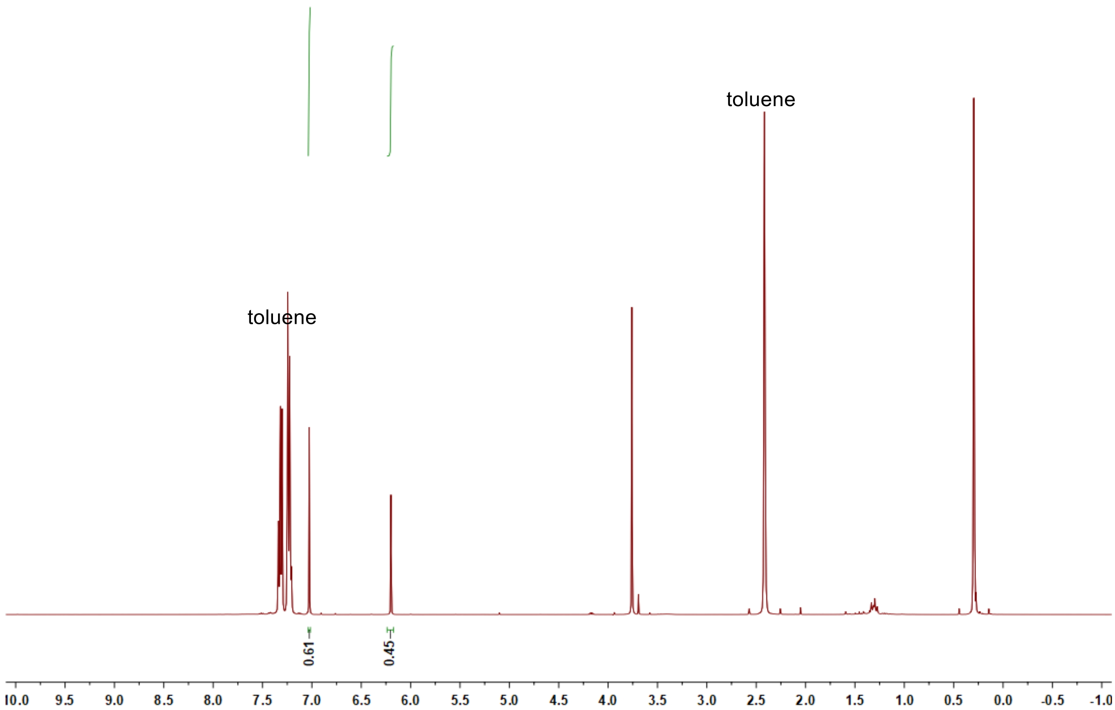

Figure S92. ^{1}H NMR (400 MHz, $CDCl_3$) spectrum of **2l**

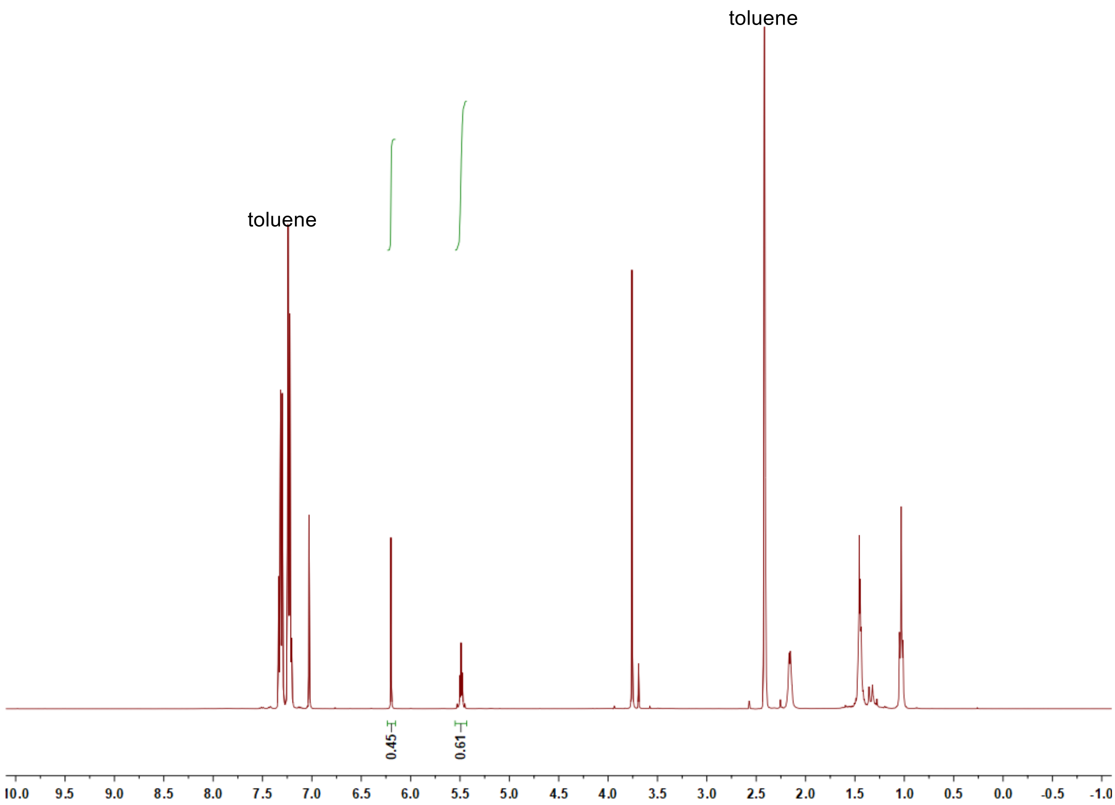

Figure S93. ^{1}H NMR (400 MHz, $CDCl_3$) spectrum of **2m**

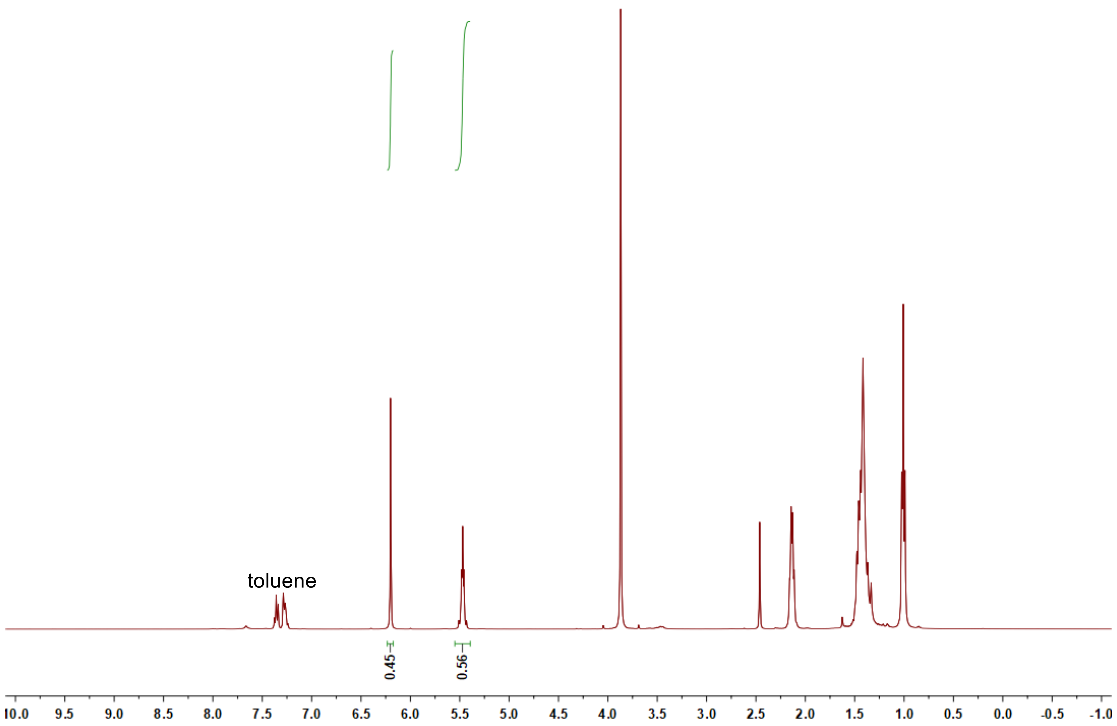

Figure S94. ^{1}H NMR (400 MHz, $CDCl_3$) spectrum of **2n**

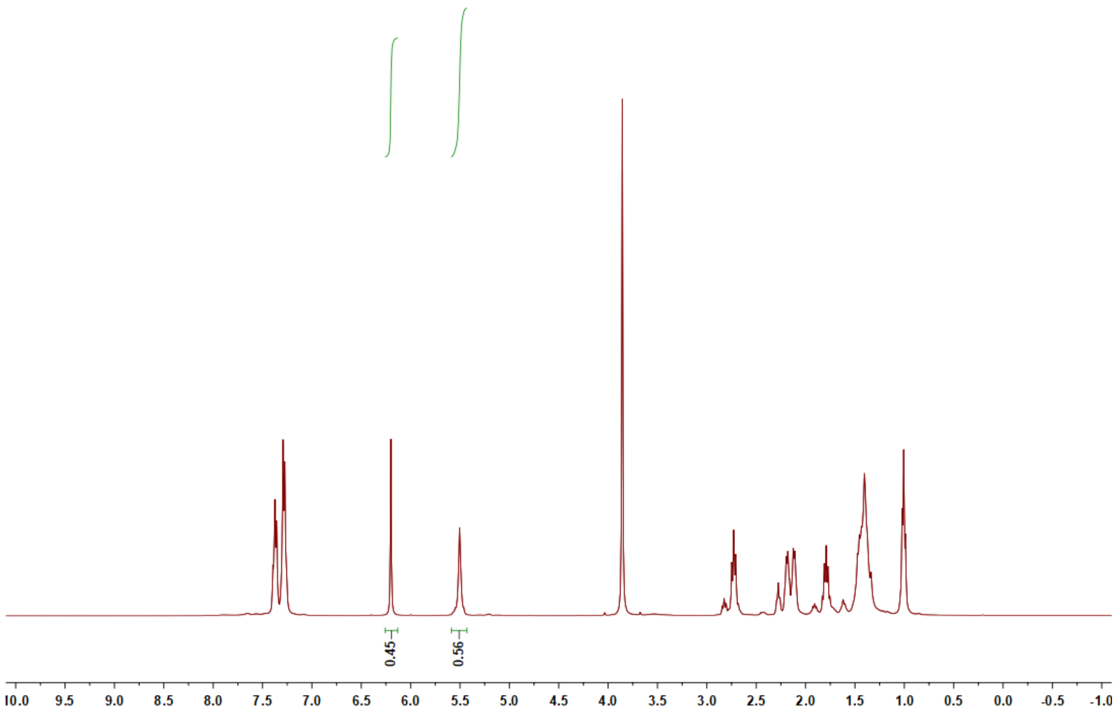

Figure S95. ^{1}H NMR (400 MHz, $CDCl_3$) spectrum of **2o**

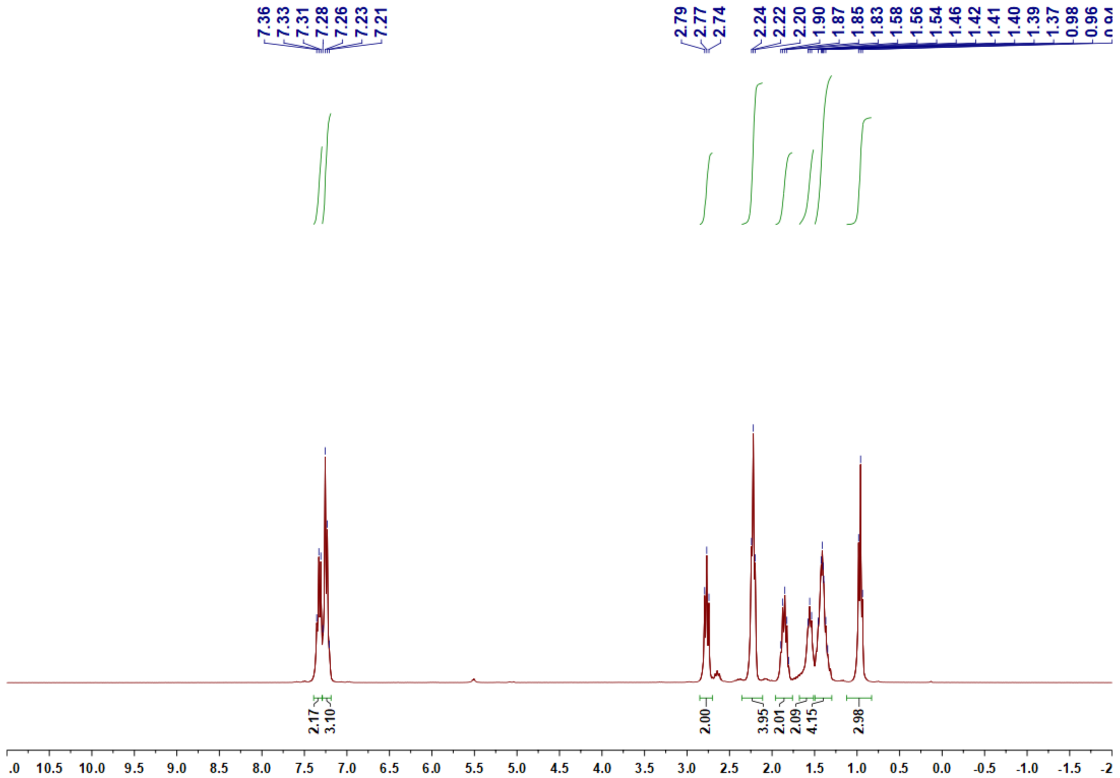

Figure S96. ^{1}H NMR (300 MHz, $CDCl_3$) spectrum of isolated **2o**

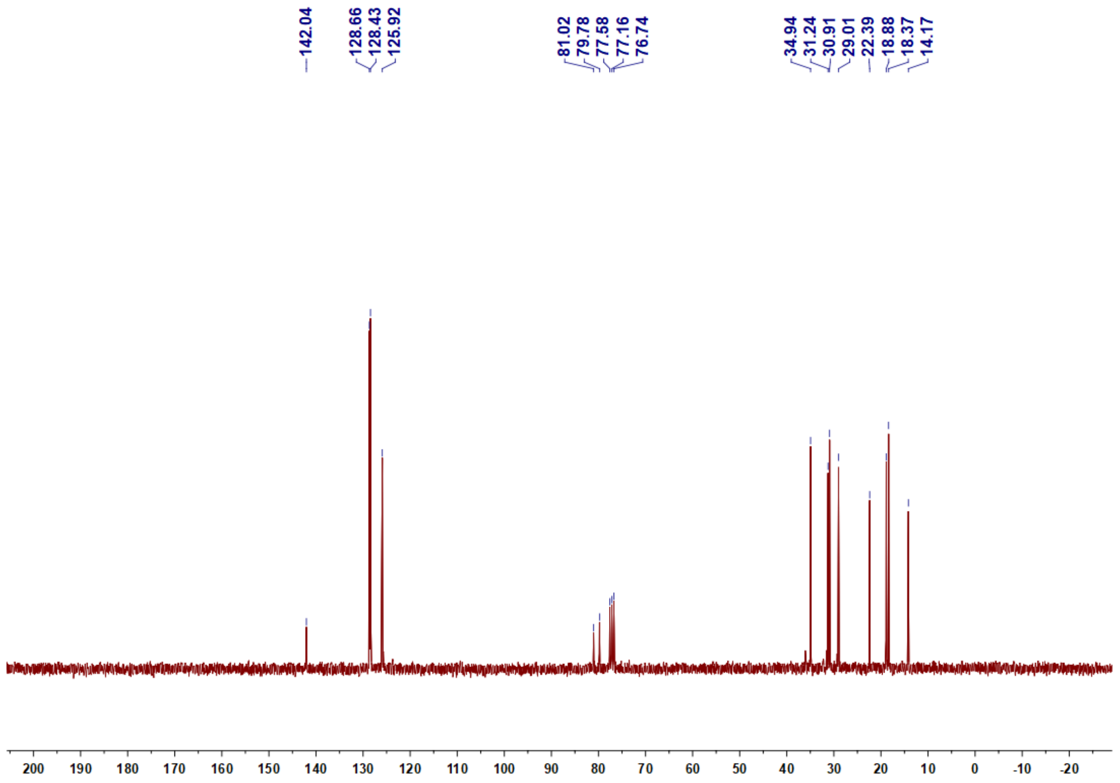

Figure S97. ^{13}C NMR (75 MHz, $CDCl_3$) spectrum of isolated **2o**

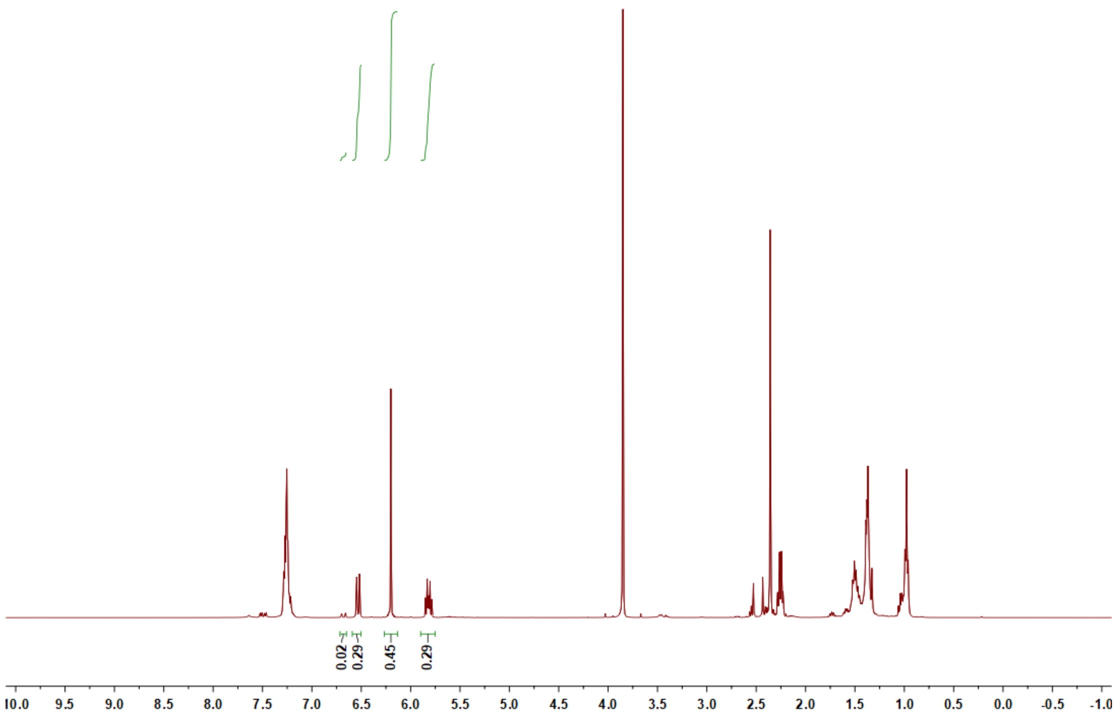

Figure S98. ¹H NMR (400 MHz, $CDCl_3$) spectrum of **2p**

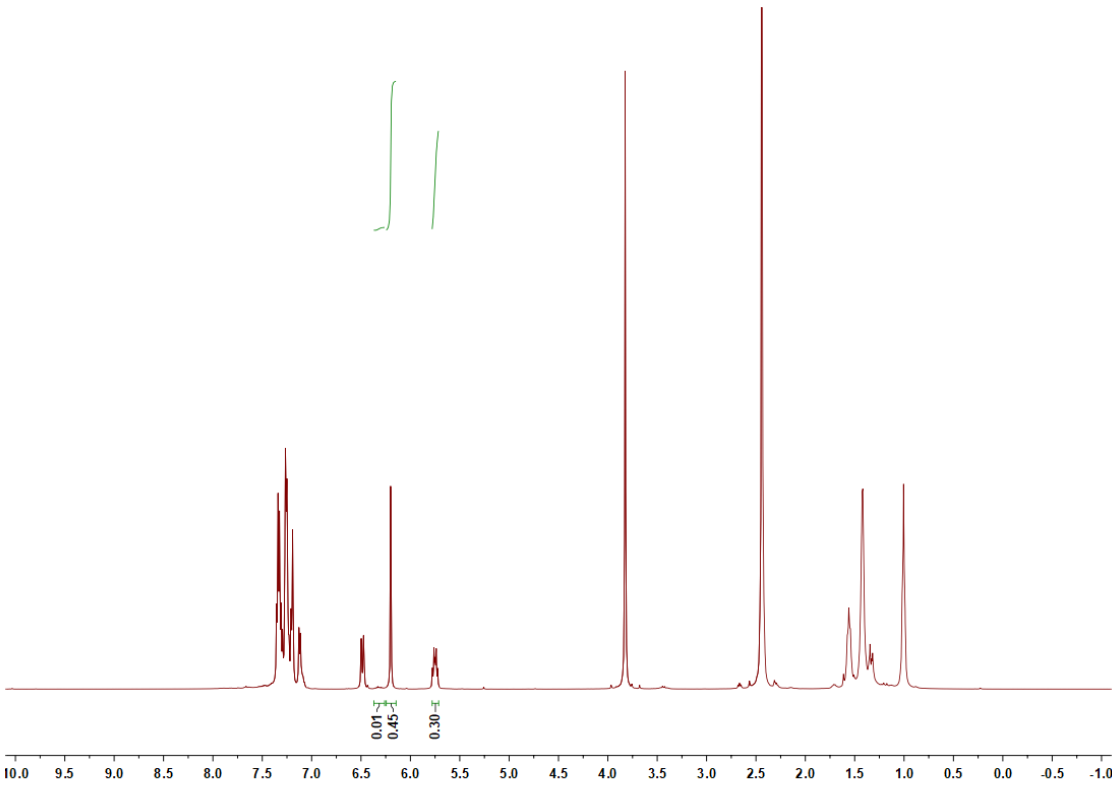

Figure S99. ¹H NMR (400 MHz, $CDCl_3$) spectrum of **2q**

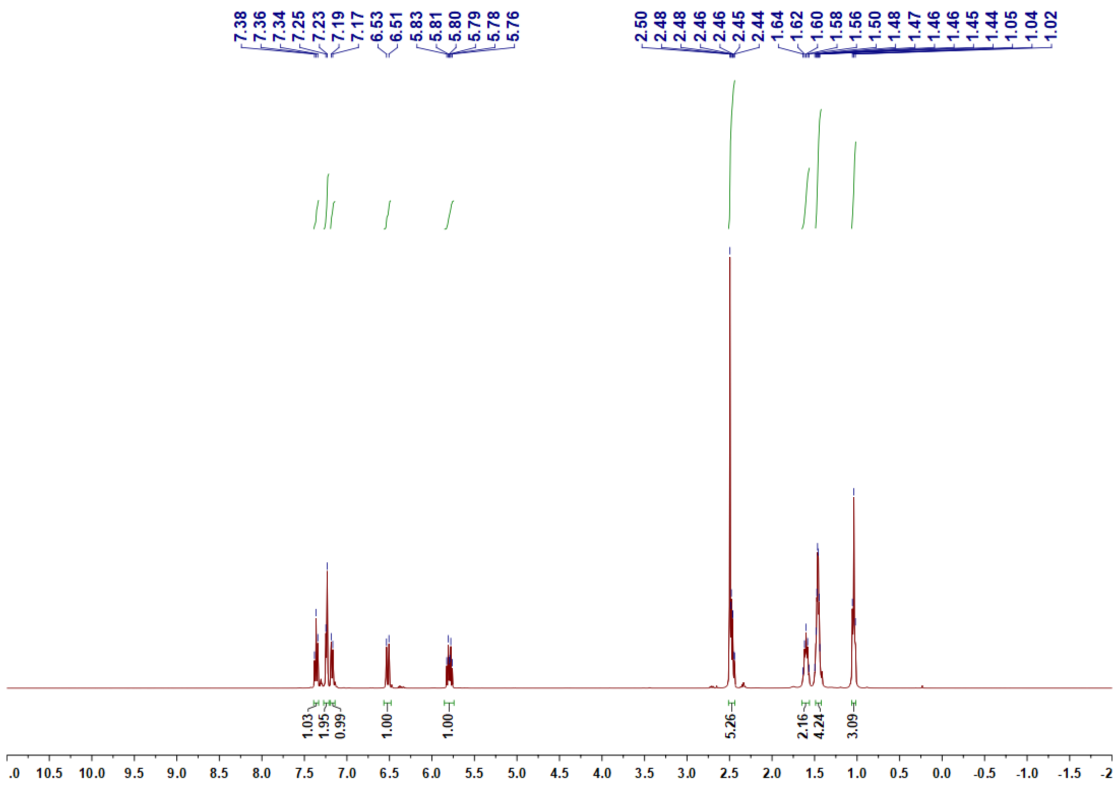

Figure S100. ^{1}H NMR (400 MHz, $CDCl_3$) spectrum of isolated **2q**

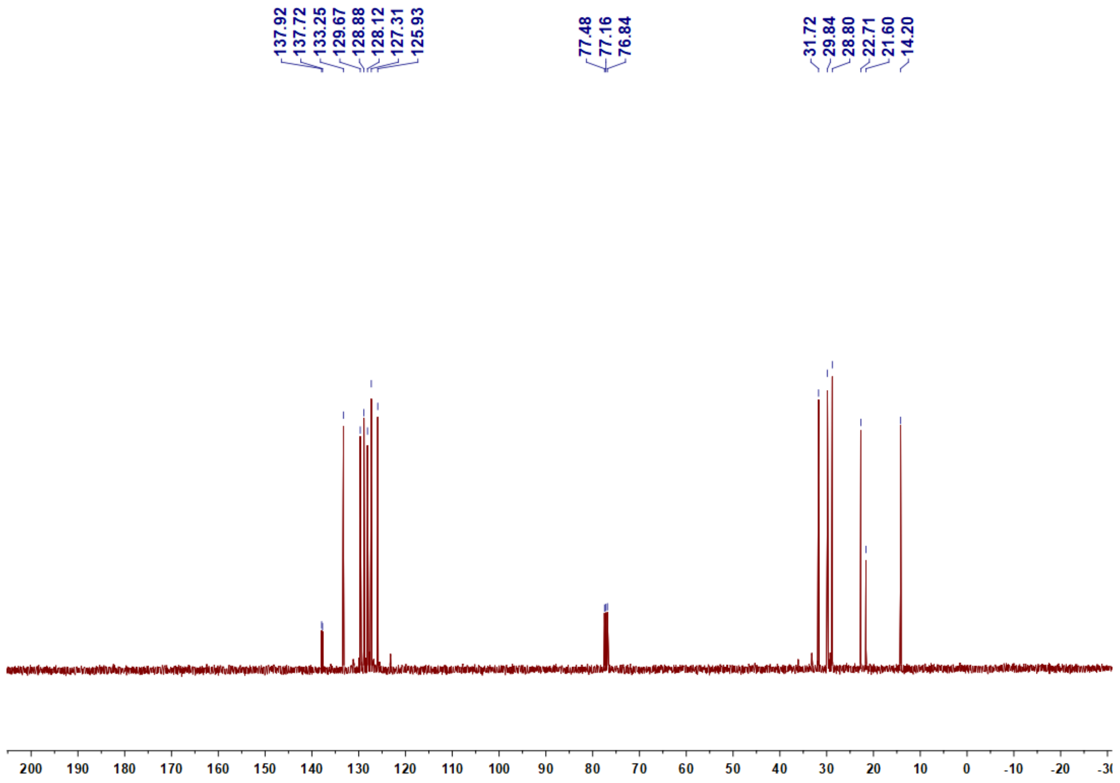

Figure S101. ^{13}C NMR (101 MHz, $CDCl_3$) spectrum of isolated **2q**

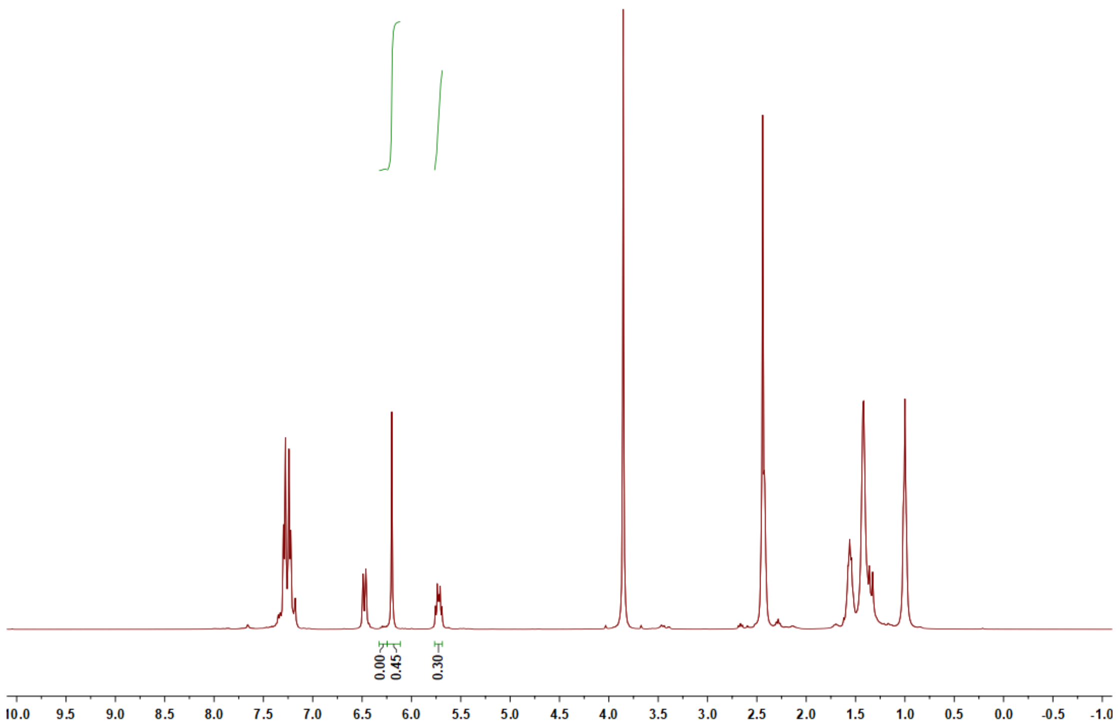

Figure S102. ^{1}H NMR (400 MHz, $CDCl_3$) spectrum of **2r**

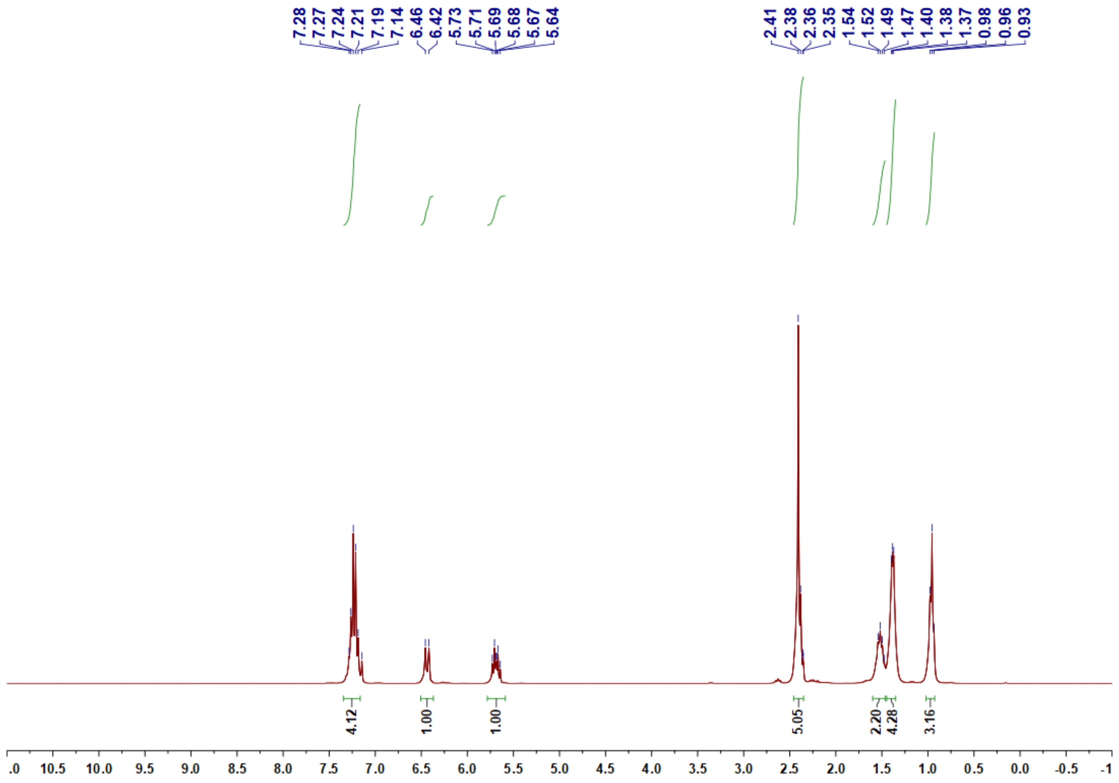

Figure S103. ^{1}H NMR (300 MHz, $CDCl_3$) spectrum of isolated **2r**

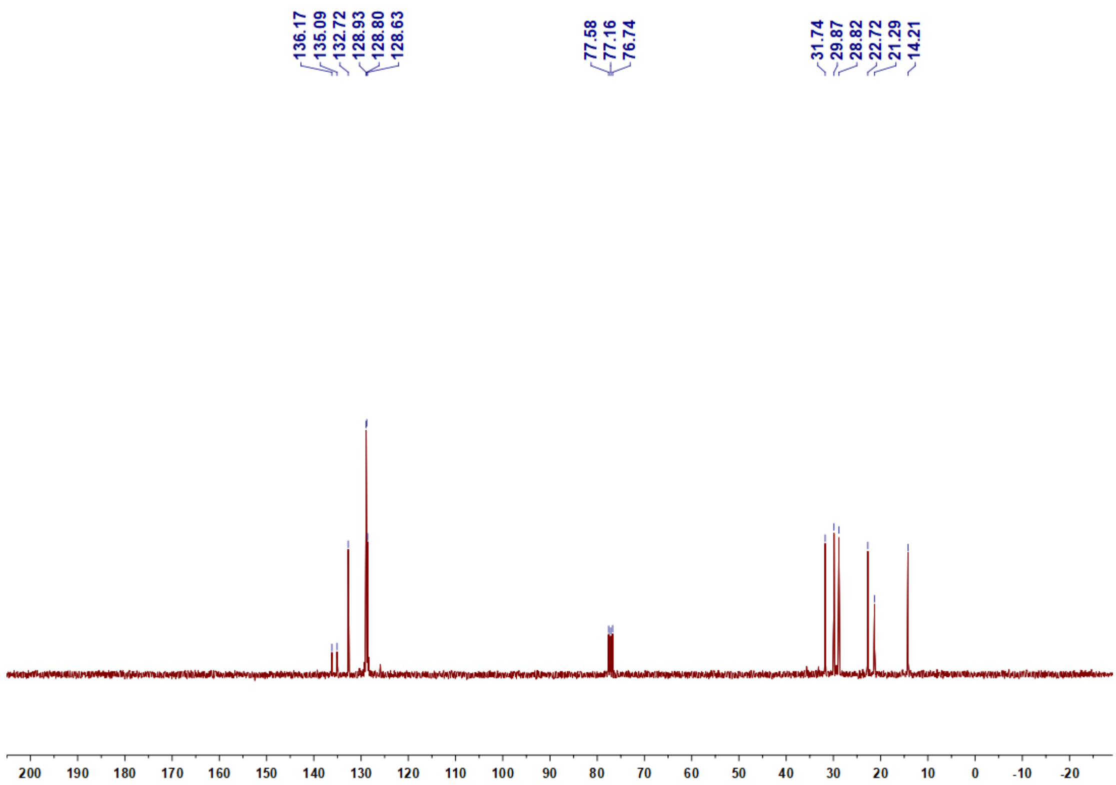

Figure S104. ^{13}C NMR (75 MHz, $CDCl_3$) spectrum of isolated **2r**

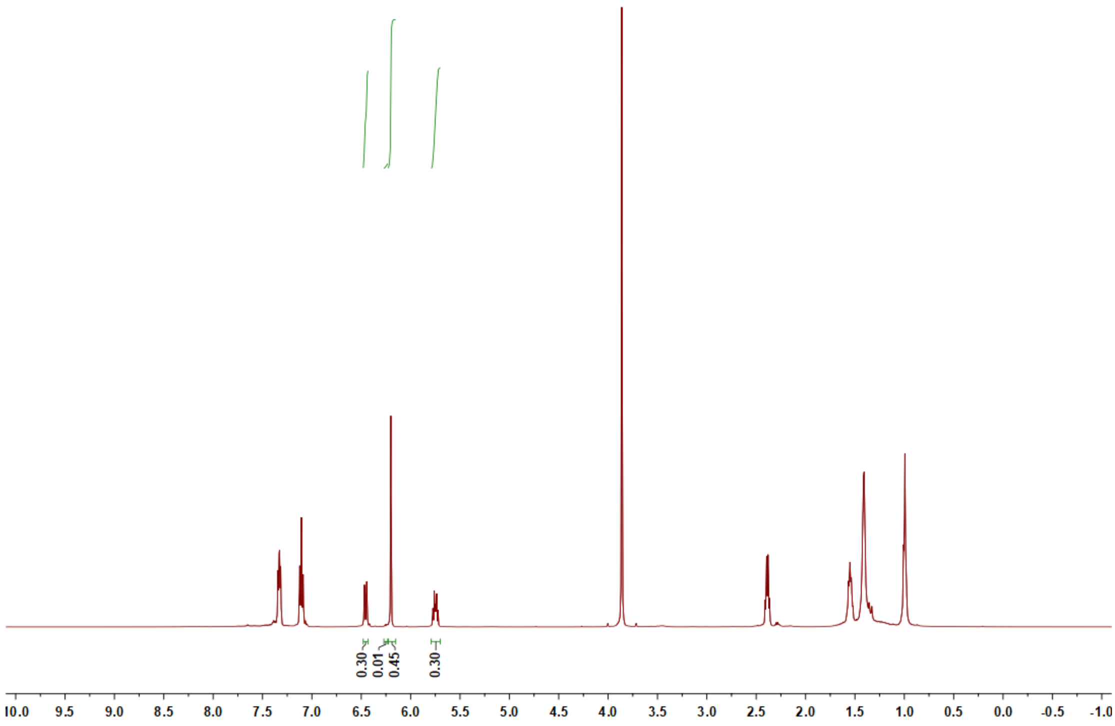

Figure S105. ^{1}H NMR (400 MHz, $CDCl_3$) spectrum of **2s**

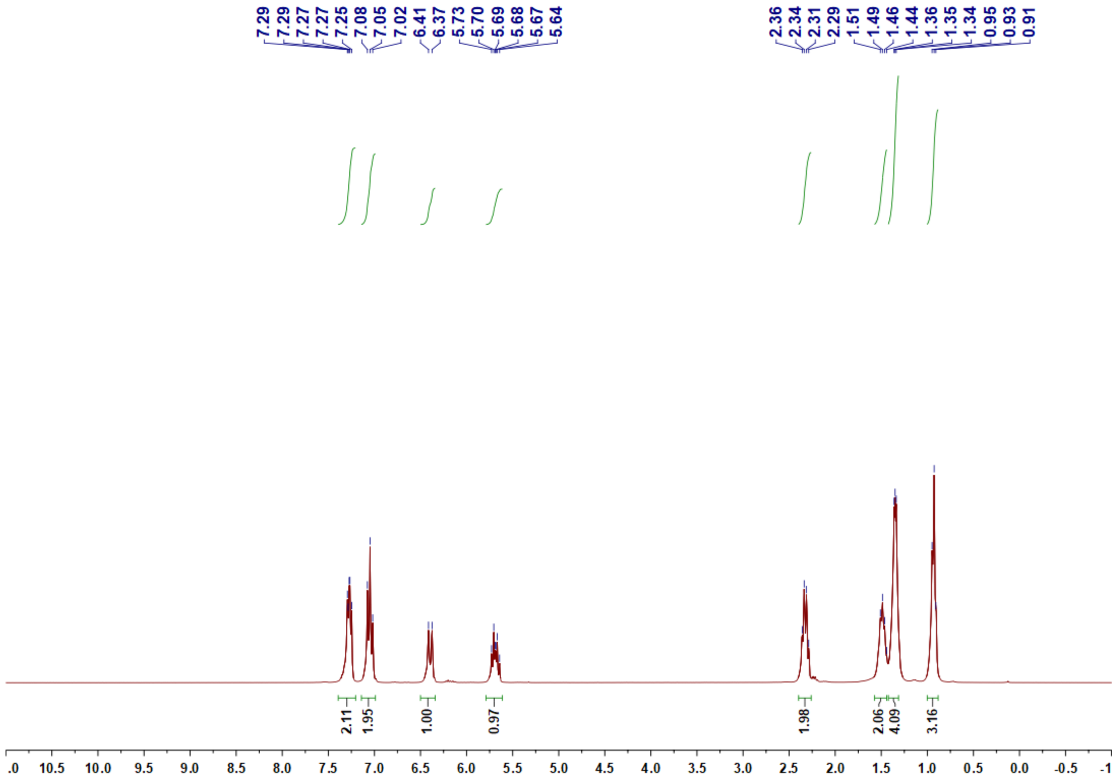

Figure S106. ^{1}H NMR (300 MHz, $CDCl_3$) spectrum of isolated **2s**

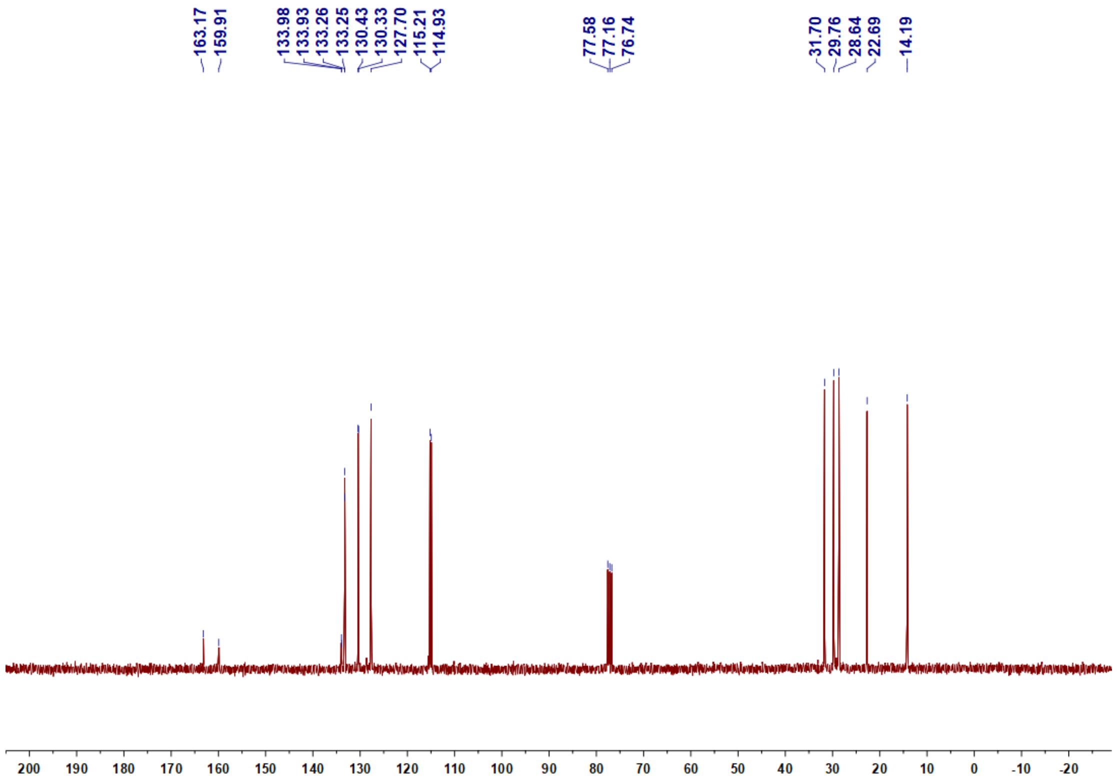

Figure S107. ^{13}C NMR (75 MHz, $CDCl_3$) spectrum of isolated **2s**

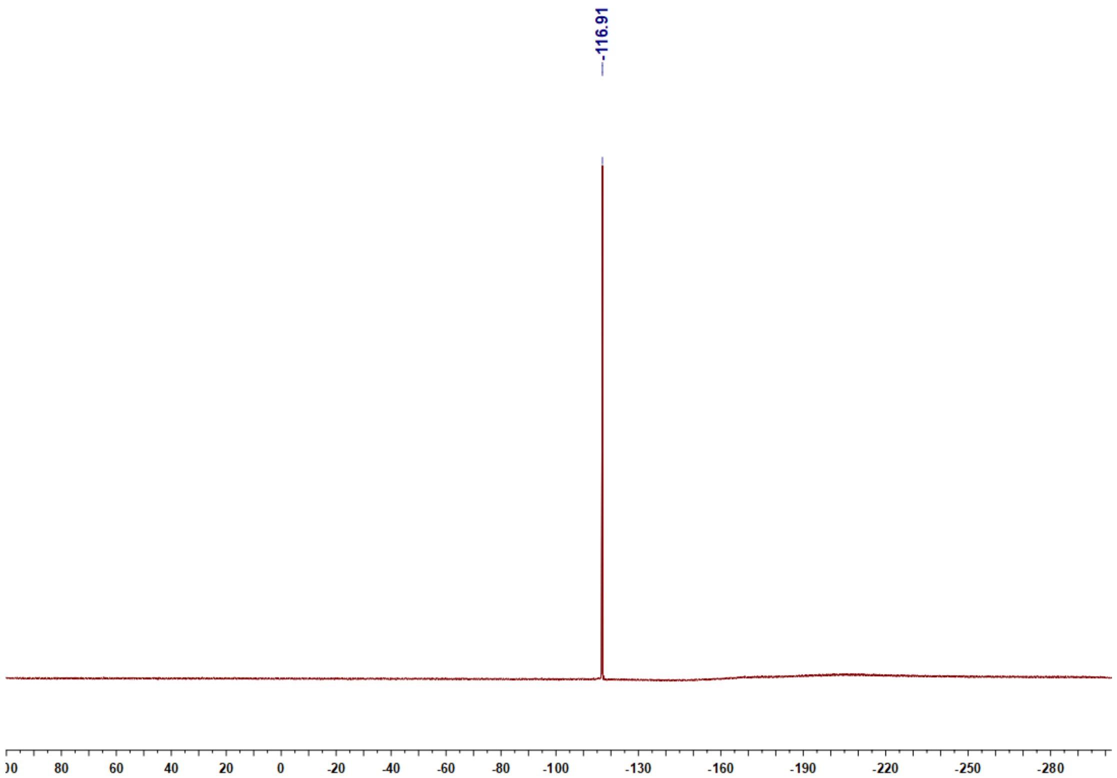

Figure S108. ^{19}F NMR (282 MHz, $CDCl_3$) spectrum of isolated **2s**

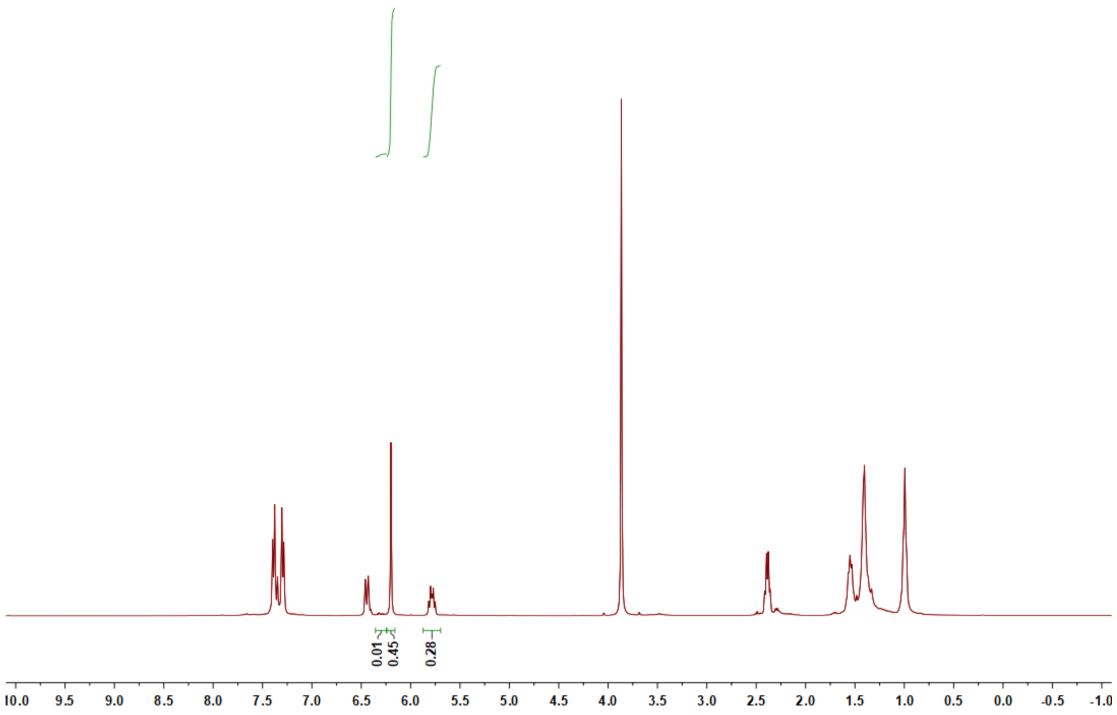

Figure S109. ^{1}H NMR (400 MHz, $CDCl_3$) spectrum of **2t**

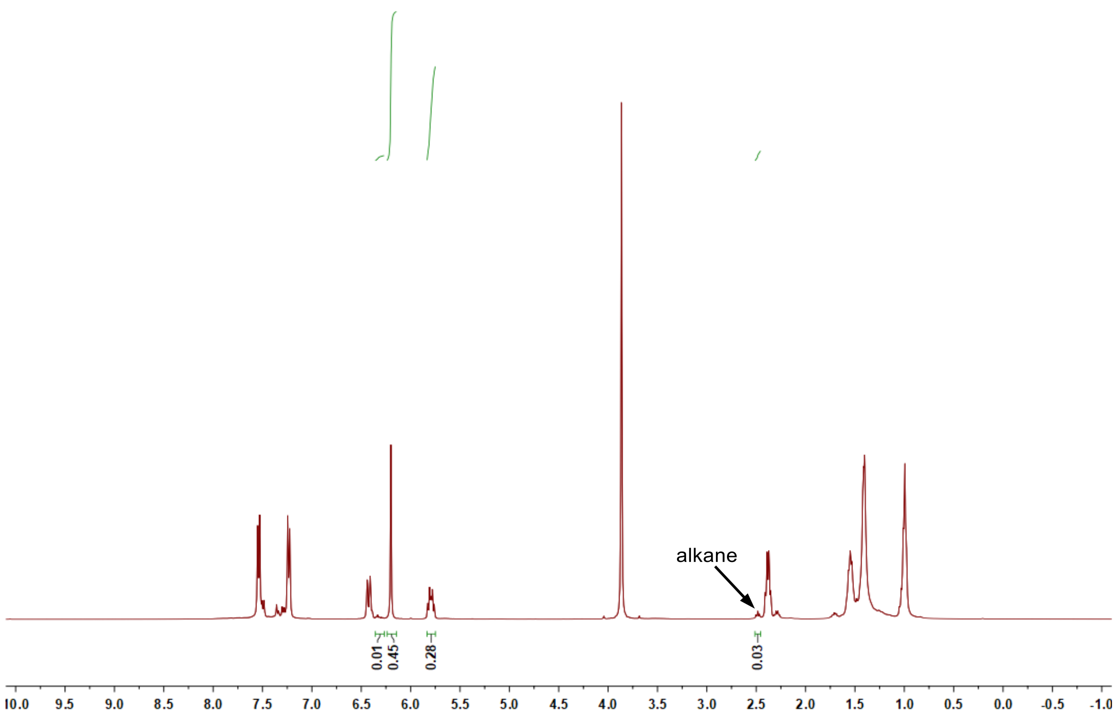

Figure S110. ^{1}H NMR (400 MHz, $CDCl_3$) spectrum of **2u**

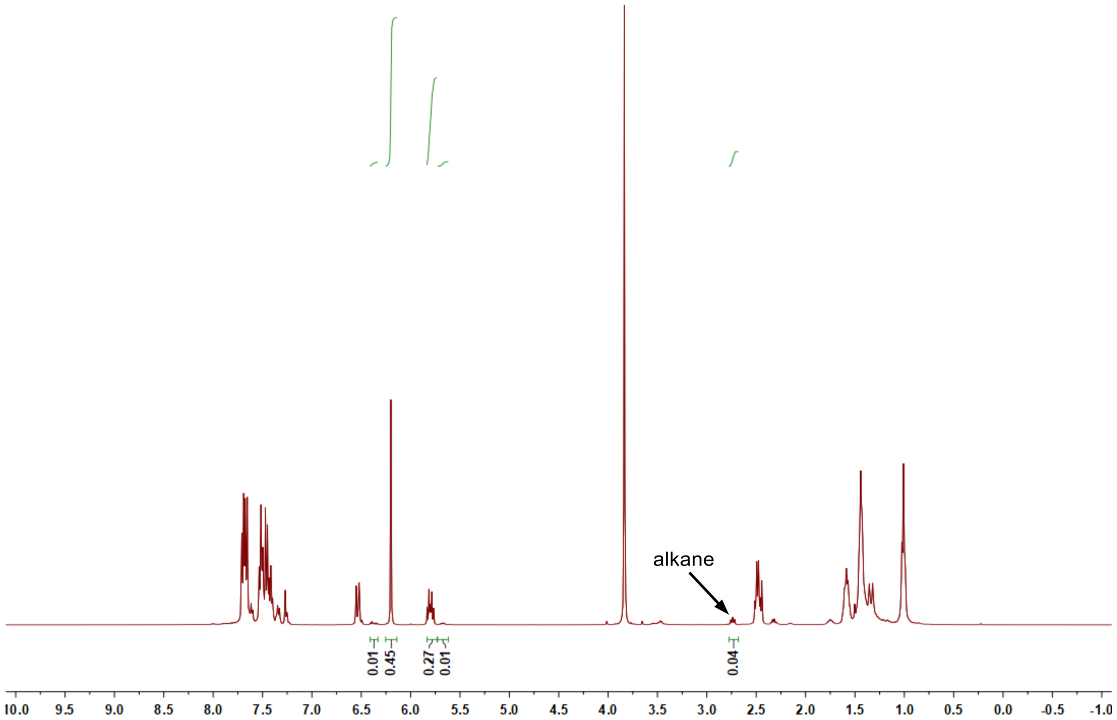

Figure S111. ^{1}H NMR (400 MHz, $CDCl_3$) spectrum of **2v**

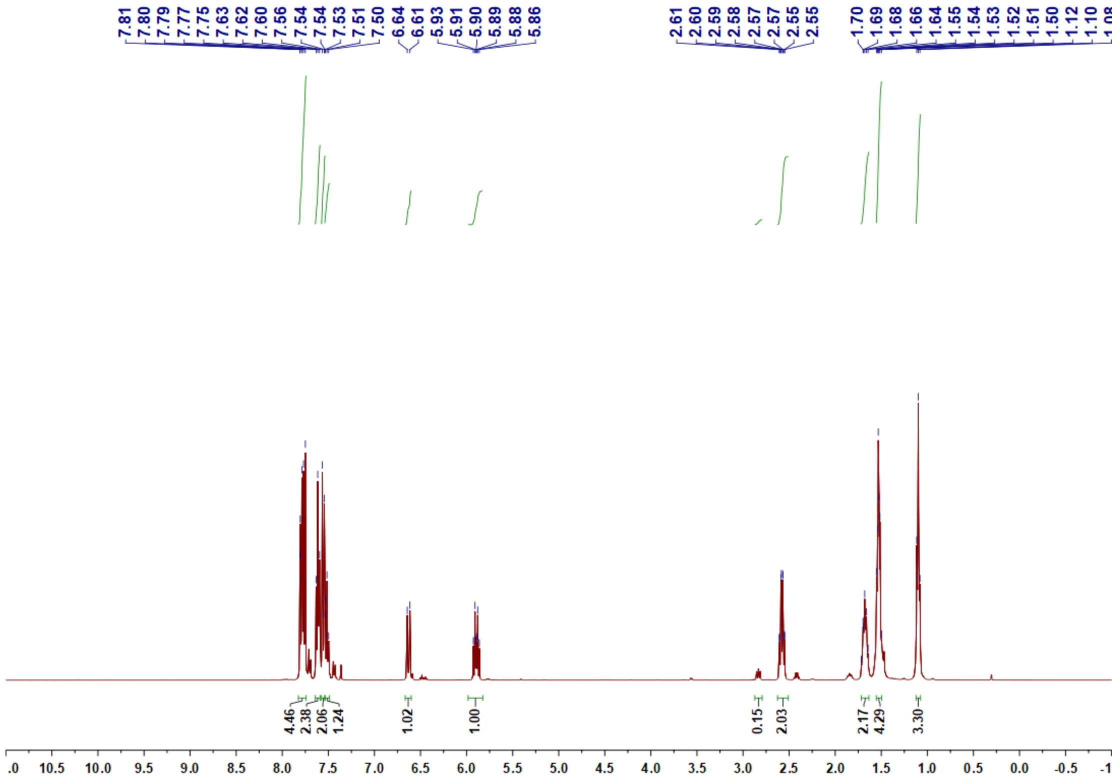

Figure S112. ^{1}H NMR (400 MHz, $CDCl_3$) spectrum of isolated **2v**

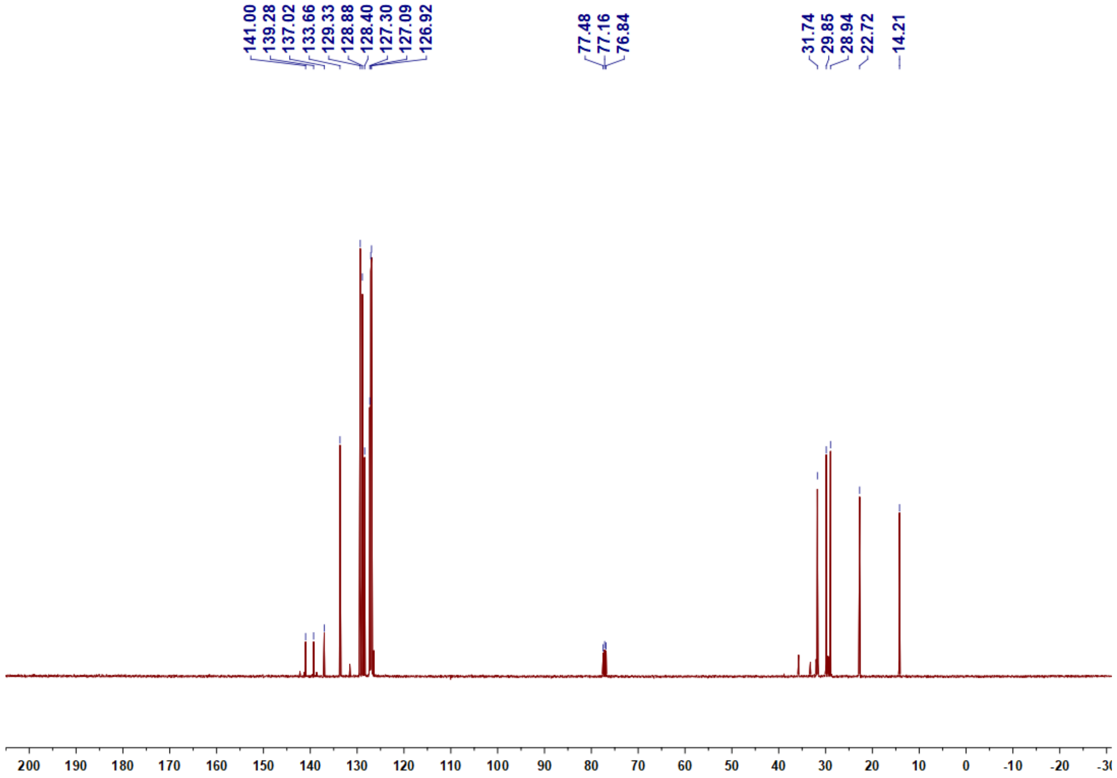

Figure S113. ^{13}C NMR (101 MHz, $CDCl_3$) spectrum of isolated **2v**

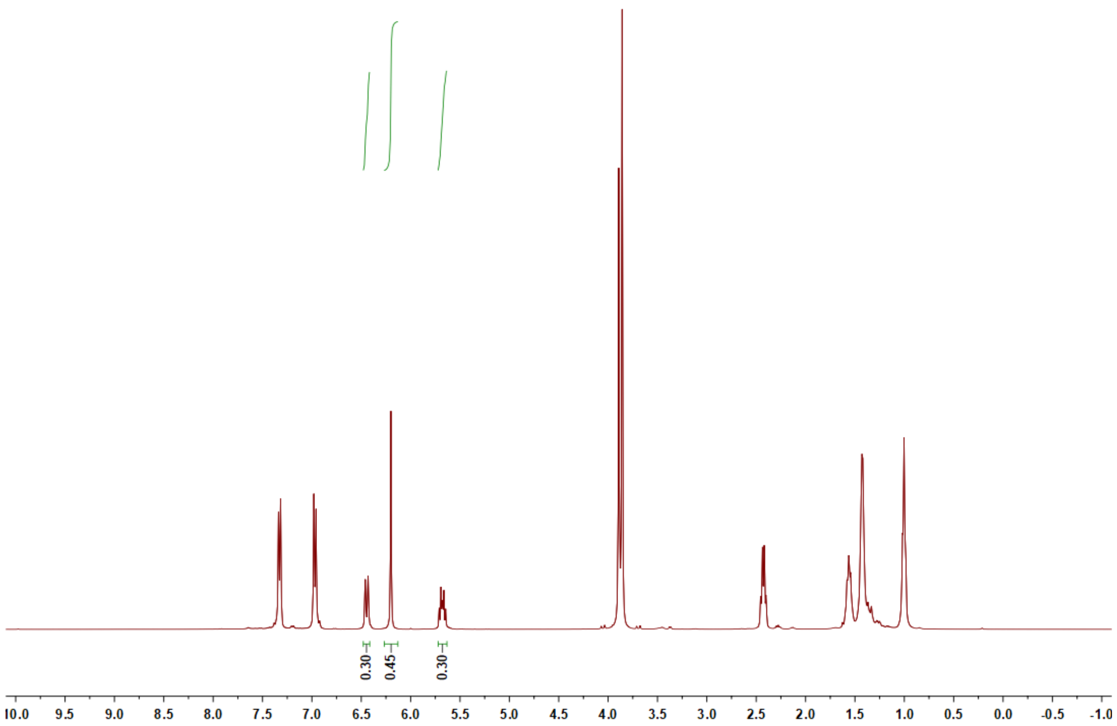

Figure S114. ^{1}H NMR (400 MHz, $CDCl_3$) spectrum of **2w**

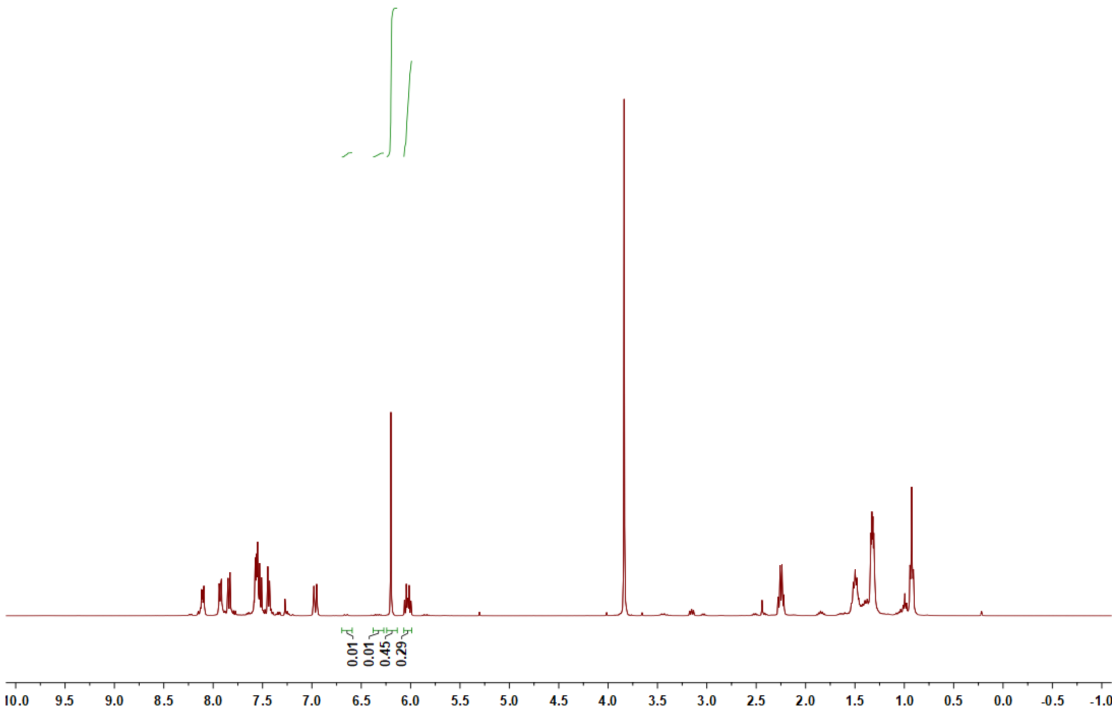

Figure S115. ^{1}H NMR (400 MHz, $CDCl_3$) spectrum of **2x**

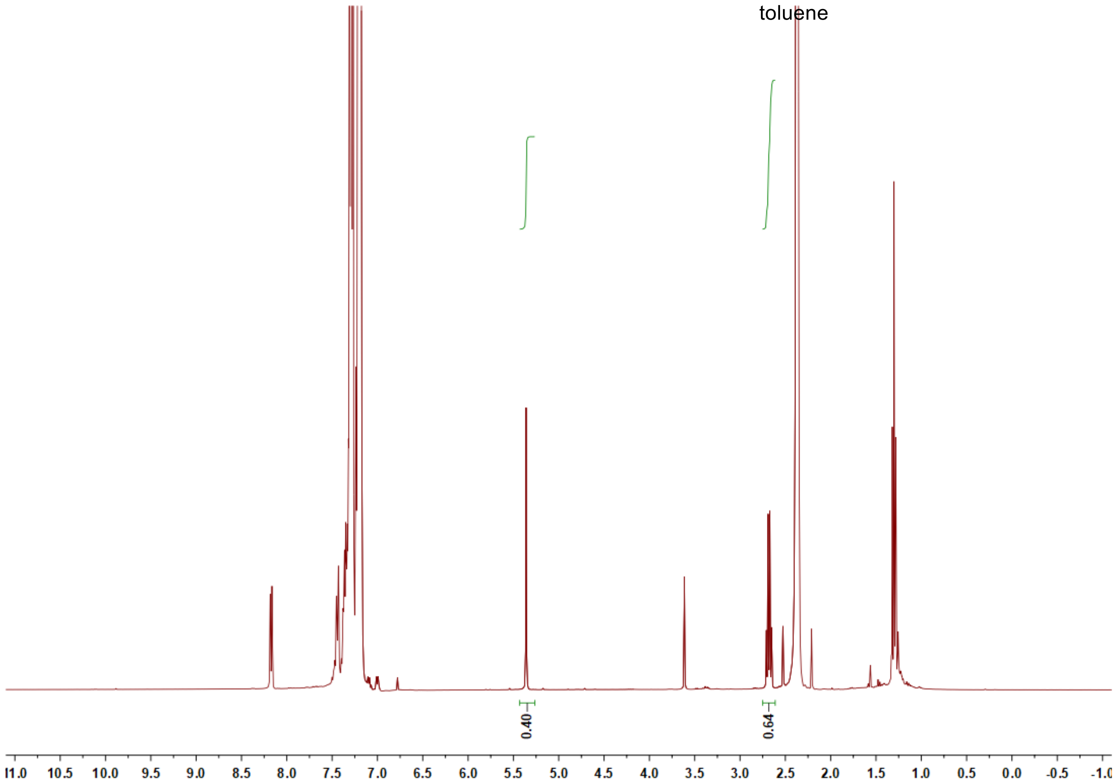

Figure S116. ^{1}H NMR (400 MHz, $CDCl_3$) spectrum of **4a**

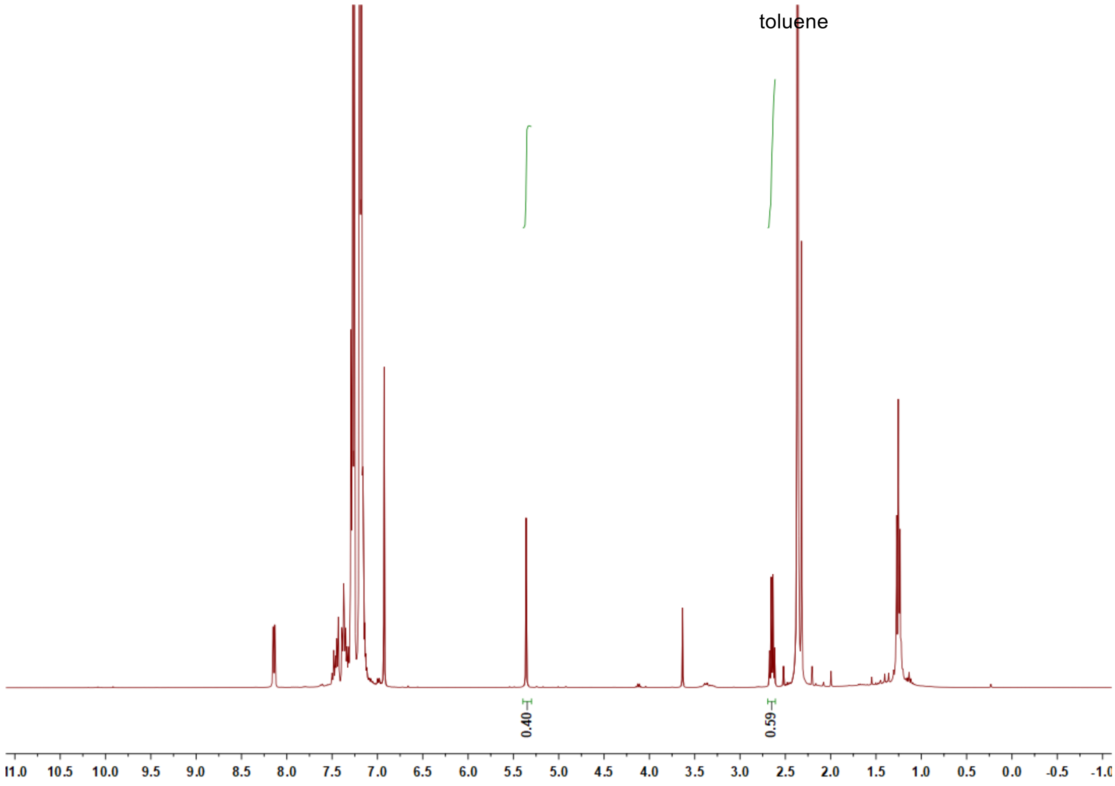

Figure S117. ^{1}H NMR (400 MHz, $CDCl_3$) spectrum of **4b**

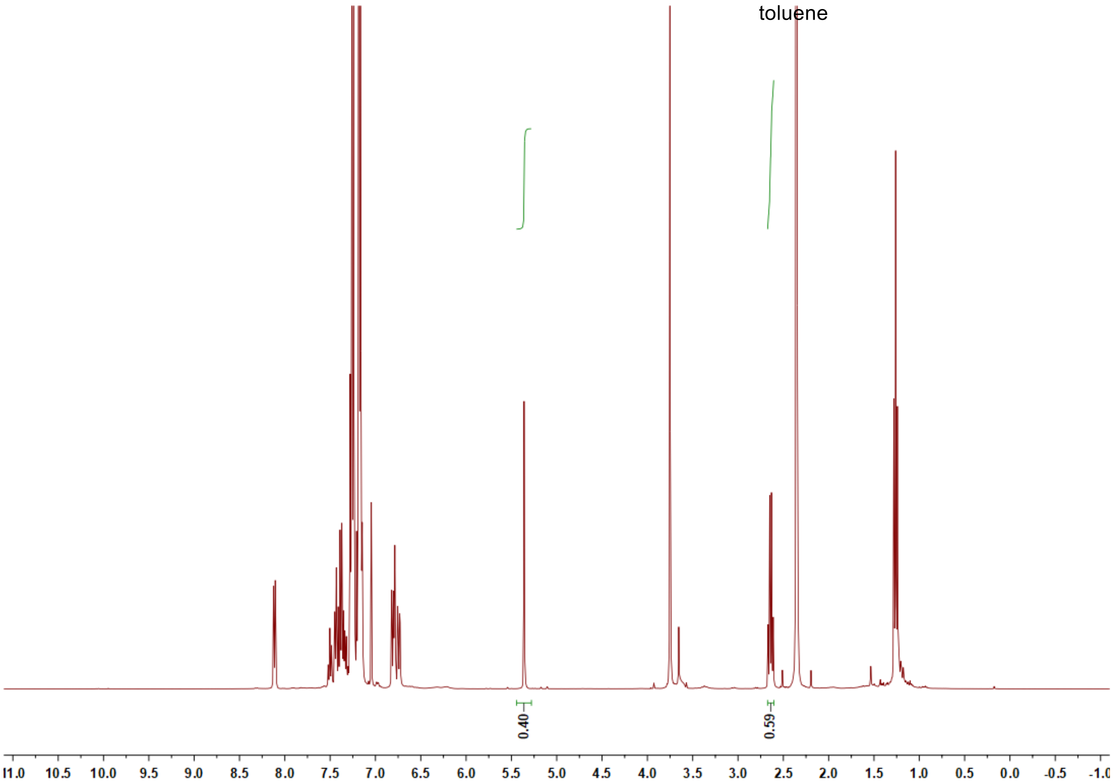

Figure S118. ^{1}H NMR (400 MHz, $CDCl_3$) spectrum of **4c**

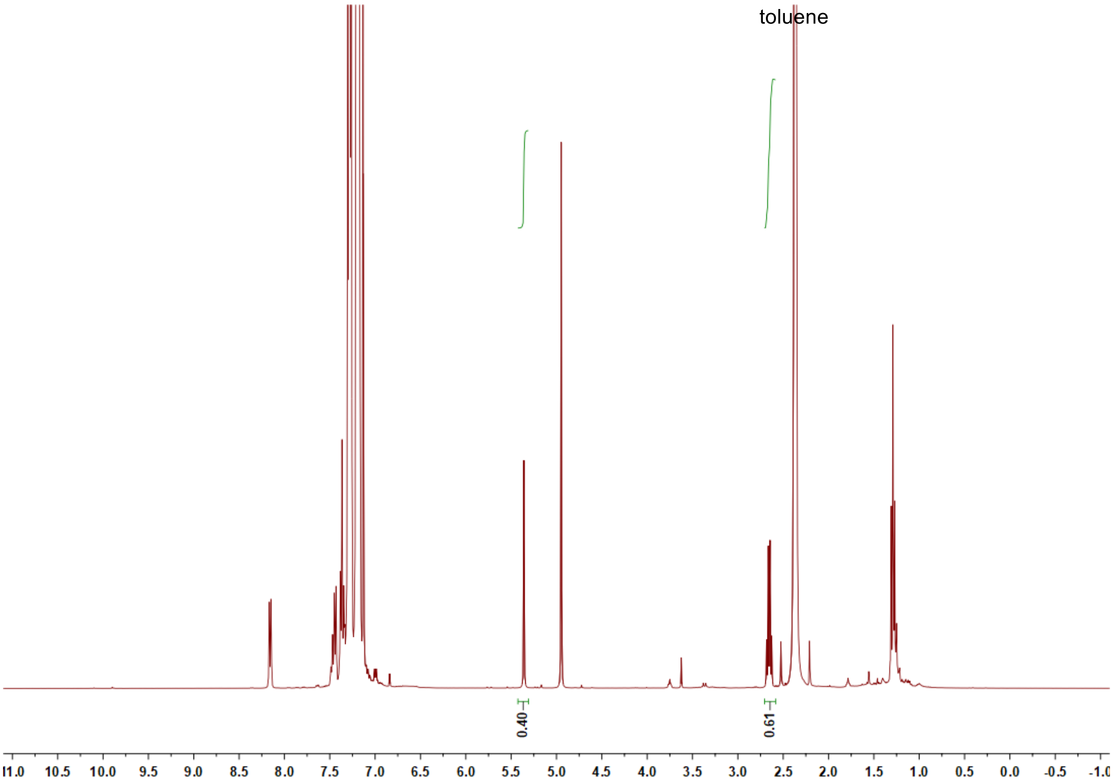

Figure S119. ^{1}H NMR (400 MHz, $CDCl_3$) spectrum of **4d**

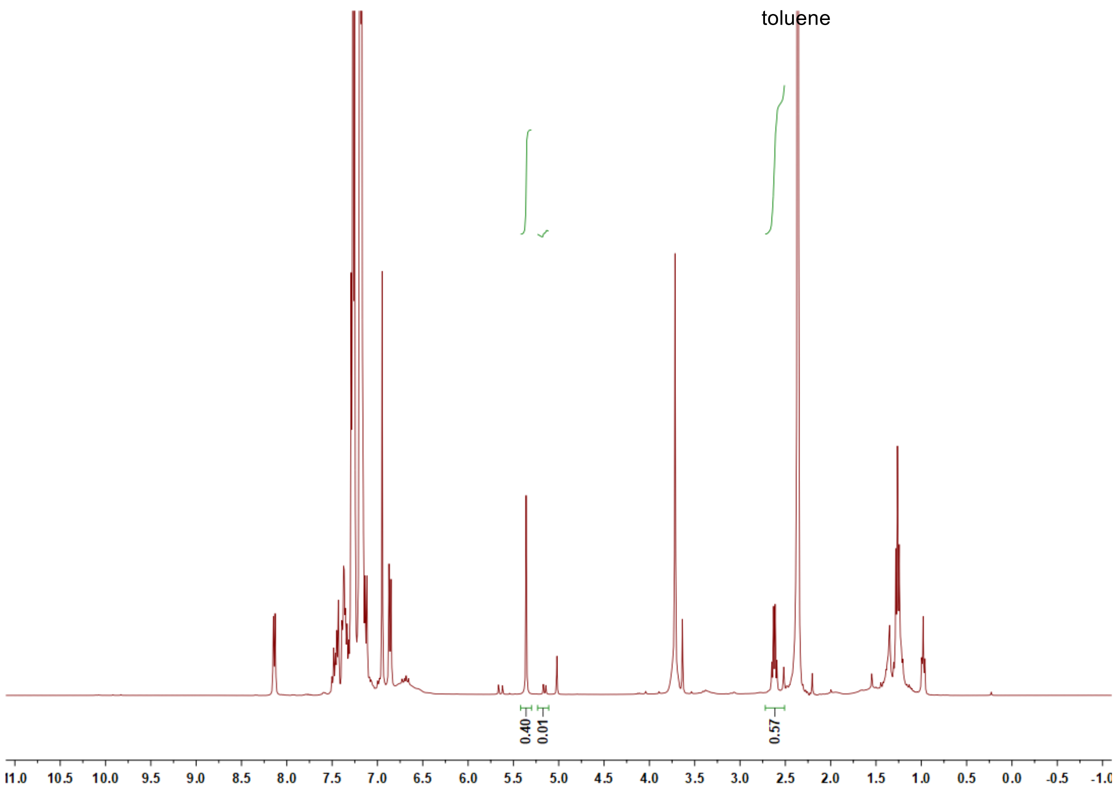

Figure S120. ^{1}H NMR (400 MHz, $CDCl_3$) spectrum of **4e**

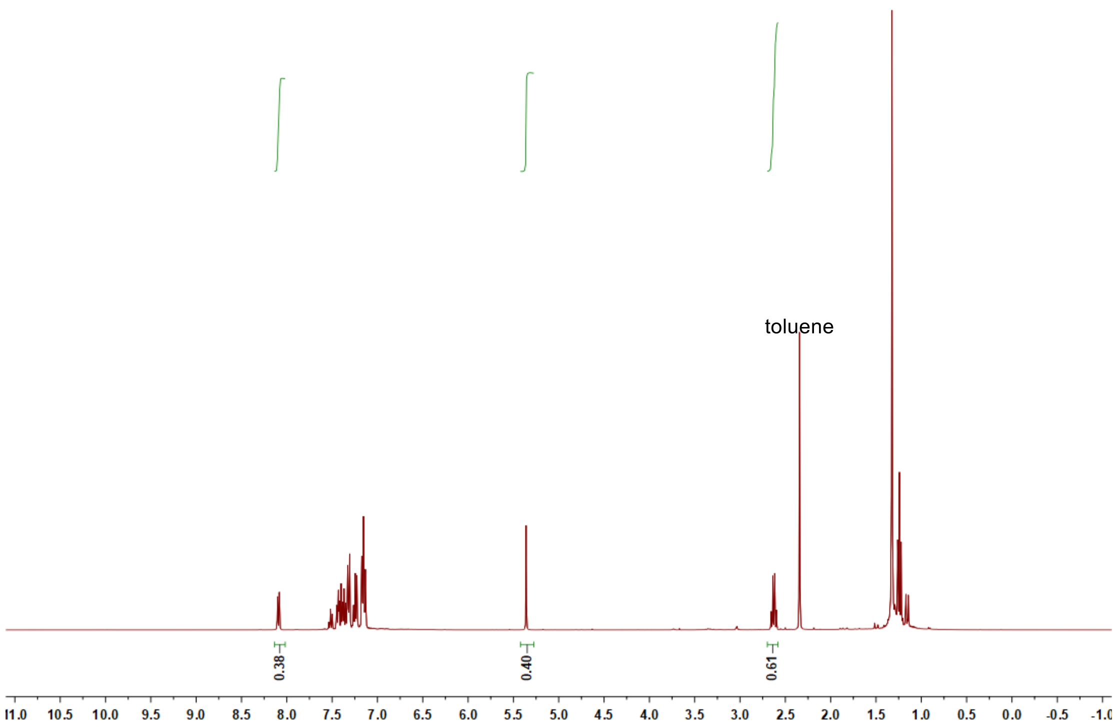

Figure S121. ^{1}H NMR (400 MHz, $CDCl_3$) spectrum of **4f**

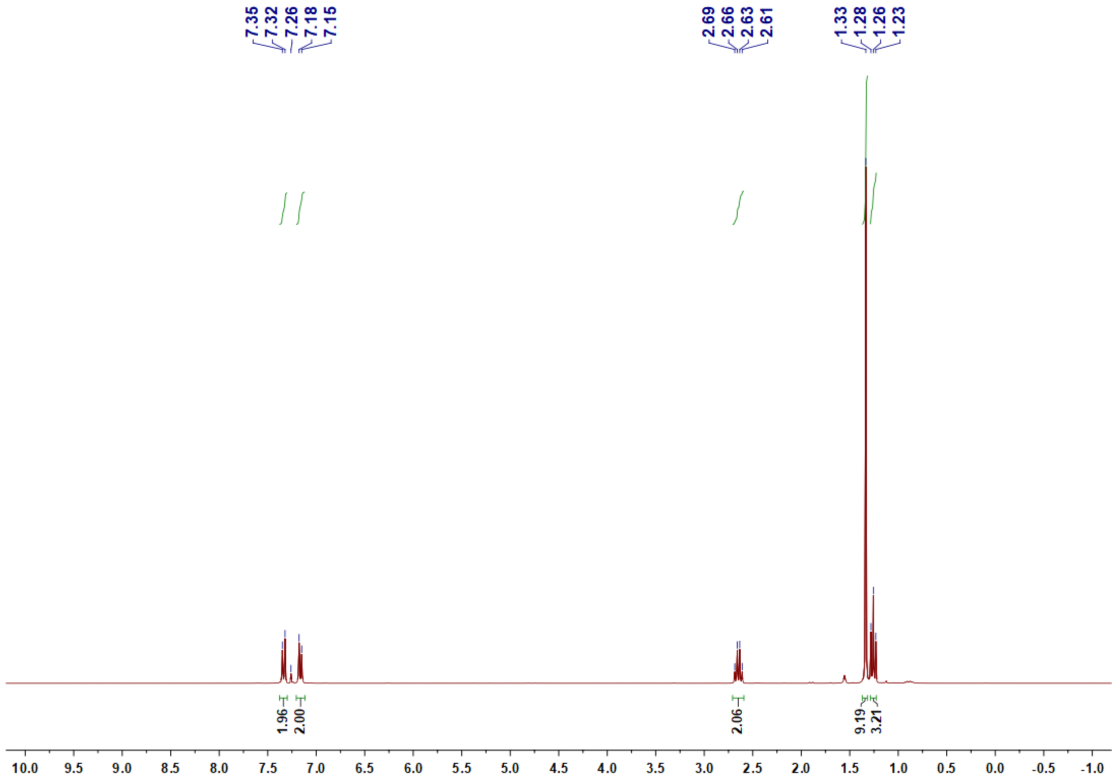

Figure S122. ^{1}H NMR (300 MHz, $CDCl_3$) spectrum of isolated **4f**

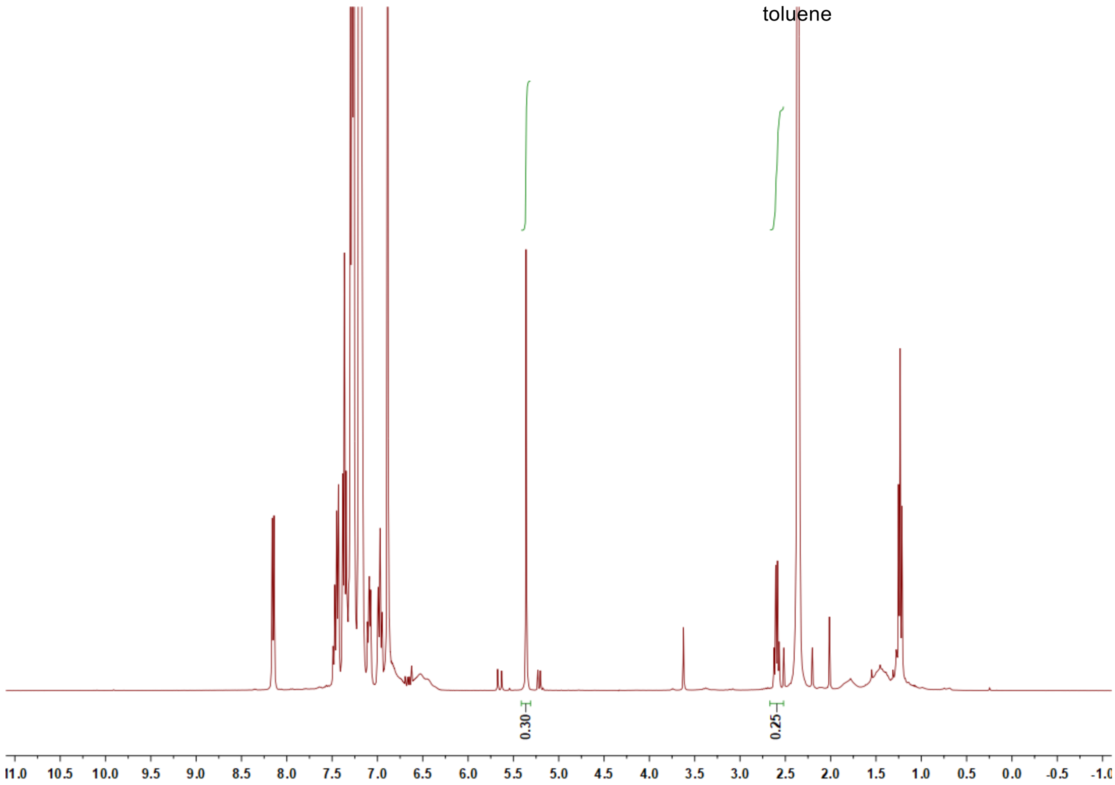

Figure S123. ^{1}H NMR (400 MHz, $CDCl_3$) spectrum of **4g**

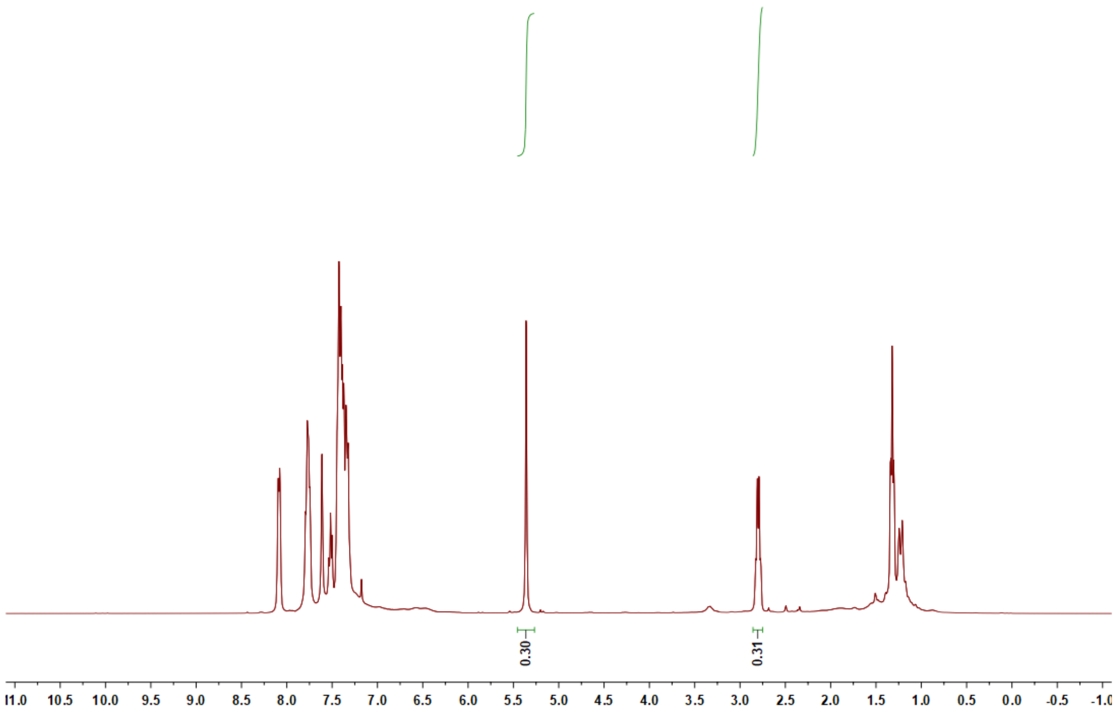

Figure S124. ^{1}H NMR (400 MHz, $CDCl_3$) spectrum of **4h**

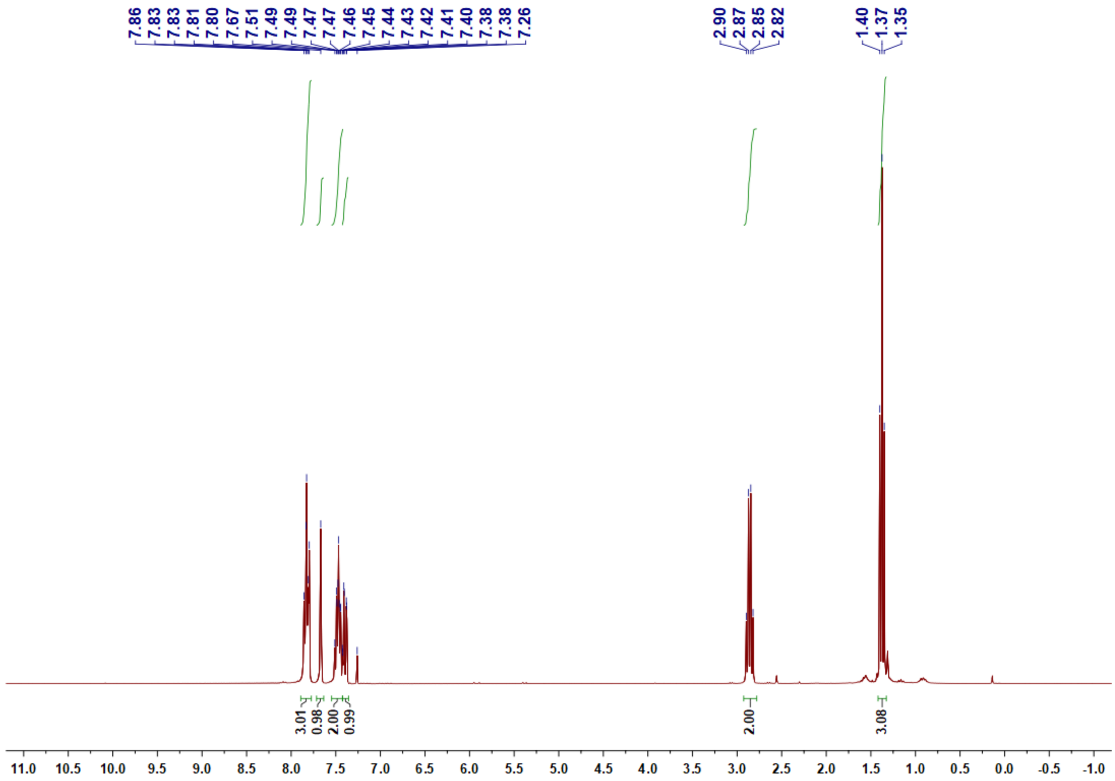

Figure S125. ^{1}H NMR (300 MHz, $CDCl_3$) spectrum of isolated **4h**

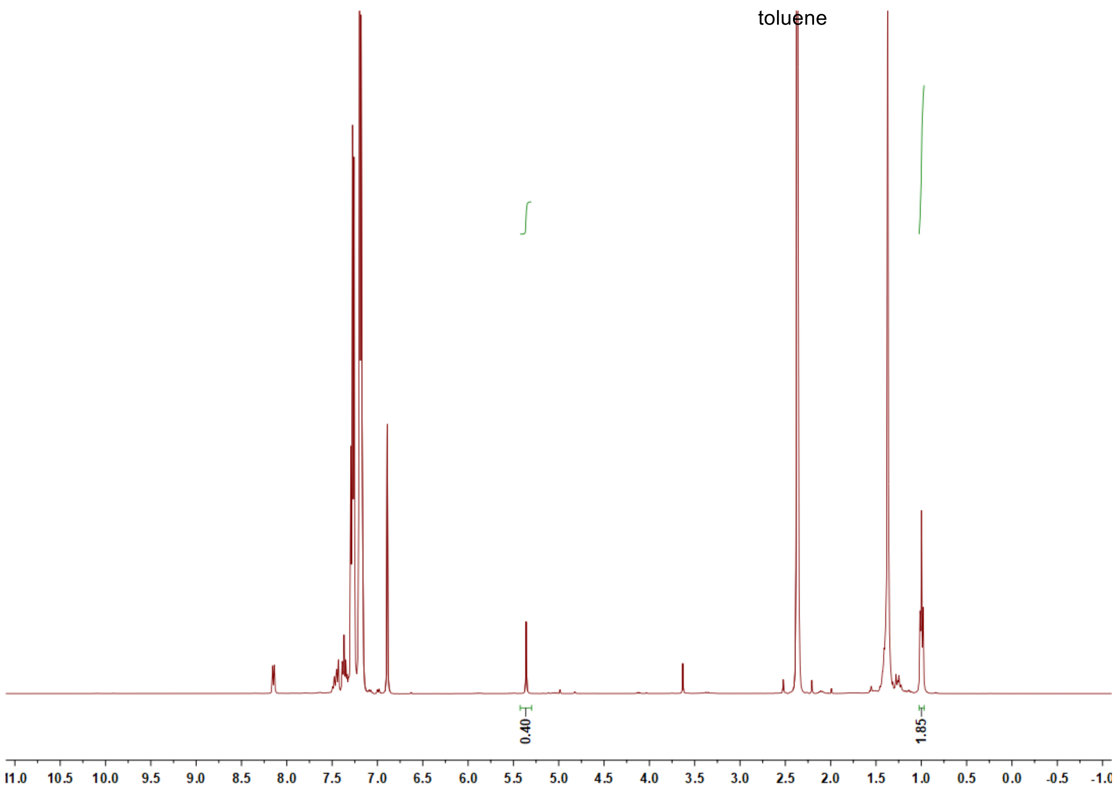

Figure S126. ^{1}H NMR (400 MHz, $CDCl_3$) spectrum of **4i**

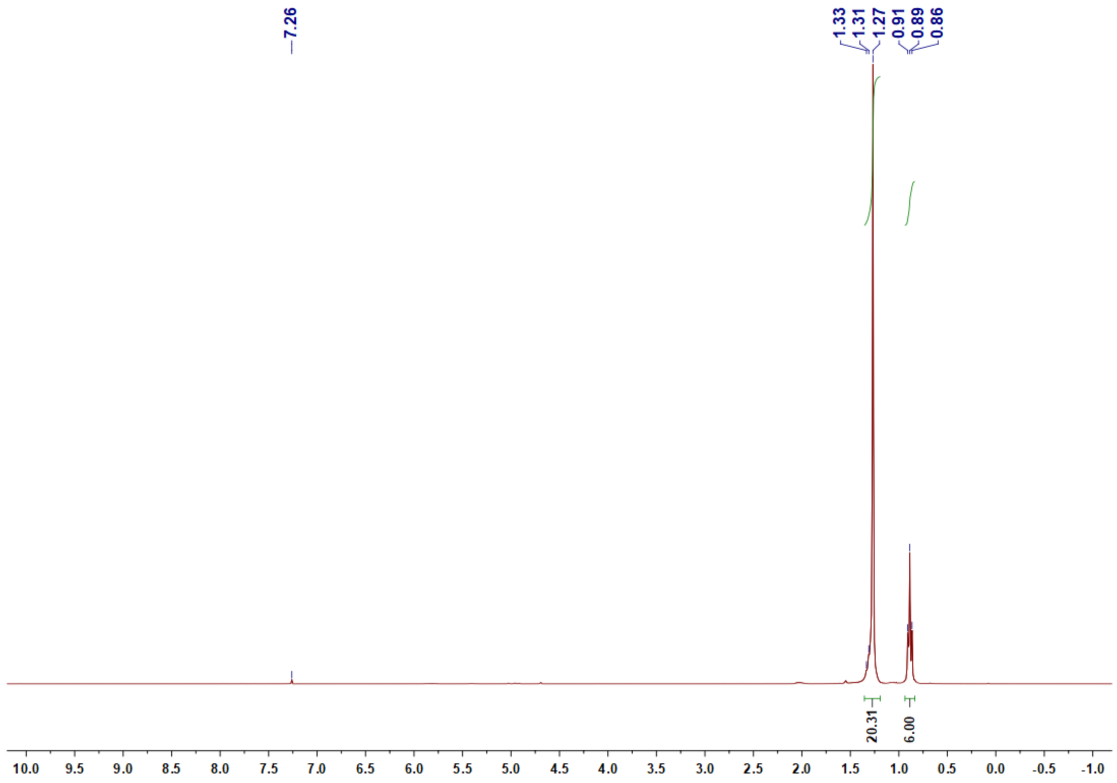

Figure S127. ^{1}H NMR (300 MHz, $CDCl_3$) spectrum of isolated **4i**

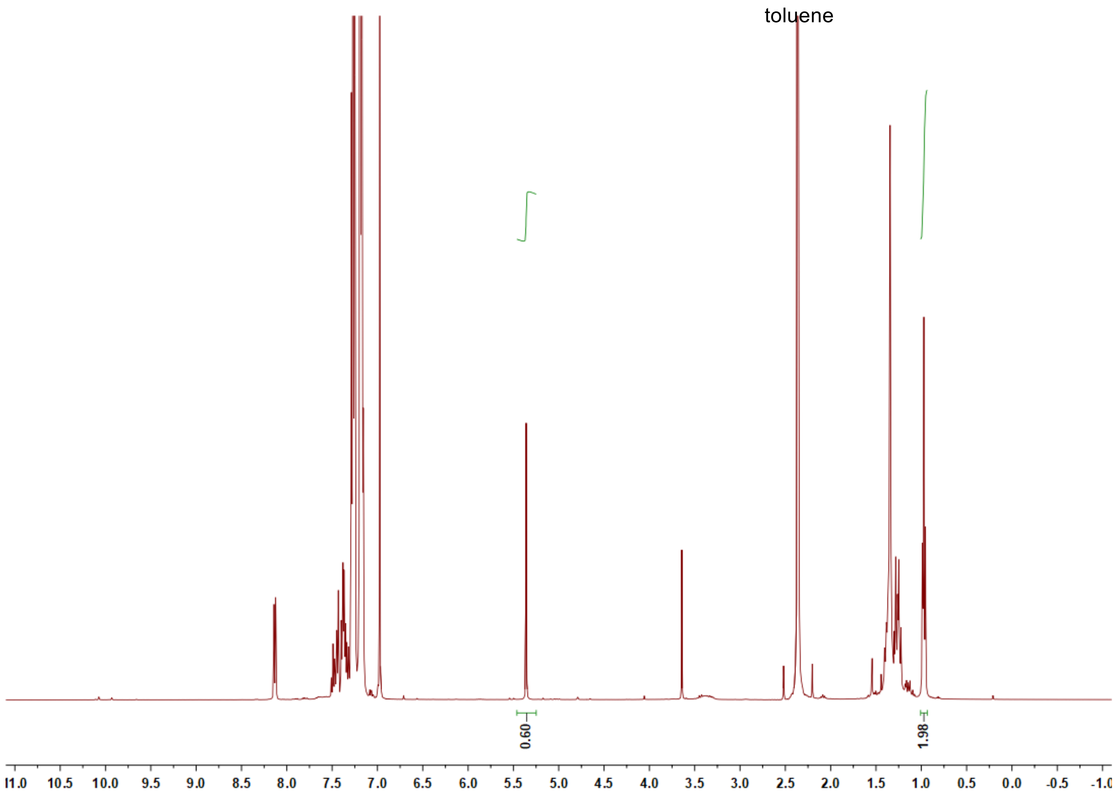

Figure S128. ^{1}H NMR (400 MHz, $CDCl_3$) spectrum of **4j**

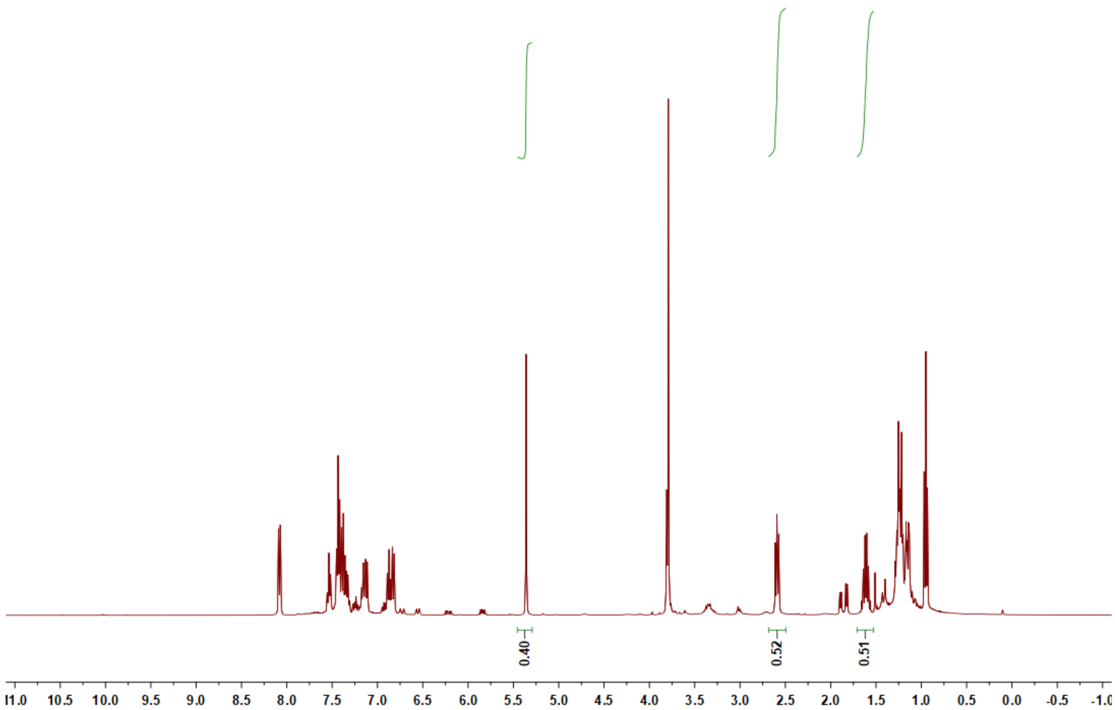

Figure S129. ^{1}H NMR (400 MHz, $CDCl_3$) spectrum of **4k**

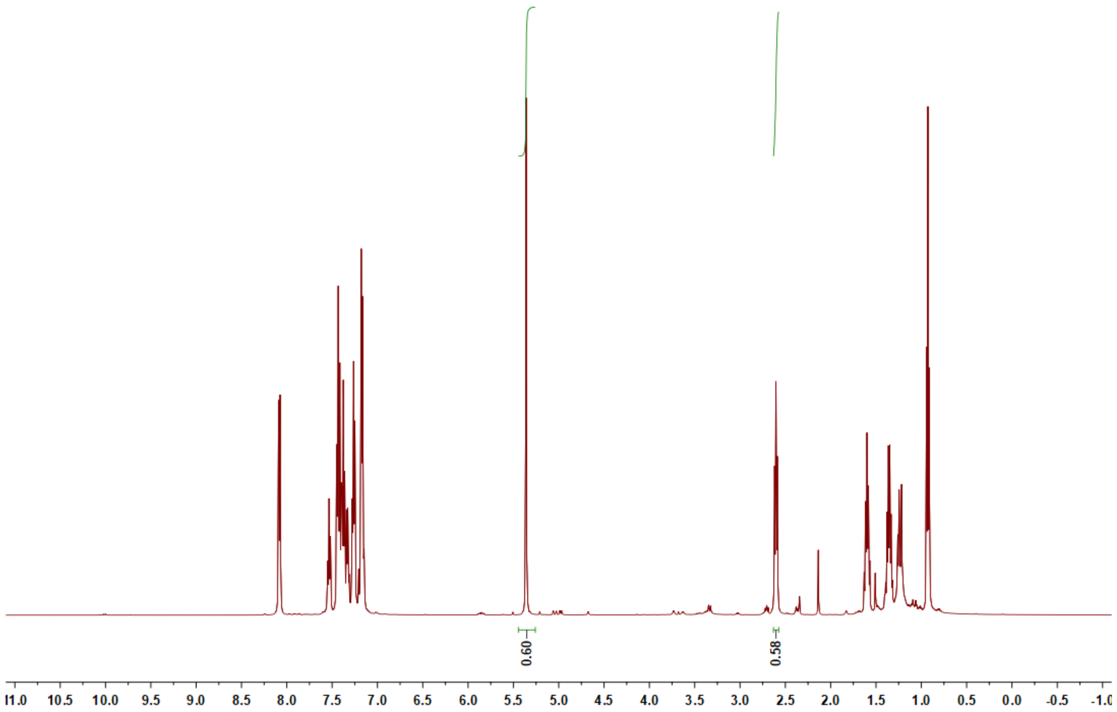

Figure S130. ^{1}H NMR (400 MHz, $CDCl_3$) spectrum of **4l**

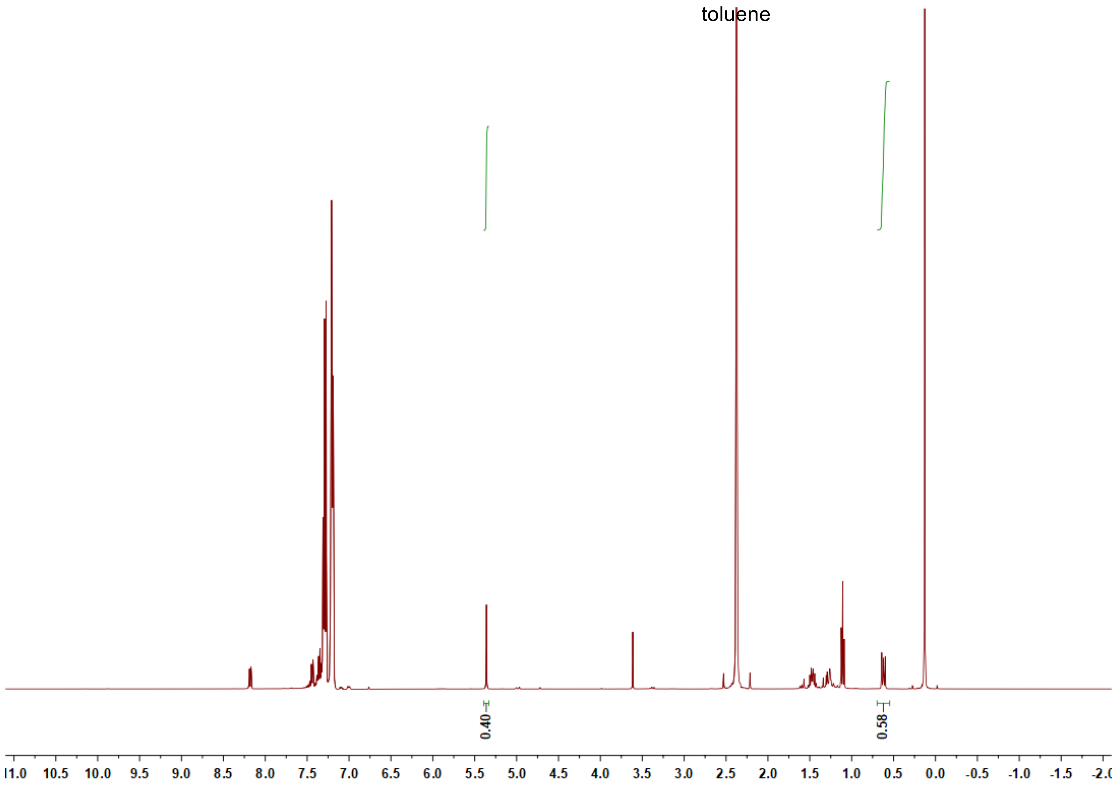

Figure S131. ^{1}H NMR (400 MHz, $CDCl_3$) spectrum of **4m**

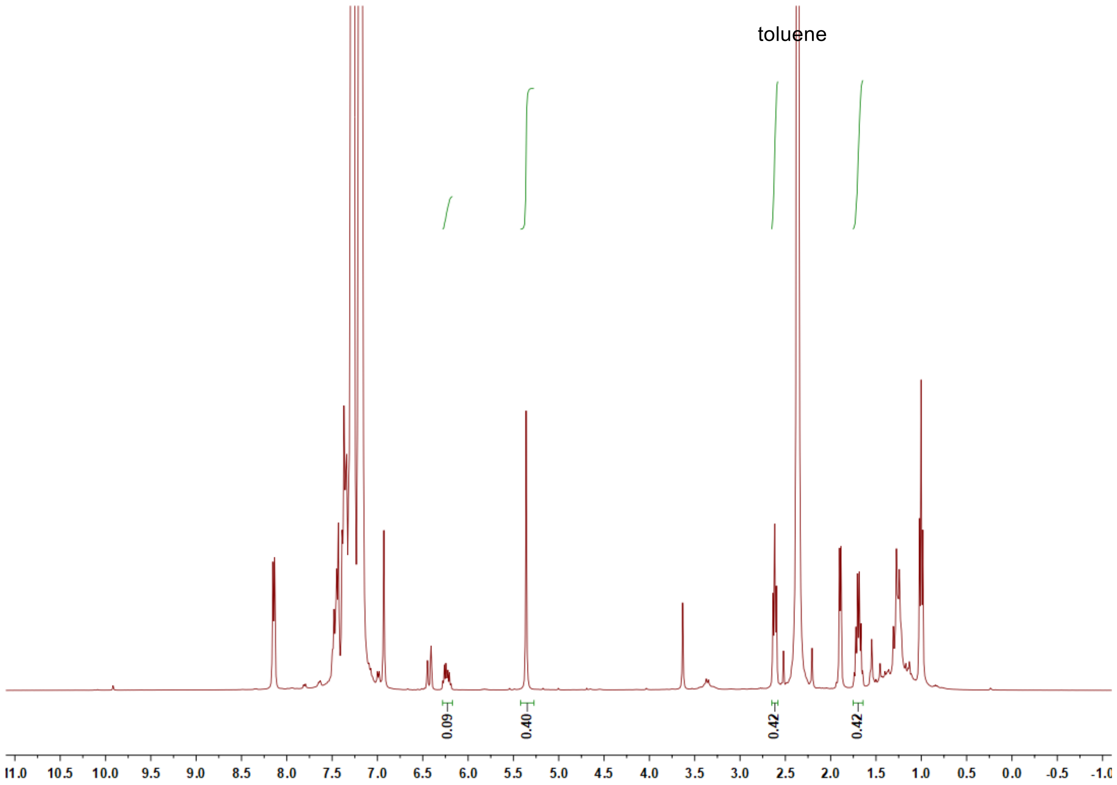

Figure S132. ^{1}H NMR (400 MHz, $CDCl_3$) spectrum of **4n**

9 References

1. Hermann, D.; Gandelman, M.; Rozenberg, H.; Shimon, L. J. W.; Milstein, D. Synthesis, Structure, and Reactivity of New Rhodium and Iridium Complexes, Bearing a Highly Electron-Donating PNP System. Iridium-Mediated Vinylic C-H Bond Activation. *Organometallics* **2002**, *21*, 812–818.
2. Zhang, J.; Leitus, G.; Ben-David, Y.; Milstein, D. Facile Conversion of Alcohols into Esters and Dihydrogen Catalyzed by New Ruthenium Complexes. *J. Am. Chem. Soc.* **2005**, *127*, 10840–10841.
3. Bai, R.; Liang, Z.; Yoon, Y.; Liu, S.; Caine, T.; Oum, Y.; Shi, Q.; Mooring, S. R.; Shim, H. Symmetrical Bis-Tertiary Amines as Novel CXCR4 Inhibitors. *Eur. J. Med. Chem.* **2016**, *118*, 340–350.
4. Johansson, A.; Hakansson, M. Absolute Asymmetric Synthesis of Stereochemically Labile Aldehyde Helicates and Subsequent Chirality Transfer Reactions. *Chem. Eur. J.* **2005**, *11*, 5238–5248.
5. Rigaku Corporation.

6. Sheldrick, G. M. *SHELXT*–Integrated Space-Group and Crystal-Structure Determinatio. *Acta Crystallogr A*, **2015**, *71*, 3–8.

7. Sheldrick, G. M. Crystal Structure Refinement with *SHELXL*. *Acta Crystallogr. C*, **2015**, *71*, 3–8.

8. Dolomanov, O. V.; Bourhis, L. J.; Gildea, R. J.; Howard, J. A. K.; Puschmann, H. *OLEX2*: A Complete Structure Solution, Refinement and Analysis Program. *J. Appl. Crystallogr*. **2009**, *42*, 339–341.

9. Gärtner, M.; Fischer, R.; Langer, J.; Görls, H.; Walther, D.; Westerhausen, M. Syntheses and Structures of Alkaline Earth Metal Bis(diphenylamides). *Inorg. Chem*. **2007**, *46*, 5118−5124.

10. Du, W.-B.; Wang, N.-N.; Pan, C.; Ni, S.-F.; Wen, L.-R.; Li, M.; Zhang, L.-B. Regio- and Stereoselective Electrochemical Synthesis of Sulfonylated Enethers from Alkynes and Sulfonyl Hydrazides. *Green Chemistry* **2021**, *23*, 2420−2426.

11. Cramer, C. J. *Essentials of Computational Chemistry: Theories and Models in 2nd edition*; John Wiley, and Sons Ltd: West Sussex, England, 2014.

12. Sparta, M.; Riplinger, C.; Neese, F. Mechanism of Olefin Asymmetric Hydrogenation Catalyzed by Iridium Phosphino-Oxazoline: A Pair Natural Orbital Coupled Cluster Study. *J. Chem. Theory and Computation* **2014**, *10*, 1099−1108.

13. Gaussian 16, Revision C.01, Frisch, M. J.; Trucks, G. W.; H. Schlegel, B.; Scuseria, G. E.; Robb, M. A.; Cheeseman, J. R.; Scalmani, G.; Barone, V.; Petersson, G. A.; Nakatsuji, H.; Li, X.; Caricato, M.; Marenich, A. V.; Bloino, J.; Janesko, B. G.; Gomperts, R.; Mennucci, B.; Hratchian, H. P.; Ortiz, J. V.; Izmaylov, A. F.; Sonnenberg, J. L.; Williams-Young, D.; Ding, F.; Lipparini, F.; Egidi, F.; Goings, J.; Peng, B.; Petrone, A.; Henderson, T.; Ranasinghe, D.; Zakrzewski, V. G.; Gao, J.; Rega, N.; Zheng, G.; Liang, W.; Hada, M.; Ehara, M.; Toyota, K.; Fukuda, R.; Hasegawa, J.; Ishida, M.; Nakajima, T.; Honda, Y.; Kitao, O.; Nakai, H.; Vreven, T.; Throssell, K.; Montgomery, Jr., J. A.; Peralta, J. E.; Ogliaro, F.; Bearpark, M. J.; Heyd, J. J.; Brothers, E. N.; Kudin, K. N.; Staroverov, V. N.; Keith, T. A.; Kobayashi, R.; Normand, J.; Raghavachari, K.; Rendell, A. P.; Burant, J. C.; Iyengar, S. S.; Tomasi, J.; Cossi, M.; Millam, J. M.; Klene, M.;

Adamo, C.; Cammi, R.; Ochterski, J. W.; Martin, R. L.; Morokuma, K.; Farkas, O.; Foresman, J. B.; Fox, D. J. Gaussian, Inc., Wallingford CT, 2016.

14. Zhao, Y.; Truhlar, D. G. A New Local Density Functional for Main-Group Thermochemistry, Transition Metal Bonding, Thermochemical Kinetics and Noncovalent Interactions. *J. Chem. Phys*. **2006**, *125*, 194101/1−18.
15. Weigend, F.; Ahlrichs, R. Balanced Basis Sets of Split Valence, Triple Zeta Valence and Quadruple Zeta Valence Quality for H to Rn: Design and Assessment of Accuracy. *Phys. Chem. Chem. Phys.* **2005**, *7*, 3297−3305.
16. Weigend, F. Accurate Coulomb-Fitting Basis Sets for H to Rn. *Phys. Chem. Chem. Phys*. **2006**, *8*, 1057−1065.
17. Grimme, S.; Antony, J.; Ehrlich, S.; Krieg, H. A Consistent and Accurate ab Initio Parametrization of Density Functional Dispersion Correction (DFT-D) for the 94 Elements HPu. *J. Chem. Phys.* **2010**, *132*, 154104/1−19.
18. Neese, F. Software Update: the ORCA Program System, Version 4.0. *WIREs Computational Molecular Science* **2018**, *8*, e1327−e1332.
19. Mardirossian, N.; Head-Gordon, M. ωB97X-V: A 10-Parameter, Range-Separated Hybrid, Generalized Gradient Approximation Density Functional with Nonlocal Correlation, Designed by a Survival-of-the-Fittest Strategy. *Phys. Chem. Chem. Phys.* **2014**, *16*, 9904−9924.
20. Vydrova, O. A.; Voorhis, T. V. Nonlocal van der Waals Density Functional: The Simpler the Better. *J. Chem. Phys.* **2010**, *133*, 244103/1−9.
21. Hujo, W.; Grimme, S. Performance of the van der Waals Density Functional VV10 and (Hybrid)GGA Variants for Thermochemistry and Noncovalent Interactions. *J. Chem. Theory Comput.* **2011**, *7*, 3866−3871.
22. Hellweg, A.; Hattig, C.; Hoefener, S.; Klopper, W. Optimized Accurate Auxiliary Basis Sets for RI-MP2 and RI-CC2 Calculations for the Atoms Rb to Rn. *Theor. Chem. Acc.* **2007**, *117*, 587−597.
23. Iron, M. A.; Janes, T. Evaluating Transition Metal Barrier Heights with the Latest Density Functional Theory Exchange-Correlation Functionals: The MOBH35 Benchmark Database. *J. Phys. Chem. A* **2019**, *123*, 3761−3781.

24. Marenich, A. V.; Cramer, C. J.; Truhlar, D. G. Universal Solvation Model Based on Solute Electron Density and on a Continuum Model of the Solvent Defined by the Bulk Dielectric Constant and Atomic Surface Tensions. *J. Phys. Chem. B* **2009**, *113*, 6378−6396.
25. Hopmann, K. H. How Accurate is DFT for Iridium-Mediated Chemistry? *Organometallics* **2016**, *35*, 3795−3807.
26. Gusev, D. G. Revised Mechanisms of the Catalytic Alcohol Dehydrogenation and Ester Reduction with the Milstein PNN Complex of Ruthenium. *Organometallics* **2020**, *39*, 258−270.
